# Erectile dysfunction, type 2 diabetes, and cardiovascular disease: a narrative review and insights from a global real-world cohort analysis

**DOI:** 10.3389/fcdhc.2026.1781581

**Published:** 2026-03-06

**Authors:** Santiago Martínez Mores, Josep Franch-Nadal, Didac Mauricio, Bogdan Vlacho

**Affiliations:** 1 Department of Endocrinology & Nutrition, IR SANT PAU, Hospital de la Santa Creu i Sant Pau Institut de Recerca Sant Pau (IR SANT PAU), Barcelona, Spain; 2 Departamento de Medicina, Facultad de Medicina y Ciencias de la Salud, Universitat de Barcelona, Barcelona, Spain; 3 Grup de Recerca Epidemiològica en Diabetis des de l´Atenció Primària (DAP_CAT) group, Unitat de Suport a la Recerca Barcelona, Fundació Institut Universitari per a la recerca a l’Atenció Primària de Salut Jordi Gol i Gurina (IDIAPJGol), Barcelona, Spain; 4 CIBER de Diabetes y Enfermedades Metabólicas Asociadas, Instituto de Salud Carlos III-Centro de Investigación Biomédica en Red de Diabetes y Enfermedades Metabólicas Asociadas, Instituto de Salud Carlos III, Madrid, Spain; 5 Faculty of Medicine, University of Vic/Central University of Catalonia (UVIC/UCC), Vic, Spain

**Keywords:** cardiovascular disease, cardiovascular risk, endothelial dysfunction, erectile dysfunction, real-world data, type 2 diabetes mellitus

## Abstract

**Background:**

Erectile dysfunction (ED) is highly prevalent among men with type 2 diabetes mellitus (T2DM) and reflects systemic vascular and metabolic dysfunction. Shared mechanisms include endothelial dysfunction, oxidative stress, inflammation, autonomic neuropathy, and hypogonadism. Therefore, ED may function not only as a complication of T2DM but also as an early clinical marker of cardiometabolic disease.

**Objective:**

This narrative review summarizes current evidence on the epidemiology, mechanisms, and cardiometabolic implications of ED in men with T2DM, and evaluates the impact of major cardiometabolic therapies on erectile function. A real-world cohort study was conducted using the TriNetX Global Collaborative Network, a large federated electronic health record database comprising healthcare organizations across multiple countries.

**Content:**

ED is closely linked with hypertension, obesity, dyslipidaemia, heart failure, ischemic heart disease, and stroke in men with T2DM, reflecting shared microvascular and macrovascular diseases. Cohort and real-world studies indicate a strong bidirectional relationship: poor cardiometabolic control worsens erectile function, whereas improvements in glycaemia, weight, blood pressure, and lipids are associated with higher International Index of Erectile Function (IIEF) scores. Using data from the TriNetX Global Collaborative Network, large-scale real-world analyses further demonstrate that the coexistence of ED and T2DM substantially increases cardiovascular risk. In a propensity-score-matched cohort (>200,000 individuals per group), men with ED and T2DM had higher risks of ischemic heart disease (15.7% vs. 11.5%, OR: 1.44; 95% CI 1.41-1.46), stroke (OR 1.45; 95% CI 1.42-1.48), peripheral artery disease (OR 1.38; 95% CI 1.35-1.41), and heart failure (8.4% vs. 4.9%, OR 1.78; 95% CI 1.74-1.81). Conversely, among individuals with T2DM, the presence of ED was associated with increased ischemic heart disease, stroke, and peripheral artery disease. Mechanistic and clinical data suggest heterogeneous treatment effects: GLP-1 receptor agonists and SGLT2 inhibitors show promising vascular benefits with mixed erectile outcomes, whereas ARBs and finerenone appear favorable compared with older agents associated with sexual adverse effects.

**Conclusion:**

ED in T2DM should be regarded as a clinically relevant marker of systemic vascular disease. Routine assessment may enhance cardiovascular risk stratification and motivate earlier, comprehensive risk-factor intervention. Future prospective and randomized studies with erectile function as a predefined endpoint are needed.

## 1 Introduction

Type 2 diabetes mellitus (T2DM) currently represents one of the most important global health challenges, with a rising prevalence in the coming decades (1). International data suggests that approximately 540 million people were living with T2DM in 2021, a number expected to rise to 643 million worldwide by 2030. Although T2DM affects both sexes, its prevalence is particularly high among men and middle-aged individuals, with risk increasing progressively with age (2).

T2DM is a leading cause of end-stage renal disease, blindness, and non-traumatic lower-limb amputations worldwide, reflecting its well-established microvascular burden (3). In parallel, diabetes is a major risk factor for cardiovascular disease (CVD), both independently and through its frequent coexistence with other cardiometabolic abnormalities such as dyslipidaemia and hypertension, making macrovascular complications the leading cause of morbidity and mortality in individuals with T2DM (4). However, this conventional micro- and macrovascular classification only partially reflects the complexity of T2DM, which should be regarded as a systemic, multisystem disease characterized by widespread metabolic, microcirculatory, and neural dysfunction affecting multiple organs (4).

Penile erection is a multifaceted physiological process that relies on the coordinated function of the vascular endothelium, intact neural pathways, and sufficient hormonal signaling (5). At the vascular level, erection is characterized by increased arterial inflow and relaxation of cavernous smooth muscle, which facilitates blood accumulation within the corpora cavernosa and restricts venous outflow (5). Nitric oxide (NO), released from endothelial cells and non-adrenergic, non-cholinergic nerves, serves as the principal mediator by initiating intracellular signaling cascades involving cyclic guanosine monophosphate (cGMP) and cyclic adenosine monophosphate (cAMP), ultimately resulting in smooth muscle relaxation (6–9). Impairment of endothelial integrity, neural signaling, or hormonal regulation can compromise erectile function. This physiological context forms the basis for understanding how metabolic and vascular disturbances in T2DM contribute to ED and its strong association with CVD (10, 11).

Erectile dysfunction is defined as the persistent inability to achieve or maintain an erection sufficient for satisfactory sexual performance (12). The European Association of Urology (EAU) classifies ED according to its underlying pathophysiological mechanisms, as outlined in **Table 1**. Etiologically, ED is typically categorized into three groups: organic, psychogenic, and mixed. Although most cases are considered mixed in origin, a simplified classification distinguishing primarily organic from primarily psychogenic ED has also been suggested (13). In men with T2DM, multiple mechanisms interact, with organic causes, particularly vascular, neurological, and hormonal disturbances, being predominant (13). ED in T2DM illustrates the systemic and multifactorial nature of the disease, arising from the interplay of vascular, neural, hormonal, metabolic, and genetic factors (14). These combined alterations extend beyond classical endothelial dysfunction and contribute to the heterogeneous clinical presentation and variable therapeutic response observed in diabetes-related ED. Among these mechanisms, endothelial dysfunction is widely recognized as the central pathophysiological link between ED and both T2DM and CVD (15).

**Table 1:** Classification of erectile dysfunction.

Vasculogenic	Recreational habits (e.g., smoking)
	Lack of regular physical exercise
	Obesity
	Cardiovascular diseases (e.g. hypertension, coronary artery disease, peripheral vascular disease)
	Diabetes mellitus type 1 and 2; hyperlipidemia; metabolic syndrome; hyperhomocysteinemia
	Major pelvic surgery (e.g., radical prostatectomy) or radiation therapy (pelvis or retroperitoneum)
Neurogenic	Core causes
	Degenerative disorders (e.g. multiple sclerosis, Parkinson’s disease, multiple atrophy, etc.)
	Trauma or spinal cord disease
	Stroke
	Tumours of the central nervous system
Peripheral causes	Diabetes mellitus types 1 and 2
	Chronic kidney failure, chronic liver failure
	Polyneuropathy
	Surgery (major pelvic/retroperitoneum surgery) or radiotherapy (pelvis or retroperitoneum)
	Urethral surgery (urethral stricture, open urethroplasty, etc.)
Anatomical or structural	Hypospadias, epispadias; micropenis
	Phimosis
	Peyronie’s disease
	Penile cancer (other tumours of the external genitalia)
Hormonal	Diabetes mellitus; Metabolic syndrome (MeTS)
	Hypogonadism (any type)
	Hyperthyroidism
	Hyper- and hypocortisolism (Cushing’s disease, etc.)
	Panhypopituitarism and multiple endocrine disorders
Mixed pathophysiological pathways	Chronic systemic diseases (e.g., diabetes mellitus, hypertension, MeTS, chronic kidney disease, chronic liver disorders, hyperhomocysteinemia, hyperuricemia, chronic obstructive pulmonary disease, rheumatic disease)
	Psoriasis, gouty arthritis, ankylosing spondylitis, non-alcoholic fatty liver disease, chronic periodontitis, open-angle glaucoma, inflammatory bowel disease, chronic fatigue syndrome, allergic rhinitis, obstructive sleep apnea, depression
	Iatrogenic causes (e.g., transrectal ultrasound-guided prostate biopsy)
Drug-induced causes	Drug-induced Antihypertensive drugs (i.e., thiazide diuretics, beta-blockers)
	Antidepressants (e.g., selective serotonin reuptake inhibitors, tricyclics)
	Antipsychotics
	Antiandrogens (GnRH analogues and antagonists; 5-ARI)
	Recreational drugs (e.g., heroin, cocaine, marijuana, methadone, synthetic drugs, anabolic steroids, excessive alcohol use)
Psychogenic	Generalized type (e.g., lack of arousal and sexual intimacy disorders)
	Situational type (e.g., relationship-related, performance-related, or distress-related problems)
Trauma	Penis fracture
	Pelvic fracture

Reproduced from the European Academy of Urology Guide (5).

Although substantial literature exists on ED in T2DM and CVD, these conditions are often studied independently. Notably, ED remains neglected as a clinical marker of cardiometabolic risk in men with T2DM. This narrative review integrates mechanistic evidence with recent epidemiological findings, including large-scale real-world data, to provide a comprehensive perspective on ED as an early indicator of cardiovascular disease in this population.

## 2 Methods

### 2.1 Literature search strategy and selection criteria

This narrative review includes epidemiological, pathophysiological, and clinical studies addressing the relationship among ED, T2DM, and CVD. While not a systematic review, it was conducted following established guidance for narrative review and incorporated a structured literature search strategy. An exhaustive PubMed search was performed, with final results retrieved on July 1, 2025. The search strategy combined Medical Subject Headings (MeSH) and free-text terms related to T2DM, ED, and CVD. Boolean operators were applied as appropriate to capture relevant associations among these conditions, and no initial filters were applied to maximize sensitivity.

Eligible studies were peer-reviewed articles published in English that reported on human populations and addressed ED, T2DM, CVD outcomes, or their interactions. Accepted study designs included randomized controlled trials, observational and cohort studies, case-control studies, population-based surveys, systematic reviews, and meta-analyses. Studies focusing on epidemiology, underlying mechanisms, cardiovascular risk, or clinical and therapeutic implications were included. Non-human studies, case reports, small case series, studies lacking explicit diagnostic criteria and publications without full text were excluded. However, a limited number of experimental and preclinical studies were considered when deemed essential to contextualize key pathophysiological mechanisms linking ED, T2DM, and CVD, without contributing to clinical risk estimation. Additional relevant publications were identified through manual screening of reference lists. All articles underwent a two-stage screening process (title and abstract review followed by full-text assessment). The review and selection were independently performed by two investigators with complementary expertise: a endocrinologist-andrologist (S.M.) and a researcher specialized in diabetes mellitus and metabolic diseases (B.V.). Any discrepancies were resolved by consensus, and only studies relevant to the review’s scope were included in the final synthesis. In total, 112 peer-reviewed articles were selected for full-text assessment, from those 98 articles fulfilled our narrative review criteria for study design and study focus. A comprehensive summary of the reviewed literature, including study design, population, and main findings, is provided in **Supplementary Table 1**. The PRISMA 2020 Checklist is available in **Supplementary Material 2**.

### 2.2 Use of real-world evidence from the TriNetX platform

To further address the objectives of this review, recent data from the TriNetX Global Collaborative Network were analyzed. This federated platform provides access to anonymized electronic health records from multiple healthcare organizations across diverse geographic regions.

A retrospective cohort was constructed, including men aged ≥20 years with a diagnosis of T2DM (ICD-10-CM: E11.x) recorded between December 9, 2024, and December 9, 2025 (over 12 months). ED was identified using ICD-10-CM (N52.x) codes and/or Anatomical, Therapeutic, Chemical (ATC) classification system (G04BE) codes for drugs used in erectile dysfunction, including organic, mixed, and unspecified forms of ED. Within these 12 months, both prevalent ED (existing diagnosis) and incident ED (newly recorded diagnosis) were captured. The same analysis was done for subjects without T2DM (excluding ICD-10-CM codes for any T2DM). Descriptive analyses estimated the prevalence rate and incidence rate of ED among men with T2DM and without T2DM during the study period.

Additionally, a separate comparative analysis using propensity score matching evaluated cardiovascular outcomes in men with ED, stratified by T2DM status. Cardiovascular outcomes were also compared between men with T2DM and ED and those with T2DM without ED. Cohorts were defined *a priori* using standardized ICD-10-CM diagnosis codes, ATC medication codes, and demographic criteria, as detailed in the **Supplementary Material** (**Appendices A–C**). Comparative analyses were performed using the built-in Compare Outcomes function, with index events defined as the first recorded diagnosis meeting cohort criteria, and outcomes assessed from 1 day after the index date onward. To minimize confounding, cohorts were balanced using propensity score matching, following a 1:1 nearest-neighbor matching approach. Propensity score matching was performed on 18 characteristics. In the Demographics category, patients were matched on Age at Index characteristic(s). In the Diagnosis category, patients were matched on Hypertensive diseases, Heart failure, Ischemic heart diseases, Cerebrovascular diseases, Diseases of arteries, arterioles and capillaries, Disorders of lipoprotein metabolism and other dyslipidaemias, chronic kidney disease (CKD), Nicotine dependence, unspecified, uncomplicated characteristic(s). In the Laboratory category patients were matched on Creatinine [Mass/volume] in Serum, Plasma or Blood, Cholesterol [Mass/volume] in Serum or Plasma, Cholesterol in LDL [Mass/volume] in Serum or Plasma, Cholesterol in HDL [Mass/volume] in Serum or Plasma, Triglyceride [Mass/volume] in Serum, Plasma or Blood, Hemoglobin A1c/Hemoglobin. Total in Blood, BMI, Blood Pressure, Systolic, Blood Pressure, Diastolic characteristic. Risk estimates, survival analyses (Kaplan–Meier with log-rank testing), and measures of association (risk ratios, odds ratios, and hazard ratios) were generated directly within the platform using default TriNetX statistical methods. All query definitions, cohort sizes, matching variables, time windows, and outcome definitions are fully reported in the **Supplementary Material** (**Appendices A–C**), enabling independent replication of the analyses by other researchers with access to the TriNetX platform.

## 3 Epidemiology of erectile dysfunction in men with type 2 diabetes mellitus: Incidence and prevalence

ED is a common condition in the general male population, with a prevalence that increases progressively with advancing age. Early epidemiological evidence, most notably from the Massachusetts Male Ageing Study (MMAS), reported an overall prevalence of ED of approximately 52% among men aged 40 to 70 years, highlighting its considerable burden in middle-aged and older individuals (15). Subsequent population-based studies have confirmed the high frequency of ED, although with notable variability across regions. For example, the Cologne Male Study reported a lower overall prevalence of 19.2% among men aged 30-80; however, age-stratified analyses revealed prevalence rates comparable to those of the MMAS in older age groups (16). Similarly, an epidemiological study conducted in Spain in a randomly selected population aged 25 to 70 years reported an ED prevalence of 18.9% (17). Variations in population characteristics, cultural variations, and the use of diverse diagnostic approaches, including validated questionnaires versus clinical interviews, likely explain differences across studies.

Compared with the general population, the prevalence of ED among men with T2DM is consistently higher. Reported prevalence estimates range widely, from 35% to 90%, reflecting heterogeneity in study design, diagnostic criteria, geographic setting, disease duration, and glycaemic control (18). Across multiple epidemiological cohorts, men with T2DM have approximately a three-fold increased risk of ED compared with non-diabetic men (19). Age remains a major determinant not only of ED occurrence but also of severity. In men with T2DM, the prevalence of mild, moderate, moderate-to-severe, and severe ED has been reported as 19.4%, 15.4%, 10.4%, and 21.6%, respectively, highlighting the progressive nature of erectile impairment with increasing age and disease burden (20).

Incidence and prevalence rates among males with T2DM were estimated using the TriNetX Global Collaborative Network over 12 months (December 9, 2024, to December 9, 2025). In a cohort of 4,013,416 men with T2DM, both the incidence and prevalence of ED increased markedly with age. During the 12-month observation window, the overall incidence proportion of ED was 6.5%, while prevalence reached 35.4%, indicating a substantial burden of ED in this population. Age-stratified analyses demonstrated a clear gradient in ED risk. Incidence was lowest among younger adults (3.3% in those aged 20–24 years) and increased progressively through middle age, peaking between 50 and 69 years, where incidence proportions ranged from 7.2% to 7.3%. Beyond age 70, incidence declined modestly, reaching 3.9% among individuals aged 80–84 years. The overall incidence rate among men aged 20 years and older was 2.89 × 10^−4^ cases per person-day, corresponding to approximately 105 cases per 1,000 person-years (**Table 2**).

**Table 2:** Incidence and prevalence rates of erectile dysfunction for different age groups using the real-world TriNetX database.

Age group (years)	Incidence cases		Incidence rates (%)		Prevalence cases		Prevalence rates (%)		Incidence rate (per 100,000 person-years)		Incidence rate (per 1,000 person-years)	
	With T2DM	Without diabetes	With T2DM	Without diabetes	With T2DM	Without diabetes	With T2DM	Without diabetes	With T2DM	Without diabetes	With T2DM	Without diabetes
20–24	152	2115	3.27	0.247	538	4082	11.06	0.476	15,520	534	15.5	5.34
25–29	396	3620	3.88	0.403	1417	7317	12.54	0.812	18,430	871	18.4	8.71
30–34	1169	6061	5.19	0.591	4148	13590	16.39	1.315	24,690	1,221	24.7	12.21
35–39	2299	9209	5.57	0.853	9655	21473	19.97	1.966	26,840	1,709	26.8	17.09
40–44	4042	12806	6.33	1.191	19570	32472	24.77	2.966	30,030	2,320	30	23.20
45–49	5836	15892	6.77	1.590	33359	42645	29.41	4.156	31,530	3,014	31.5	30.14
50–54	8479	21064	7.29	2.092	54391	59187	33.66	5.663	33,200	3,855	33.2	38.55
55–59	10457	25675	7.2	2.429	75940	74478	36.23	6.734	32,350	4,370	32.4	43.70
60–64	12812	31885	7.25	2.774	100396	93964	38.03	7.757	31,980	4,862	32	48.62
65–69	13951	33907	7.26	3.048	114363	102389	39.27	8.669	31,740	5,217	31.7	52.17
70–74	11849	26417	6.48	2.759	107462	83058	38.65	8.189	28,080	4,638	28.1	46.38
75–79	8929	16554	5.76	2.194	83754	51857	36.49	6.564	25,010	3,652	25	36.52
80–84	2798	8097	3.9	1.603	41117	24684	37.33	4.731	16,450	2,656	16.5	26.56
≥85	640	3875	3.47	0.925	11509	11374	39.15	2.666	14,590	1,589	14.6	15.89
Overall (≥20)	83809	217177	6.51	1.663	657619	622570	35.43	4.621	105,500	3,097	105.5	30.97

T2DM: type 2 diabetes mellitus.

Prevalence demonstrated a pronounced age-related increase. ED affected approximately 11% of men aged 20–24 years and rose steadily with age, reaching nearly 40% among those aged 55 years and older. The highest prevalence was observed in individuals aged 65–69 years and 85 years or older, approaching 39%. These findings indicate that, although incident ED becomes less frequent at very advanced ages, potentially due to survival effects or underdiagnosis, the cumulative burden of ED remains substantial throughout the lifespan of men with T2DM (**Table 2**).

Compared with subjects without T2DM (67,108,795 subjects), we consistently observed a lower incidence and prevalence of erectile dysfunction across all age groups compared with those with T2DM. In non-diabetic men, prevalence generally remained below 10%, whereas it reached around 35–40% in men with T2DM. Similarly, incidence proportions and incidence rates were markedly lower in those without T2DM throughout the entire age spectrum (**Table 2**).

## 4 Pathophysiological aspects of erectile dysfunction in T2DM

In T2DM, the physiological mechanisms controlling erection are compromised by the interaction of multiple pathological factors such as neurogenic failure, endothelial dysfunction and a decrease in nNO and eNO bioavailability (21). Among these, endothelial dysfunction is widely recognized as the primary cause of erectile impairment in men with T2DM and as a key mechanism linking ED with CVD (21). At the same time, oxidative stress and hypogonadism further contribute to the severity and complexity of erectile impairment.

Persistent hyperglycaemia induces oxidative stress, which decreases nitric oxide (NO) bioavailability and impairs endothelium-dependent vasodilation in both penile and systemic vasculature (9, 22). Increased levels of reactive oxygen species (ROS) directly inactivate NO and promote the formation of reactive nitrogen species, such as peroxynitrite, further disrupting NO signaling (23). Simultaneously, oxidative stress disrupts essential cofactors for NO synthesis, such as tetrahydrobiopterin, leading to endothelial NO synthase uncoupling and worsening endothelial dysfunction (23). These changes reduce cavernous smooth muscle relaxation and decrease penile blood flow, paralleling vascular abnormalities seen in coronary and peripheral arteries. Advanced glycation end-products (AGEs), formed during prolonged hyperglycaemia, significantly contribute to vascular damage. AGEs decrease NO availability, increase vascular stiffness, and promote smooth muscle dysfunction in the corpora cavernosa (6–8, 21). Additionally, upregulation of arginase II in T2DM reduces L-arginine availability for NO production, further impairing endothelial function (8, 9). Collectively, these vascular and oxidative mechanisms explain the close temporal and pathological association between ED and CVD in men with T2DM.

Chronic inflammation in T2DM, characterized by elevated circulating proinflammatory cytokines (24) and adhesion molecules (25), accelerates endothelial injury and atherosclerosis, reinforcing the shared vascular pathophysiological basis of ED and CVD. Key cytokines such as tumor necrosis factor-α (TNF-α), interleukin (IL)-6, IL-8, IL-1β, IL-18, and interferon-γ–inducible protein-10 (IP-10) are increased and have been associated with a higher risk of ED, whereas lower levels of the anti-inflammatory cytokine IL-10 appear to exert a protective effect. In parallel, elevated levels of soluble adhesion molecules, including soluble intercellular adhesion molecule-1 (sICAM-1), soluble vascular cell adhesion molecule-1 (sVCAM-1), and E-selectin, reflect endothelial activation and dysfunction, further contributing to vascular impairment.

Testosterone is essential for erectile physiology, affecting libido, penile smooth muscle structure, and NO synthase activity (26). Hypogonadotropic hypogonadism is common in men with T2DM, impacting approximately one-third of patients, and is often linked to obesity, insulin resistance, and chronic low-grade inflammation (27). Epidemiological studies consistently report a high prevalence of hypogonadism among men with metabolic syndrome, including T2DM among its criteria, 19%, 11% and 18% lower levels of total testosterone, calculated free testosterone and sex hormone-binding globulin than men without the syndrome (28). Conversely, low circulating testosterone levels are associated with an increased risk of developing T2DM, particularly in individuals with obesity and multiple metabolic syndrome components (29). A notable example of this bidirectional relationship is seen in men receiving androgen deprivation therapy for prostate cancer, who exhibit a significantly increased risk of metabolic dysfunction and T2DM (30). This complex association is primarily mediated by insulin resistance and dysfunctional adipose tissue, especially in obese individuals, which promotes hormonal imbalance and contributes to both ED and adverse cardiometabolic outcomes (31–37).

## 5 Association of erectile dysfunction with diabetic neuropathy, diabetic foot, diabetic retinopathy and diabetic kidney diseases

Diabetic neuropathy is a prevalent and clinically significant factor contributing to ED in T2DM. Typically, diabetic neuropathy develops due to microangiopathy resulting from chronic poor glycaemic control, which induces neurotoxicity through mechanisms such as increased AGEs, elevated oxidative stress, polyol pathway impairment, and enhanced activity of enzymes like protein kinase C polymerase (38, 39). Nerve damage reduces neuronal NO synthase production, leading to decreased neuronal NO release in the smooth muscle of the corpus cavernosum (40). Neuropathy impairs erectile function via two main pathways: a) peripheral sensory-motor neuropathy, where reduced sensory input diminishes sexual stimulation and impaired motor function affects pelvic floor muscles required for effective veno-occlusion (41); b) autonomic neuropathy, in which damage to autonomic fibers decreases parasympathetic activity and impairs neuronal NO release, compromising both initiation and maintenance of erection (42).

Epidemiological studies have shown a substantially higher prevalence of peripheral neuropathy among men with T2DM and ED compared with those without ED, with reported rates of 44% vs 7%, 57% vs 27%, and 72% vs 44% across the 20-34, 35-49, and 50–59 year age groups, respectively (43). Many of these studies were able to describe a strong association between diabetic neuropathy and ED in patients with T2DM (44–48), and some prospective cohorts report that the severity of neuropathy correlates with worse erectile function (49). For instance, a Japanese study, using the Sexual Health Inventory for Men (SHIM), a validated five-item version of the International Index of Erectile Function questionnaire, found that severe ED was independently associated with diabetic neuropathy (OR 1.90 (95% CI: 1.08–3.38). However, this relationship was not observed with retinopathy or nephropathy (50).

The association between ED and diabetic foot (DF) disease has been documented in several studies involving T2DM patients. DF represents a complex and severe complication that may result from the interplay of peripheral neuropathy, repetitive trauma, and peripheral arterial occlusive disease. This combination predisposes patients to recurrent infections, impaired wound healing, amputations, motor disability, and reduced quality of life (51). More than half of patients with DF have been reported to experience ED. The coexistence of both conditions has been associated with worse clinical outcomes. In particular, men with ED show poorer wound healing and higher rates of minor amputations and mortality compared with those without ED (wound healing: 73.3% vs 90.2%; minor amputations: 13.7% vs 4.8%; mortality: 25.7% vs 0.7%) (52). Moreover, in a cohort of 197 men with T2DM, ED severity assessed using the IIEF-5 questionnaire was significantly associated with a higher burden of both microvascular and macrovascular complications, including longer T2DM duration, proliferative retinopathy, peripheral neuropathy, increased urinary albumin excretion, and the presence of multiple cardiovascular risk factors (49).

A comparable association has been identified between ED and diabetic retinopathy (DR), a progressive microvascular complication of T2DM and a primary cause of vision loss. Research involving men with T2DM indicates that greater DR severity is associated with a significantly increased risk of ED, as demonstrated by a higher prevalence of significant ED (87.5% vs 50.0%; P < 0.0001) and lower mean Sexual Health Inventory for Men (SHIM) scores (9.5 ± 5.4 vs 14.7 ± 6.9; P < 0.0001) compared to controls (53). Notably, individuals with severe non-proliferative or proliferative DR have a three- to fourfold higher risk of ED than those with mild or no retinopathy, independent of age, T2DM duration, macrovascular comorbidities, and cardiovascular risk factors (54). These findings support the hypothesis that ED and DR share underlying mechanisms involving microvascular injury, endothelial dysfunction, and chronic hyperglycaemia-induced tissue damage.

Diabetic kidney disease (DKD), defined by albuminuria, reduced estimated glomerular filtration rate (eGFR), or both, is strongly associated with ED. Prevalence studies report that approximately 80-95% of men with DKD experience some degree of ED, establishing it as one of the most common comorbidities in this group (54). Chronic kidney disease (CKD), even without T2DM, disrupts the hypothalamic-pituitary-gonadal axis and may result in hyperprolactinemia due to decreased renal clearance (55). The combination of renal dysfunction with the metabolic and vascular complications of T2DM further increases both the prevalence and severity of ED (56). Meta-analyses of clinical and metabolic risk factors in men with T2DM confirm that ED is independently associated with DKD and other indicators of advanced systemic vascular damage, including CVD and microvascular complications. Reported odds ratios consistently range from approximately 1.9 to 2.7 across major cardiometabolic conditions (57, 58).

## 6 Association of erectile dysfunction with hypertension, dyslipidaemia, and obesity

The presence of additional cardiovascular risk factors substantially increases the burden of ED in men with T2DM. High blood pressure, dyslipidaemia, and obesity contribute independently to endothelial dysfunction and vascular impairment, amplifying the risk and severity of ED beyond the effect of hyperglycaemia alone (15). Long-term data from the Diabetes Prevention Program (DPP) Outcomes Study support this concept. After 15 years of follow-up, men with T2DM showed a significantly higher likelihood of developing ED; interestingly, that association appeared to be more closely related to features of the metabolic syndrome than to glycaemic control by itself, with an adjusted odds ratio of 1.85 (95% CI: 1.14-3.01), underscoring the predominant role of cardiometabolic factors in the development of ED among men with T2DM (59). These findings reinforce the notion that ED reflects the cumulative impact of cardiometabolic risk factors on vascular health. The association between hypertension and ED has been consistently demonstrated, both in terms of increased prevalence and greater severity of ED among hypertensive patients compared with normotensive individuals (60). Besides this, men receiving antihypertensive therapy have been reported to exhibit a higher risk of ED compared to untreated patients, pointing to the contribution of specific antihypertensive agents to erectile impairment. The effects of these medications are heterogeneous: diuretics and β-blockers are associated with less favorable erectile outcomes, whereas angiotensin receptor antagonists and nebivolol appear to have neutral or even beneficial effects on erectile function, likely due to their differential effects on penile haemodynamic, endothelial function, and nitric oxide bioavailability, with nebivolol uniquely enhancing endothelial nitric oxide synthase activity and cavernous vasodilation (60).

Dyslipidaemia, characterized by altered lipid levels, impairs erectile function primarily by inducing endothelial dysfunction and substantially reducing nitric oxide (NO) bioavailability (61). Oxidized low-density lipoprotein (LDL) has been identified as a key factor in compromising the relaxation response of the corpus cavernosum. Evidence demonstrates a direct association between lipid profiles and erectile dysfunction risk: each 1.0 mmol/l increase in total cholesterol raises the risk of erectile dysfunction by 1.32-fold, while higher concentrations of high-density lipoprotein (HDL) cholesterol significantly decrease the likelihood of developing the condition (61). Hypercholesterolemia contributes to atherosclerotic damage and the development of atherosclerotic plaques within the cavernous sinusoids, thereby restricting the blood flow required for erectile function. Lipid abnormalities frequently interact with other metabolic disorders; for instance, among patients with T2DM, the prevalence of erectile dysfunction reaches 72.73% when both hypercholesterolemia and hypertriglyceridemia are present (61, 62).

The association among obesity, body mass index (BMI), ED, and T2DM is explained by the “common background” hypothesis, which suggests that these conditions arise from a shared pathological environment defined by insulin resistance, chronic inflammation, and oxidative stress (63). Clinical studies indicate that obesity is an independent risk factor for ED, increasing the likelihood by approximately 1.3 times (64). A cross-sectional study further reveals that the prevalence of ED is notably higher in obese men (67.3%) compared to those with a normal BMI (51.1%) (64). Additionally, an elevated BMI is a strong predictor of increased ED severity; obese men (BMI ≥ 29 kg/m²) have a 1.78 to 1.97 times greater risk of ED, and the odds of developing moderate-to-severe ED are 2.51 to 2.71 times higher than in individuals of normal weight (64). Evidence from the Look AHEAD trial illustrates the potential impact of lifestyle modification. In a subgroup analysis of 372 overweight or obese men with T2DM followed for 1 year, participants assigned to an intensive lifestyle intervention achieved greater weight loss than those receiving standard diabetes education and support (9.9% vs 0.6%), accompanied by modest improvements in erectile function (65). These changes occurred alongside improvements in HbA1c, HDL cholesterol, and blood pressure. However, the observed benefits in erectile function were relatively modest and were interpreted as preservation rather than full recovery of erectile capacity, as only 22% of men reported improvements in erectile function over one year. This suggests that weight loss alone may not fully reverse established ED. Persistent factors such as visceral adiposity, testosterone deficiency, and suboptimal metabolic control may continue to impair endothelial function and contribute to chronic vascular dysfunction, even in the presence of lifestyle-induced improvements (65).

## 7 Association of erectile dysfunction with coronary artery disease, stroke, peripheral artery disease and heart failure

Despite advances in T2DM management, epidemiological data consistently show a markedly higher prevalence of CVD in people with T2DM compared with non-diabetic populations (66). Extensive contemporary studies, including the CAPTURE study, indicate that approximately one-third of adults with T2DM have established CVD, with coronary artery disease being the most frequent manifestation, with reported prevalence estimates of 34.8% (95% CI 32.7–36.8) for overall CVD and 31.8% (95% CI 29.7–33.8) for atherosclerotic CVD, and highlight a concerning trend toward earlier onset of macrovascular complications, particularly among men (67).

**Figure 1:**
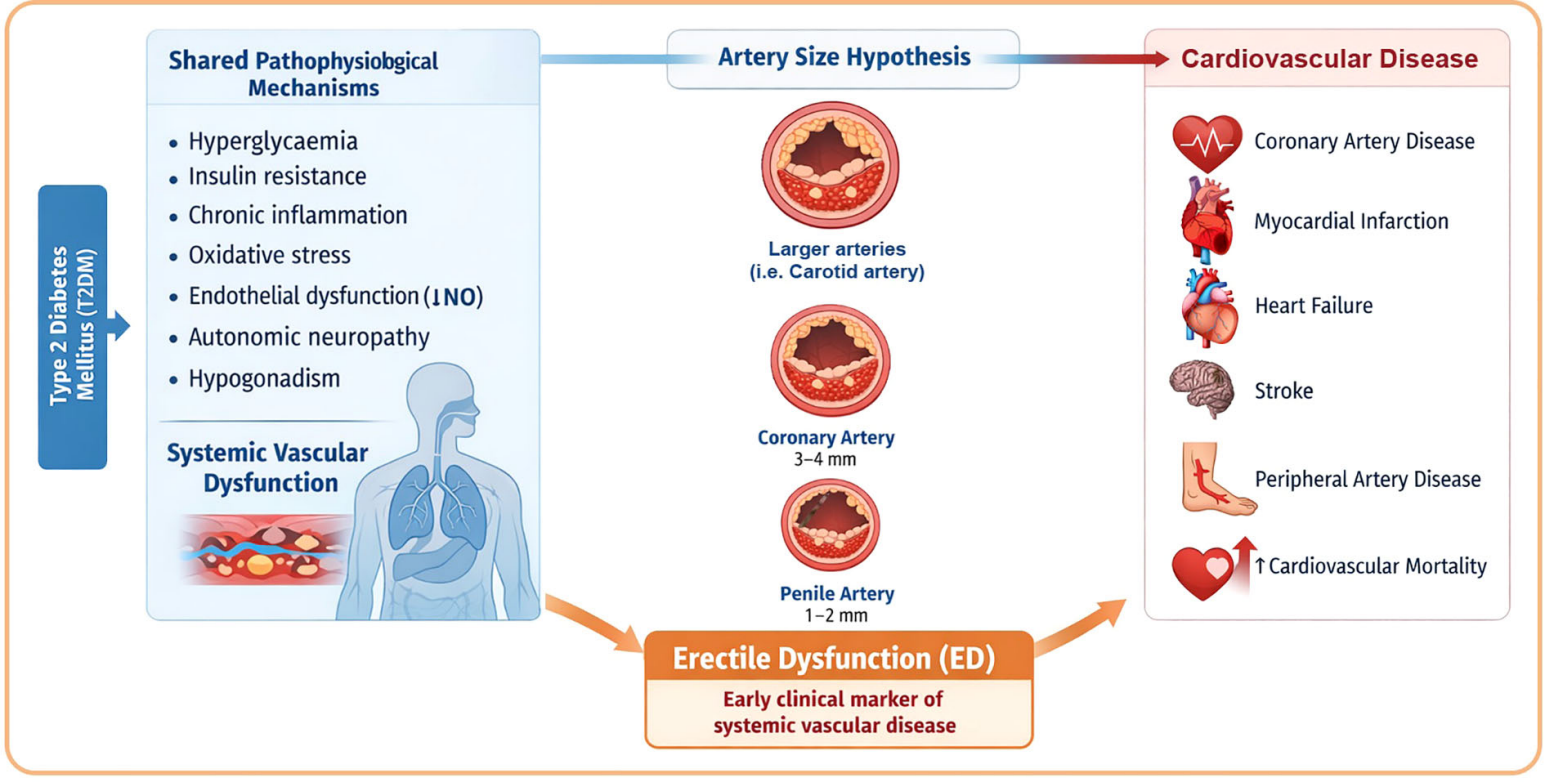
Shared pathophysiological mechanisms for erectile dysfunction, artery size hypothesis and cardiovascular disease Artery size hypothesis. Penile arteries (1–2 mm) are two to four times smaller than coronary or carotid arteries (3–4 mm). For an equivalent atherosclerotic plaque burden, smaller vessels develop proportionally greater luminal obstruction, leading to earlier impairment of blood flow. Created with Adobe Illustrator by María Belén Martínez Mores and Santiago Martínez Mores (S.M.).

The penile vasculature is uniquely sensitive to vascular injury due to the small diameter of penile arteries (1–2 mm), which is considerably smaller than coronary or carotid arteries (up to 4 times larger), indeed, according to the “artery size hypothesis,” (**Figure 1**) atherosclerotic plaque of comparable thickness will compromise penile blood flow much earlier than flow in larger vessels (68).

The association between ED and the degree of arterial involvement has been established, even in subclinical stages. Evidence from the COBRA trial shows that ED closely reflects the extent of arterial involvement, with its prevalence increasing from 22% in single-vessel disease to 55-65% in multivessel coronary disease, and that in patients with chronic coronary syndromes, ED preceded the first cardiovascular event in 93% of cases by a mean interval of approximately 2 to 3 years (69). In addition, ED appears to parallel the progression of CVD, becoming more pronounced as coronary involvement increases, with men with multivessel disease reporting substantially worse erectile function than those with single-vessel disease or normal coronary arteries (median SHIM scores 13–15 vs 22), and a SHIM score below 18 independently identifying patients with a higher likelihood of multivessel coronary disease (OR 0.84, 95% CI 0.73–0.97) (70).

A systematic review from 2019 reported significantly higher risks of cardiovascular events in men with ED, including increased incidence of general CVD (43%), coronary artery disease (59%), stroke (34%), and all-cause mortality (33%) (71). On the other hand, an observational study published in 2015 in a sample of 965 men without CVD showed that younger men, particularly those under 50 years of age, with transient or persistent ED have a higher cardiovascular risk according to the Framingham score (72). These findings support ED as a potential marker of vascular pathology. Consequently, clinical guidelines emphasize the importance of including sexual function assessment in cardiovascular risk stratification (73, 74). The Third Princeton Consensus Conference (2010) highlighted vasculogenic ED as an independent predictor of cardiovascular risk, recommending routine evaluation of erectile symptoms in cardiovascular prevention. The recommendation emphasized that sexual function should be incorporated into a comprehensive approach to CV risk factors, based on evidence that ED would allow the identification of subjects at risk for subclinical CVD (75). Similarly, in 2017, the QRISK score, a UK-developed cardiovascular risk prediction tool widely used to estimate 10-year cardiovascular risk in clinical practice, was updated to its third version (QRISK3), incorporating ED as an independent risk factor and reflecting growing evidence of its relevance in cardiovascular risk stratification (76).

ED is recognized as an early marker and predictor of stroke and other major adverse cardiovascular events (MACE), particularly in high-risk populations like men with T2DM (77). In patients with T2DM, the presence of ED is associated with an elevated risk of cerebrovascular disease (HR: 1.36, 95% CI: 1.11, 1.67) (78). Meta-analyses of prospective cohort studies have further quantified this relationship, finding that men with ED have an overall 1.35 times higher relative risk for stroke (95% CI: 1.19–1.54) compared to those without erectile issues (79). Genetic studies using Mendelian randomization support a causal link, suggesting that a genetic susceptibility to ischemic stroke is associated with a 34% higher risk for developing ED (80). Furthermore, among diabetic men, the prevalence of a composite endpoint including stroke and transient ischaemic attacks (TIA) is significantly higher in those with ED, often because the smaller penile arteries (1–2 mm) show symptoms of endothelial dysfunction and atherosclerosis years before the larger cerebral arteries (5–7 mm) (81).

The association between T2DM, peripheral artery disease (PAD), and ED is underpinned by a common pathophysiological basis (endothelial dysfunction, diminished NO bioavailability, and accelerated atherosclerosis). Among individuals with T2DM, PAD prevalence is estimated at 20-30%, while ED prevalence ranges from 35% to 75%, typically presenting 10 to 15 years earlier than in non-diabetic men (82). The artery-size hypothesis posits that smaller penile arteries are more likely to develop atherosclerotic obstruction before larger peripheral or coronary vessels, positioning ED as an early indicator of systemic vascular disease. Data from NHANES (2001–2004) indicate that PAD prevalence is over four times higher in men with ED (11.4%) compared to those without ED (2.6%). After adjustment for confounding variables, ED remains an independent predictor of PAD (OR: 2.05, 95% CI 1.24-3.39) (83). Additionally, PAD prevalence increases progressively with ED severity, rising from 28% in mild ED to 33% in moderate ED and 40% in severe ED. The most significant clinical risk is observed in men with both ED and T2DM, where PAD prevalence reaches 41% (84). Therefore, the presence of ED in a diabetic patient should prompt aggressive screening for PAD using the ankle-brachial index (ABI) and a thorough reassessment of cardiovascular risk. Further evidence links ED with subclinical atherosclerosis in men with T2DM. Studies evaluating carotid intima-media thickness (IMT) and peripheral arterial plaques have demonstrated that men with ED exhibit greater carotid IMT and a higher prevalence of lower-extremity atherosclerotic plaques compared to those without ED. These vascular markers also predict ED severity, supporting the concept that ED reflects early and generalized atherosclerotic disease in this population (85).

ED is a highly prevalent complication among individuals with heart failure (HF), with epidemiological studies reporting a prevalence ranging from 74% to 84%, and up to 90% in certain chronic subgroups (86). Multivariate analyses have identified T2DM as a major comorbidity that is significantly associated with both the onset and clinical severity of ED in this population (87). The principal pathophysiological link among HF, ED, and T2DM is similar to that in the rest of CVD, involving a deficit in the NO-cGMP signaling pathway, which is critical for cavernosal smooth muscle relaxation and the maintenance of normal penile hemodynamic. The degree of erectile impairment also correlates with the clinical progression of HF; approximately 70% of patients in New York Heart Association (NYHA) Class I demonstrate normal or only mildly impaired erectile function (87). Iatrogenic factors further contribute to ED, as key HF pharmacotherapies, including digoxin, thiazide diuretics, and spironolactone, have been shown to exacerbate ED through mechanisms such as sodium-pump inhibition and antiandrogenic effects (87).

## 8 Erectile dysfunction unmasks cardiovascular risk in type 2 diabetes mellitus

Early studies demonstrated that men with T2DM and ED experience significantly more cardiovascular events during long-term follow-up than those without ED, establishing ED as an independent predictor of cardiovascular morbidity and mortality in this population (HR: 2.1; 95% CI 1.6-2.6) (77). Subsequent cohort studies confirmed these findings, showing that men with T2DM and ED at baseline have a higher incidence of CVD and a more adverse cardiometabolic profile. Notably, ED remains an independent predictor of coronary heart disease after adjustment for age, T2DM duration, antihypertensive treatment, and albuminuria (HR 1.58; 95% CI 1.08-2.30) (88).

A recent meta-analysis quantified this association, reporting a 68% higher risk of CVD among men with both T2DM and ED compared to diabetic men without ED (89). Importantly, the same analysis did not identify increased cardiovascular risk when comparing men with ED from the general population to those with T2DM alone, suggesting that diabetes mellitus amplifies the cardiovascular impact of ED. Therefore, ED has greater prognostic significance in the context of T2DM, where vascular disease progression is accelerated.

The relationship between ED and cardiovascular risk is bidirectional. Improvements in cardiometabolic risk factors, such as glycaemic control, lipid levels, and blood pressure, have been associated with better erectile function, as indicated by higher IIEF-5 scores (90). This evidence suggests that interventions targeting cardiovascular risk may reduce future cardiovascular events while simultaneously improving sexual function. While observational and interventional studies have consistently supported this interaction, large real-world datasets now allow for a more detailed assessment of how ED and T2DM jointly influence cardiovascular risk.

To further examine this interaction, we present a recent propensity-score-matched comparative analysis using real-world data from the TriNetX Global Collaborative Network to evaluate the cardiovascular risk profile of men with ED by T2DM status. After matching for demographic characteristics, comorbidities, and laboratory variables, both cohorts included over 200,000 individuals for each cardiovascular outcome. As shown in **Table 3**, the presence of T2DM significantly increased the risk of all major cardiovascular events among men with ED. The risk of ischemic heart disease was 15.7% in individuals with both ED and T2DM, compared to 11.5% in those with ED but without T2DM (OR: 1.44; 95% CI 1.41-1.46). Similar patterns were observed for stroke (OR 1.45; 95% CI 1.42-1.48) and peripheral artery disease (OR 1.38; 95% CI 1.35-1.41), while the risk of heart failure was nearly doubled in the presence of T2DM (8.4% vs. 4.9%; OR 1.78; 95% CI 1.74-1.81). For the composite CVD outcome, men with ED and T2DM had a substantially higher risk than those without T2DM (24.2% vs. 18.3%; OR 1.43; 95% CI 1.41-1.45), collectively demonstrating that T2DM markedly increases cardiovascular vulnerability in men with ED.

**Table 3:** Risk of cardiovascular events associated with erectile dysfunction in people with and without type 2 diabetes from the TriNetX cohort.

Outcome	Cohort	Patients in the cohort	Patients with outcome	Risk	Odds ratios	95% CI
Ischemic heart disease	ED with T2DM	238,185	37,506	0.157	1.435	(1.411, 1.459)
	ED without T2DM	241,115	27,784	0.115		
Stroke	ED with T2DM	285,311	22,450	0.079	1.450	(1.420, 1.481)
	ED without T2DM	285,837	15,895	0.056		
Heart failure	ED with T2DM	285,408	23,842	0.084	1.775	(1.737, 1.813)
	ED without T2DM	287,971	14,069	0.049		
Peripheral artery disease	ED with T2DM	280,898	23,016	0.082	1.377	(1.349, 1.405)
	ED without T2DM	286,968	17,473	0.061		
CVD	ED with T2DM	201,440	48,769	0.242	1.426	(1.405, 1.448)
	ED without T2DM	207,930	38,055	0.183		

CVD: cardiovascular disease, CVD includes: ischemic heart disease, stroke, heart failure or peripheral artery disease; T2DM: type 2 diabetes mellitus; ED: erectile dysfunction; CI: confidence intervals.

A complementary propensity-score-matched analysis evaluated whether ED confers additional cardiovascular risk among individuals with established T2DM (**Table 4**). In this cohort, ED was consistently associated with modest but clinically significant increases in risk. Men with T2DM and ED had higher rates of ischemic heart disease (16.9% vs. 15.6%; OR 1.10; 95% CI 1.09-1.12) and stroke (9.1% vs. 8.4%; OR 1.09; 95% CI 1.07-1.10) compared to diabetic men without ED. Peripheral artery disease was also more prevalent in the presence of ED (8.7% vs. 7.6%; OR 1.17; 95% CI 1.15-1.18), while heart failure risk differed minimally between groups (OR 1.01; 95% CI 1.00-1.03). For the composite CVD outcome, ED remained associated with increased risk in individuals with T2DM (25.8% vs. 23.6%; OR 1.12; 95% CI 1.11-1.14).

**Table 4:** Risk of different cardiovascular events in people with type 2 diabetes with and without erectile dysfunction from the TriNetX cohort.

Outcome	Cohort	Patients in the cohort	Patients with outcome	Risk	Odds ratios	95% CI
Ischemic heart disease	T2DM with ED	385,971	65,253	0.169	1.103	(1.090, 1.116)
	T2DM without ED	382,050	59,504	0.156		
Stroke	T2DM with ED	493,588	44,683	0.091	1.087	(1.072, 1.102)
	T2DM without ED	490,722	41,161	0.084		
Heart failure	T2DM with ED	484,863	49,149	0.101	1.013	(0.999, 1.026)
	T2DM without ED	482,451	48,350	0.100		
Peripheral artery disease	T2DM with ED	497,508	43,283	0.087	1.166	(1.149, 1.182)
	T2DM without ED	507,938	38,386	0.076		
CVD	T2DM with ED	321,207	82,829	0.258	1.122	(1.109, 1.135)
	T2DM without ED	311,504	73,660	0.236		

CVD: cardiovascular disease, CVD include: Ischemic heart disease, stroke, heart failure or Peripheral artery disease; T2DM: type 2 diabetes mellitus; ED: erectile dysfunction; CI: confidence intervals.

Taken together, these findings indicate that ED functions as a clinical marker reflecting shared vascular and metabolic disturbances associated with increased cardiovascular risk in men with T2DM. This underscores the need for systematic cardiovascular screening and intensive risk factor management in this population.

## 9 Erectile dysfunction and cardiovascular- renal protective drugs

Cardiovascular and renal protective medications are essential in managing chronic conditions (heart failure, CKD, etc.) in the presence of T2DM, but their impact on ED is different. Management of T2DM involves a broad spectrum of pharmacological therapies with effects extending beyond glycaemic control, potentially influencing both CVD and ED.

### 9.1 Sodium–glucose cotransporter-2 inhibitors

Sodium–glucose cotransporter-2 inhibitors (SGLT2) inhibitors (gliflozins) provide cardiorenal protection by blocking glucose and sodium reabsorption in the proximal tubule, restoring tubule-glomerular feedback (91). Trials such as CREDENCE and DAPA-CKD showed reduced risk of end-stage kidney disease, slower eGFR decline, and 30–50% reductions in albuminuria (92). Cardiovascular benefits include fewer cardiovascular deaths and fewer heart failure hospitalizations across both HFrEF and HFpEF (e.g., DAPA-HF, EMPEROR-Preserved). Additional mechanisms include metabolic efficiency via ketone utilization and anti-inflammatory/antifibrotic effects (92). These benefits are attributed to favorable haemodynamic effects, reduced oxidative stress, and renal protection. The association between SGLT2i and ED shows a gap between promising mechanisms and mixed clinical outcomes. Mechanistically, SGLT2i may enhance erectile function by reducing oxidative stress and inflammation, improving endothelial function, and facilitating vasodilation (94). Experimental studies with empagliflozin have demonstrated improved nitric oxide-mediated relaxation of the corpus cavernosum (93). In contrast, clinical data remain inconsistent. A large retrospective cohort study of 322,089 SGLT2i users reported a higher risk of ED than non-users (risk ratio 1.507), and a higher frequency of phosphodiesterase inhibitor use among SGLT2i users (8.2% versus 5.2%) (94). Due to these conflicting findings and the scarcity of prospective data, additional well-designed clinical trials are required to clarify the impact of SGLT2i on ED in people with T2DM.

### 9.2 Glucagon-lik1 peptide-1 receptor agonists

Glucagon-like peptide-1 receptor agonists (GLP-1RAs) primarily protect the heart and kidneys by acting as incretin mimetics that enhance metabolic health and reduce systemic inflammation. Regarding cardiovascular outcomes, GLP-1RAs consistently reduce the risk of MACE, including cardiovascular death, nonfatal myocardial infarction, and nonfatal stroke (91). Clinical trials such as LEADER and SUSTAIN-6 have confirmed these benefits in high-risk patients with T2DM. These properties are particularly relevant in patients with T2DM at high cardiovascular risk. The relationship between GLP-1RAs and erectile function reflects the balance between potential vascular benefits and emerging safety concerns (95). Supportive evidence comprises exploratory analyses from the REWIND trial demonstrating reduced incidence and progression of moderate-to-severe ED with dulaglutide (96). Mendelian randomization studies indicate a protective causal effect, partly mediated by weight loss and cardiometabolic improvements, and meta-analyses show greater improvement in IIEF-5 scores compared with metformin, particularly among individuals with obesity (97, 98). Nevertheless, retrospective cohort studies have reported higher rates of newly diagnosed ED and testosterone deficiency among non-diabetic men with obesity treated with semaglutide for weight loss, and pharmacovigilance databases have recorded reports of ED and reduced libido associated with GLP-1RAs. However, these signals remain weak and potentially confounded (99). GLP-1RAs show promise for addressing vascular contributors to ED. Current evidence remains inconclusive, and dedicated randomized controlled trials are needed to confirm the effect of these drugs on ED.

### 9.3 Metformin

Metformin remains the first-line pharmacological therapy for T2DM worldwide. In addition to its glucose-lowering effects through enhanced insulin sensitivity and reduced hepatic gluconeogenesis, metformin has been associated with cardiovascular benefits, likely mediated by reductions in oxidative stress, improvements in endothelial function, and anti-inflammatory actions. These mechanisms could theoretically translate into indirect benefits for erectile function; however, direct clinical evidence supporting a specific effect of metformin on ED remains limited (100).

### 9.4 Nonsteroidal mineralocorticoid receptor antagonists

Finerenone is a highly selective, nonsteroidal mineralocorticoid receptor antagonist developed for patients with T2DM and CKD. Its nonsteroidal structure enables balanced distribution to cardiac and renal tissues and confers anti-inflammatory and antifibrotic effects by inhibiting mineralocorticoid receptor overactivation. The FIDELIO-DKD and FIGARO-DKD trials demonstrated significant reductions in kidney disease progression and major cardiovascular events, particularly hospitalization for heart failure, with a favorable safety profile (101). A key distinction from older steroidal MRAs is its impact on sexual function. Spironolactone and similar agents interact with androgen and progesterone receptors, contributing to erectile dysfunction, gynecomastia, and reduced libido. Finerenone shows minimal affinity for sex-hormone receptors and therefore substantially reduces the risk of treatment-related sexual dysfunction while preserving strong cardiorenal benefits (101).

### 9.5 Angiotensin-converting enzyme inhibitors and angiotensin II receptor blockers

Angiotensin-converting enzyme inhibitors (ACEIs) and angiotensin II receptor blockers (ARBs) have long served as foundational therapies for cardiorenal protection. In renal physiology, ACEIs and ARBs produce a specific hemodynamic effect by dilating the efferent arteriole, thereby reducing intraglomerular pressure. This action is essential for preventing glomerular hyperfiltration and reducing albuminuria, thereby slowing the progression of CKD. These drug classes are also established treatments for HF, improving survival and reducing hospitalizations by counteracting the harmful effects of renin-angiotensin system overactivation. Among antihypertensive agents, ACEIs and ARBs are preferred for men concerned about ED, as they do not exhibit the adverse sexual effects frequently associated with thiazides and non-selective β-blockers (102). ACEIs lower angiotensin II levels and increase bradykinin availability, promoting nitric oxide-mediated vasodilation (103). Most clinical studies report a neutral impact on erectile function, with no significant changes in International Index of Erectile Function (IIEF) scores compared to baseline or to other neutral antihypertensives (102). In contrast, ARBs block AT₁ receptors, thereby preventing angiotensin II–induced vasoconstriction of cavernosal smooth muscle. Evidence regarding ARBs is more consistently positive: randomized and observational studies demonstrate significant increases in sexual activity frequency and improvements in IIEF scores, particularly among hypertensive men with pre-existing ED (104). Meta-analyses reveal modest but statistically significant improvements compared to placebo or other drug classes, although study heterogeneity persists.

### 9.6 Pharmacological treatment of ED and its impact on cardiovascular disease

Phosphodiesterase type 5 inhibitors (PDE5i), including sildenafil, tadalafil, and vardenafil, were initially developed in the 1990s as antianginal agents due to their effects on cyclic guanosine monophosphate (cGMP) metabolism and are currently the most widely used pharmacological treatment for ED. Although they were not designed to improve cardiovascular outcomes, growing evidence suggests that PDE5i may exert favorable vascular effects.

Mechanistically, PDE5i enhance nitric oxide cGMP signaling, promote vasodilation, and reduce systemic inflammation, thereby improving endothelial function across multiple vascular territories (105, 106). These effects have been associated with reduced arterial stiffness and improved vascular compliance, both of which are relevant to cardiovascular health (107).

Observational studies have reported an association between PDE5i use and lower cardiovascular mortality, as well as a reduced incidence of major adverse cardiovascular events in men with T2DM (77, 108). However, despite involving large cohorts, these findings remain observational, and randomized controlled trials are required to establish causality and quantify the magnitude of any cardioprotective effects (109, 110).

From a clinical perspective, it is essential to note that a subset of men with T2DM exhibit a suboptimal response to PDE5 inhibitors. This reduced efficacy may be related to multiple factors, including testosterone deficiency, advanced macrovascular disease, impaired neurogenic and endothelium-dependent vasodilation, and the concomitant use of medications for T2DM and associated comorbidities. As a result, higher or maximal recommended doses of PDE5 inhibitors are often required in this population to achieve satisfactory erectile responses (18).

Finally, preclinical studies have explored the cardiovascular effects of PDE5 inhibition in experimental models of diabetic cardiomyopathy. In animal studies, chronic administration of tadalafil and vardenafil has been shown to modulate redox signaling pathways, attenuate myocardial hypertrophy, and reduce fibrotic remodeling, suggesting potential cardioprotective mechanisms in diabetic conditions (111, 112). While these findings are intriguing, their clinical relevance remains to be established.

## 10 Clinical implications and future directions

ED in men with T2DM should be recognized as both a quality-of-life concern and a clinically significant indicator of early and systemic vascular disease. The strong association between ED and conditions such as endothelial dysfunction, subclinical atherosclerosis, neuropathy, and hypogonadism underscore the importance of systematically integrating sexual health assessment into routine T2DM care. Early detection of ED provides an opportunity to identify occult cardiovascular disease and to intensify comprehensive risk factor management, including blood pressure, lipid levels, smoking cessation, and glycaemic control.

In clinical practice, structured assessment of ED using validated questionnaires can improve case detection, particularly among younger or recently diagnosed men in whom ED may precede overt cardiovascular events. Multidisciplinary management that includes primary care, endocrinology, cardiology, and andrology is likely to achieve better outcomes than isolated specialty-based approaches. Pharmacologically, phosphodiesterase type-5 inhibitors remain the first-line treatment; however, optimal management of underlying metabolic abnormalities, such as weight reduction, improved insulin sensitivity, and correction of testosterone deficiency when present, is essential for sustained therapeutic benefit.

Future research should prioritize longitudinal studies that evaluate ED as a prognostic biomarker for cardiovascular and renal outcomes in T2DM, ideally incorporating imaging surrogates of vascular disease and mechanistic biomarkers. Randomized trials are also needed to determine whether erectile dysfunction-guided cardiovascular screening or intensified cardiometabolic treatment strategies can improve clinical outcomes. Additionally, real-world evidence platforms, such as federated electronic health record networks, provide valuable opportunities to capture large-scale data across diverse populations and to examine treatment patterns, disparities, and long-term outcomes.

## 11 Limitations

This narrative review is subject to inherent limitations. Although a structured literature search was performed, the absence of a formal systematic methodology may introduce selection bias and limit reproducibility. Furthermore, restricting the search to English-language peer-reviewed literature may have introduced publication and language bias, and relevant studies published in other languages or negative results may have been underrepresented. Finally, heterogeneity across studies in the definition and assessment of ED and CVD, along with the predominance of observational designs, restricts causal inference and may affect the comparability of findings.

The real-world analysis using the TriNetX platform also has limitations related to reliance on administrative coding, which may underestimate ED prevalence due to underdiagnosis in routine clinical practice. The lack of validated measures of ED severity and limited information on disease duration, metabolic control, and lifestyle factors further constrain interpretation. Despite these limitations, combining traditional evidence with large-scale real-world data provides a complementary perspective on the relationship between ED, T2DM, and CVD.

## 12 Conclusions

Erectile dysfunction is highly prevalent among men with T2DM and reflects the systemic nature of diabetic complications. Shared mechanisms such as endothelial dysfunction, chronic inflammation, oxidative stress, neuropathy, and hypogonadism contribute to its close association with cardiovascular disease. Erectile dysfunction often precedes overt cardiovascular disease and therefore serves as a clinically accessible marker of increased cardiometabolic risk.

Incorporating erectile dysfunction screening into routine T2DM management may enable earlier detection of subclinical vascular disease and support comprehensive risk factor intervention. Evidence from epidemiological studies and real-world data consistently demonstrates the substantial burden and prognostic significance of erectile dysfunction in T2DM. Future prospective studies are required to clarify causal relationships, refine risk stratification strategies, and determine whether targeted interventions based on erectile dysfunction status can improve cardiovascular and patient-reported outcomes. Collectively, these efforts have the potential to establish erectile dysfunction as a key component of holistic T2DM care rather than an under-recognized complication.
