# Supplementary material for: Erectile dysfunction, type 2 diabetes, and cardiovascular disease: a narrative review and insights from a global real-world cohort analysis
Source: Front Clin Diabetes Healthc. 2026 Mar 6;7:1781581. doi: 10.3389/fcdhc.2026.1781581 (PMC13003461; doi:10.3389/fcdhc.2026.1781581)
Supplement: Supplementary file 1 [file DataSheet1.docx]

Erectile dysfunction, type 2 diabetes, and cardiovascular disease: a narrative review and insights from a global real-world cohort analysis

Santiago Martínez Mores, Josep Franch, Didac Mauricio, Bogdan Vlacho

| Title | Page |
| --- | --- |
| Supplementary Table S1. Summary table of the included articles | 3 |
| Appendix A: Analysis Report: Prevalence, incidence, risk factors and complications related to erectile dysfunction among people with type 2 diabetes mellitus Incidence and Prevalence | 30 |
| Appendix B: Propensity-score–matched comparative analysis for estimating the risk for different cardiovascular events among people with erectile dysfunction in the presence of type 2 diabetes and the absence of diabetes | 34 |
| Appendix C: Propensity-score–matched comparative analysis for estimating the risk for different cardiovascular events among people with type 2 diabetes regarding the presence or absence of erectile dysfunction | 64 |

Supplementary Table S1. Summary table of the included articles

| **Number of the reference in the manuscript** | **Study identification** | **Study design** | **Participants** | **Interventions** | **Outcomes** | **Results** | **Quality/Risk of Bias** |
| --- | --- | --- | --- | --- | --- | --- | --- |
| 8 | Jiaan, D B et al. (1995) | Experimental, cross sectional tissue study | Human penile tissue specimens from autopsy and living subjects (cadaveric and surgical samples) | Quantification of pentosidine (advanced glycation end product) in corpus cavernosum and tunica albuginea | AGE accumulation in penile tissue; mechanisms related to nitric oxide bioavailability | Pentosidine levels increased exponentially with age in penile tissues (≈4–6-fold from puberty to old age), indicating AGE-related collagen modification and impaired tissue turnover, providing a mechanistic link between aging, diabetes, and erectile dysfunction | Moderate |
| 11 | Seftel et al., 1997 | Experimental, cross sectional human tissue study | Diabetic and nondiabetic men; human penile corpus cavernosum and tunica albuginea specimens | Quantification of AGEs (pentosidine, pyrraline) and assessment of NOS isoforms (iNOS, eNOS) in penile tissue; pharmacological iNOS inhibition | AGE accumulation, NOS expression, cavernosal smooth muscle relaxation | Diabetic penile tissue showed significantly higher AGE (pentosidine) levels localized to tunical and cavernosal collagen, with altered NOS expression (iNOS upregulation and reduced eNOS); selective iNOS inhibition significantly improved cavernosal relaxation, supporting an AGE-mediated mechanism of erectile dysfunction | Moderate |
| 14 | Hostnik et al., 2025 | Narrative review | Men with diabetes mellitus and erectile dysfunction (literature-based) | Review of pathophysiological, genetic, and therapeutic studies | ED prevalence, mechanisms, genetic associations, and treatment response | ED prevalence was >3.5-fold higher in men with diabetes; diabetic ED was shown to be multifactorial, involving endothelial dysfunction, neuropathy, atherosclerosis, hormonal and structural changes; genetic polymorphisms were associated with ED risk and treatment response | Moderate |
| 15 | Feldman et al., 1994 | Observational cohort study | Non-institutionalized men aged 40–70 years from the general population | Assessment of erectile function using a self-administered questionnaire; evaluation of medical, hormonal, and psychosocial factors | Prevalence of erectile dysfunction and associated medical and psychosocial correlates | Overall prevalence of erectile dysfunction was 52%, with complete ED increasing threefold from ages 40 to 70; ED was independently associated with diabetes, heart disease, hypertension, related medications, smoking, and depressive symptoms, supporting a strong link between ED and vascular risk factors | Low |
| 16 | Braun et al., 2000 | Cross sectional survey | Representative sample of men aged 30–80 years from the Cologne urban district, Germany (n = 4,489 respondents) | Assessment of erectile function and sexual satisfaction using a validated self-administered questionnaire | Prevalence of erectile dysfunction and associated comorbidities | ED prevalence was 19.2%, with a marked age-related increase (2.3–53.4%); ED was frequently associated with hypertension, diabetes, pelvic surgery, and lower urinary tract symptoms, and was linked to significant dissatisfaction with sexual life | Moderate |
| 17 | Martin-Morales et al., 2001 | Cross sectional survey | Non-institutionalized Spanish men aged 25–70 years (n = 2,476) | Assessment of erectile function using self-report and the International Index of Erectile Function; collection of medical, lifestyle, and medication data | Prevalence of erectile dysfunction and independent risk factors | ED prevalence ranged from 12.1% (self assessment) to 18.9% (IIEF); diabetes showed the strongest independent association with ED (age-adjusted OR= 4), followed by peripheral vascular disease (OR 2.63), hypertension (OR 1.58), hypercholesterolemia (OR 1.63), cardiac disease (OR 1.79), smoking (OR 2.5), and alcohol use (OR 1.53) | Moderate |
| 19 | Saigal et al., 2006 | Cross sectional study (NHANES 2001–2002) | Non-institutionalized US men aged ≥20 years (n = 3,566) | Assessment of erectile dysfunction through standardized household interviews and medical history questionnaires | Prevalence of erectile dysfunction and independent risk factors | ED affected nearly 20% of adult men and 77.5% of those aged ≥75 years; diabetes was independently associated with ED (OR 2.69), obesity (OR 1.60), smoking (OR 1.74), and hypertension (OR 1.56); Hispanic ethnicity was associated with higher ED risk (OR 1.89) | Moderate |
| 20 | Corona et al., 2014 | Multicenter prospective observational study: SUBITO-DE | Men with newly or recently diagnosed type 2 diabetes mellitus from 27 Italian diabetes centers (n = 499) | Clinical assessment of sexual function, hormonal profile, depressive symptoms, and cardiometabolic parameters | Prevalence and determinants of erectile dysfunction and other sexual dysfunctions at T2DM onset | ED was highly prevalent at T2DM diagnosis, with moderate-to-severe ED in 32% of patients; hypogonadism was present in 20%, and depressive symptoms and diabetes-related complications independently increased ED risk, highlighting early involvement of hormonal, psychological, and cardiovascular factors | Moderate |
| 22 | Guay, 2007 | Narrative review | Men with erectile dysfunction and cardiovascular risk factors | Conceptual analysis of endothelial function linking erectile dysfunction and cardiovascular disease | Endothelial dysfunction as a shared mechanism between ED and CVD | The review proposed erectile dysfunction as a clinical manifestation of systemic endothelial dysfunction, highlighting that penile arteries, due to their smaller diameter, may become symptomatic earlier than coronary arteries, positioning ED as a potential early marker of cardiovascular disease and an opportunity for early cardiovascular risk assessment | Moderate |
| 24 | Liu et al., 2024 | Mendelian randomisation study | Genome-wide association study (GWAS) data on inflammatory cytokines (n = 8,293) and erectile dysfunction cases and controls from FINNGEN R9 (2,205 ED cases; 164,104 controls) | Genetic instrumental variable analysis of 41 inflammatory cytokines using MR-Egger, IVW, and weighted median methods | Genetic causal associations between inflammatory cytokines and erectile dysfunction | Genetically predicted higher levels of IP-10 were associated with increased ED risk (OR 1.27, 95% CI 1.01-1.60), whereas higher IL-1RA levels were protective (OR 0.77, 95% CI 0.60-0.98); reverse MR analyses did not support causality from ED to cytokine levels | Moderate |
| 25 | Hegazy et al., 2020 | Cross sectional clinical study | Controls (n=21), T2DM without CVD (n=30), and T2DM with macrovascular CVD (n=30) | Measurement of circulating soluble adhesion molecules (sVCAM-1, sICAM-1, sE-selectin) by ELISA | Endothelial activation markers and macrovascular complications in T2DM | Levels of sVCAM-1, sICAM-1, and sE-selectin were higher in T2DM patients than controls; sVCAM-1 was significantly higher in T2DM patients with CVD than those without CVD and showed positive correlations with other adhesion molecules, identifying sVCAM-1 as a marker of macrovascular disease in T2DM | Moderate |
| 26 | Isidori et al., 2014 | Systematic review | Men with erectile dysfunction with or without hypogonadism (literature-based) | Systematic review of molecular studies, observational cohorts, and randomized controlled trials assessing testosterone and erectile function | Role of testosterone in erectile physiology and response to treatment | Testosterone was shown to modulate multiple components of erectile function, including endothelial cells, smooth muscle, neural pathways, and sexual desire; hypogonadism frequently coexisted with ED, and testosterone replacement therapy improved erectile function in appropriately selected hypogonadal men, particularly when used to restore physiological androgen levels rather than as routine combination therapy | Moderate |
| 27 | Dhindsa et al., 2004 | Cross sectional clinical study | Men with type 2 diabetes mellitus (n = 103; mean age 54.7 years) | Measurement of total testosterone, free testosterone, SHBG, LH, FSH, and prolactin | Prevalence and type of hypogonadism in T2DM | Hypogonadism was present in 33% of men with T2DM and was predominantly hypogonadotropic, with significantly lower LH and FSH levels; free testosterone was inversely correlated with BMI, supporting a central, obesity-related mechanism | Moderate |
| 28 | Laaksonen et al., 2003 | Cross sectional study | Non-diabetic middle-aged Finnish men from a community cohort (n = 1,896) | Measurement of total testosterone, calculated free testosterone, SHBG, and inflammatory markers | Association between sex hormones, metabolic syndrome, and inflammatory markers | Men with metabolic syndrome had significantly lower free testosterone (-11%) and SHBG (-18%); men in the lowest tertile of free testosterone had a higher likelihood of metabolic syndrome (OR 2.7, 95% CI 2.0-3.7; OR 1.7 after BMI adjustment), with inverse associations between sex hormones and insulin resistance, dyslipidemia, and inflammation | Moderate |
| 29 | Haffner et al., 1996 | Prospective case control study | Initially nondiabetic men from the Multiple Risk Factor Intervention Trial (MRFIT) cohort (176 incident diabetes cases and matched controls) | Baseline measurement of total and free testosterone, SHBG, insulin, and glucose | Baseline measurement of total and free testosterone, SHBG, insulin, and glucose | Lower baseline levels of testosterone and SHBG were associated with a higher risk of developing diabetes over 5 years of follow up; these associations were attenuated after tight matching for BMI and fasting glucose, suggesting that low testosterone and SHBG are features of the prediabetic metabolic state rather than independent causal factors | Moderate |
| 30 | Saad, 2009 | Narrative review | Men with type 2 diabetes mellitus, metabolic syndrome, or hypogonadism | Review of epidemiological and interventional studies evaluating testosterone and metabolic outcomes | Role of testosterone in glucose homeostasis, metabolic syndrome, and diabetes risk | Low testosterone and SHBG levels were consistently associated with insulin resistance, metabolic syndrome, and increased risk of type 2 diabetes; testosterone deficiency was proposed as a component of the metabolic syndrome, and testosterone replacement in hypogonadal men was associated with partial improvement of cardiometabolic risk factors, although long-term outcome data were limited | Moderate |
| 31 | Vikan et al., 2010 | Prospective cohort study | Community dwelling men from the Tromsø Study (n = 1,454) | Baseline measurement of total testosterone, SHBG, and estradiol with longitudinal follow-up for incident diabetes | Incident type 2 diabetes mellitus | Higher total testosterone and SHBG levels were associated with a lower risk of incident diabetes (e.g., testosterone HR 0.71 per SD; SHBG HR 0.55 per SD), although these associations were attenuated after adjustment for waist circumference; higher estradiol levels independently predicted a higher risk of diabetes, even after accounting for obesity | Moderate |
| 32 | Simon et al., 1997 | Case control study within a population cohort (Telecom Study) | Healthy adult men matched by age and ethnicity, stratified by total testosterone levels (n = 50) | Comparison of cardiometabolic risk factors according to plasma total testosterone and SHBG levels | Cardiovascular and metabolic risk factors associated with low testosterone | Men with low total testosterone had significantly higher BMI, waist/hip ratio, blood pressure, fasting and post load glucose, triglycerides, LDL cholesterol, and insulin levels, and lower HDL cholesterol and SHBG; associations with insulin and triglycerides persisted after adjustment for adiposity, linking low testosterone to insulin resistance and adverse cardiometabolic profile | Moderate |
| 33 | Andersson et al., 1994 | Cross sectional clinical study | Men and women with non–insulin-dependent diabetes mellitus and non-diabetic controls | Measurement of total testosterone, free testosterone (calculated), SHBG, insulin, and anthropometric parameters | Associations between sex hormones, SHBG, insulin resistance, and NIDDM | Men with NIDDM had significantly lower total testosterone and SHBG levels than controls, with insulin concentrations inversely correlated with testosterone and SHBG; women with NIDDM showed lower SHBG and higher free testosterone, with insulin resistance closely associated with hyperandrogenicity and adiposity, highlighting sex-specific hormonal patterns linked to insulin resistance | Moderate |
| 34 | Pitteloud et al., 2005 | Mechanistic clinical study with metabolic clamp | Men with normal glucose tolerance, impaired glucose tolerance, and diabetes (n = 21; subset n = 18 for LH pulsatility) | Hyperinsulinemic euglycemic clamp, frequent LH sampling, GnRH stimulation, and hCG testing to assess pituitary and testicular function | Relationship between insulin resistance and hypothalamic pituitary gonadal axis function | Insulin sensitivity (M value) was positively associated with total testosterone levels (r = 0.46) and strongly correlated with testosterone response to hCG (r = 0.73), while LH secretion and pituitary responsiveness were preserved, indicating that insulin resistance is associated with reduced Leydig cell testosterone secretion rather than central hypogonadism | Moderate |
| 35 | Osuna et al., 2006 | Cross sectional clinical study | Adult men stratified by BMI (normal weight, overweight, obese; n = 77) | Measurement of sex hormones (total and free testosterone, estradiol), SHBG, insulin, leptin, and insulin resistance (HOMA-IR) | Associations between obesity, insulin resistance, and sex hormone levels | Obese men had significantly lower total testosterone and SHBG and higher insulin levels than normal-weight and overweight men; total testosterone was inversely correlated with BMI, waist circumference, leptin, insulin, and HOMA-IR, supporting a close link between adiposity, insulin resistance, and androgen deficiency | Moderate |
| 36 | Rhoden et al., 2005 | Cross sectional clinical study | Sexually active men with diabetes mellitus and erectile dysfunction (n = 115) | Assessment of erectile function using the IIEF questionnaire and measurement of fasting glucose and HbA1c levels | Association between glycaemic control and severity of erectile dysfunction | Higher HbA1c levels were associated with more severe ED: among men with HbA1c <8%, severe ED occurred in 32%, compared with 46% in those with HbA1c ≥8%; HbA1c ≥11% was associated with a significantly higher prevalence of severe ED, particularly in men with longer diabetes duration (>5 years), indicating a link between poor glycaemic control and ED severity | Moderate |
| 37 | Rhoden et al., 2005 | Cross sectional study | Adult men with and without diabetes mellitus, age-matched (n = 746; 116 with DM) | Measurement of total and free testosterone, BMI and waist-to-hip ratio; stratified analysis by diabetes and adiposity | Association between diabetes, obesity, and subnormal testosterone levels | Subnormal free testosterone was present in 46% of diabetic men versus 24% of non-diabetics and was strongly associated with diabetes (OR 2.7; 95% CI 1.8 - 4.1), independent of BMI; in contrast, subnormal total testosterone was closely associated with obesity and central adiposity than with diabetes, highlighting distinct metabolic determinants of androgen deficiency | Moderate |
| 38 | Yagihashi et al., 2007 | Narrative review | Patients with diabetes mellitus and impaired glucose tolerance (human pathological and clinical studies) | Histopathological evaluation of peripheral nerves and review of experimental and clinical data | Pathological features and pathogenetic mechanisms of diabetic neuropathy | Diabetic neuropathy is characterized by progressive, pan-modal nerve fiber loss with a distal predominance, accompanied by endoneurial microangiopathy that inversely correlates with nerve fiber density; small-caliber nerve fiber damage begins early, even at the stage of impaired glucose tolerance, and precedes overt nerve trunk involvement; multiple mechanisms, including glycation, polyol pathway activation, oxidative stress, and altered protein kinase C signaling, contribute to nerve injury | Low |
| 39 | Sima et al, 1999 | Narrative review | Experimental animal models of type 1 and type 2 diabetes (mainly diabetic rodents) | Induction of diabetes and assessment of metabolic, vascular, and neural alterations | Pathogenetic mechanisms and structural functional features of diabetic neuropathy | Experimental studies demonstrate that diabetic neuropathy is a multifactorial disorder involving increased polyol pathway activity, non enzymatic glycation, oxidative stress, altered vasoactive signaling, and impaired neurotrophism; distinct functional and structural differences were identified between neuropathy in type 1 and type 2 diabetes, highlighting heterogeneity in underlying mechanisms | Low |
| 40 | Vernet et al., 1995 | Experimental animal models study (rats) | Diabetic rat models of type 1 (BB/WORdp) and type 2 diabetes (BBZ/WORdp) and age matched non diabetic controls | Determinations of serum glucose, testosterone, and penile reflexes (cups and flips) | Penile nitric oxide synthase activity, erectile function, and hormonal status | Both type 1 and type 2 diabetic rats exhibited severe erectile dysfunction (>95% reduction in erectile reflexes), marked reductions in serum testosterone, and significant decreases in penile NOS activity (74% in type 1 and 55% in type 2 diabetes), with parallel reductions in nNOS protein, supporting a common NOS related mechanism of diabetic ED partly linked to androgen deficiency | Low |
| 41 | Fowler et al., 1988 | Cross sectional clinical study | Men presenting with erectile dysfunction, including diabetic patients (n = 33) | Assessment of small-fiber neuropathy using thermal sensory threshold testing; electrophysiological testing of bulbocavernosus reflex | Presence of unmyelinated and small-fiber sensory neuropathy in diabetic erectile dysfunction | All men with clinically neuropathic erectile dysfunction showed abnormal thermal thresholds, indicating small-fiber neuropathy, whereas diabetics with non-neuropathic ED had normal results; standard electrophysiological testing failed to detect abnormalities in a substantial proportion of neuropathic cases, highlighting the sensitivity of small-fiber testing | Moderate |
| 42 | Hurt et al., 2002 | Experimental study (animal models) | Mouse models, including eNOS knockout mice | Cavernous nerve stimulation, intracavernosal papaverine injection, pharmacological inhibition of PI3K/Akt pathway | Role of PI3K/Akt-mediated eNOS activation in penile erection | Cavernous nerve stimulation and papaverine rapidly increased Akt and eNOS phosphorylation in penile tissue; inhibition of PI3K/Akt signaling reduced eNOS activation and erectile responses, while eNOS-deficient mice showed markedly impaired erections, demonstrating that Akt-dependent eNOS activation is essential for sustained NO production and full erectile functio | Moderate |
| 43 | McCulloch et al., 1980 | Cross sectional epidemiological study | Men with diabetes mellitus aged 20–59 years (n = 541) | Clinical survey assessing erectile function and diabetic complications | Prevalence of erectile dysfunction and associated clinical factors | Erectile dysfunction was present in 35% of diabetic men and was independently associated with age, diabetes treatment, retinopathy, symptomatic peripheral neuropathy, and autonomic neuropathy; the strongest associations were observed in patients with severe microangiopathy, particularly proliferative retinopathy and autonomic neuropathy, supporting a multifactorial etiology of diabetic ED | Moderate |
| 44 | Cho et al., 2006 | Multicentre cross sectional study | Korean men with type 2 diabetes mellitus (n = 1312) | Assessment of erectile function using modified IIEF-5 and standardized face-to-face questionnaires | Prevalence and risk factors for erectile dysfunction | Overall prevalence of ED was 65.4%; age, longer diabetes duration, higher HbA1c, insulin use, neuropathy, and macrovascular complications were independently associated with ED, with the highest risk observed in men aged >60 years with diabetes duration >10 years. | Moderate |
| 45 | Zheng et al., 2006 | Cross sectional clinical study | Cross-sectional clinical study | Face to face interviews, psychogenic assessment, penile hemodynamic testing, hormone measurements, and review of diabetic complications | Predictors of erectile dysfunction among diabetic men | Erectile dysfunction was diagnosed in 39.4% of participants and was independently associated with older age (OR 1.16), longer diabetes duration (OR 1.30), lower physical activity (OR 1.67), retinopathy (OR 1.15), neuropathy (OR 2.07), and depression (OR 1.46); men with ED exhibited more severe diabetic complications, supporting ED as a sentinel marker of underlying systemic disease | Moderate |
| 46 | Siu et al., 2001 | Cross sectional clinical study | Chinese men with diabetes mellitus (n = 486) | Structured interviews assessing erectile dysfunction (NIH 1993 definition) and review of clinical records for diabetic complications | Prevalence, severity, and risk factors for erectile dysfunction | The prevalence of ED was 63.6%, increasing markedly with age and diabetes duration; ED prevalence rose from 56.0% in men with diabetes duration <5 years to 72.0% in those with >20 years, with severity also increasing over time; ED was independently associated with diabetes duration, retinopathy, albuminuria, sensory neuropathy, and higher educational level, while age was the main determinant of ED severity | Moderate |
| 47 | Klein et al., 1996 | Population based cohort study (comparative study) | Men with long term insulin dependent diabetes mellitus (onset <30 years, duration ≥10 years; n = 365) | Self reported assessment of erectile dysfunction and evaluation of clinical and metabolic characteristics | Prevalence of erectile dysfunction and associated clinical factors | Erectile dysfunction was reported by 20% of participants and increased markedly with age and diabetes duration; ED was associated with severe retinopathy, peripheral neuropathy, cardiovascular disease, higher HbA1c, antihypertensive use, and higher BMI, indicating a strong link with cumulative microvascular and macrovascular damage | Moderate |
| 48 | Fedele et al., 1998 | Multicentre cross-sectional study | Men with type 1 and type 2 diabetes mellitus aged 20 - 69 years, attending diabetes clinics in Italy (n = 9,868) | Structured interviews assessing erectile dysfunction and collection of clinical, metabolic, and lifestyle data | Prevalence of erectile dysfunction and associated risk factors | Erectile dysfunction was reported by 35.8% of diabetic men, with prevalence increasing markedly with age and diabetes duration; poorer glycaemic control, longer disease duration, microvascular complications (retinopathy, nephropathy, neuropathy), arterial disease, and smoking were independently associated with ED, while ED was less frequently reported in men with type 2 than type 1 diabetes after age adjustment | Moderate |
| 49 | Fukui et al., 2011 | Cross-sectional clinical study | Men with type 2 diabetes mellitus (n = 197; vascular assessments in subsets of n = 125) | Assessment of erectile function using IIEF-5 and evaluation of albuminuria, pulse wave velocity, ankle-toe-brachial index, and diabetic complications | Association between erectile dysfunction, albuminuria, and subclinical atherosclerosis | Lower IIEF-5 scores were significantly associated with higher urinary albumin excretion, increased pulse wave velocity, and lower toe-brachial index; ED severity was greater in men with proliferative retinopathy, macroalbuminuria, neuropathy, or cardiovascular disease, supporting a close link between ED and both micro- and macrovascular disease | Moderate |
| 50 | Furukawa et al., 2017 (Dogo Study) | Multicentre cross-sectional study | Japanese men with type 2 diabetes mellitus aged 19- 65 years (n = 287) | Assessment of diabetic neuropathy using clinical criteria and erectile function using SHIM scores | Association between diabetic neuropathy and erectile dysfunction severity | Diabetic neuropathy was present in 47.0% of participants and severe ED in 39.0%; neuropathy was independently associated with severe ED, while no significant associations were observed with mild or moderate ED, nor with retinopathy or nephropathy, indicating a specific link between neuropathy and advanced erectile dysfunction | Moderate |
| 51 | Bandyk DF, 2018 | Narrative review | Patients with diabetes mellitus, particularly those with diabetic foot complications | - | Pathophysiology of diabetic neuropathy, peripheral arterial disease, and critical limb ischemia | Diabetic neuropathy and peripheral arterial occlusive disease act synergistically, leading to micro- and macrovascular dysfunction, tissue ischemia, and impaired healing; highlights the systemic nature of diabetic neurovascular damage | Low-moderate |
| 52 | Coppola A et al., 2023 | Retrospective cohort study | Men with type 2 diabetes and a recent, single diabetic foot ulcer. (n = 326) | Not applicable (observational study) | Wound healing, minor amputation, mortality, ulcer recurrence | ED prevalence was 56.1%. Wound healing was lower in men with ED vs no ED. Minor amputation and mortality were higher in the ED group. ED independently predicted mortality and ulcer recurrence, while absence of ED was associated with wound healing | Moderate |
| 53 | Henis O et al., 2011 | Cross sectional study | Men with type 2 diabetes (n=102); mean age 64.0 ± 8.2 years; mean diabetes duration 14.5 ± 8.9 years | Not applicable (observational study) | Presence and severity of erectile dysfunction (SHIM score) according to diabetic retinopathy severity | ED prevalence was 73.5%. ED was significantly more frequent in men with severe NPDR or PDR compared with controls (87.5% vs 50.0%, p<0.0001). Mean SHIM score was lower in severe retinopathy. ED and SHIM score were independently associated with retinopathy severity after adjustment for age, diabetes duration, ischemic heart disease, cerebrovascular disease, hypertension, dyslipidemia, and smoking | Moderate |
| 54 | Chew SK et al., 2013 | Cross sectional study (Hospital based) | Men with type 2 diabetes (n=324); mean age 65.2 ± 11.1 years | Comprehensive interview, a complete eye examination, fasting blood tests, and retinal and macula assessments using fundus images and optical coherence tomography | Presence of erectile dysfunction in relation to diabetic retinopathy (DR) and diabetic macular oedema (DME) | Compared with men without DR, those with any DR had a twofold higher odds of ED. Severe NPDR was associated with a fourfold increase in ED risk, and PDR with an approximately threefold increase. Diabetic macular oedema was not independently associated with ED, regardless of severity | Moderate |
| 55 | Katsimardou A et al., 2023 | Cross sectional observational study | Patients with T2DM (n=80; 50 men, 30 women) | Comprehensive interviews with International Index of Erectile Function and the Female Sexual Function Index questionnaires for males and females, respectively, and patients were evaluated for DKD | Association between diabetic kidney disease (DKD), eGFR, albuminuria and sexual dysfunction (SD), including ED and FSD | Sexual dysfunction was present in 80% of participants; 45% had DKD. Reduced eGFR was significantly associated with SD, ED and FSD. In multivariable linear regression analyses, SD and ED were significant determinants of lower eGFR values. Albuminuria/proteinuria showed weaker and non-independent associations. In women, lower eGFR correlated with poorer desire, arousal, lubrication and total FSFI scores, although these associations did not persist after multivariate adjustment | Moderate |
| 56 | Fu R et al., 2024 | Crosssectional study (non interventional observational) | 72 adult male patients with chronic kidney disease (CKD), China | Data collection included socio-demographic information, assessments via the 5-item version of the International Index of Erectile Function (IIEF-5), the Chinese version of the Premature Ejaculation Diagnostic Tool, the Patient Health Quentionnnaire-9 and the General Anxiety Disorder-7 | Prevalence of erectile dysfunction (ED) and premature ejaculation (PE) and their association with kidney function and psychological factors | ED was present in 56.9% of CKD patients and PE in 29.2%. Sexual dysfunction was associated with reduced eGFR, higher albumin-to-creatinine ratio, psychological distress (depression and anxiety), and medication use. Findings support a multifactorial link between CKD severity and male sexual dysfunction | Moderate |
| 57 | Muniandy V et al., 2025 | Cross sectional observational study | Men with type 2 diabetes mellitus and chronic kidney disease (n=280) | Questionnaires containing demographic and clinical information and the Malay Version of the Hospital Anxiety and Depression Scale (HADS). Sexual function was evaluated using the Malay Version of International Index of Erectile Function-5 (IIEF-5) | Prevalence of erectile dysfunction and associated clinical and metabolic determinants | ED prevalence was 95.0%. Independent factors associated with ED included metformin use, higher urea levels, higher HbA1c, while higher educational attainment was inversely associated with ED | Moderate |
| 58 | Dilixiati D et al., 2024 | Systematic review and meta-analysis | 58 studies; 66,925 men with diabetes mellitus (with and without erectile dysfunction) | Systematic review, encompassing studies published in the PubMed, Scopus and Embase databases up to August 24th, 2023 | Identification of risk factors associated with erectile dysfunction in men with diabetes | ED risk was significantly associated with older age (OR 1.31), higher HbA1c (OR 1.44), longer diabetes duration (OR 1.39), diabetic neuropathy (OR 3.47), retinopathy (OR 3.01), diabetic foot (OR 3.96), cardiovascular disease (OR 1.92), hypertension (OR 1.74), microvascular disease (OR 2.14), vascular disease (OR 2.75), nephropathy (OR 2.67), metabolic syndrome (OR 2.22), depression (OR 1.82), smoking (OR 1.32), and diuretic treatment (OR 2.42) | Low-moderate |
| 59 | Blair YA et al., 2024 | Prospective cohort analysis within a randomized clinical trial (DPPOS) | 568 men with prediabetes (PreD) or type 2 diabetes (T2D) from the Diabetes Prevention Program Outcomes Study | Prior randomization to intensive lifestyle intervention (ILS), metformin, or placebo (DPP); no ED-specific intervention | Prevalence of erectile dysfunction and identification of clinical, metabolic, and psychosocial predictors | ED prevalence was 38% overall, similar in PreD (41%) and T2D (37%). Age increased ED risk in all men. In PreD, ILS was associated with reduced odds of ED versus placebo (OR 0.35; 95% CI 0.13–0.94), whereas metformin showed no effect. In T2D, metabolic syndrome (OR 1.85; 95% CI 1.14–3.01) and depression (OR 2.05; 95% CI 1.10–3.79) were independently associated with ED | Low-moderate |
| 60 | Viigimaa M et al., 2020 | Expert consensus, position paper | Men with arterial hypertension, with or without erectile dysfunction | Review of antihypertensive drug classes and their impact on erectile function; no experimental intervention | Association between arterial hypertension, erectile dysfunction, cardiovascular risk, and effects of antihypertensive treatment | ED frequently precedes overt coronary artery disease, supporting the artery size hypothesis. Hypertension and ED share common risk factors and mechanisms, including endothelial dysfunction and atherosclerosis. Treated hypertensive patients report ED more frequently than untreated ones. Antihypertensive drug classes show heterogeneous effects: diuretics and traditional beta-blockers are associated with worse erectile outcomes, whereas angiotensin receptor blockers and nebivolol show neutral or favorable profiles | Low |
| 61 | Vrentzos GE et al., 2007 | Narrative review | Men with erectile dysfunction and cardiometabolic risk factors, including dyslipidemia | Review of lipid abnormalities, endothelial dysfunction, and pharmacological treatments (statins, PDE5 inhibitors) | Association between dyslipidemia and erectile dysfunction; endothelial and NO related mechanisms | Dyslipidemia is identified as an independent and contributory risk factor for ED. Elevated total and LDL cholesterol and reduced HDL cholesterol are associated with impaired erectile function. Oxidized LDL directly disrupts corpus cavernosum smooth muscle relaxation through endothelial dysfunction and reduced nitric oxide bioavailability. ED is highlighted as an early marker of subclinical vascular disease. Statin therapy may improve erectile function and enhance responsiveness to PDE5 inhibitors by restoring endothelial function | Moderate |
| 62 | Bosevski M et al., 2019 | Prospective cohort study | 264 patients with type 2 diabetes and established coronary artery disease | Serial carotid ultrasound (CIMT, plaque detection, carotid stenosis) during follow-up | Cardiovascular events (new angina, myocardial infarction, stroke) | Progression of carotid artery disease was frequent (CIMT progression 86.8%, new plaques 41.8%). New carotid plaque formation was a strong independent predictor of total cardiovascular events (OR 13.5, 95% CI 1.68–108.4). CIMT progression showed modest association with new angina, while plaque progression carried greater prognostic value than CIMT alone | Moderate |
| 63 | Moon KH et al., 2019 | Narrative review | Men with obesity and erectile dysfunction | Review of metabolic pathways (insulin resistance, leptin resistance, inflammation, oxidative stress) and combined therapeutic approaches (metformin, PDE5 inhibitors) | Association between obesity and erectile dysfunction; impact of metabolic modulation on erectile outcomes | Obesity and ED share a common pathophysiological background characterized by inflammation, oxidative stress, insulin and leptin resistance. Severity of ED correlates with obesity-related comorbidities. Metformin improves metabolic derangements, reduces oxidative stress and inflammation, and has been associated with improvements in erectile function in experimental and clinical studies. Combination therapy with metformin and PDE5 inhibitors improves erectile function in insulin resistant, sildenafil non responders | Moderate |
| 64 | Liu Y et al., 2023 | Cross sectional observational study | 878 men attending an andrology clinic in Central China | Erectile function was assessed by the International Index of Erectile Function (IIEF) scores. Questionnaires included questions about demographic characteristics (age, height, weight, educational status), lifestyle habits (drinking, smoking, sleep time), and medical history. | Presence and severity of erectile dysfunction assessed by IIEF | ED prevalence was 53.1%. BMI was higher in men with ED compared to non ED. Obesity was associated with increased ED risk. Obesity was strongly associated with moderate-severe ED, independent of demographic and lifestyle confounders | Moderate |
| 65 | Wing RR et al., 2010 | Randomized controlled tria | 372 overweight/obese men with type 2 diabetes at baseline; | Intensive lifestyle intervention (ILI: weight loss + physical activity) vs diabetes support and education (DSE) | Change in erectile function assessed by IIEF-EF domain | ILI achieved greater weight loss (9.9% vs 0.6%) and fitness improvement (22.7% vs 4.6%). EF scores improved modestly in ILI compared with DSE (P = 0.04). EF category improved in 22% of ILI vs 23% of DSE, but worsening was significantly less frequent in ILI (8% vs 20%; P = 0.006), indicating preservation rather than recovery of erectile function | Low |
| 66 | Sattar N et al., 2023 | Observational study | 679,072 individuals with type 2 diabetes and 2,643,800 matched controls from the Swedish National Diabetes Register | Observational; longitudinal assessment of cardiometabolic risk factor control | Incident coronary artery disease, myocardial infarction, cerebrovascular disease, and heart failure | Marked decline in atherosclerotic events over time in T2DM, but heart failure incidence plateaued after 2013. Glycated hemoglobin and systolic blood pressure were most strongly associated with atherosclerotic events, while body mass index explained >30% of heart failure risk. Individuals with T2DM and all risk factors within target had no excess atherosclerotic risk compared with controls, except for heart failure (HR 1.50, 95% CI 1.35- 1.67) | Low |
| 67 | Mosenzon O et al., 2021 | Cross sectional study | 9,823 adults with type 2 diabetes from 13 countries across five continents | Observational; standardized assessment of cardiovascular disease prevalence and treatment patterns | Prevalence of established cardiovascular disease and atherosclerotic cardiovascular disease | Overall weighted prevalence of CVD was 34.8% (95% CI 32.7–36.8) and of atherosclerotic CVD 31.8% (95% CI 29.7–33.8). Median diabetes duration was 10.7 years and median HbA1c 7.3%. Use of glucose-lowering agents with proven cardiovascular benefit was low (21.9%) and similar in patients with and without established CVD | Low |
| 68 | Montorsi P et al., 2005 | Narrative review | Men with erectile dysfunction and/or coronary artery disease, based on clinical and epidemiological evidence | Not aplicable | Conceptual framework linking erectile dysfunction and coronary artery disease | Proposes the “artery size hypothesis”, which states that atherosclerosis affects all vascular beds similarly, but symptoms appear earlier in smaller arteries. Because penile arteries are smaller than coronary arteries, erectile dysfunction often precedes clinical manifestations of coronary artery disease, whereas patients with established CAD frequently report ED | Low |
| 69 | Montorsi P et al., 2006 | Observational, cross sectional study | 285 men with angiographically proven coronary artery disease (acute coronary syndrome or chronic coronary syndrome) and 95 control subjects with normal coronary arteries | Diagnostic coronary angiography with quantitative assessment (vessel involvement and Gensini score), erectile function evaluation using the International Index of Erectile Function (IIEF-EFD), and laboratory assessment of cardiovascular and hormonal risk factors | Prevalence of erectile dysfunction according to coronary clinical presentation (ACS vs CCS) and extent of coronary artery disease (single- vs multi-vessel disease) | Erectile dysfunction prevalence increased with greater atherosclerotic burden and chronic coronary syndrome. ED prevalence was 22% in ACS with single-vessel disease, 55% in ACS with multi-vessel disease, and 65% in chronic coronary syndrome. In patients with chronic coronary syndrome and ED, erectile dysfunction preceded coronary artery disease in 93% of cases by a mean of 24 months. Multivessel disease and chronic coronary syndrome were independent predictors of ED | Moderate |
| 70 | Akili H et al., 2007 | Cross sectional observational study | 105 men with chest pain, positive exercise treadmill test, and no previous diagnosis of coronary artery disease | Exercise treadmill testing followed by diagnostic coronary angiography, and assessment of erectile function using the Sexual Health Inventory for Men (SHIM). | Significance of erectile dysfunction in males with a positive exercise treadmill test (ETT) to predict the severity of coronary artery disease (CAD) | The SHIM score is an independent parameter to define the presence of significant lesions in two or more coronary arteries (odds ratio, 0.84; 95% CI, 0.73-0.97; P = 0.019). | Moderate |
| 71 | Zhao B et al., 2019 | Systematic review and metaanalysis | 25 studies including 154,794 men from cohort and observational studies | Literature review and meta-analysis | Cardiovascular disease (CVD), coronary heart disease (CHD), stroke, all-cause mortality | Erectile dysfunction was associated with a significantly increased risk of CVD (RR 1.43), CHD (RR 1.59), stroke (RR 1.34), and all-cause mortality (RR 1.33), all p<0.001. Risk was higher in men ≥55 years, with shorter ED duration (<7 years), higher prevalence of diabetes (≥20%) and smoking (≥40%). Severe ED predicted the highest risk of CVD and mortality | Low |
| 72 | Fang SC et al., 2015 | Longitudinal population-based cohort study | 965 men free of cardiovascular disease at baseline | ED was assessed with the five-item International Index of Erectile Function and classified as no ED/transient ED/persistent ED. CVD risk was assessed with 10-year Framingham CVD risk algorithm. | Framingham 10-year cardiovascular risk and change in Framingham risk over time | Transient and persistent erectile dysfunction were associated with higher Framingham cardiovascular risk and greater increase in risk over time, independent of baseline risk and other covariates. Persistent ED was associated with a higher Framingham risk particularly in men <50 years. Associations were stronger in younger men | Low |
| 73 | Gazzaruso C et al., 2011 | Cross sectional study | 293 men with newly diagnosed type 2 diabetes and no apparent vascular complications | Type 2 diabetes mellitus were evaluated to find patients with asymptomatic CAD and to assess the presence of diabetic complications with exercise stress testing. The presence and the degree of ED were assessed by the validated International Index Erectile Function-5 (IIEF-5) questionnaire | Presence of asymptomatic coronary artery disease (angiographically proven) | Erectile dysfunction was significantly more prevalent in men with asymptomatic CAD than in those without CAD (37.8% vs 15.1%). ED independently predicted silent CAD (OR 4.4; 95% CI 2.1–9.0). Adding ED to current screening risk factors increased sensitivity from 62% to 89%, improved negative predictive value from 82% to 94%, and reduced missed silent CAD cases from 37.8% to 10.8%, with minimal loss of specificity | Moderate |
| 74 | Turek SJ et al., 2013 | Cross sectional analysis within a longitudinal cohort | 301 men with ≥50 years of type 1 diabetes (Joslin Medalist Study | Self assessment of SD in males of the Medalist cohort by self-reported sexual problems with CVD. Sexual dysfunction is validated through the use of the abbreviated International Index of Erectile Dysfunction (IIEF). | Presence of cardiovascular disease | Sexual dysfunction was reported by 69.8% of participants. SD was independently associated with cardiovascular disease after adjustment for age, HbA1c, and BMI (OR 1.9; 95% CI 1.0–3.5). SD was associated with adverse cardiometabolic and inflammatory profiles (higher HbA1c, BMI, total cholesterol, IL-6; lower HDL). Retinal, neural, and renal microvascular complications were not associated with SD | Moderate |
| 75 | Nehra A et al., 2012 | Expert consensus / clinical guideline | Men with erectile dysfunction with or without known cardiovascular disease | Cardiovascular risk stratification, exercise capacity assessment, and guideline-based management of ED | Identification of occult CVD; cardiovascular risk associated with sexual activity | The consensus highlights ED as an early clinical marker of cardiovascular disease, recommending systematic cardiovascular risk assessment in men with ED, including stress testing when indicated, to guide both sexual and cardiovascular management | Low |
| 76 | Hippisley-Cox J et al., 2017 | Prospective open cohort study | Adults aged 25–84 years without baseline CVD from UK primary care (derivation cohort: 7.89 million; validation cohort: 2.67 million) | Development and validation of the QRISK3 cardiovascular risk prediction model, including erectile dysfunction diagnosis or treatment as a candidate risk factor in men | Incident cardiovascular disease (coronary heart disease and stroke) | Erectile dysfunction met inclusion criteria and was incorporated as an independent variable in the QRISK3 algorithm, which demonstrated good calibration and discrimination for 10-year CVD risk prediction | Low |
| 77 | Gazzaruso et al., 2008 | Prospective cohort study | Men with type 2 diabetes and angiographically proven asymptomatic CAD (n = 291) | Erectile dysfunction assessed by IIEF-5; use of statins and PDE-5 inhibitors | Major adverse cardiovascular events (MACE) and all-cause mortality | ED prevalence was higher among patients with MACE (61.2% vs 36.4%; *p* = 0.001), and ED independently predicted MACE (HR 2.1; 95% CI 1.6–2.6; *p* < 0.001); statin use was associated with reduced MACE risk (HR 0.66; 95% CI 0.46–0.97), while PDE-5 inhibitor use showed a borderline protective association | Moderate |
| 78 | Batty et al., 2010 | Prospective cohort study (post-hoc analysis of RCT cohort) | Men with type 2 diabetes aged 55–88 years from the ADVANCE trial (n = 6,304) | Erectile dysfunction assessed by self-report at baseline and follow-up | Incident cardiovascular disease (overall CVD, CHD, cerebrovascular events) | Baseline ED was independently associated with higher risk of all CVD events (HR 1.19; 95% CI 1.08–1.32), coronary heart disease (HR 1.35; 95% CI 1.16–1.56), and cerebrovascular disease (HR 1.36; 95% CI 1.11–1.67); persistent ED conferred the highest risk | Low |
| 79 | Grant et al., 2013 | Narrative review | Men with erectile dysfunction | Clinical assessment of ED and associated cardiometabolic risk factors | Cardiovascular disease, diabetes mellitus, depression | ED is recognised as an early clinical marker of CVD and diabetes, sharing major risk factors with cardiometabolic disease and frequently under-recognised in routine clinical practice | Moderate |
| 80 | Zhang et al., 2025 | Mendelian randomization study | Genetic datasets for erectile dysfunction and myocardial infarction derived from large genome-wide association data | Genetic instruments for ED and MI; multivariable and mediation Mendelian randomization analyses | Myocardial infarction | Genetically predicted ED was associated with an increased risk of MI, with consistent findings in validation datasets and no evidence of horizontal pleiotropy; smoking showed partial mediation | Low |
| 81 | Hermans et al., 2009 | Crosssectional observational study | Men with type 2 diabetes mellitus attending outpatient clinics (n = 221) | Erectile dysfunction assessed using IIEF-5; comparison between ED(+) and ED(–) groups | Microangiopathy (retinopathy, neuropathy, albuminuria), metabolic syndrome, macrovascular disease, UKPDS CV risk | ED was associated with a higher prevalence of metabolic syndrome, central adiposity, and microangiopathy (retinopathy, neuropathy, albuminuria), as well as increased peripheral vascular and cerebrovascular events, despite similar glycaemic control and UKPDS 10-year CV risk | Moderate |
| 82 | Birgün et al., 2025 | Narrative review | Patients with diabetes mellitus and sexual dysfunction and/or peripheral artery disease | Review of vascular and neural mechanisms linking sexual dysfunction and PAD in diabetes | Peripheral artery disease, sexual dysfunction, cardiovascular risk | Sexual dysfunction and peripheral artery disease share common vascular and neural mechanisms in diabetes, including endothelial dysfunction, atherosclerosis, and diabetic neuropathy, supporting sexual dysfunction as an early marker of systemic vascular impairment and increased cardiovascular risk | Moderate |
| 83 | Wang & Ni, 2024 | Cross sectional study | Adult men from NHANES (n = 2,394), aged ≥40 years | Erectile dysfunction assessed by self-reported erection problems; PAD defined by ABI <0.9 | Peripheral arterial disease | ED was independently associated with a higher prevalence of PAD after multivariable adjustment (OR 2.05; 95% CI 1.24–3.39), with consistent associations across multiple subgroups | Moderate |
| 84 | Polonsky et al., 2009 | Prospective observational screening study | Men referred for stress testing without known PAD (n = 690) | ED assessed by IIEF; PAD screened using ankle–brachial index (ABI) | Peripheral arterial disease | ED was independently associated with PAD (OR 1.97; 95% CI 1.32–2.94), with a higher prevalence of PAD in men with ED compared with those without ED (32% vs 16%) and a stepwise increase in PAD prevalence with increasing ED severity | Moderate |
| 85 | Yuan et al., 2020 | Cross sectional observational study | Chinese men with type 2 diabetes mellitus without clinical CVD (n =71) | Erectile function assessed by IIEF-5; carotid and lower limb Doppler ultrasound | Subclinical atherosclerosis (carotid IMT, peripheral plaques) and ED | ED prevalence was 71.2%; men with ED had higher carotid IMT and more frequent lower limb plaques; carotid IMT >0.75 mm and peripheral plaques remained independently associated with ED severity after adjustment | Moderate |
| 86 | Schwarz et al., 2006 | Narrative review | Men with chronic heart failure | Clinical assessment of ED in the context of HF and standard HF therapies | Erectile dysfunction prevalence and contributing factors in HF | ED is highly prevalent in men with heart failure and reflects shared cardiometabolic risk factors as well as HF specific physiological and psychological mechanisms; standard HF therapies (e.g., beta blockers, thiazide diuretics) may further worsen sexual function | Moderate |
| 87 | Carella et al., 2023 | Narrative review | Narrative review | Clinical assessment and management of ED in HF, including pharmacological and non-pharmacological strategies | Erectile dysfunction prevalence, cardiovascular events, mortality | ED is a frequent complication of heart failure and may act as a predictor of cardiovascular events and mortality; its pathophysiology is multifactorial, and individualized management may improve sexual function and cardiopulmonary parameters | Moderate |
| 88 | Ma et al.,2008 | Prospective cohort study | Chinese men with T2DM and no clinically overt CVD at baseline (n = 2,306) | Presence of erectile dysfunction at baseline, defined by NIH criteria | Incident coronary heart disease (CHD) events | ED was present in 26.7% of participants and was associated with a higher incidence of CHD events (19.7 vs 9.5 per 1,000 person years); ED remained an independent predictor of CHD after multivariable adjustment (HR 1.58; 95% CI 1.08 - 2.30) | Low |
| 89 | Seidu et al., 2022 | Systematic review and meta analysis | Adults with erectile dysfunction, with and without diabetes mellitus (18 studies) | Presence of ED; use of phosphodiesterase-5 inhibitors (PDE5i) | Cardiovascular disease, MACE, CHD, stroke, all cause mortality | ED was associated with increased risk of CVD and mortality in both the general population and people with diabetes (RRs 1.3 - 1.7); in diabetes, ED was associated with higher risks of composite CVD/MACE and CHD; limited RCT data showed no significant reduction in cardiovascular outcomes with PDE5i use | Low |
| 90 | Khatana et al., 2008 | Prospective interventional study | Men with type 2 diabetes mellitus (veterans; n = 41) | Short term behavioral and pharmacologic cardiovascular risk reduction program | Change in erectile function (IIEF-5 score) | Improvements in blood pressure and glycaemic control were significantly associated with improvements in erectile function; changes in systolic and diastolic blood pressure and maintenance of HbA1c <7% correlated with IIEF-5 improvement | Moderate |
| 91 | Fu et al., 2024 | Narrative review | Individuals with type 2 diabetes and non diabetic populations with cardiovascular and renal disease | Use of SGLT2 inhibitors, GLP-1 receptor agonists, and DPP-4 inhibitors | Cardiovascular and renal outcomes; cardiorenal protection mechanisms | Emerging antidiabetic drugs (SGLT2i, GLP-1RAs, DPP-4i) demonstrate consistent cardiorenal protective effects through glucose dependent and independent mechanisms, improving cardiovascular and renal outcomes beyond glycaemic control | Moderate |
| 92 | Salvatore et al., 2022 | Narrative review | Individuals with type 2 diabetes and patients with cardiovascular and renal disease | Use of SGLT2 inhibitors | Cardiovascular and renal outcomes; mechanisms of cardiorenal protection | SGLT2 inhibitors exert cardiorenal protective effects beyond glycaemic control through haemodynamic changes (diuresis, natriuresis), metabolic shifts in myocardial substrate use, improved vascular function, and antifibrotic, anti inflammatory, and antioxidative mechanisms | Moderate |
| 93 | Yang et al., 2025 | Systematic review and meta analysis | Adults with diabetes mellitus and erectile dysfunction (3 clinical studies included) | Use of antidiabetic drugs (GLP-1RAs, metformin, thiazolidinediones, SGLT2i, insulin, sulfonylureas) | Change in erectile function | GLP-1 receptor agonists showed greater improvement in erectile function compared with metformin (Z = 2.41; *P* = 0.02), with a more pronounced effect in overweight or obese patients; other antidiabetic drug classes showed potential benefits, but evidence was limited and heterogeneous | Moderate |
| 94 | Endocrine Practice, 2024 (TriNetX cohort) | Retrospective cohort study (real-world data) | Male patients with type 2 diabetes mellitus (SGLT2i users n=322,000; non users n=1,280,000) | Use of SGLT2 inhibitors vs non-use (propensity score matched) | Incident erectile dysfunction; use of phosphodiesterase-5 inhibitors | SGLT2 inhibitor use was associated with a higher incidence of ED diagnoses (RR 1.51; *P* < 0.001) and greater use of PDE5 inhibitors (RR 1.57; *P* < 0.001) compared with non users | Moderate |
| 95 | Kounatidis et al., 2025 | Narrative review | Narrative review | Use of glucagon-like peptide-1 receptor agonists (GLP-1 RAs) | Erectile function; mechanistic links with microvascular disease and metabolic control | GLP-1 RAs may improve erectile function through weight loss, improved glycaemic control, endothelial function, and androgen profile; however, available evidence is heterogeneous and preliminary signals of a possible association with ED have also been reported, precluding definitive conclusions | Moderate |
| 96 | Bajaj et al., 202 | Double-blind, placebo-controlled randomized trial | Men with type 2 diabetes mellitus participating in REWIND (n = 3,725) | Dulaglutide vs placebo | Incident moderate or severe erectile dysfunction; change in erectile function score (IIEF) | Dulaglutide was associated with a lower incidence of moderate or severe ED compared with placebo (21.3 vs 22.0 per 100 person years; HR 0.92, 95% CI 0.85 - 0.99) and a smaller decline in erectile function scores over time | Low |
| 97 | Giagulli VA et al., 2015 | Retrospective observational study | Obese men with type 2 diabetes, overt hypogonadism, and erectile dysfunction (n = 43) | Addition of liraglutide to lifestyle intervention, metformin, and testosterone replacement therapy | Change in erectile function (IIEF score); metabolic and hormonal parameters | In men with inadequate metabolic control on testosterone plus metformin, the addition of liraglutide was associated with improved glycaemic control, weight loss, increased testosterone and SHBG levels, and a significant further improvement in erectile function scores; no further ED improvement was observed in patients who did not receive liraglutide | Moderate |
| 98 | An et al., 2024 | Mendelian randomization study | Genetic datasets representing individuals with type 2 diabetes, obesity, hypertension, and cardiovascular disease | Genetically proxied exposure to GLP-1 receptor agonists | Erectile dysfunction | Genetically proxied GLP-1RA exposure was associated with a lower risk of ED (OR 0.49; 95% CI 0.43- 0.57); mediation analyses suggested that a small proportion of this effect was mediated through reductions in T2D, obesity, hypertension, and CVD | Moderate |
| 99 | Pourabhari Langroudi et al., 2025 | Cross sectional study | Male patients reported in FAERS receiving GLP-1 receptor agonists | Exposure to GLP-1 receptor agonists (tirzepatide, semaglutide, dulaglutide, exenatide, lixisenatide, liraglutide) | Reports of male sexual dysfunction (erectile dysfunction, orgasmic dysfunction, reduced libido) | Among 182 reported cases, disproportionality analyses showed statistically significant signals but low effect sizes (ROR =0.41), indicating a weak association between GLP-1RA use and male sexual dysfunction; overall clinical risk appeared low | High |
| 100 | UKPDS Group, 1998 | Randomized controlled trial | Overweight patients with newly diagnosed type 2 diabetes mellitus (n = 753 in metformin RCT) | Intensive glucose control with metformin vs conventional therapy (diet) or other glucose-lowering agents | Diabetes related clinical endpoints, cardiovascular outcomes, all-cause mortality | Intensive treatment with metformin significantly reduced diabetes related endpoints (32%), diabetes related mortality (42%), and all cause mortality (36%) compared with conventional therapy, and showed superior outcomes compared with sulphonylureas or insulin | Low |
| 101 | Shah et al., 2023 | Narrative review | Patients with type 2 diabetes mellitus and chronic kidney disease, with or without cardiovascular disease | Use of finerenone | Cardiovascular and renal outcomes | Evidence from large clinical trials (FIDELIO-DKD, FIGARO-DKD) shows that finerenone reduces cardiovascular events and slows kidney disease progression in patients with T2DM and CKD, supporting a role in cardiorenal protection beyond glycaemic control | Moderate |
| 102 | Doumas & Douma, 2006 | Narrative review | Men with hypertension, with or without erectile dysfunction | Antihypertensive drug classes (beta-blockers, diuretics, ACE inhibitors, calcium channel blockers, ARBs); use of PDE5 inhibitors | Erectile function; treatment adherence | Older generation antihypertensive drugs (beta blockers, diuretics, centrally acting agents) were associated with worsening erectile function, whereas newer agents (ACE inhibitors, calcium channel blockers) appeared neutral and angiotensin receptor blockers showed potential beneficial effects; PDE5 inhibitors were considered safe and effective in hypertensive patients | Moderate |
| 103 | Dash et al., 2024 | Systematic review | Hypertensive men with erectile dysfunction (12 studies; n = 11,672) | Angiotensin receptor blockers compared with other antihypertensive drug classes | Erectile function, sexual activity, treatment satisfaction | Across included studies, ARBs were associated with significant improvements in erectile function, frequency of sexual activity, and overall sexual satisfaction compared with older-generation antihypertensive agents, while effectively reducing blood pressure | Moderate |
| 104 | Ismail et al., 2019 | Meta analysis of randomized controlled trials | Hypertensive men, with or without diabetes (4 RCTs; n = 2,809) | Angiotensin receptor blockers (valsartan, losartan, telmisartan) vs conventional therapy or placebo | Erectile function and sexual activity | ARBs were associated with improved sexual activity, particularly with valsartan (MD 0.71; 95% CI 0.66 - 0.76); however, improvements in erectile function were not consistently observed across ARBs, with substantial heterogeneity for erectile function outcomes | Moderate |
| 105 | Vlachopoulos et al., 2003 | Randomized, placebo controlled, double-blind crossover study | Men with coronary artery disease, including hypertensive patients (n = 24) | Sildenafil 50 mg vs placebo | Arterial stiffness and wave reflection (pulse wave velocity, augmentation index) | Sildenafil significantly reduced aortic stiffness (PWV -0.65 m/s) and wave reflection (augmentation index -4.47%), indicating improved central arterial function beyond its effects on erectile performance | Moderate |
| 106 | Santi et al., 2015 | Systematic review and meta-analysis of RCTs | Men with type 2 diabetes mellitus (6 RCTs; n = 476) | Chronic phosphodiesterase-5 inhibitor therapy (sildenafil) vs placebo | Endothelial function markers (flow-mediated dilation, inflammatory biomarkers) | Endothelial function markers (flow-mediated dilation, inflammatory biomarkers) | Moderate |
| 107 | Sanchez et al., 2017 | Narrative review | Men with erectile dysfunction, metabolic syndrome, and cardiovascular risk factors | Clinical evaluation and risk stratification in men with ED | Cardiovascular disease risk; metabolic syndrome | ED shares common risk factors with metabolic syndrome and cardiovascular disease and is considered an independent predictor of future cardiovascular events; clinical encounters for ED represent an opportunity for cardiovascular risk assessment and stratification | Moderate |
| 108 | Roy et al., 2003 | Narrative review | Patients with erectile dysfunction and cardiovascular disease; experimental models of cardiac disease | Phosphodiesterase 5 inhibitors | Cardiovascular outcomes; cardiac structure and function; safety | Preclinical studies suggest potential cardioprotective effects of PDE5 inhibitors through improved endothelial function and modulation of ischemia–reperfusion injury and cardiac remodeling; however, clinical trials in heart failure and coronary artery disease have shown mixed or neutral efficacy despite a favorable safety profile | Moderate |
| 109 | Kloner et al., 2023 | Retrospective observational study | Men with erectile dysfunction and cardiovascular risk factors, without prior MACE (n =72,498) | Exposure to phosphodiesterase-5 inhibitors vs non exposure | Major adverse cardiovascular events (MACE) and all cause mortality | PDE-5i exposure was associated with a lower risk of MACE (HR 0.87; 95% CI 0.79 - 0.95) and overall mortality (HR 0.75; 95% CI 0.65 - 0.87); risk reduction showed a dose response relationship and was also observed in men with type 2 diabetes | Moderate |
| 110 | Soulaidopoulos et al., 2024 | Systematic review and meta-analysis | Men with erectile dysfunction, with or without coronary artery disease (16 cohort studies; n=1,257,759) | Use of phosphodiesterase-5 inhibitors vs placebo, other ED treatments, or no treatment | Major adverse cardiovascular events (MACE) and all cause mortality | PDE5i use was associated with a significant reduction in MACE (RR 0.78; 95% CI 0.69-0.89) and all cause mortality (RR 0.70; 95% CI 0.56-0.87) over long term follow-up | Moderate |
| 111 | Koka et al., 2012 | Experimental preclinical study | Diabetic db/db mouse model of type 2 diabetes | Chronic treatment with long acting PDE5i (tadalafil) | Cardiac proteomic profile; myocardial structure and redox signaling | Chronic tadalafil treatment reversed diabetes-associated proteomic alterations related to cytoskeletal organization, contractile dysfunction, and oxidative stress, improving myocardial structure and redox balance in diabetic hearts | Moderate |
| 112 | Koka et al., 2014 | Experimental preclinical study | Diabetic db/db mouse model of type 2 diabetes; wild-type controls | Chronic treatment with long acting PDE-5 inhibitor (tadalafil) for 8 weeks | Mitochondrial function, NO/SIRT1/PGC-1α signaling, oxidative stress, and left ventricular function | Chronic tadalafil treatment activated NO-SIRT1-PGC-1α signaling, improved mitochondrial oxidative phosphorylation, reduced mitochondrial ROS generation, and attenuated left ventricular dysfunction in diabetic hearts | Moderate |

**Appendix A:** Analysis Report: Prevalence, incidence, risk factors and complications related to erectile dysfunction among people with type 2 diabetes mellitus Incidence and Prevalence

TriNetX is the global federated health research network providing access to electronic medical records (diagnoses, procedures, medications, laboratory values, genomic information) across large healthcare organizations (HCOs). This report was run on the set of HCOs grouped into a network called Global Collaborative Network. This network included 165 HCO(s). This report describes an Incidence and Prevalence Analysis, named WITH T2DM, generated by the TriNetX platform on Dec 9, 2025, 12:38:46 UTC. This analysis describes events of interests for the cohort T2DM 09.12 with 4,321,390 patients.

This analysis was run by Bogdan Vlacho (bvlacho@idiapjgol.info) and downloaded by Bogdan Vlacho (bvlacho@idiapjgol.info).

Analysis Inputs for definition of cohort with T2DM

*Methods*

The analysis process includes two main steps: 1) Defining the cohort through query criteria; 2) Setting up and running the analysis.

*Cohorts definition*

This section lists all terms used in the definitions of the cohort.

*Query Criteria for the Cohort (query name: T2DM 09.12)*

This query was run on the network Global Collaborative Network with 165 HCO(s) queried and 165 HCO(s) responded. A total of 161 provider(s) responded with patients. The final cohort included 4,321,390 patients who matched the query criteria listed in the table below.


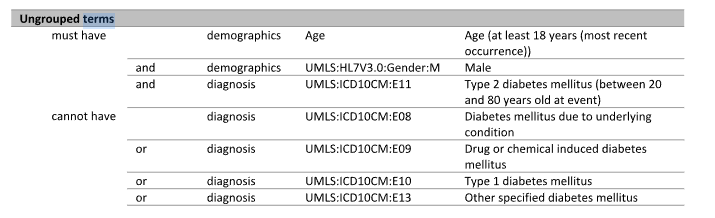


*Lookback Period*

A lookback period determines how far back in the database the analysis will look when determining whether cases are incident, prevalent, or neither. Patients who had an instance of the event of interest during the lookback period will be considered a prevalent case if they have an instance of the event of interest in the time window. The following is the setting used for this analysis’ lookback period:

Look back from any time to one day before the start of each time window.

*Strata Inputs*

Below are the selected strata inputs used to stratify the results of the analysis:

Age: Selected

Sex: Not Selected

Race: Selected

Ethnicity: Selected

# Analysis Inputs for cohort without diabetes

# Methods

The analysis process includes two main steps: 1) Defining the cohort through query criteria; 2) Setting up and running the analysis.

## Cohorts definition

This section lists all terms used in the definitions of the cohort.

### Query Criteria for the Cohort (query name: without diabetes)

This query was run on the network Global Collaborative Network with 168 HCO(s) queried and 168 HCO(s) responded. A total of 168 provider(s) responded with patients. The final cohort included 67,108,795 patients who matched the query criteria listed in the table below. For the text representation of the query criteria please see Appendix A.

|  | | | | | |
| --- | --- | --- | --- | --- | --- |
| Ungrouped terms | | | | | |
|  | must have |  | demographics | Age | Age (at least 20 years (most recent occurrence)) |
|  |  | and | demographics | UMLS:HL7V3.0:Gender:M | Male |
|  | cannot have |  | diagnosis | UMLS:ICD10CM:E08 | Diabetes mellitus due to underlying condition |
|  |  | or | diagnosis | UMLS:ICD10CM:E09 | Drug or chemical induced diabetes mellitus |
|  |  | or | diagnosis | UMLS:ICD10CM:E10 | Type 1 diabetes mellitus |
|  |  | or | diagnosis | UMLS:ICD10CM:E13 | Other specified diabetes mellitus |
|  |  | or | diagnosis | UMLS:ICD10CM:E11 | Type 2 diabetes mellitus (between 20 and 80 years old at event) |

## Time Window Inputs

| Time Window Start | Time Window End | Label |
| --- | --- | --- |
| 2024-12-09 | 2025-12-09 |  |

## Lookback Period

A lookback period determines how far back in the database the analysis will look when determining whether cases are incident, prevalent, or neither. Patients who had an instance of the event of interest during the lookback period will be considered a prevalent case if they have an instance of the event of interest in the time window. The following is the setting used for this analysis’ lookback period:

Look back from 360 days to one day before the start of each time window.

## Strata Inputs

Below are the selected strata inputs used to stratify the results of the analysis:

Age: Selected

Sex: Not Selected

Race: Selected

Ethnicity: Selected

*Event of Interest Definitions*

Table below outlines definitions for each event of interest. For the definitions of the events of interest consisting of more than one term, at least one term must match. Please see Appendix B for the text representation of these definitions.


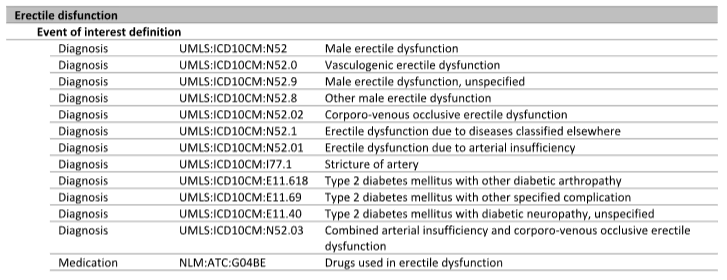


**Appendix B:** Propensity-score–matched comparative analysis for estimating the risk for different cardiovascular events among people with erectile dysfunction in the presence of type 2 diabetes and the absence of diabetes

# Methods

The analysis process includes two main steps: 1) Defining the cohorts through query criteria; 2) Setting up and running the analysis. Setting up the analysis requires definitions for the index event, outcomes criteria, and the time frame. Compare outcomes supports four analyses: Measures of Association, Survival, Number of Instances and Lab result distribution. These analyses have additional options that are listed in the Outcomes Definitions and Analyses Specifications section below. Furthermore, characteristics of the cohorts that are balanced using propensity score matching are also included in the Propensity Score Matching section.

## Cohorts definition

This section lists all terms used in the definitions of the two cohorts.

### Query Criteria for Cohort 1 (query name: ED with T2DM)

This query was run on the network Global Collaborative Network with 161 HCO(s) queried and 161 HCO(s) responded. A total of 83 provider(s) responded with patients. The final cohort included 312,644 patients who matched the query criteria listed in the table below. For the text representation of the query criteria please see Appendix A.

|  | | | | | |
| --- | --- | --- | --- | --- | --- |
| Ungrouped terms | | | | | |
|  | must have |  | demographics | Age | Age (between 20 and 80 years (most recent occurrence)) |
|  |  | and | demographics | UMLS:HL7V3.0:Gender:M | Male |
|  |  | and any of | diagnosis | UMLS:ICD10CM:N52 | Male erectile dysfunction |
|  |  |  | diagnosis | UMLS:ICD10CM:N52.0 | Vasculogenic erectile dysfunction |
|  |  | and any of | diagnosis | UMLS:ICD10CM:N52.9 | Male erectile dysfunction, unspecified |
|  |  |  | diagnosis | UMLS:ICD10CM:N52.8 | Other male erectile dysfunction |
|  |  |  | diagnosis | UMLS:ICD10CM:N52.02 | Corporo-venous occlusive erectile dysfunction |
|  |  |  | medication | NLM:ATC:G04BE | Drugs used in erectile dysfunction |
|  |  |  | diagnosis | UMLS:ICD10CM:N52.1 | Erectile dysfunction due to diseases classified elsewhere |
|  |  |  | diagnosis | UMLS:ICD10CM:N52.01 | Erectile dysfunction due to arterial insufficiency |
|  |  |  | diagnosis | UMLS:ICD10CM:N52.03 | Combined arterial insufficiency and corporo-venous occlusive erectile dysfunction |
|  |  | and | diagnosis | UMLS:ICD10CM:E11 | Type 2 diabetes mellitus |
|  | cannot have |  | diagnosis | UMLS:ICD10CM:E08 | Diabetes mellitus due to underlying condition |
|  |  | or | diagnosis | UMLS:ICD10CM:E09 | Drug or chemical induced diabetes mellitus |
|  |  | or | diagnosis | UMLS:ICD10CM:E10 | Type 1 diabetes mellitus |
|  |  | or | diagnosis | UMLS:ICD10CM:E13 | Other specified diabetes mellitus |

### Query Criteria for Cohort 2 (query name: ED without T2DM)

This query was run on the network Global Collaborative Network with 161 HCO(s) queried and 161 HCO(s) responded. A total of 150 provider(s) responded with patients. The final cohort included 1,459,702 patients who matched the query criteria listed in the table below.

| Ungrouped terms | | | | | |
| --- | --- | --- | --- | --- | --- |
|  | must have |  | demographics | Age | Age (between 20 and 80 years (most recent occurrence)) |
|  |  | and | demographics | UMLS:HL7V3.0:Gender:M | Male |
|  |  | and any of | diagnosis | UMLS:ICD10CM:N52 | Male erectile dysfunction |
|  |  |  | diagnosis | UMLS:ICD10CM:N52.0 | Vasculogenic erectile dysfunction |
|  |  |  | diagnosis | UMLS:ICD10CM:N52.9 | Male erectile dysfunction, unspecified |
|  |  |  | diagnosis | UMLS:ICD10CM:N52.8 | Other male erectile dysfunction |
|  |  |  | diagnosis | UMLS:ICD10CM:N52.02 | Corporo-venous occlusive erectile dysfunction |
|  |  |  | medication | NLM:ATC:G04BE | Drugs used in erectile dysfunction |
|  |  |  | diagnosis | UMLS:ICD10CM:N52.1 | Erectile dysfunction due to diseases classified elsewhere |
|  |  |  | diagnosis | UMLS:ICD10CM:N52.01 | Erectile dysfunction due to arterial insufficiency |
|  |  |  | diagnosis | UMLS:ICD10CM:N52.03 | Combined arterial insufficiency and corporo-venous occlusive erectile dysfunction |
|  | cannot have |  | diagnosis | UMLS:ICD10CM:E08-E13 | Diabetes mellitus |

## Analysis Setup

This section contains the Index Event and Time Window definitions and a list of selected outcomes and the analyses.

### Index Event & Time Window Definitions

The index event defines the point in time when each patient in the cohort enters the analysis. To define an index event for the cohort, one or more criteria for the cohort must be selected. The index date for each patient within a cohort is the day on which the patient first met the selected criteria for the cohort (listed in the table below).

As the index event defines the earliest time point after which outcomes are analyzed, the time window defines the duration during which outcomes are analyzed. The time window can start on the same day as the index event or at any specified time interval after the index event. The time window can end any time after the start date. Outcomes are defined as diagnoses, medications, procedures, or laboratory values that happened in the time window starting after the first occurrence of the index event.

### Time Window Used in this Analysis

This analysis included outcomes that occurred in the time window that started 1 day after the first occurrence of the index event. Since no end date was specified all outcomes after the first occurrence of the index event were included.

The index event only includes events that occurred up to 20 years ago. Patients whose index event occurred 20 years or more ago are excluded. In this analysis, 0 patients in Cohort 1 and 0 patients in Cohort 2 were excluded because they met the index event more than 20 years ago.

### Index Events Used in this Analysis

Index events for the Compare Outcomes analysis were derived from the cohort definitions. Index events were defined separately for each cohort and were based on the criteria used in the original cohort definition. Please see Appendix B for the text representation of the index event definition.

The index event for Cohort 1 (query name: ED with T2DM) was defined as the following:

|  | | | | | |
| --- | --- | --- | --- | --- | --- |
| Ungrouped terms | | | | | |
|  | must have | any of | diagnosis | UMLS:ICD10CM:N52 | Male erectile dysfunction |
|  |  |  | diagnosis | UMLS:ICD10CM:N52.0 | Vasculogenic erectile dysfunction |
|  |  | and any of | diagnosis | UMLS:ICD10CM:N52.9 | Male erectile dysfunction, unspecified |
|  |  |  | diagnosis | UMLS:ICD10CM:N52.8 | Other male erectile dysfunction |
|  |  |  | diagnosis | UMLS:ICD10CM:N52.02 | Corporo-venous occlusive erectile dysfunction |
|  |  |  | medication | NLM:ATC:G04BE | Drugs used in erectile dysfunction |
|  |  |  | diagnosis | UMLS:ICD10CM:N52.1 | Erectile dysfunction due to diseases classified elsewhere |
|  |  |  | diagnosis | UMLS:ICD10CM:N52.01 | Erectile dysfunction due to arterial insufficiency |
|  |  |  | diagnosis | UMLS:ICD10CM:N52.03 | Combined arterial insufficiency and corporo-venous occlusive erectile dysfunction |
|  |  | and | diagnosis | UMLS:ICD10CM:E11 | Type 2 diabetes mellitus |

The index event for Cohort 2 (query name: ED without T2DM) was defined as the following:

|  | | | | | |
| --- | --- | --- | --- | --- | --- |
| Ungrouped terms | | | | | |
|  | must have | any of | diagnosis | UMLS:ICD10CM:N52 | Male erectile dysfunction |
|  |  |  | diagnosis | UMLS:ICD10CM:N52.0 | Vasculogenic erectile dysfunction |
|  |  |  | diagnosis | UMLS:ICD10CM:N52.9 | Male erectile dysfunction, unspecified |
|  |  |  | diagnosis | UMLS:ICD10CM:N52.8 | Other male erectile dysfunction |
|  |  |  | diagnosis | UMLS:ICD10CM:N52.02 | Corporo-venous occlusive erectile dysfunction |
|  |  |  | medication | NLM:ATC:G04BE | Drugs used in erectile dysfunction |
|  |  |  | diagnosis | UMLS:ICD10CM:N52.1 | Erectile dysfunction due to diseases classified elsewhere |
|  |  |  | diagnosis | UMLS:ICD10CM:N52.01 | Erectile dysfunction due to arterial insufficiency |
|  |  |  | diagnosis | UMLS:ICD10CM:N52.03 | Combined arterial insufficiency and corporo-venous occlusive erectile dysfunction |

### Analyses Specifications

The Compare Outcomes Analytic supports four types of analyses: Measure of Association, Survival, Number of Instances, and Lab result distribution. The first three analyses support the “exclude patients with outcomes prior to the window” setting. This option can exclude patients from the analysis if they are not at risk for an outcome (e.g., if the outcome is a chronic disease). When "exclude patients with the outcome prior to the time window" is not checked, all patients in the cohort are included in the analysis, regardless of whether they had the outcome prior to the time window. When "exclude patients with the outcome prior to the time window" is checked, patients are excluded from the analysis if their record includes the outcome prior to the beginning of the time window. This selection will exclude all patients with the outcome prior to the index event. If the start of the time window for the analysis falls some days after the index event, patients will also be excluded if they have the outcome between the index event and the start of the time window.

### Measure of Association Analysis

The Measure of Association Analysis calculates and compares the fraction of patients with the selected outcome. The output summary includes: Patients in each Cohort (count of patients meeting query criteria); Patients with Outcome in each Cohort (of the patients in the cohort, count of patients that had the outcome in the time window); and Risk (the fraction of patients in the cohort that have the outcome in the time window, i.e. Patients with Outcome / Patients in Cohort). In addition, Risk Difference (the difference in the risks in Cohort 1 and Cohort 2), Risk Ratio (the ratio of the risks in Cohort 1 and Cohort 2), and Odds Ratio (the ratio of the odds in Cohort 1 and Cohort 2). The bar chart shows the risk of the outcome for the both cohorts.

### Survival Analysis

The Kaplan-Meier Analysis estimates probability of the outcome at a respective time interval (daily time interval is used in this analysis). In order to account for the patients who exited the cohort during the analysis period, and therefore should not be included in the analysis, censoring is applied. In this analysis, patients are removed from the analysis (censored) after the last fact in their record.

The output summary includes: Patients in each Cohort (count of patients meeting query criteria); Patients with Outcome (of the patients in the cohort, count of patients that had the outcome in the time window); Median Survival (the number of days when the survival drops below 50%; the “-” indicates that survival does not drop below 50% during the time window); and Survival Probability at End of Time Window (the % survival at the end of the time window). In addition, Log-Rank test, Hazard Ratio and test for Proportionality.

### Number of Instances Analysis

The Number of Instances Analysis calculates how many times the outcome occurred in the time window. This analysis includes two additional settings: include patients with zero instances; the definition of an instance.

Selecting to exclude patients with zero instances will remove these patients from the calculations for mean number of instances, standard deviation, or median. The histogram showing the distribution of patients by number of instances will not contain a bar for zero. Alternatively, by selecting to include patients with zero instances, the mean, standard deviation, and median for number of instances will reflect these patients. The histogram will contain a bar for zero patients.

The definition of an instance affects how counts are analyzed. By selecting Date, each calendar date on which any of the terms selected in the outcome are recorded will represent one instance. For example, if the outcome is “Med A or Med B,” and a patient has “Med A” on January 3, then both medications on January 4, then “Med B” on January 6, then that patient is considered to have three instances– January 3, January 4, and January 6. Note that if an outcome occurs across several dates (e.g. Visit: inpatient encounter), then only the start date is tracked for the purpose of counting instances. A patient who begins at stay on January 1, ends that stay on January 3, begins another stay on January 10, and ends that stay on January 15, is considered to have two instances of the outcome.

Selecting Visit as an Instance will count any visit that includes the outcome as one instance, regardless of how many times it occurred. For instance, consider a patient administered an analgesic on each of the three days that make up an inpatient stay following some index event. If analgesic is an outcome, these three administrations will represent only one instance, because all three are associated with the same visit.

The output summary includes: Patients in Cohort (count of patients meeting query criteria); Patients with Outcome (of the patients in the cohort, count of patients that had the outcome in the time window); Mean (mean of the counts); Standard Deviation (standard deviation of the counts); Median (median of the counts); and Median (1+ instances) when patients with zero instances included in the analysis. In addition, T-Test statistics testing for the difference between the cohorts is included.

### Laboratory Results Analysis

Lab Results can be included in the analysis only for the outcomes that are labs. Only the most recent lab values in the time window are included. For the lab results that are numeric, the outcome summary includes: Patients in Cohort (count of patients meeting query criteria); Patients with Outcome (of the patients in the cohort, count of patients that had the outcome in the time window); Mean (mean of the counts); and Standard Deviation (the standard deviation for lab values across patients in the cohort). In addition, T-Test statistics testing for the difference between the cohorts is included.

For the non-numeric lab results, three values are reported: counts of Negative; Positives; and Unknowns.

The counts are represented in the bar chart as percentages of the total counts.

### Outcome Definitions

Table below outlines the definitions for each outcome and the analysis specifications. For outcome definitions consisting of more than one term, at least one term must match. Please see Appendix C for the text representation of the outcome definitions.

| Ischemic hearth disease | | | | |
| --- | --- | --- | --- | --- |
|  | Outcome definition | | | |
|  | | Diagnosis | UMLS:ICD10CM:I20-I25 | Ischemic heart diseases |
|  | Settings for the performed analyses | | | |
|  | | Kaplan - Meier survival analysis | | excluding patients with outcome prior to the time window |
|  | | Risk analysis | | excluding patients with outcome prior to the time window |
|  | | Number of instances analysis | | excluding patients with outcome prior to the time window excluding patients with zero outcomes counts are grouped by date |
| Stroke | | | | |
|  | Outcome definition | | | |
|  | | Diagnosis | UMLS:ICD10CM:I60-I69 | Cerebrovascular diseases |
|  | Settings for the performed analyses | | | |
|  | | Risk analysis | | excluding patients with outcome prior to the time window |
|  | | Kaplan - Meier survival analysis | | excluding patients with outcome prior to the time window |
|  | | Number of instances analysis | | excluding patients with outcome prior to the time window excluding patients with zero outcomes counts are grouped by date |
| Heart failure | | | | |
|  | Outcome definition | | | |
|  | | Diagnosis | UMLS:ICD10CM:I50 | Heart failure |
|  | Settings for the performed analyses | | | |
|  | | Risk analysis | | excluding patients with outcome prior to the time window |
|  | | Kaplan - Meier survival analysis | | excluding patients with outcome prior to the time window |
|  | | Number of instances analysis | | excluding patients with outcome prior to the time window excluding patients with zero outcomes counts are grouped by date |
| Peripheral artery disease | | | | |
|  | Outcome definition | | | |
|  | | Diagnosis | UMLS:ICD10CM:I80-I89 | Diseases of veins, lymphatic vessels and lymph nodes, not elsewhere classified |
|  | Settings for the performed analyses | | | |
|  | | Risk analysis | | excluding patients with outcome prior to the time window |
|  | | Kaplan - Meier survival analysis | | excluding patients with outcome prior to the time window |
|  | | Number of instances analysis | | excluding patients with outcome prior to the time window excluding patients with zero outcomes counts are grouped by date |
| CVD | | | | |
|  | Outcome definition | | | |
|  | | Diagnosis | UMLS:ICD10CM:I20-I25 | Ischemic heart diseases |
|  | | Diagnosis | UMLS:ICD10CM:I60-I69 | Cerebrovascular diseases |
|  | | Diagnosis | UMLS:ICD10CM:I80-I89 | Diseases of veins, lymphatic vessels and lymph nodes, not elsewhere classified |
|  | | Diagnosis | UMLS:ICD10CM:I50 | Heart failure |
|  | Settings for the performed analyses | | | |
|  | | Risk analysis | | excluding patients with outcome prior to the time window |
|  | | Kaplan - Meier survival analysis | | excluding patients with outcome prior to the time window |
|  | | Number of instances analysis | | excluding patients with outcome prior to the time window excluding patients with zero outcomes counts are grouped by date |

## Propensity Score Matching

Propensity score matching was performed on 18 characteristic(s). In the Demographics category patients were matched on Age at Index characteristic(s). In the Diagnosis category patients were matched on Hypertensive diseases, Heart failure, Ischemic heart diseases, Cerebrovascular diseases, Diseases of arteries, arterioles and capillaries, Disorders of lipoprotein metabolism and other lipidemias, Chronic kidney disease (CKD), Nicotine dependence, unspecified, uncomplicated characteristic(s). In the Laboratory category patients were matched on Creatinine [Mass/volume] in Serum, Plasma or Blood, Cholesterol [Mass/volume] in Serum or Plasma, Cholesterol in LDL [Mass/volume] in Serum or Plasma, Cholesterol in HDL [Mass/volume] in Serum or Plasma, Triglyceride [Mass/volume] in Serum, Plasma or Blood, Hemoglobin A1c/Hemoglobin.total in Blood, BMI, Blood Pressure, Systolic, Blood Pressure, Diastolic characteristic(s). Characteristics of the cohorts before and after matching are summarized in the table below.

| Cohort 1 and cohort 2 patient count before and after propensity score matching | | | | | | | | | | | | |
| --- | --- | --- | --- | --- | --- | --- | --- | --- | --- | --- | --- | --- |
|  | | | Cohort | | | Patient count before matching | | | | Patient count after matching | | |
|  | | | 1 - ED with T2DM | | | 331,649 | | | | 312,545 | | |
|  | | | 2 - ED without T2DM | | | 1,579,633 | | | | 312,545 | | |
| Propensity score density function - Before and after matching (cohort 1 - purple, cohort 2 - green) | | | | | | | | | | | | |
|  |  | | 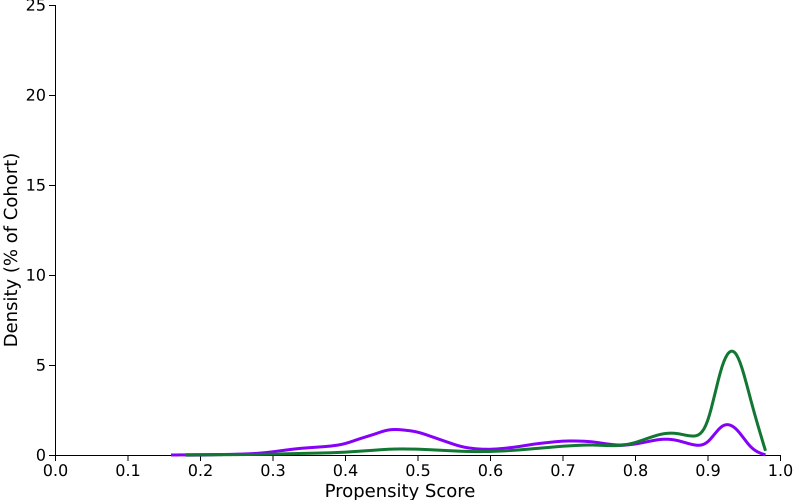 | | | | 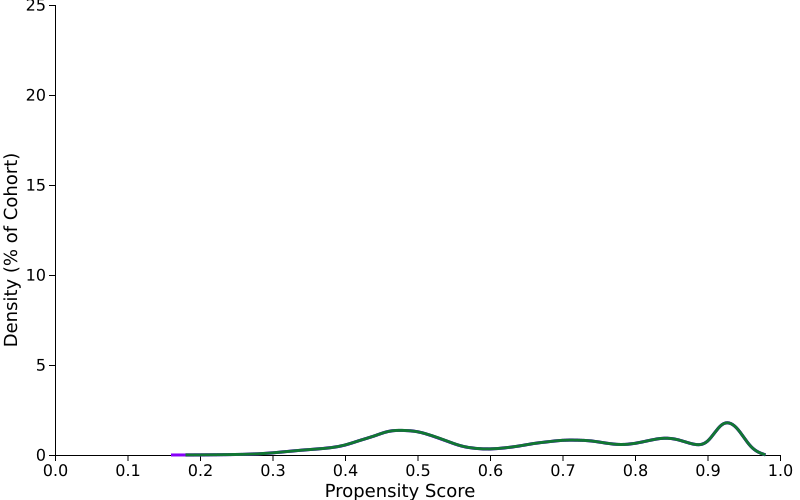 | | | | | |
| Cohort 1 (N = 331,649) and cohort 2 (N = 1,579,633) characteristics before propensity score matching | | | | | | | | | | | | |
|  | Demographics | | | | | | | | | | | |
|  |  | Cohort | | |  | Mean ± SD | | Patients | % of Cohort | | P-Value | Std diff. |
|  |  | 1 2 | | AI | Age at Index | 59.2 +/- 9.7 54.8 +/- 12.6 | | 330,120 1,561,521 | 100% 100% | | <0.001 | 0.395 |
|  | Diagnosis | | | | | | | | | | | |
|  |  | Cohort | | |  | Mean ± SD | | Patients | % of Cohort | | P-Value | Std diff. |
|  |  | 1 2 | | I10-I1A | Hypertensive diseases |  | | 224,758 447,998 | 68.1% 28.7% | | <0.001 | 0.858 |
|  |  | 1 2 | | I50 | Heart failure |  | | 27,479 41,632 | 8.3% 2.7% | | <0.001 | 0.250 |
|  |  | 1 2 | | I20-I25 | Ischemic heart diseases |  | | 71,403 149,539 | 21.6% 9.6% | | <0.001 | 0.337 |
|  |  | 1 2 | | I60-I69 | Cerebrovascular diseases |  | | 26,554 53,714 | 8.0% 3.4% | | <0.001 | 0.199 |
|  |  | 1 2 | | I70-I79 | Diseases of arteries, arterioles and capillaries |  | | 41,037 86,108 | 12.4% 5.5% | | <0.001 | 0.244 |
|  |  | 1 2 | | E78 | Disorders of lipoprotein metabolism and other lipidemias |  | | 211,948 429,807 | 64.2% 27.5% | | <0.001 | 0.792 |
|  |  | 1 2 | | N18 | Chronic kidney disease (CKD) |  | | 36,688 48,816 | 11.1% 3.1% | | <0.001 | 0.314 |
|  |  | 1 2 | | F17.200 | Nicotine dependence, unspecified, uncomplicated |  | | 41,768 103,886 | 12.7% 6.7% | | <0.001 | 0.204 |
|  | Procedure | | | | | | | | | | | |
|  |  | Cohort | | |  | Mean ± SD | | Patients | % of Cohort | | P-Value | Std diff. |
|  |  | 1 2 | | 1018513 | Smoking and tobacco use cessation counseling visit |  | | 5,069 9,755 | 1.5% 0.6% | | <0.001 | 0.088 |
|  | Laboratory | | | | | | | | | | | |
|  |  | Cohort | | |  | Mean ± SD | | Patients | % of Cohort | | P-Value | Std diff. |
|  |  | 1 2 | | 9024 | Creatinine [Mass/volume] in Serum, Plasma or Blood | 1.3 +/- 4.7 1.2 +/- 4.3 | | 256,699 822,643 | 77.8% 52.7% | | <0.001 | 0.011 |
|  |  | 1 2 | |  | 0 - 0 mg/dL |  | | 256,712 822,650 | 77.8% 52.7% | | <0.001 | 0.546 |
|  |  | 1 2 | | 9000 | Cholesterol [Mass/volume] in Serum or Plasma | 167.1 +/- 46.4 180.8 +/- 42.7 | | 213,096 577,231 | 64.6% 37.0% | | <0.001 | 0.307 |
|  |  | 1 2 | |  | 0 - 0 mg/dL |  | | 213,101 577,234 | 64.6% 37.0% | | <0.001 | 0.574 |
|  |  | 1 2 | | 9002 | Cholesterol in LDL [Mass/volume] in Serum or Plasma | 93.0 +/- 37.1 107.5 +/- 35.9 | | 211,929 575,517 | 64.2% 36.9% | | <0.001 | 0.396 |
|  |  | 1 2 | |  | 0 - 0 mg/dL |  | | 211,944 575,529 | 64.2% 36.9% | | <0.001 | 0.569 |
|  |  | 1 2 | | 9001 | Cholesterol in HDL [Mass/volume] in Serum or Plasma | 40.2 +/- 14.8 46.6 +/- 16.5 | | 214,268 579,912 | 64.9% 37.1% | | <0.001 | 0.411 |
|  |  | 1 2 | |  | 0 - 0 mg/dL |  | | 214,270 579,920 | 64.9% 37.1% | | <0.001 | 0.578 |
|  |  | 1 2 | | 9004 | Triglyceride [Mass/volume] in Serum, Plasma or Blood | 173.6 +/- 165.6 133.5 +/- 97.2 | | 212,708 576,165 | 64.4% 36.9% | | <0.001 | 0.296 |
|  |  | 1 2 | |  | 0 - 0 mg/dL |  | | 212,708 576,166 | 64.4% 36.9% | | <0.001 | 0.573 |
|  |  | 1 2 | | 9037 | Hemoglobin A1c/Hemoglobin.total in Blood | 7.1 +/- 1.8 5.6 +/- 0.8 | | 216,100 335,298 | 65.5% 21.5% | | <0.001 | 1.128 |
|  |  | 1 2 | |  | 0 - 0 % |  | | 216,104 335,323 | 65.5% 21.5% | | <0.001 | 0.990 |
|  |  | 1 2 | | 9083 | BMI | 32.8 +/- 6.6 29.3 +/- 5.6 | | 216,226 737,920 | 65.5% 47.3% | | <0.001 | 0.575 |
|  |  | 1 2 | |  | 0 - 0 kg/m2 |  | | 216,260 738,063 | 65.5% 47.3% | | <0.001 | 0.374 |
|  |  | 1 2 | | 9085 | Blood Pressure, Systolic | 132.4 +/- 18.1 129.2 +/- 17.2 | | 243,416 855,820 | 73.7% 54.8% | | <0.001 | 0.184 |
|  |  | 1 2 | |  | 0 - 0 mm[Hg] |  | | 243,418 855,834 | 73.7% 54.8% | | <0.001 | 0.403 |
|  |  | 1 2 | | 9086 | Blood Pressure, Diastolic | 78.6 +/- 11.3 78.4 +/- 11.2 | | 243,525 856,159 | 73.8% 54.8% | | <0.001 | 0.021 |
|  |  | 1 2 | |  | 0 - 0 mm[Hg] |  | | 243,528 856,187 | 73.8% 54.8% | | <0.001 | 0.403 |
|  |  | 1 2 | | 88031-0 | Smokeless tobacco status |  | | 0 0 | 0% 0% | | -- | -- |
|  |  | 1 2 | |  | 0 - 0 units |  | | 0 0 | 0% 0% | | -- | -- |
| Cohort 1 (N = 312,545) and cohort 2 (N = 312,545) characteristics after propensity score matching | | | | | | | | | | | | |
|  | Demographics | | | | | | | | | | | |
|  |  | Cohort | | |  | Mean ± SD | | Patients | % of Cohort | | P-Value | Std diff. |
|  |  | 1 2 | | AI | Age at Index | 58.9 +/- 9.7 59.2 +/- 9.9 | | 312,545 312,545 | 100% 100% | | <0.001 | 0.027 |
|  | Diagnosis | | | | | | | | | | | |
|  |  | Cohort | | |  | Mean ± SD | | Patients | % of Cohort | | P-Value | Std diff. |
|  |  | 1 2 | | I10-I1A | Hypertensive diseases |  | | 207,192 206,609 | 66.3% 66.1% | | 0.119 | 0.004 |
|  |  | 1 2 | | I50 | Heart failure |  | | 23,721 23,052 | 7.6% 7.4% | | 0.001 | 0.008 |
|  |  | 1 2 | | I20-I25 | Ischemic heart diseases |  | | 66,114 69,036 | 21.2% 22.1% | | <0.001 | 0.023 |
|  |  | 1 2 | | I60-I69 | Cerebrovascular diseases |  | | 24,629 25,656 | 7.9% 8.2% | | <0.001 | 0.012 |
|  |  | 1 2 | | I70-I79 | Diseases of arteries, arterioles and capillaries |  | | 37,444 37,429 | 12.0% 12.0% | | 0.953 | <0.001 |
|  |  | 1 2 | | E78 | Disorders of lipoprotein metabolism and other lipidemias |  | | 194,620 191,900 | 62.3% 61.4% | | <0.001 | 0.018 |
|  |  | 1 2 | | N18 | Chronic kidney disease (CKD) |  | | 30,036 27,526 | 9.6% 8.8% | | <0.001 | 0.028 |
|  |  | 1 2 | | F17.200 | Nicotine dependence, unspecified, uncomplicated |  | | 37,949 36,684 | 12.1% 11.7% | | <0.001 | 0.012 |
|  | Procedure | | | | | | | | | | | |
|  |  | Cohort | | |  | Mean ± SD | | Patients | % of Cohort | | P-Value | Std diff. |
|  |  | 1 2 | | 1018513 | Smoking and tobacco use cessation counseling visit |  | | 4,574 4,409 | 1.5% 1.4% | | 0.080 | 0.004 |
|  | Laboratory | | | | | | | | | | | |
|  |  | Cohort | | |  | Mean ± SD | | Patients | % of Cohort | | P-Value | Std diff. |
|  |  | 1 2 | | 9024 | Creatinine [Mass/volume] in Serum, Plasma or Blood | 1.3 +/- 4.8 1.3 +/- 4.6 | | 240,001 240,227 | 76.8% 76.9% | | 0.072 | 0.005 |
|  |  | 1 2 | |  | 0 - 0 mg/dL |  | | 240,014 240,228 | 76.8% 76.9% | | 0.521 | 0.002 |
|  |  | 1 2 | | 9000 | Cholesterol [Mass/volume] in Serum or Plasma | 167.6 +/- 46.5 176.7 +/- 43.3 | | 198,490 193,984 | 63.5% 62.1% | | <0.001 | 0.203 |
|  |  | 1 2 | |  | 0 - 0 mg/dL |  | | 198,495 193,984 | 63.5% 62.1% | | <0.001 | 0.030 |
|  |  | 1 2 | | 9002 | Cholesterol in LDL [Mass/volume] in Serum or Plasma | 93.5 +/- 37.2 103.1 +/- 36.8 | | 197,592 193,371 | 63.2% 61.9% | | <0.001 | 0.260 |
|  |  | 1 2 | |  | 0 - 0 mg/dL |  | | 197,607 193,374 | 63.2% 61.9% | | <0.001 | 0.028 |
|  |  | 1 2 | | 9001 | Cholesterol in HDL [Mass/volume] in Serum or Plasma | 40.2 +/- 14.7 45.5 +/- 16.7 | | 199,619 195,335 | 63.9% 62.5% | | <0.001 | 0.338 |
|  |  | 1 2 | |  | 0 - 0 mg/dL |  | | 199,621 195,337 | 63.9% 62.5% | | <0.001 | 0.028 |
|  |  | 1 2 | | 9004 | Triglyceride [Mass/volume] in Serum, Plasma or Blood | 174.0 +/- 166.0 138.6 +/- 101.5 | | 198,156 193,882 | 63.4% 62.0% | | <0.001 | 0.257 |
|  |  | 1 2 | |  | 0 - 0 mg/dL |  | | 198,156 193,882 | 63.4% 62.0% | | <0.001 | 0.028 |
|  |  | 1 2 | | 9037 | Hemoglobin A1c/Hemoglobin.total in Blood | 7.2 +/- 1.8 5.6 +/- 0.6 | | 198,526 199,242 | 63.5% 63.7% | | <0.001 | 1.187 |
|  |  | 1 2 | |  | 0 - 0 % |  | | 198,529 199,251 | 63.5% 63.8% | | 0.058 | 0.005 |
|  |  | 1 2 | | 9083 | BMI | 32.8 +/- 6.6 30.0 +/- 5.8 | | 201,990 202,197 | 64.6% 64.7% | | <0.001 | 0.458 |
|  |  | 1 2 | |  | 0 - 0 kg/m2 |  | | 202,023 202,218 | 64.6% 64.7% | | 0.606 | 0.001 |
|  |  | 1 2 | | 9085 | Blood Pressure, Systolic | 132.5 +/- 18.0 130.7 +/- 17.8 | | 227,817 227,535 | 72.9% 72.8% | | <0.001 | 0.100 |
|  |  | 1 2 | |  | 0 - 0 mm[Hg] |  | | 227,819 227,537 | 72.9% 72.8% | | 0.423 | 0.002 |
|  |  | 1 2 | | 9086 | Blood Pressure, Diastolic | 78.7 +/- 11.2 78.6 +/- 11.4 | | 227,926 227,588 | 72.9% 72.8% | | <0.001 | 0.013 |
|  |  | 1 2 | |  | 0 - 0 mm[Hg] |  | | 227,929 227,594 | 72.9% 72.8% | | 0.341 | 0.002 |
|  |  | 1 2 | | 88031-0 | Smokeless tobacco status |  | | 0 0 | 0% 0% | | -- | -- |
|  |  | 1 2 | |  | 0 - 0 units |  | | 0 0 | 0% 0% | | -- | -- |

# Results

Results are summarized in the tables below. Outcomes analysis was performed on the cohorts after propensity score matching.

| Follow-up Time (Before Matching) | | | | | | |
| --- | --- | --- | --- | --- | --- | --- |
|  |  | Cohort | Mean Follow-up (Days) | Standard Deviation | Median Follow-up (Days) | Interquartile Range |
|  |  | ED with T2DM | 1527.913 | 1318.283 | 1177 | 1788 |
|  |  | ED without T2DM | 1586.411 | 1463.342 | 1180 | 2052 |
| 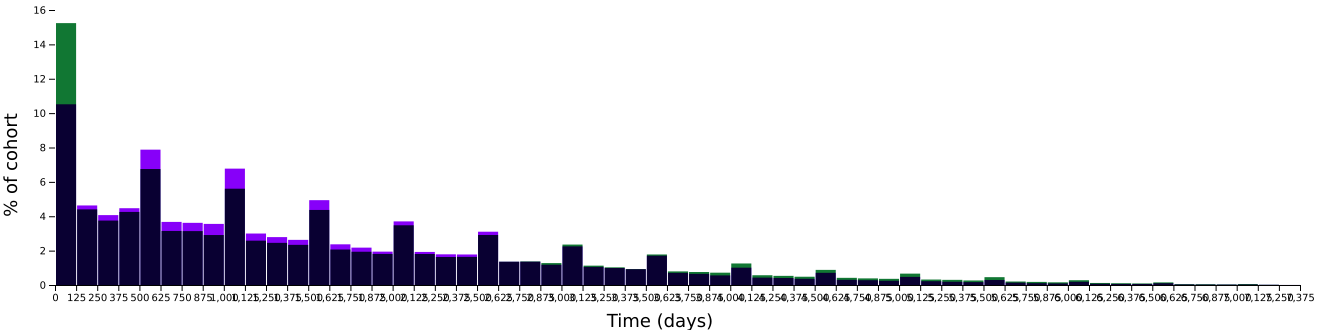 | | | | | | |
| 1,529 patients in Cohort 1 and 18,112 patients in Cohort 2 were excluded because they met the index event more than 20 years ago. | | | | | | |
|  | | | | | | |
| Follow-up Time (After Matching) | | | | | | |
|  |  | Cohort | Mean Follow-up (Days) | Standard Deviation | Median Follow-up (Days) | Interquartile Range |
|  |  | ED with T2DM | 1541.981 | 1327.086 | 1190 | 1808 |
|  |  | ED without T2DM | 1415.098 | 1287.523 | 1058 | 1715 |
| 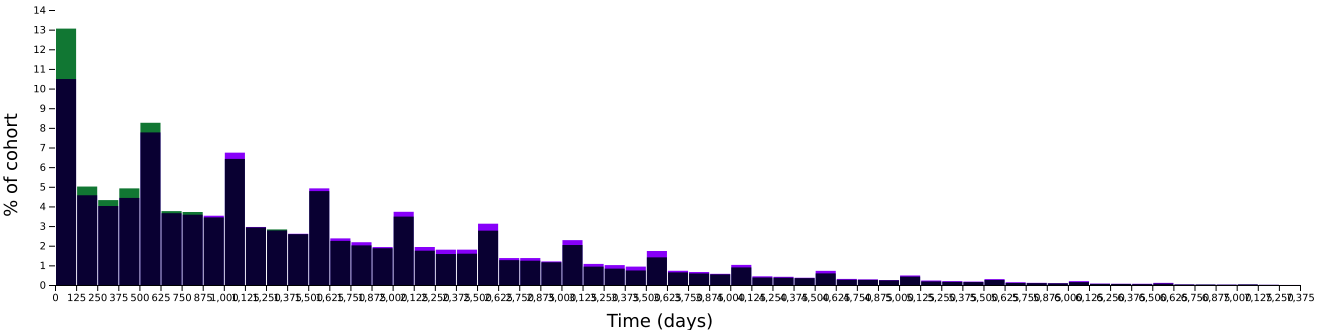 | | | | | | |

| 1 Ischemic hearth disease | | | | | | | | | | | | |
| --- | --- | --- | --- | --- | --- | --- | --- | --- | --- | --- | --- | --- |
|  | | Risk analysis excluding patients with outcome prior to the time window | | | | | | | | | | |
|  |  | | | Cohort | | | Patients in cohort | Patients with outcome | Risk | | | |
|  | | |  | 1 | | ED with T2DM | 238,185 | 37,506 | 0.157 | | | |
|  | | |  | 2 | | ED without T2DM | 241,115 | 27,784 | 0.115 | | | |
|  | | | | | | | | | | | | |
|  | | |  |  | | |  | 95% CI | z | p |  |  |
|  | | |  | Risk Difference | | | 0.042 | (0.040, 0.044) | 42.620 | 0 |  |  |
|  | | |  | Risk Ratio | | | 1.367 | (1.347, 1.386) | N/A | N/A |  |  |
|  | | |  | Odds Ratio | | | 1.435 | (1.411, 1.459) | N/A | N/A |  |  |
|  | | | | | | | | | | | | |
|  | |  | | | 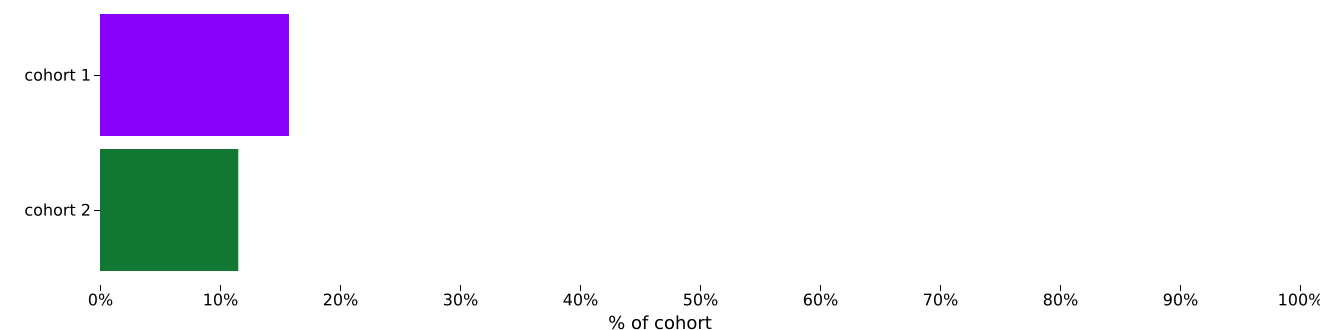 | | | | | | | |
|  | |  | | | 74,360 patients in Cohort 1 and 71,430 patients in Cohort 2 were excluded from results because they had the outcome prior to the time window. | | | | | | | |
|  | | Kaplan - Meier survival analysis excluding patients with outcome prior to the time window | | | | | | | | | | |
|  | | |  | Cohort | | | Patients in cohort | Patients with outcome | Median survival (days) | Survival probability at end of time window | | |
|  | | |  | 1 | | ED with T2DM | 238,185 | 37,506 | 5911 | 40.38% | | |
|  | | |  | 2 | | ED without T2DM | 241,115 | 27,784 | 6842 | 47.74% | | |
|  | | | | | | | | | | | | |
|  | | |  |  | | | χ^2^ | df | p |  |  |  |
|  | | |  | Log-Rank Test | | | 1057.189 | 1 | 0.000 |  |  |  |
|  | | | | | | | | | | | | |
|  | | |  |  | | | Hazard Ratio | 95% CI | χ^2^ | df | p | |
|  | | |  | Hazard Ratio and Proportionality | | | 1.293 | (1.273, 1.313) | 0.753 | 1 | 0.385 | |
|  | | | | | | | | | | | | |
|  | |  | | | 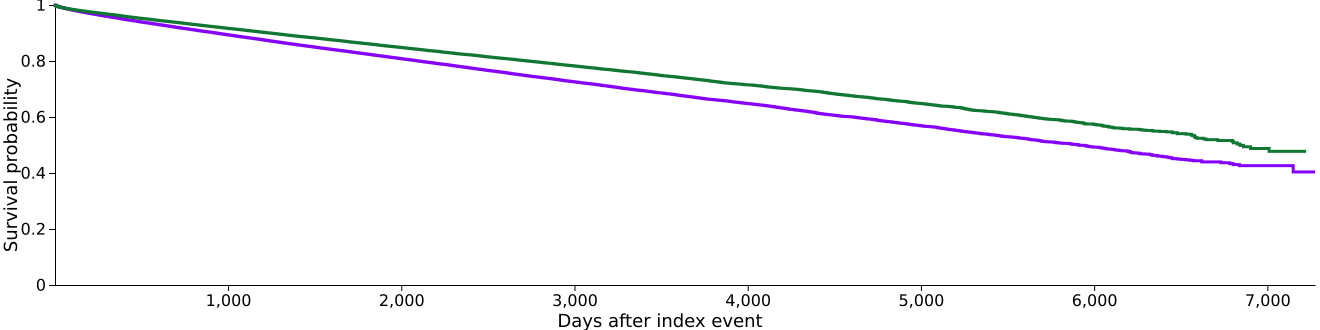 | | | | | | | |
|  | |  | | | 74,360 patients in Cohort 1 and 71,430 patients in Cohort 2 were excluded from results because they had the outcome prior to the time window. | | | | | | | |
|  | | Number of instances excluding patients with outcome prior to the time window | | | | | | | | | | |
|  | | |  | Cohort | | | Patients in cohort | Patients with outcome | Mean | Standard Deviation | Median | |
|  | | |  | 1 | | ED with T2DM | 238,185 | 37,506 | 7.896 | 14.527 | 3 | |
|  | | |  | 2 | | ED without T2DM | 241,115 | 27,784 | 6.128 | 11.018 | 2 | |
|  | | | | | | | | | | | | |
|  | | |  |  | | | t | df | p |  |  |  |
|  | | |  | Test Statistics | | | 16.984 | 65288 | 0.000 |  |  |  |
|  | | | | | | | | | | | | |
|  | |  | | | 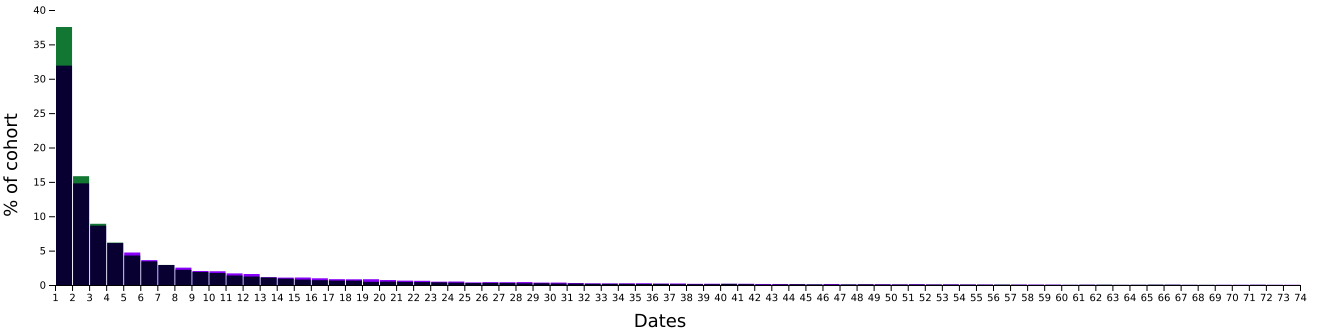 | | | | | | | |
|  | |  | | | 239 data points for Cohort 1 and 103 data points for Cohort 2 were omitted for display purposes. 74,360 patients in Cohort 1 and 71,430 patients in Cohort 2 were excluded from results because they had the outcome prior to the time window. | | | | | | | |
| 2 Stroke | | | | | | | | | | | | |
|  | | Risk analysis excluding patients with outcome prior to the time window | | | | | | | | | | |
|  |  | | | Cohort | | | Patients in cohort | Patients with outcome | Risk | | | |
|  | | |  | 1 | | ED with T2DM | 285,311 | 22,450 | 0.079 | | | |
|  | | |  | 2 | | ED without T2DM | 285,837 | 15,895 | 0.056 | | | |
|  | | | | | | | | | | | | |
|  | | |  |  | | |  | 95% CI | z | p |  |  |
|  | | |  | Risk Difference | | | 0.023 | (0.022, 0.024) | 34.845 | 0.000 |  |  |
|  | | |  | Risk Ratio | | | 1.415 | (1.387, 1.443) | N/A | N/A |  |  |
|  | | |  | Odds Ratio | | | 1.450 | (1.420, 1.481) | N/A | N/A |  |  |
|  | | | | | | | | | | | | |
|  | |  | | | 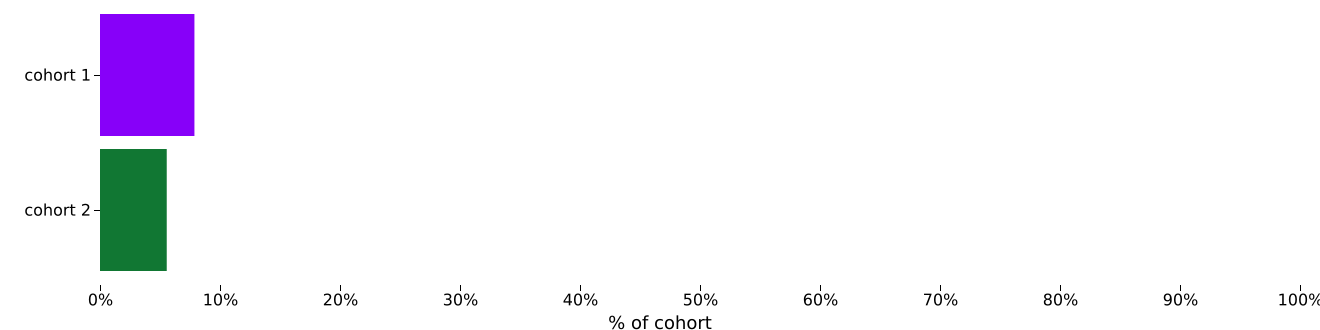 | | | | | | | |
|  | |  | | | 27,234 patients in Cohort 1 and 26,708 patients in Cohort 2 were excluded from results because they had the outcome prior to the time window. | | | | | | | |
|  | | Kaplan - Meier survival analysis excluding patients with outcome prior to the time window | | | | | | | | | | |
|  | | |  | Cohort | | | Patients in cohort | Patients with outcome | Median survival (days) | Survival probability at end of time window | | |
|  | | |  | 1 | | ED with T2DM | 285,311 | 22,450 | -- | 60.82% | | |
|  | | |  | 2 | | ED without T2DM | 285,837 | 15,895 | -- | 66.70% | | |
|  | | | | | | | | | | | | |
|  | | |  |  | | | χ^2^ | df | p |  |  |  |
|  | | |  | Log-Rank Test | | | 699.087 | 1 | 0.000 |  |  |  |
|  | | | | | | | | | | | | |
|  | | |  |  | | | Hazard Ratio | 95% CI | χ^2^ | df | p | |
|  | | |  | Hazard Ratio and Proportionality | | | 1.314 | (1.288, 1.341) | 10.877 | 1 | 0.001 | |
|  | | | | | | | | | | | | |
|  | |  | | | 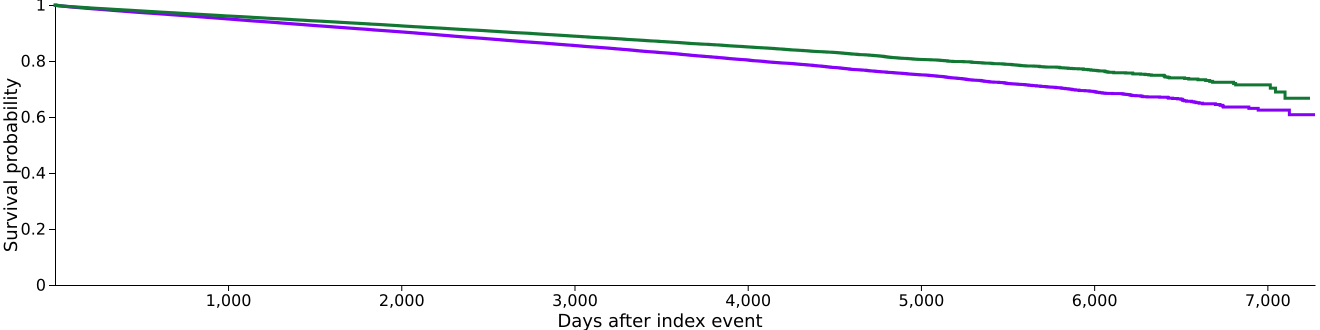 | | | | | | | |
|  | |  | | | 27,234 patients in Cohort 1 and 26,708 patients in Cohort 2 were excluded from results because they had the outcome prior to the time window. | | | | | | | |
|  | | Number of instances excluding patients with outcome prior to the time window | | | | | | | | | | |
|  | | |  | Cohort | | | Patients in cohort | Patients with outcome | Mean | Standard Deviation | Median | |
|  | | |  | 1 | | ED with T2DM | 285,311 | 22,450 | 5.667 | 11.526 | 2 | |
|  | | |  | 2 | | ED without T2DM | 285,837 | 15,895 | 4.878 | 10.219 | 2 | |
|  | | | | | | | | | | | | |
|  | | |  |  | | | t | df | p |  |  |  |
|  | | |  | Test Statistics | | | 6.921 | 38343 | 0.000 |  |  |  |
|  | | | | | | | | | | | | |
|  | |  | | | 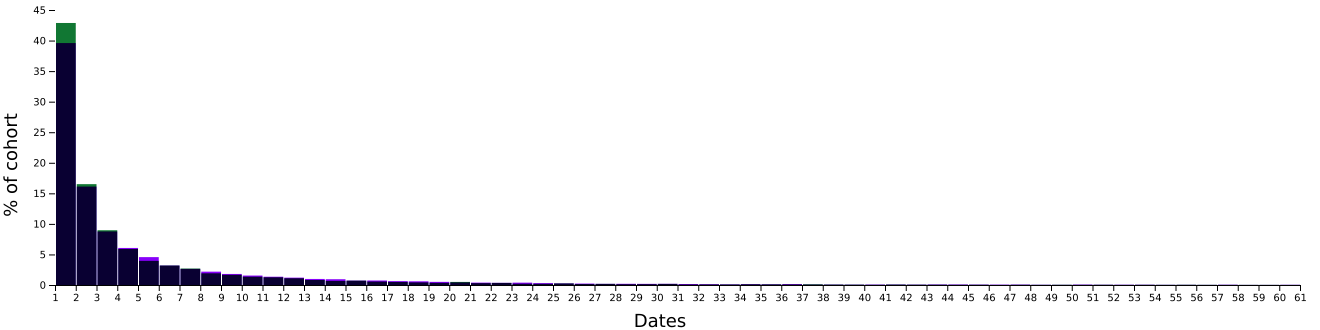 | | | | | | | |
|  | |  | | | 154 data points for Cohort 1 and 64 data points for Cohort 2 were omitted for display purposes. 27,234 patients in Cohort 1 and 26,708 patients in Cohort 2 were excluded from results because they had the outcome prior to the time window. | | | | | | | |
| 3 Heart failure | | | | | | | | | | | | |
|  | | Risk analysis excluding patients with outcome prior to the time window | | | | | | | | | | |
|  |  | | | Cohort | | | Patients in cohort | Patients with outcome | Risk | | | |
|  | | |  | 1 | | ED with T2DM | 285,408 | 23,842 | 0.084 | | | |
|  | | |  | 2 | | ED without T2DM | 287,971 | 14,069 | 0.049 | | | |
|  | | | | | | | | | | | | |
|  | | |  |  | | |  | 95% CI | z | p |  |  |
|  | | |  | Risk Difference | | | 0.035 | (0.033, 0.036) | 52.841 | 0 |  |  |
|  | | |  | Risk Ratio | | | 1.710 | (1.676, 1.745) | N/A | N/A |  |  |
|  | | |  | Odds Ratio | | | 1.775 | (1.737, 1.813) | N/A | N/A |  |  |
|  | | | | | | | | | | | | |
|  | |  | | | 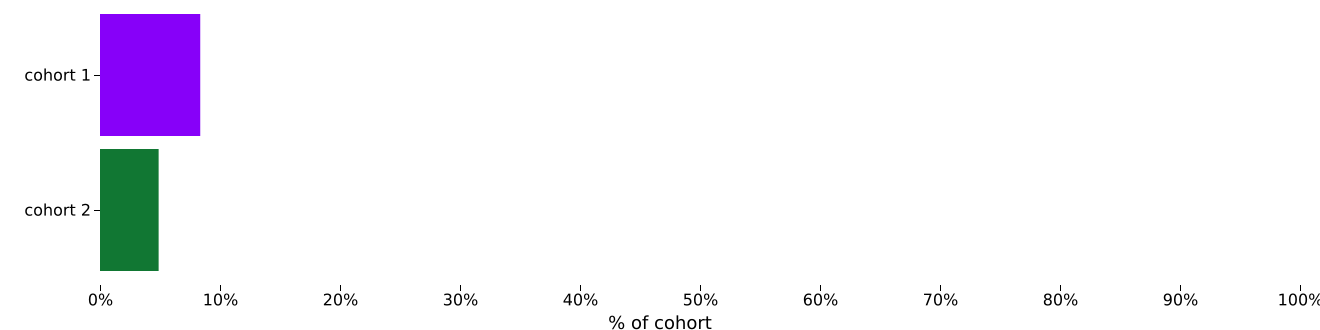 | | | | | | | |
|  | |  | | | 27,137 patients in Cohort 1 and 24,574 patients in Cohort 2 were excluded from results because they had the outcome prior to the time window. | | | | | | | |
|  | | Kaplan - Meier survival analysis excluding patients with outcome prior to the time window | | | | | | | | | | |
|  | | |  | Cohort | | | Patients in cohort | Patients with outcome | Median survival (days) | Survival probability at end of time window | | |
|  | | |  | 1 | | ED with T2DM | 285,408 | 23,842 | -- | 58.30% | | |
|  | | |  | 2 | | ED without T2DM | 287,971 | 14,069 | -- | 70.75% | | |
|  | | | | | | | | | | | | |
|  | | |  |  | | | χ^2^ | df | p |  |  |  |
|  | | |  | Log-Rank Test | | | 1985.220 | 1 | 0 |  |  |  |
|  | | | | | | | | | | | | |
|  | | |  |  | | | Hazard Ratio | 95% CI | χ^2^ | df | p | |
|  | | |  | Hazard Ratio and Proportionality | | | 1.599 | (1.566, 1.633) | 42.686 | 1 | 0.000 | |
|  | | | | | | | | | | | | |
|  | |  | | | 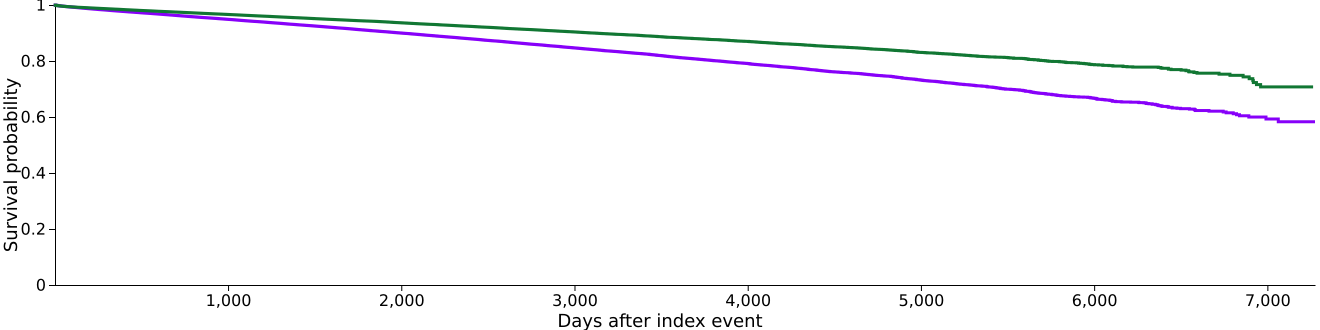 | | | | | | | |
|  | |  | | | 27,137 patients in Cohort 1 and 24,574 patients in Cohort 2 were excluded from results because they had the outcome prior to the time window. | | | | | | | |
|  | | Number of instances excluding patients with outcome prior to the time window | | | | | | | | | | |
|  | | |  | Cohort | | | Patients in cohort | Patients with outcome | Mean | Standard Deviation | Median | |
|  | | |  | 1 | | ED with T2DM | 285,408 | 23,842 | 9.756 | 19.006 | 3 | |
|  | | |  | 2 | | ED without T2DM | 287,971 | 14,069 | 7.040 | 12.338 | 3 | |
|  | | | | | | | | | | | | |
|  | | |  |  | | | t | df | p |  |  |  |
|  | | |  | Test Statistics | | | 15.168 | 37909 | 0.000 |  |  |  |
|  | | | | | | | | | | | | |
|  | |  | | | 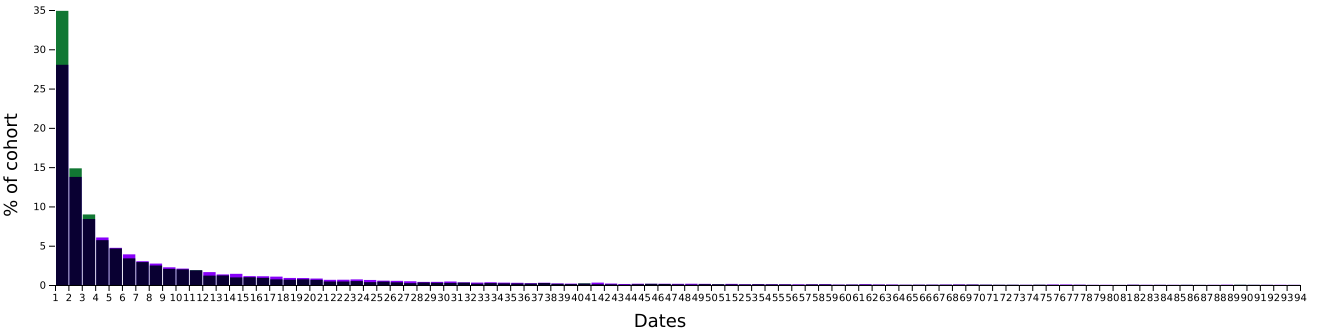 | | | | | | | |
|  | |  | | | 167 data points for Cohort 1 and 45 data points for Cohort 2 were omitted for display purposes. 27,137 patients in Cohort 1 and 24,574 patients in Cohort 2 were excluded from results because they had the outcome prior to the time window. | | | | | | | |
| 4 Peripheral artery disease | | | | | | | | | | | | |
|  | | Risk analysis excluding patients with outcome prior to the time window | | | | | | | | | | |
|  |  | | | Cohort | | | Patients in cohort | Patients with outcome | Risk | | | |
|  | | |  | 1 | | ED with T2DM | 280,898 | 23,016 | 0.082 | | | |
|  | | |  | 2 | | ED without T2DM | 286,968 | 17,473 | 0.061 | | | |
|  | | | | | | | | | | | | |
|  | | |  |  | | |  | 95% CI | z | p |  |  |
|  | | |  | Risk Difference | | | 0.021 | (0.020, 0.022) | 30.819 | 0.000 |  |  |
|  | | |  | Risk Ratio | | | 1.346 | (1.320, 1.371) | N/A | N/A |  |  |
|  | | |  | Odds Ratio | | | 1.377 | (1.349, 1.405) | N/A | N/A |  |  |
|  | | | | | | | | | | | | |
|  | |  | | | 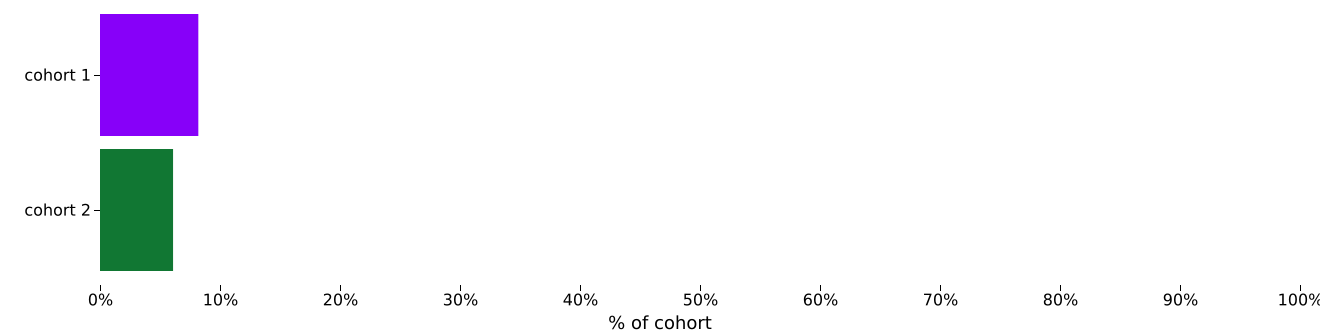 | | | | | | | |
|  | |  | | | 31,647 patients in Cohort 1 and 25,577 patients in Cohort 2 were excluded from results because they had the outcome prior to the time window. | | | | | | | |
|  | | Kaplan - Meier survival analysis excluding patients with outcome prior to the time window | | | | | | | | | | |
|  | | |  | Cohort | | | Patients in cohort | Patients with outcome | Median survival (days) | Survival probability at end of time window | | |
|  | | |  | 1 | | ED with T2DM | 280,898 | 23,016 | -- | 67.00% | | |
|  | | |  | 2 | | ED without T2DM | 286,968 | 17,473 | -- | 71.75% | | |
|  | | | | | | | | | | | | |
|  | | |  |  | | | χ^2^ | df | p |  |  |  |
|  | | |  | Log-Rank Test | | | 491.828 | 1 | 0.000 |  |  |  |
|  | | | | | | | | | | | | |
|  | | |  |  | | | Hazard Ratio | 95% CI | χ^2^ | df | p | |
|  | | |  | Hazard Ratio and Proportionality | | | 1.249 | (1.224, 1.274) | 15.526 | 1 | 0.000 | |
|  | | | | | | | | | | | | |
|  | |  | | | 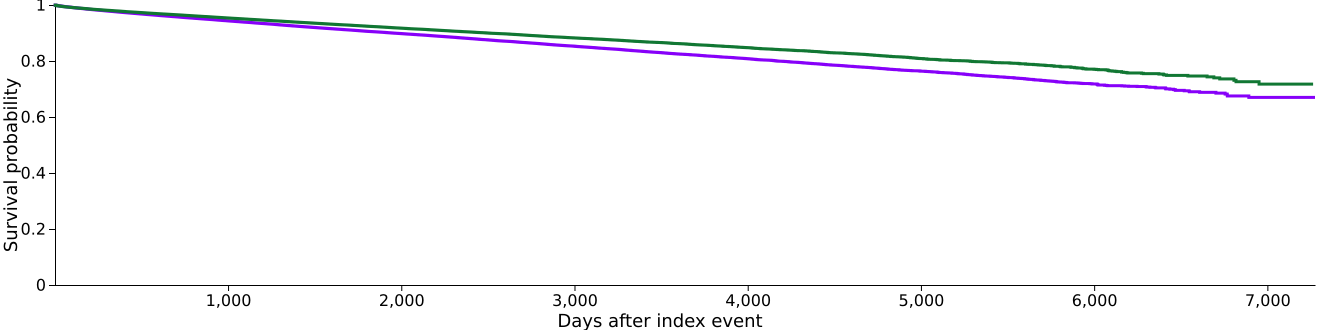 | | | | | | | |
|  | |  | | | 31,647 patients in Cohort 1 and 25,577 patients in Cohort 2 were excluded from results because they had the outcome prior to the time window. | | | | | | | |
|  | | Number of instances excluding patients with outcome prior to the time window | | | | | | | | | | |
|  | | |  | Cohort | | | Patients in cohort | Patients with outcome | Mean | Standard Deviation | Median | |
|  | | |  | 1 | | ED with T2DM | 280,898 | 23,016 | 4.701 | 12.596 | 2 | |
|  | | |  | 2 | | ED without T2DM | 286,968 | 17,473 | 4.219 | 9.795 | 2 | |
|  | | | | | | | | | | | | |
|  | | |  |  | | | t | df | p |  |  |  |
|  | | |  | Test Statistics | | | 4.189 | 40487 | 0.000 |  |  |  |
|  | | | | | | | | | | | | |
|  | |  | | | 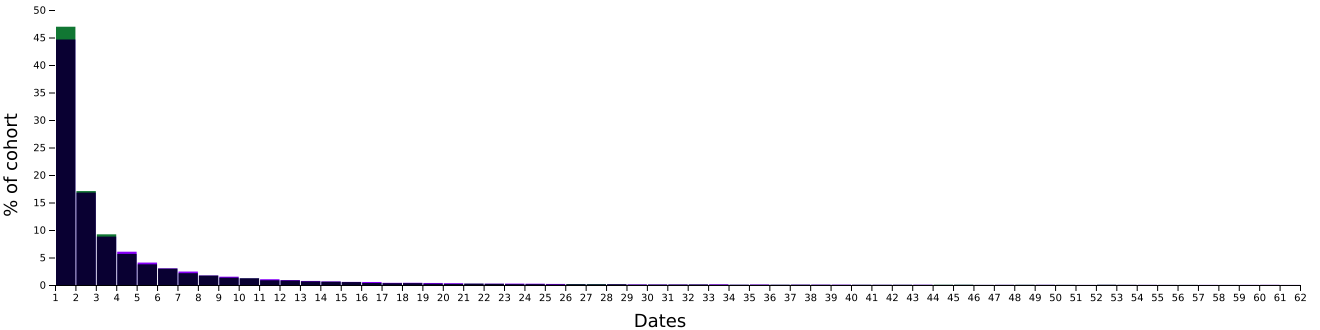 | | | | | | | |
|  | |  | | | 121 data points for Cohort 1 and 82 data points for Cohort 2 were omitted for display purposes. 31,647 patients in Cohort 1 and 25,577 patients in Cohort 2 were excluded from results because they had the outcome prior to the time window. | | | | | | | |
| 5 CVD | | | | | | | | | | | | |
|  | | Risk analysis excluding patients with outcome prior to the time window | | | | | | | | | | |
|  |  | | | Cohort | | | Patients in cohort | Patients with outcome | Risk | | | |
|  | | |  | 1 | | ED with T2DM | 201,440 | 48,769 | 0.242 | | | |
|  | | |  | 2 | | ED without T2DM | 207,930 | 38,055 | 0.183 | | | |
|  | | | | | | | | | | | | |
|  | | |  |  | | |  | 95% CI | z | p |  |  |
|  | | |  | Risk Difference | | | 0.059 | (0.057, 0.062) | 46.232 | 0 |  |  |
|  | | |  | Risk Ratio | | | 1.323 | (1.307, 1.339) | N/A | N/A |  |  |
|  | | |  | Odds Ratio | | | 1.426 | (1.405, 1.448) | N/A | N/A |  |  |
|  | | | | | | | | | | | | |
|  | |  | | | 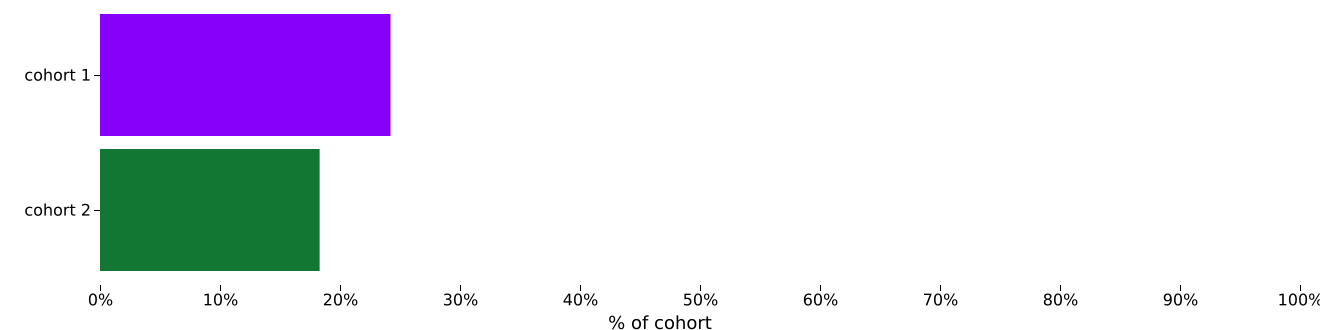 | | | | | | | |
|  | |  | | | 111,105 patients in Cohort 1 and 104,615 patients in Cohort 2 were excluded from results because they had the outcome prior to the time window. | | | | | | | |
|  | | Kaplan - Meier survival analysis excluding patients with outcome prior to the time window | | | | | | | | | | |
|  | | |  | Cohort | | | Patients in cohort | Patients with outcome | Median survival (days) | Survival probability at end of time window | | |
|  | | |  | 1 | | ED with T2DM | 201,440 | 48,769 | 3977 | 22.05% | | |
|  | | |  | 2 | | ED without T2DM | 207,930 | 38,055 | 4917 | 30.80% | | |
|  | | | | | | | | | | | | |
|  | | |  |  | | | χ^2^ | df | p |  |  |  |
|  | | |  | Log-Rank Test | | | 1166.514 | 1 | 0.000 |  |  |  |
|  | | | | | | | | | | | | |
|  | | |  |  | | | Hazard Ratio | 95% CI | χ^2^ | df | p | |
|  | | |  | Hazard Ratio and Proportionality | | | 1.262 | (1.246, 1.280) | 4.541 | 1 | 0.033 | |
|  | | | | | | | | | | | | |
|  | |  | | | 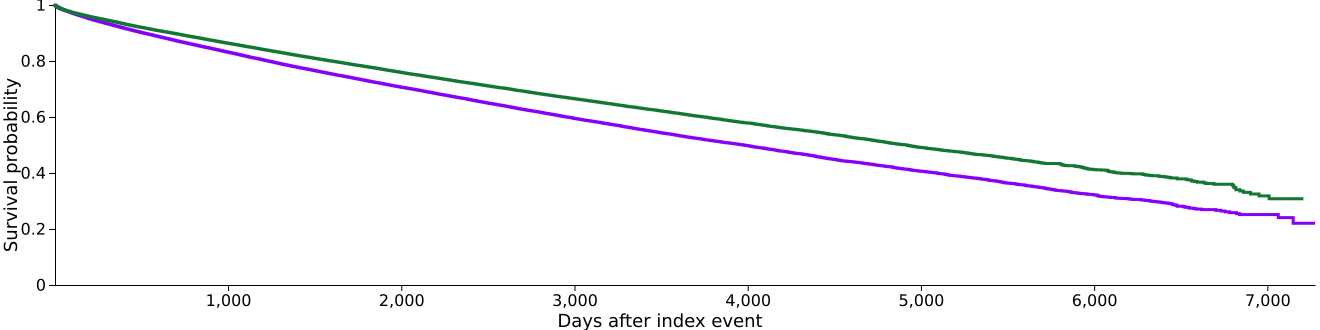 | | | | | | | |
|  | |  | | | 111,105 patients in Cohort 1 and 104,615 patients in Cohort 2 were excluded from results because they had the outcome prior to the time window. | | | | | | | |
|  | | Number of instances excluding patients with outcome prior to the time window | | | | | | | | | | |
|  | | |  | Cohort | | | Patients in cohort | Patients with outcome | Mean | Standard Deviation | Median | |
|  | | |  | 1 | | ED with T2DM | 201,440 | 48,769 | 9.428 | 18.244 | 3 | |
|  | | |  | 2 | | ED without T2DM | 207,930 | 38,055 | 6.949 | 12.934 | 3 | |
|  | | | | | | | | | | | | |
|  | | |  |  | | | t | df | p |  |  |  |
|  | | |  | Test Statistics | | | 22.469 | 86822 | 0.000 |  |  |  |
|  | | | | | | | | | | | | |
|  | |  | | | 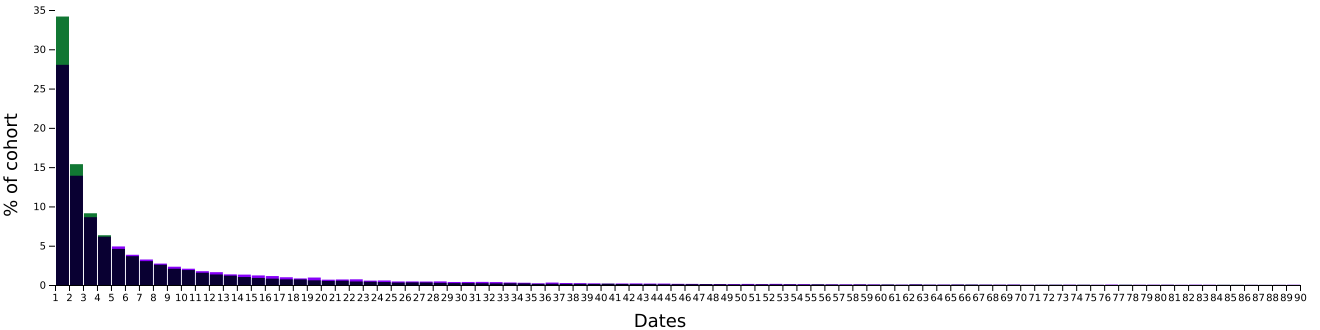 | | | | | | | |
|  | |  | | | 343 data points for Cohort 1 and 133 data points for Cohort 2 were omitted for display purposes. 111,105 patients in Cohort 1 and 104,615 patients in Cohort 2 were excluded from results because they had the outcome prior to the time window. | | | | | | | |

# Appendix A – Text Representation of the Cohorts Definition

This section lists all terms used in the definitions of the two cohorts.

### Query Criteria for Cohort 1 (query name: ED with T2DM)

Patients must have:
 all of the following:
 Age (Age) (between 20 and 80 years (most recent occurrence)); and
 Male (UMLS:HL7V3.0:Gender:M); and
 any of the following:
 Male erectile dysfunction (UMLS:ICD10CM:N52); or
 Vasculogenic erectile dysfunction (UMLS:ICD10CM:N52.0); and
 any of the following:
 Male erectile dysfunction, unspecified (UMLS:ICD10CM:N52.9); or
 Other male erectile dysfunction (UMLS:ICD10CM:N52.8); or
 Corporo-venous occlusive erectile dysfunction (UMLS:ICD10CM:N52.02); or
 Drugs used in erectile dysfunction (NLM:ATC:G04BE); or
 Erectile dysfunction due to diseases classified elsewhere (UMLS:ICD10CM:N52.1); or
 Erectile dysfunction due to arterial insufficiency (UMLS:ICD10CM:N52.01); or
 Combined arterial insufficiency and corporo-venous occlusive erectile dysfunction (UMLS:ICD10CM:N52.03); and
 Type 2 diabetes mellitus (UMLS:ICD10CM:E11).

Patients cannot have:
 any of the following:
 Diabetes mellitus due to underlying condition (UMLS:ICD10CM:E08); or
 Drug or chemical induced diabetes mellitus (UMLS:ICD10CM:E09); or
 Type 1 diabetes mellitus (UMLS:ICD10CM:E10); or
 Other specified diabetes mellitus (UMLS:ICD10CM:E13).

### Query Criteria for Cohort 2 (query name: ED without T2DM)

Patients must have:
 all of the following:
 Age (Age) (between 20 and 80 years (most recent occurrence)); and
 Male (UMLS:HL7V3.0:Gender:M); and
 any of the following:
 Male erectile dysfunction (UMLS:ICD10CM:N52); or
 Vasculogenic erectile dysfunction (UMLS:ICD10CM:N52.0); or
 Male erectile dysfunction, unspecified (UMLS:ICD10CM:N52.9); or
 Other male erectile dysfunction (UMLS:ICD10CM:N52.8); or
 Corporo-venous occlusive erectile dysfunction (UMLS:ICD10CM:N52.02); or
 Drugs used in erectile dysfunction (NLM:ATC:G04BE); or
 Erectile dysfunction due to diseases classified elsewhere (UMLS:ICD10CM:N52.1); or
 Erectile dysfunction due to arterial insufficiency (UMLS:ICD10CM:N52.01); or
 Combined arterial insufficiency and corporo-venous occlusive erectile dysfunction (UMLS:ICD10CM:N52.03).

Patients cannot have:
 Diabetes mellitus (UMLS:ICD10CM:E08-E13).

# Appendix B – Text Representation of the Analysis Setup

This section contains the Index Event definition for each cohort.

The index event for Cohort 1 (query name: ED with T2DM) is defined as the following:

Patients must have:
 all of the following:
 any of the following:
 Male erectile dysfunction (UMLS:ICD10CM:N52); or
 Vasculogenic erectile dysfunction (UMLS:ICD10CM:N52.0); and
 any of the following:
 Male erectile dysfunction, unspecified (UMLS:ICD10CM:N52.9); or
 Other male erectile dysfunction (UMLS:ICD10CM:N52.8); or
 Corporo-venous occlusive erectile dysfunction (UMLS:ICD10CM:N52.02); or
 Drugs used in erectile dysfunction (NLM:ATC:G04BE); or
 Erectile dysfunction due to diseases classified elsewhere (UMLS:ICD10CM:N52.1); or
 Erectile dysfunction due to arterial insufficiency (UMLS:ICD10CM:N52.01); or
 Combined arterial insufficiency and corporo-venous occlusive erectile dysfunction (UMLS:ICD10CM:N52.03); and
 Type 2 diabetes mellitus (UMLS:ICD10CM:E11).

The index event for Cohort 2 (query name: ED without T2DM) is defined as the following:

Patients must have:
 any of the following:
 Male erectile dysfunction (UMLS:ICD10CM:N52); or
 Vasculogenic erectile dysfunction (UMLS:ICD10CM:N52.0); or
 Male erectile dysfunction, unspecified (UMLS:ICD10CM:N52.9); or
 Other male erectile dysfunction (UMLS:ICD10CM:N52.8); or
 Corporo-venous occlusive erectile dysfunction (UMLS:ICD10CM:N52.02); or
 Drugs used in erectile dysfunction (NLM:ATC:G04BE); or
 Erectile dysfunction due to diseases classified elsewhere (UMLS:ICD10CM:N52.1); or
 Erectile dysfunction due to arterial insufficiency (UMLS:ICD10CM:N52.01); or
 Combined arterial insufficiency and corporo-venous occlusive erectile dysfunction (UMLS:ICD10CM:N52.03).

# Appendix C – Text Representation of the Outcomes Definition

This analysis includes the following outcomes:

Ischemic hearth disease
 Patients must have:
 Ischemic heart diseases (UMLS:ICD10CM:I20-I25).

Stroke
 Patients must have:
 Cerebrovascular diseases (UMLS:ICD10CM:I60-I69).

Heart failure
 Patients must have:
 Heart failure (UMLS:ICD10CM:I50).

Peripheral artery disease
 Patients must have:
 Diseases of veins, lymphatic vessels and lymph nodes, not elsewhere classified (UMLS:ICD10CM:I80-I89).

CVD
 Patients must have:
 any of the following:
 Ischemic heart diseases (UMLS:ICD10CM:I20-I25); or
 Cerebrovascular diseases (UMLS:ICD10CM:I60-I69); or
 Diseases of veins, lymphatic vessels and lymph nodes, not elsewhere classified (UMLS:ICD10CM:I80-I89); or
 Heart failure (UMLS:ICD10CM:I50).

**Appendix C:** Propensity-score–matched comparative analysis for estimating the risk for different cardiovascular events among people with type 2 diabetes regarding the presence or absence of erectile dysfunction

# Methods

The analysis process includes two main steps: 1) Defining the cohorts through query criteria; 2) Setting up and running the analysis. Setting up the analysis requires definitions for the index event, outcomes criteria, and the time frame. Compare outcomes supports four analyses: Measures of Association, Survival, Number of Instances and Lab result distribution. These analyses have additional options that are listed in the Outcomes Definitions and Analyses Specifications section below. Furthermore, characteristics of the cohorts that are balanced using propensity score matching are also included in the Propensity Score Matching section.

## Cohorts definition

This section lists all terms used in the definitions of the two cohorts.

### Query Criteria for Cohort 1 (query name: T2DM with ED)

This query was run on the network Global Collaborative Network with 166 HCO(s) queried and 166 HCO(s) responded. A total of 147 provider(s) responded with patients. The final cohort included 568,893 patients who matched the query criteria listed in the table below. For the text representation of the query criteria please see Appendix A.

|  | | | | | |
| --- | --- | --- | --- | --- | --- |
| Ungrouped terms | | | | | |
|  | must have |  | demographics | Age | Age (at least 18 years (most recent occurrence)) |
|  |  | and | demographics | UMLS:HL7V3.0:Gender:M | Male |
|  |  | and | diagnosis | UMLS:ICD10CM:E11 | Type 2 diabetes mellitus (between 20 and 80 years old at event) |
|  |  | and any of | diagnosis | UMLS:ICD10CM:N52 | Male erectile dysfunction |
|  |  |  | diagnosis | UMLS:ICD10CM:N52.0 | Vasculogenic erectile dysfunction |
|  |  |  | diagnosis | UMLS:ICD10CM:N52.8 | Other male erectile dysfunction |
|  |  |  | diagnosis | UMLS:ICD10CM:N52.9 | Male erectile dysfunction, unspecified |
|  |  |  | diagnosis | UMLS:ICD10CM:N52.02 | Corporo-venous occlusive erectile dysfunction |
|  |  |  | medication | NLM:ATC:G04BE | Drugs used in erectile dysfunction |
|  |  |  | diagnosis | UMLS:ICD10CM:N50.1 | Vascular disorders of male genital organs |
|  |  |  | diagnosis | UMLS:ICD10CM:N52.1 | Erectile dysfunction due to diseases classified elsewhere |
|  |  |  | diagnosis | UMLS:ICD10CM:N52.01 | Erectile dysfunction due to arterial insufficiency |
|  | cannot have |  | diagnosis | UMLS:ICD10CM:E08 | Diabetes mellitus due to underlying condition |
|  |  | or | diagnosis | UMLS:ICD10CM:E09 | Drug or chemical induced diabetes mellitus |
|  |  | or | diagnosis | UMLS:ICD10CM:E10 | Type 1 diabetes mellitus |
|  |  | or | diagnosis | UMLS:ICD10CM:E13 | Other specified diabetes mellitus |

### Query Criteria for Cohort 2 (query name: T2DM without ED)

This query was run on the network Global Collaborative Network with 161 HCO(s) queried and 161 HCO(s) responded. A total of 157 provider(s) responded with patients. The final cohort included 3,428,405 patients who matched the query criteria listed in the table below.

| Ungrouped terms | | | | | |
| --- | --- | --- | --- | --- | --- |
|  | must have |  | demographics | Age | Age (at least 18 years (most recent occurrence)) |
|  |  | and | demographics | UMLS:HL7V3.0:Gender:M | Male |
|  |  | and | diagnosis | UMLS:ICD10CM:E11 | Type 2 diabetes mellitus (between 20 and 80 years old at event) |
|  | cannot have |  | diagnosis | UMLS:ICD10CM:E08 | Diabetes mellitus due to underlying condition |
|  |  | or | diagnosis | UMLS:ICD10CM:E09 | Drug or chemical induced diabetes mellitus |
|  |  | or | diagnosis | UMLS:ICD10CM:E10 | Type 1 diabetes mellitus |
|  |  | or | diagnosis | UMLS:ICD10CM:E13 | Other specified diabetes mellitus |
|  |  | or | diagnosis | UMLS:ICD10CM:N52 | Male erectile dysfunction |
|  |  | or | diagnosis | UMLS:ICD10CM:N52.0 | Vasculogenic erectile dysfunction |
|  |  | or | diagnosis | UMLS:ICD10CM:N52.8 | Other male erectile dysfunction |
|  |  | or | diagnosis | UMLS:ICD10CM:N52.02 | Corporo-venous occlusive erectile dysfunction |
|  |  | or | diagnosis | UMLS:ICD10CM:N52.9 | Male erectile dysfunction, unspecified |
|  |  | or | medication | NLM:ATC:G04BE | Drugs used in erectile dysfunction |
|  |  | or | diagnosis | UMLS:ICD10CM:N52.01 | Erectile dysfunction due to arterial insufficiency |
|  |  | or | diagnosis | UMLS:ICD10CM:N50.1 | Vascular disorders of male genital organs |
|  |  | or | diagnosis | UMLS:ICD10CM:N52.1 | Erectile dysfunction due to diseases classified elsewhere |

## Analysis Setup

This section contains the Index Event and Time Window definitions and a list of selected outcomes and the analyses.

### Index Event & Time Window Definitions

The index event defines the point in time when each patient in the cohort enters the analysis. To define an index event for the cohort, one or more criteria for the cohort must be selected. The index date for each patient within a cohort is the day on which the patient first met the selected criteria for the cohort (listed in the table below).

As the index event defines the earliest time point after which outcomes are analyzed, the time window defines the duration during which outcomes are analyzed. The time window can start on the same day as the index event or at any specified time interval after the index event. The time window can end any time after the start date. Outcomes are defined as diagnoses, medications, procedures, or laboratory values that happened in the time window starting after the first occurrence of the index event.

### Time Window Used in this Analysis

This analysis included outcomes that occurred in the time window that started 1 day after the first occurrence of the index event. Since no end date was specified all outcomes after the first occurrence of the index event were included.

The index event only includes events that occurred up to 20 years ago. Patients whose index event occurred 20 years or more ago are excluded. In this analysis, 0 patients in Cohort 1 and 0 patients in Cohort 2 were excluded because they met the index event more than 20 years ago.

### Index Events Used in this Analysis

Index events for the Compare Outcomes analysis were derived from the cohort definitions. Index events were defined separately for each cohort and were based on the criteria used in the original cohort definition. Please see Appendix B for the text representation of the index event definition.

The index event for Cohort 1 (query name: T2DM with ED) was defined as the following:

|  | | | | | |
| --- | --- | --- | --- | --- | --- |
| Ungrouped terms | | | | | |
|  | must have |  | diagnosis | UMLS:ICD10CM:E11 | Type 2 diabetes mellitus (between 20 and 80 years old at event) |
|  |  | and any of | diagnosis | UMLS:ICD10CM:N52 | Male erectile dysfunction |
|  |  |  | diagnosis | UMLS:ICD10CM:N52.0 | Vasculogenic erectile dysfunction |
|  |  |  | diagnosis | UMLS:ICD10CM:N52.8 | Other male erectile dysfunction |
|  |  |  | diagnosis | UMLS:ICD10CM:N52.9 | Male erectile dysfunction, unspecified |
|  |  |  | diagnosis | UMLS:ICD10CM:N52.02 | Corporo-venous occlusive erectile dysfunction |
|  |  |  | medication | NLM:ATC:G04BE | Drugs used in erectile dysfunction |
|  |  |  | diagnosis | UMLS:ICD10CM:N50.1 | Vascular disorders of male genital organs |
|  |  |  | diagnosis | UMLS:ICD10CM:N52.1 | Erectile dysfunction due to diseases classified elsewhere |
|  |  |  | diagnosis | UMLS:ICD10CM:N52.01 | Erectile dysfunction due to arterial insufficiency |

The index event for Cohort 2 (query name: T2DM without ED) was defined as the following:

|  | | | | | |
| --- | --- | --- | --- | --- | --- |
| Ungrouped terms | | | | | |
|  | must have |  | diagnosis | UMLS:ICD10CM:E11 | Type 2 diabetes mellitus (between 20 and 80 years old at event) |

### Analyses Specifications

The Compare Outcomes Analytic supports four types of analyses: Measure of Association, Survival, Number of Instances, and Lab result distribution. The first three analyses support the “exclude patients with outcomes prior to the window” setting. This option can exclude patients from the analysis if they are not at risk for an outcome (e.g., if the outcome is a chronic disease). When "exclude patients with the outcome prior to the time window" is not checked, all patients in the cohort are included in the analysis, regardless of whether they had the outcome prior to the time window. When "exclude patients with the outcome prior to the time window" is checked, patients are excluded from the analysis if their record includes the outcome prior to the beginning of the time window. This selection will exclude all patients with the outcome prior to the index event. If the start of the time window for the analysis falls some days after the index event, patients will also be excluded if they have the outcome between the index event and the start of the time window.

### Measure of Association Analysis

The Measure of Association Analysis calculates and compares the fraction of patients with the selected outcome. The output summary includes: Patients in each Cohort (count of patients meeting query criteria); Patients with Outcome in each Cohort (of the patients in the cohort, count of patients that had the outcome in the time window); and Risk (the fraction of patients in the cohort that have the outcome in the time window, i.e. Patients with Outcome / Patients in Cohort). In addition, Risk Difference (the difference in the risks in Cohort 1 and Cohort 2), Risk Ratio (the ratio of the risks in Cohort 1 and Cohort 2), and Odds Ratio (the ratio of the odds in Cohort 1 and Cohort 2). The bar chart shows the risk of the outcome for the both cohorts.

### Survival Analysis

The Kaplan-Meier Analysis estimates probability of the outcome at a respective time interval (daily time interval is used in this analysis). In order to account for the patients who exited the cohort during the analysis period, and therefore should not be included in the analysis, censoring is applied. In this analysis, patients are removed from the analysis (censored) after the last fact in their record.

The output summary includes: Patients in each Cohort (count of patients meeting query criteria); Patients with Outcome (of the patients in the cohort, count of patients that had the outcome in the time window); Median Survival (the number of days when the survival drops below 50%; the “-” indicates that survival does not drop below 50% during the time window); and Survival Probability at End of Time Window (the % survival at the end of the time window). In addition, Log-Rank test, Hazard Ratio and test for Proportionality.

### Number of Instances Analysis

The Number of Instances Analysis calculates how many times the outcome occurred in the time window. This analysis includes two additional settings: include patients with zero instances; the definition of an instance.

Selecting to exclude patients with zero instances will remove these patients from the calculations for mean number of instances, standard deviation, or median. The histogram showing the distribution of patients by number of instances will not contain a bar for zero. Alternatively, by selecting to include patients with zero instances, the mean, standard deviation, and median for number of instances will reflect these patients. The histogram will contain a bar for zero patients.

The definition of an instance affects how counts are analyzed. By selecting Date, each calendar date on which any of the terms selected in the outcome are recorded will represent one instance. For example, if the outcome is “Med A or Med B,” and a patient has “Med A” on January 3, then both medications on January 4, then “Med B” on January 6, then that patient is considered to have three instances– January 3, January 4, and January 6. Note that if an outcome occurs across several dates (e.g. Visit: inpatient encounter), then only the start date is tracked for the purpose of counting instances. A patient who begins at stay on January 1, ends that stay on January 3, begins another stay on January 10, and ends that stay on January 15, is considered to have two instances of the outcome.

Selecting Visit as an Instance will count any visit that includes the outcome as one instance, regardless of how many times it occurred. For instance, consider a patient administered an analgesic on each of the three days that make up an inpatient stay following some index event. If analgesic is an outcome, these three administrations will represent only one instance, because all three are associated with the same visit.

The output summary includes: Patients in Cohort (count of patients meeting query criteria); Patients with Outcome (of the patients in the cohort, count of patients that had the outcome in the time window); Mean (mean of the counts); Standard Deviation (standard deviation of the counts); Median (median of the counts); and Median (1+ instances) when patients with zero instances included in the analysis. In addition, T-Test statistics testing for the difference between the cohorts is included.

### Laboratory Results Analysis

Lab Results can be included in the analysis only for the outcomes that are labs. Only the most recent lab values in the time window are included. For the lab results that are numeric, the outcome summary includes: Patients in Cohort (count of patients meeting query criteria); Patients with Outcome (of the patients in the cohort, count of patients that had the outcome in the time window); Mean (mean of the counts); and Standard Deviation (the standard deviation for lab values across patients in the cohort). In addition, T-Test statistics testing for the difference between the cohorts is included.

For the non-numeric lab results, three values are reported: counts of Negative; Positives; and Unknowns.

The counts are represented in the bar chart as percentages of the total counts.

### Outcome Definitions

Table below outlines the definitions for each outcome and the analysis specifications. For outcome definitions consisting of more than one term, at least one term must match. Please see Appendix C for the text representation of the outcome definitions.

| Ischemic hearth disease | | | | |
| --- | --- | --- | --- | --- |
|  | Outcome definition | | | |
|  | | Diagnosis | UMLS:ICD10CM:I20-I25 | Ischemic heart diseases |
|  | Settings for the performed analyses | | | |
|  | | Kaplan - Meier survival analysis | | excluding patients with outcome prior to the time window |
|  | | Risk analysis | | excluding patients with outcome prior to the time window |
|  | | Number of instances analysis | | excluding patients with outcome prior to the time window excluding patients with zero outcomes counts are grouped by date |
| Stroke | | | | |
|  | Outcome definition | | | |
|  | | Diagnosis | UMLS:ICD10CM:I60-I69 | Cerebrovascular diseases |
|  | Settings for the performed analyses | | | |
|  | | Risk analysis | | excluding patients with outcome prior to the time window |
|  | | Kaplan - Meier survival analysis | | excluding patients with outcome prior to the time window |
|  | | Number of instances analysis | | excluding patients with outcome prior to the time window excluding patients with zero outcomes counts are grouped by date |
| Heart failure | | | | |
|  | Outcome definition | | | |
|  | | Diagnosis | UMLS:ICD10CM:I50 | Heart failure |
|  | Settings for the performed analyses | | | |
|  | | Risk analysis | | excluding patients with outcome prior to the time window |
|  | | Kaplan - Meier survival analysis | | excluding patients with outcome prior to the time window |
|  | | Number of instances analysis | | excluding patients with outcome prior to the time window excluding patients with zero outcomes counts are grouped by date |
| Peripheral artery disease | | | | |
|  | Outcome definition | | | |
|  | | Diagnosis | UMLS:ICD10CM:I80-I89 | Diseases of veins, lymphatic vessels and lymph nodes, not elsewhere classified |
|  | Settings for the performed analyses | | | |
|  | | Risk analysis | | excluding patients with outcome prior to the time window |
|  | | Kaplan - Meier survival analysis | | excluding patients with outcome prior to the time window |
|  | | Number of instances analysis | | excluding patients with outcome prior to the time window excluding patients with zero outcomes counts are grouped by date |
| CVD | | | | |
|  | Outcome definition | | | |
|  | | Diagnosis | UMLS:ICD10CM:I20-I25 | Ischemic heart diseases |
|  | | Diagnosis | UMLS:ICD10CM:I60-I69 | Cerebrovascular diseases |
|  | | Diagnosis | UMLS:ICD10CM:I80-I89 | Diseases of veins, lymphatic vessels and lymph nodes, not elsewhere classified |
|  | | Diagnosis | UMLS:ICD10CM:I50 | Heart failure |
|  | Settings for the performed analyses | | | |
|  | | Risk analysis | | excluding patients with outcome prior to the time window |
|  | | Kaplan - Meier survival analysis | | excluding patients with outcome prior to the time window |
|  | | Number of instances analysis | | excluding patients with outcome prior to the time window excluding patients with zero outcomes counts are grouped by date |

## Propensity Score Matching

Propensity score matching was performed on 4 characteristic(s). In the Demographics category patients were matched on Age at Index characteristic(s). In the Diagnosis category patients were matched on Hypertensive diseases, Disorders of lipoprotein metabolism and other lipidemias, Nicotine dependence, unspecified, uncomplicated characteristic(s). Characteristics of the cohorts before and after matching are summarized in the table below.

| Cohort 1 and cohort 2 patient count before and after propensity score matching | | | | | | | | | | | | |
| --- | --- | --- | --- | --- | --- | --- | --- | --- | --- | --- | --- | --- |
|  | | | Cohort | | | Patient count before matching | | | | Patient count after matching | | |
|  | | | 1 - T2DM with ED | | | 559,583 | | | | 555,508 | | |
|  | | | 2 - T2DM without ED | | | 3,412,080 | | | | 555,508 | | |
| Propensity score density function - Before and after matching (cohort 1 - purple, cohort 2 - green) | | | | | | | | | | | | |
|  |  | | 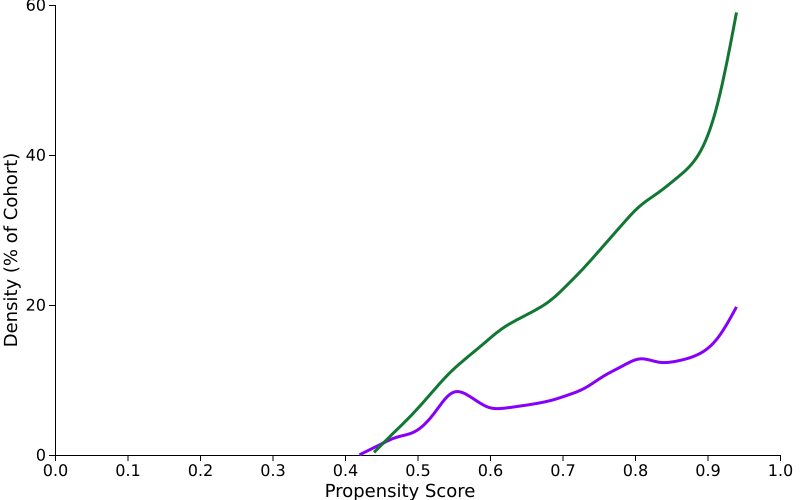 | | | | 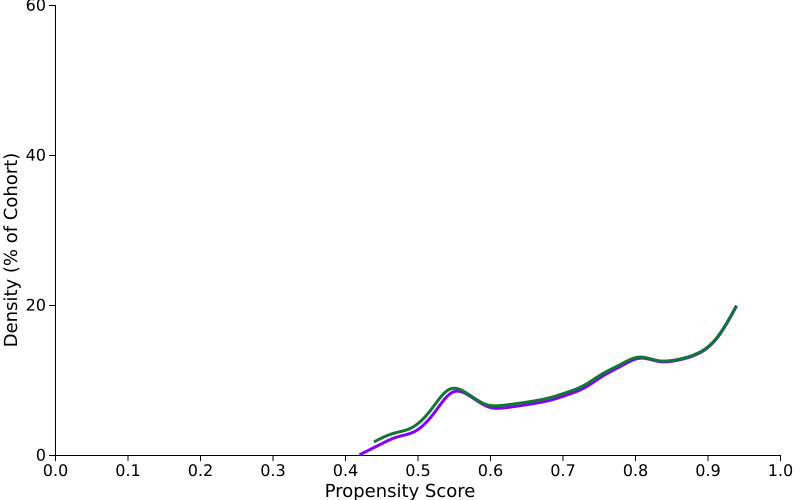 | | | | | |
| Cohort 1 (N = 559,583) and cohort 2 (N = 3,412,080) characteristics before propensity score matching | | | | | | | | | | | | |
|  | Demographics | | | | | | | | | | | |
|  |  | Cohort | | |  | Mean ± SD | | Patients | % of Cohort | | P-Value | Std diff. |
|  |  | 1 2 | | AI | Age at Index | 61.1 +/- 10.4 59.0 +/- 12.7 | | 555,508 3,340,610 | 100% 100% | | <0.001 | 0.183 |
|  | Diagnosis | | | | | | | | | | | |
|  |  | Cohort | | |  | Mean ± SD | | Patients | % of Cohort | | P-Value | Std diff. |
|  |  | 1 2 | | I10-I1A | Hypertensive diseases |  | | 367,715 652,169 | 66.2% 19.5% | | <0.001 | 1.069 |
|  |  | 1 2 | | I50 | Heart failure |  | | 61,364 110,545 | 11.0% 3.3% | | <0.001 | 0.303 |
|  |  | 1 2 | | I20-I25 | Ischemic heart diseases |  | | 151,294 279,758 | 27.2% 8.4% | | <0.001 | 0.509 |
|  |  | 1 2 | | I60-I69 | Cerebrovascular diseases |  | | 55,890 118,511 | 10.1% 3.5% | | <0.001 | 0.261 |
|  |  | 1 2 | | I70-I79 | Diseases of arteries, arterioles and capillaries |  | | 79,060 128,701 | 14.2% 3.9% | | <0.001 | 0.368 |
|  |  | 1 2 | | E78 | Disorders of lipoprotein metabolism and other lipidemias |  | | 336,630 475,858 | 60.6% 14.2% | | <0.001 | 1.091 |
|  |  | 1 2 | | N18 | Chronic kidney disease (CKD) |  | | 73,962 119,487 | 13.3% 3.6% | | <0.001 | 0.356 |
|  |  | 1 2 | | F17.200 | Nicotine dependence, unspecified, uncomplicated |  | | 60,804 118,741 | 10.9% 3.6% | | <0.001 | 0.288 |
|  | Procedure | | | | | | | | | | | |
|  |  | Cohort | | |  | Mean ± SD | | Patients | % of Cohort | | P-Value | Std diff. |
|  |  | 1 2 | | 1018513 | Smoking and tobacco use cessation counseling visit |  | | 7,166 10,311 | 1.3% 0.3% | | <0.001 | 0.110 |
|  | Laboratory | | | | | | | | | | | |
|  |  | Cohort | | |  | Mean ± SD | | Patients | % of Cohort | | P-Value | Std diff. |
|  |  | 1 2 | | 9024 | Creatinine [Mass/volume] in Serum, Plasma or Blood | 1.4 +/- 4.2 1.4 +/- 5.4 | | 419,804 1,081,815 | 75.6% 32.4% | | <0.001 | 0.012 |
|  |  | 1 2 | |  | 0 - 0 mg/dL |  | | 419,820 1,081,912 | 75.6% 32.4% | | <0.001 | 0.961 |
|  |  | 1 2 | | 9000 | Cholesterol [Mass/volume] in Serum or Plasma | 164.4 +/- 46.7 172.2 +/- 49.5 | | 326,523 600,666 | 58.8% 18.0% | | <0.001 | 0.162 |
|  |  | 1 2 | |  | 0 - 0 mg/dL |  | | 326,529 600,686 | 58.8% 18.0% | | <0.001 | 0.924 |
|  |  | 1 2 | | 9002 | Cholesterol in LDL [Mass/volume] in Serum or Plasma | 91.3 +/- 37.2 98.0 +/- 38.4 | | 324,562 588,710 | 58.4% 17.6% | | <0.001 | 0.178 |
|  |  | 1 2 | |  | 0 - 0 mg/dL |  | | 324,584 588,767 | 58.4% 17.6% | | <0.001 | 0.926 |
|  |  | 1 2 | | 9001 | Cholesterol in HDL [Mass/volume] in Serum or Plasma | 40.3 +/- 14.6 40.2 +/- 14.2 | | 328,183 594,834 | 59.1% 17.8% | | 0.006 | 0.006 |
|  |  | 1 2 | |  | 0 - 0 mg/dL |  | | 328,186 594,864 | 59.1% 17.8% | | <0.001 | 0.937 |
|  |  | 1 2 | | 9004 | Triglyceride [Mass/volume] in Serum, Plasma or Blood | 170.1 +/- 163.6 181.7 +/- 199.6 | | 326,299 595,689 | 58.7% 17.8% | | <0.001 | 0.064 |
|  |  | 1 2 | |  | 0 - 0 mg/dL |  | | 326,299 595,690 | 58.7% 17.8% | | <0.001 | 0.928 |
|  |  | 1 2 | | 9037 | Hemoglobin A1c/Hemoglobin.total in Blood | 7.1 +/- 1.8 7.1 +/- 1.8 | | 340,933 624,831 | 61.4% 18.7% | | <0.001 | 0.010 |
|  |  | 1 2 | |  | 0 - 0 % |  | | 340,938 624,873 | 61.4% 18.7% | | <0.001 | 0.967 |
|  |  | 1 2 | | 9083 | BMI | 32.2 +/- 6.6 32.5 +/- 7.5 | | 350,386 945,040 | 63.1% 28.3% | | <0.001 | 0.044 |
|  |  | 1 2 | |  | 0 - 0 kg/m2 |  | | 350,452 945,777 | 63.1% 28.3% | | <0.001 | 0.745 |
|  |  | 1 2 | | 9085 | Blood Pressure, Systolic | 131.9 +/- 18.7 133.5 +/- 20.0 | | 398,775 1,085,031 | 71.8% 32.5% | | <0.001 | 0.083 |
|  |  | 1 2 | |  | 0 - 0 mm[Hg] |  | | 398,777 1,085,078 | 71.8% 32.5% | | <0.001 | 0.856 |
|  |  | 1 2 | | 9086 | Blood Pressure, Diastolic | 77.2 +/- 11.7 78.3 +/- 12.7 | | 398,991 1,085,248 | 71.8% 32.5% | | <0.001 | 0.093 |
|  |  | 1 2 | |  | 0 - 0 mm[Hg] |  | | 399,008 1,085,340 | 71.8% 32.5% | | <0.001 | 0.857 |
|  |  | 1 2 | | 88031-0 | Smokeless tobacco status |  | | 0 0 | 0% 0% | | -- | -- |
|  |  | 1 2 | |  | 0 - 0 units |  | | 0 0 | 0% 0% | | -- | -- |
| Cohort 1 (N = 555,508) and cohort 2 (N = 555,508) characteristics after propensity score matching | | | | | | | | | | | | |
|  | Demographics | | | | | | | | | | | |
|  |  | Cohort | | |  | Mean ± SD | | Patients | % of Cohort | | P-Value | Std diff. |
|  |  | 1 2 | | AI | Age at Index | 61.1 +/- 10.4 61.0 +/- 10.4 | | 555,508 555,508 | 100% 100% | | <0.001 | 0.015 |
|  | Diagnosis | | | | | | | | | | | |
|  |  | Cohort | | |  | Mean ± SD | | Patients | % of Cohort | | P-Value | Std diff. |
|  |  | 1 2 | | I10-I1A | Hypertensive diseases |  | | 367,715 367,437 | 66.2% 66.1% | | 0.577 | 0.001 |
|  |  | 1 2 | | I50 | Heart failure |  | | 61,364 52,575 | 11.0% 9.5% | | <0.001 | 0.052 |
|  |  | 1 2 | | I20-I25 | Ischemic heart diseases |  | | 151,294 136,268 | 27.2% 24.5% | | <0.001 | 0.062 |
|  |  | 1 2 | | I60-I69 | Cerebrovascular diseases |  | | 55,890 50,330 | 10.1% 9.1% | | <0.001 | 0.034 |
|  |  | 1 2 | | I70-I79 | Diseases of arteries, arterioles and capillaries |  | | 79,060 61,901 | 14.2% 11.1% | | <0.001 | 0.093 |
|  |  | 1 2 | | E78 | Disorders of lipoprotein metabolism and other lipidemias |  | | 336,630 336,908 | 60.6% 60.6% | | 0.589 | 0.001 |
|  |  | 1 2 | | N18 | Chronic kidney disease (CKD) |  | | 73,962 50,858 | 13.3% 9.2% | | <0.001 | 0.132 |
|  |  | 1 2 | | F17.200 | Nicotine dependence, unspecified, uncomplicated |  | | 60,804 62,530 | 10.9% 11.3% | | <0.001 | 0.010 |
|  | Procedure | | | | | | | | | | | |
|  |  | Cohort | | |  | Mean ± SD | | Patients | % of Cohort | | P-Value | Std diff. |
|  |  | 1 2 | | 1018513 | Smoking and tobacco use cessation counseling visit |  | | 7,166 6,242 | 1.3% 1.1% | | <0.001 | 0.015 |
|  | Laboratory | | | | | | | | | | | |
|  |  | Cohort | | |  | Mean ± SD | | Patients | % of Cohort | | P-Value | Std diff. |
|  |  | 1 2 | | 9024 | Creatinine [Mass/volume] in Serum, Plasma or Blood | 1.4 +/- 4.2 1.3 +/- 4.4 | | 419,804 335,761 | 75.6% 60.4% | | 0.006 | 0.006 |
|  |  | 1 2 | |  | 0 - 0 mg/dL |  | | 419,820 335,771 | 75.6% 60.4% | | <0.001 | 0.329 |
|  |  | 1 2 | | 9000 | Cholesterol [Mass/volume] in Serum or Plasma | 164.4 +/- 46.7 170.6 +/- 48.1 | | 326,523 240,489 | 58.8% 43.3% | | <0.001 | 0.131 |
|  |  | 1 2 | |  | 0 - 0 mg/dL |  | | 326,529 240,492 | 58.8% 43.3% | | <0.001 | 0.314 |
|  |  | 1 2 | | 9002 | Cholesterol in LDL [Mass/volume] in Serum or Plasma | 91.3 +/- 37.2 96.4 +/- 38.3 | | 324,562 237,943 | 58.4% 42.8% | | <0.001 | 0.136 |
|  |  | 1 2 | |  | 0 - 0 mg/dL |  | | 324,584 237,955 | 58.4% 42.8% | | <0.001 | 0.316 |
|  |  | 1 2 | | 9001 | Cholesterol in HDL [Mass/volume] in Serum or Plasma | 40.3 +/- 14.6 40.3 +/- 14.3 | | 328,183 239,520 | 59.1% 43.1% | | 0.472 | 0.002 |
|  |  | 1 2 | |  | 0 - 0 mg/dL |  | | 328,186 239,525 | 59.1% 43.1% | | <0.001 | 0.323 |
|  |  | 1 2 | | 9004 | Triglyceride [Mass/volume] in Serum, Plasma or Blood | 170.1 +/- 163.6 178.3 +/- 171.5 | | 326,299 238,295 | 58.7% 42.9% | | <0.001 | 0.049 |
|  |  | 1 2 | |  | 0 - 0 mg/dL |  | | 326,299 238,295 | 58.7% 42.9% | | <0.001 | 0.321 |
|  |  | 1 2 | | 9037 | Hemoglobin A1c/Hemoglobin.total in Blood | 7.1 +/- 1.8 6.8 +/- 1.5 | | 340,933 223,255 | 61.4% 40.2% | | <0.001 | 0.203 |
|  |  | 1 2 | |  | 0 - 0 % |  | | 340,938 223,261 | 61.4% 40.2% | | <0.001 | 0.434 |
|  |  | 1 2 | | 9083 | BMI | 32.2 +/- 6.6 32.6 +/- 7.3 | | 350,386 278,869 | 63.1% 50.2% | | <0.001 | 0.064 |
|  |  | 1 2 | |  | 0 - 0 kg/m2 |  | | 350,452 279,006 | 63.1% 50.2% | | <0.001 | 0.262 |
|  |  | 1 2 | | 9085 | Blood Pressure, Systolic | 131.9 +/- 18.7 133.3 +/- 19.7 | | 398,775 318,288 | 71.8% 57.3% | | <0.001 | 0.070 |
|  |  | 1 2 | |  | 0 - 0 mm[Hg] |  | | 398,777 318,294 | 71.8% 57.3% | | <0.001 | 0.306 |
|  |  | 1 2 | | 9086 | Blood Pressure, Diastolic | 77.2 +/- 11.7 78.3 +/- 12.4 | | 398,991 318,379 | 71.8% 57.3% | | <0.001 | 0.094 |
|  |  | 1 2 | |  | 0 - 0 mm[Hg] |  | | 399,008 318,395 | 71.8% 57.3% | | <0.001 | 0.307 |
|  |  | 1 2 | | 88031-0 | Smokeless tobacco status |  | | 0 0 | 0% 0% | | -- | -- |
|  |  | 1 2 | |  | 0 - 0 units |  | | 0 0 | 0% 0% | | -- | -- |

# Results

Results are summarized in the tables below. Outcomes analysis was performed on the cohorts after propensity score matching.

| Follow-up Time (Before Matching) | | | | | | |
| --- | --- | --- | --- | --- | --- | --- |
|  |  | Cohort | Mean Follow-up (Days) | Standard Deviation | Median Follow-up (Days) | Interquartile Range |
|  |  | T2DM with ED | 1528.561 | 1355.267 | 1160 | 1850 |
|  |  | T2DM without ED | 1233.571 | 1361.267 | 768 | 1858 |
| 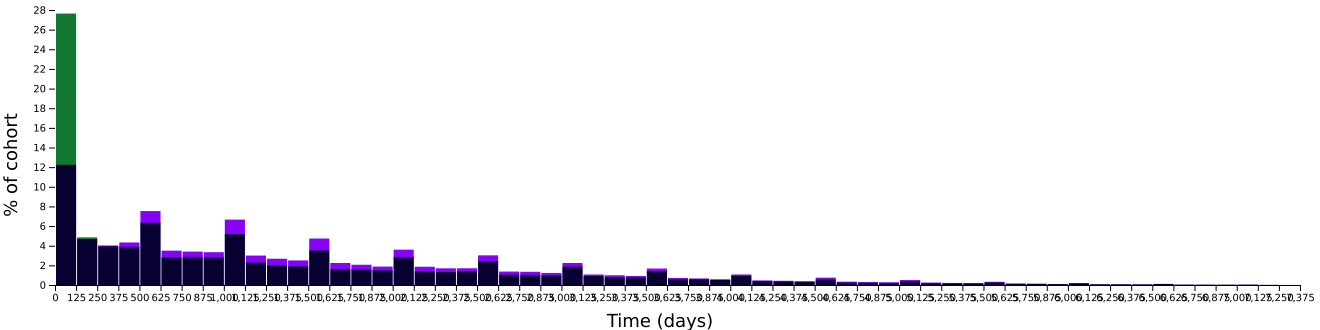 | | | | | | |
| 4,075 patients in Cohort 1 and 71,470 patients in Cohort 2 were excluded because they met the index event more than 20 years ago. | | | | | | |
|  | | | | | | |
| Follow-up Time (After Matching) | | | | | | |
|  |  | Cohort | Mean Follow-up (Days) | Standard Deviation | Median Follow-up (Days) | Interquartile Range |
|  |  | T2DM with ED | 1528.561 | 1355.267 | 1160 | 1850 |
|  |  | T2DM without ED | 1368.141 | 1350.528 | 960 | 1859 |
| 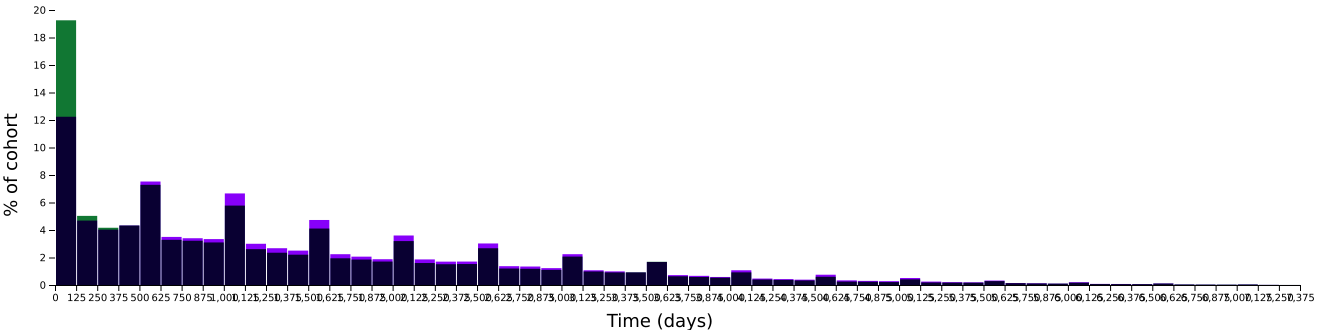 | | | | | | |

| 1 Ischemic hearth disease | | | | | | | | | | | | |
| --- | --- | --- | --- | --- | --- | --- | --- | --- | --- | --- | --- | --- |
|  | | Risk analysis excluding patients with outcome prior to the time window | | | | | | | | | | |
|  |  | | | Cohort | | | Patients in cohort | Patients with outcome | Risk | | | |
|  | | |  | 1 | | T2DM with ED | 385,971 | 65,253 | 0.169 | | | |
|  | | |  | 2 | | T2DM without ED | 382,050 | 59,504 | 0.156 | | | |
|  | | | | | | | | | | | | |
|  | | |  |  | | |  | 95% CI | z | p |  |  |
|  | | |  | Risk Difference | | | 0.013 | (0.012, 0.015) | 15.815 | 0.000 |  |  |
|  | | |  | Risk Ratio | | | 1.085 | (1.074, 1.097) | N/A | N/A |  |  |
|  | | |  | Odds Ratio | | | 1.103 | (1.090, 1.116) | N/A | N/A |  |  |
|  | | | | | | | | | | | | |
|  | |  | | | 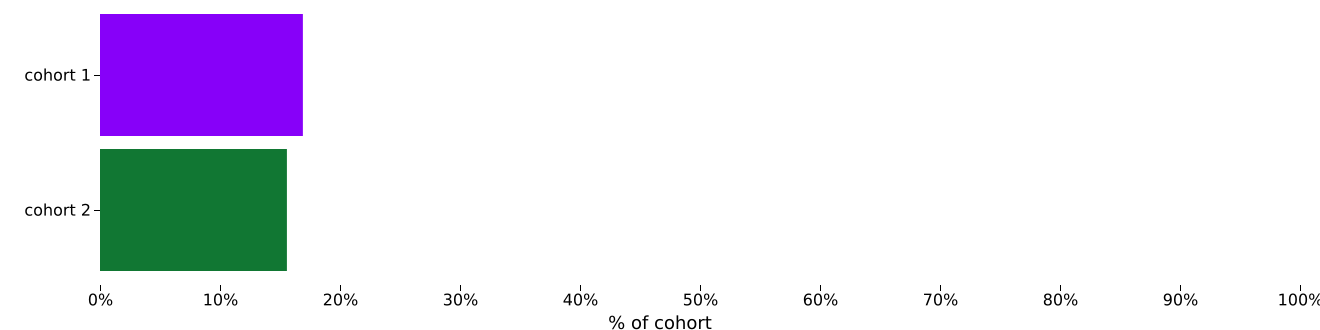 | | | | | | | |
|  | |  | | | 169,537 patients in Cohort 1 and 173,458 patients in Cohort 2 were excluded from results because they had the outcome prior to the time window. | | | | | | | |
|  | | Kaplan - Meier survival analysis excluding patients with outcome prior to the time window | | | | | | | | | | |
|  | | |  | Cohort | | | Patients in cohort | Patients with outcome | Median survival (days) | Survival probability at end of time window | | |
|  | | |  | 1 | | T2DM with ED | 385,971 | 65,253 | 5733 | 39.00% | | |
|  | | |  | 2 | | T2DM without ED | 382,050 | 59,504 | 5845 | 40.86% | | |
|  | | | | | | | | | | | | |
|  | | |  |  | | | χ^2^ | df | p |  |  |  |
|  | | |  | Log-Rank Test | | | 71.241 | 1 | 0.000 |  |  |  |
|  | | | | | | | | | | | | |
|  | | |  |  | | | Hazard Ratio | 95% CI | χ^2^ | df | p | |
|  | | |  | Hazard Ratio and Proportionality | | | 0.953 | (0.943, 0.964) | 160.843 | 1 | 0.000 | |
|  | | | | | | | | | | | | |
|  | |  | | | 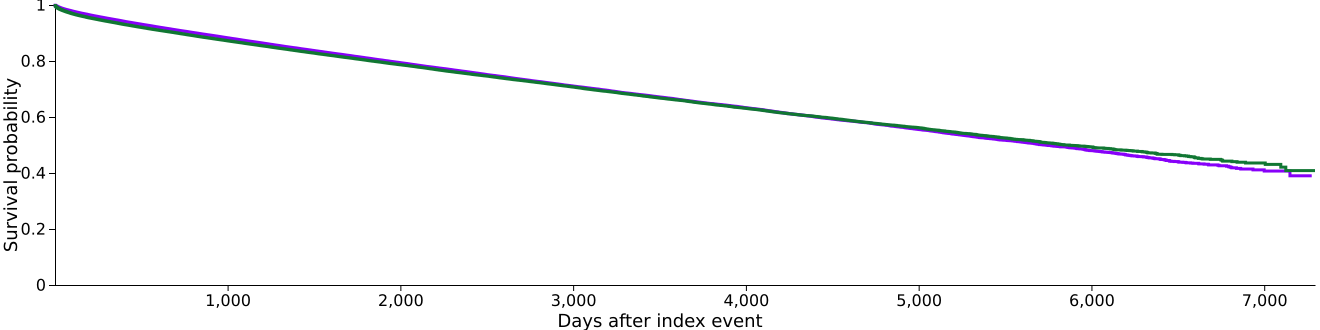 | | | | | | | |
|  | |  | | | 169,537 patients in Cohort 1 and 173,458 patients in Cohort 2 were excluded from results because they had the outcome prior to the time window. | | | | | | | |
|  | | Number of instances excluding patients with outcome prior to the time window | | | | | | | | | | |
|  | | |  | Cohort | | | Patients in cohort | Patients with outcome | Mean | Standard Deviation | Median | |
|  | | |  | 1 | | T2DM with ED | 385,971 | 65,253 | 7.911 | 14.450 | 3 | |
|  | | |  | 2 | | T2DM without ED | 382,050 | 59,504 | 6.920 | 13.320 | 2 | |
|  | | | | | | | | | | | | |
|  | | |  |  | | | t | df | p |  |  |  |
|  | | |  | Test Statistics | | | 12.562 | 124755 | 0.000 |  |  |  |
|  | | | | | | | | | | | | |
|  | |  | | | 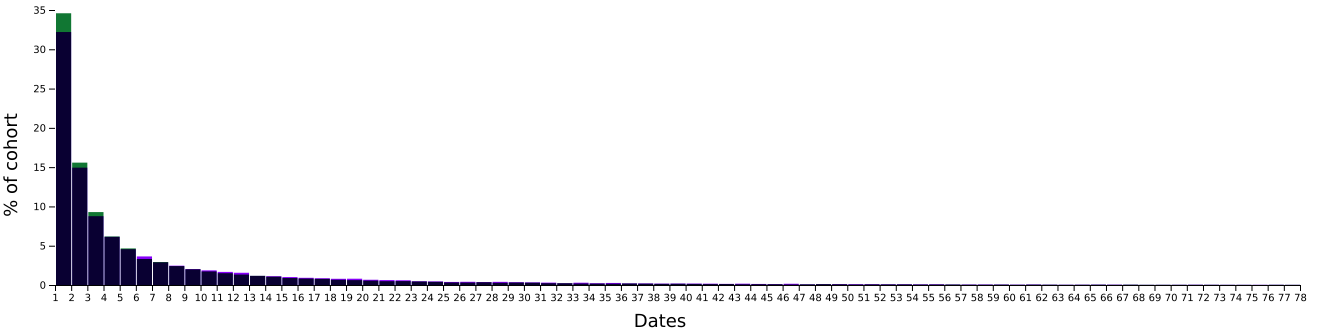 | | | | | | | |
|  | |  | | | 382 data points for Cohort 1 and 286 data points for Cohort 2 were omitted for display purposes. 169,537 patients in Cohort 1 and 173,458 patients in Cohort 2 were excluded from results because they had the outcome prior to the time window. | | | | | | | |
| 2 Stroke | | | | | | | | | | | | |
|  | | Risk analysis excluding patients with outcome prior to the time window | | | | | | | | | | |
|  |  | | | Cohort | | | Patients in cohort | Patients with outcome | Risk | | | |
|  | | |  | 1 | | T2DM with ED | 493,588 | 44,683 | 0.091 | | | |
|  | | |  | 2 | | T2DM without ED | 490,722 | 41,161 | 0.084 | | | |
|  | | | | | | | | | | | | |
|  | | |  |  | | |  | 95% CI | z | p |  |  |
|  | | |  | Risk Difference | | | 0.007 | (0.006, 0.008) | 11.689 | 0.000 |  |  |
|  | | |  | Risk Ratio | | | 1.079 | (1.066, 1.093) | N/A | N/A |  |  |
|  | | |  | Odds Ratio | | | 1.087 | (1.072, 1.102) | N/A | N/A |  |  |
|  | | | | | | | | | | | | |
|  | |  | | | 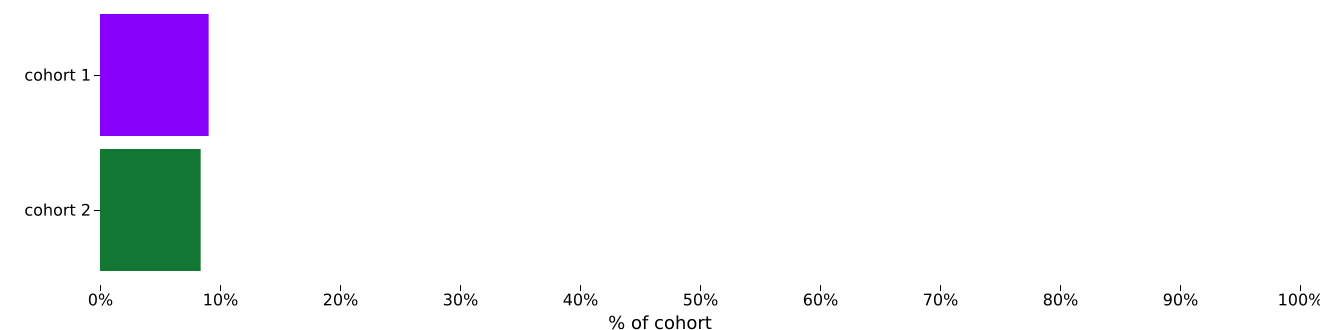 | | | | | | | |
|  | |  | | | 61,920 patients in Cohort 1 and 64,786 patients in Cohort 2 were excluded from results because they had the outcome prior to the time window. | | | | | | | |
|  | | Kaplan - Meier survival analysis excluding patients with outcome prior to the time window | | | | | | | | | | |
|  | | |  | Cohort | | | Patients in cohort | Patients with outcome | Median survival (days) | Survival probability at end of time window | | |
|  | | |  | 1 | | T2DM with ED | 493,588 | 44,683 | -- | 55.25% | | |
|  | | |  | 2 | | T2DM without ED | 490,722 | 41,161 | -- | 58.31% | | |
|  | | | | | | | | | | | | |
|  | | |  |  | | | χ^2^ | df | p |  |  |  |
|  | | |  | Log-Rank Test | | | 23.270 | 1 | 0.000 |  |  |  |
|  | | | | | | | | | | | | |
|  | | |  |  | | | Hazard Ratio | 95% CI | χ^2^ | df | p | |
|  | | |  | Hazard Ratio and Proportionality | | | 0.968 | (0.955, 0.981) | 116.012 | 1 | 0.000 | |
|  | | | | | | | | | | | | |
|  | |  | | | 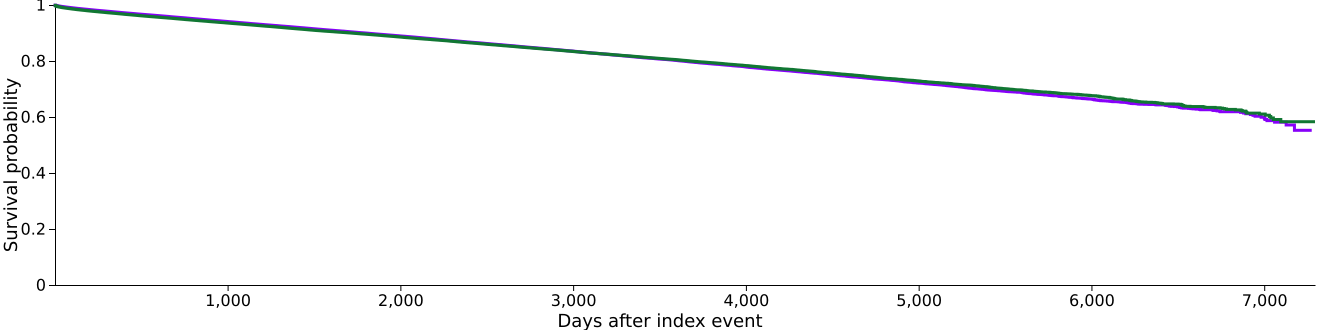 | | | | | | | |
|  | |  | | | 61,920 patients in Cohort 1 and 64,786 patients in Cohort 2 were excluded from results because they had the outcome prior to the time window. | | | | | | | |
|  | | Number of instances excluding patients with outcome prior to the time window | | | | | | | | | | |
|  | | |  | Cohort | | | Patients in cohort | Patients with outcome | Mean | Standard Deviation | Median | |
|  | | |  | 1 | | T2DM with ED | 493,588 | 44,683 | 5.543 | 11.258 | 2 | |
|  | | |  | 2 | | T2DM without ED | 490,722 | 41,161 | 5.220 | 11.969 | 2 | |
|  | | | | | | | | | | | | |
|  | | |  |  | | | t | df | p |  |  |  |
|  | | |  | Test Statistics | | | 4.079 | 85842 | 0.000 |  |  |  |
|  | | | | | | | | | | | | |
|  | |  | | | 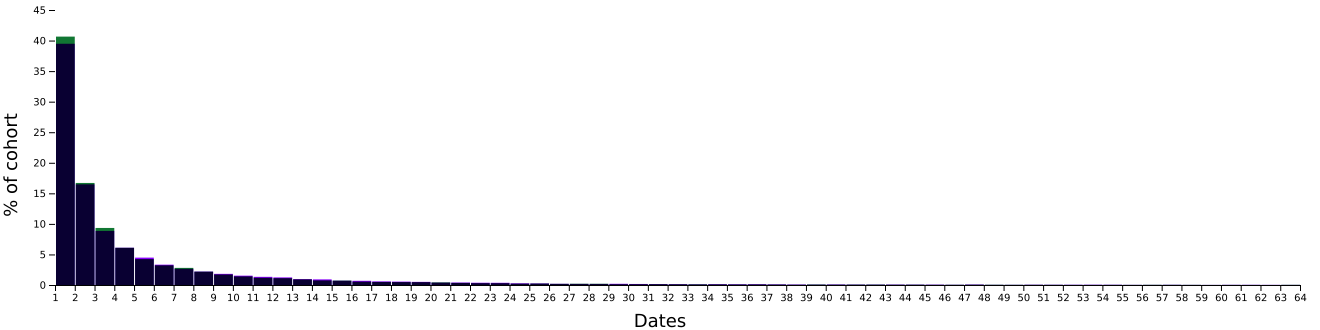 | | | | | | | |
|  | |  | | | 253 data points for Cohort 1 and 214 data points for Cohort 2 were omitted for display purposes. 61,920 patients in Cohort 1 and 64,786 patients in Cohort 2 were excluded from results because they had the outcome prior to the time window. | | | | | | | |
| 3 Heart failure | | | | | | | | | | | | |
|  | | Risk analysis excluding patients with outcome prior to the time window | | | | | | | | | | |
|  |  | | | Cohort | | | Patients in cohort | Patients with outcome | Risk | | | |
|  | | |  | 1 | | T2DM with ED | 484,863 | 49,149 | 0.101 | | | |
|  | | |  | 2 | | T2DM without ED | 482,451 | 48,350 | 0.100 | | | |
|  | | | | | | | | | | | | |
|  | | |  |  | | |  | 95% CI | z | p |  |  |
|  | | |  | Risk Difference | | | 0.001 | (-0.000, 0.002) | 1.877 | 0.060 |  |  |
|  | | |  | Risk Ratio | | | 1.011 | (0.999, 1.024) | N/A | N/A |  |  |
|  | | |  | Odds Ratio | | | 1.013 | (0.999, 1.026) | N/A | N/A |  |  |
|  | | | | | | | | | | | | |
|  | |  | | | 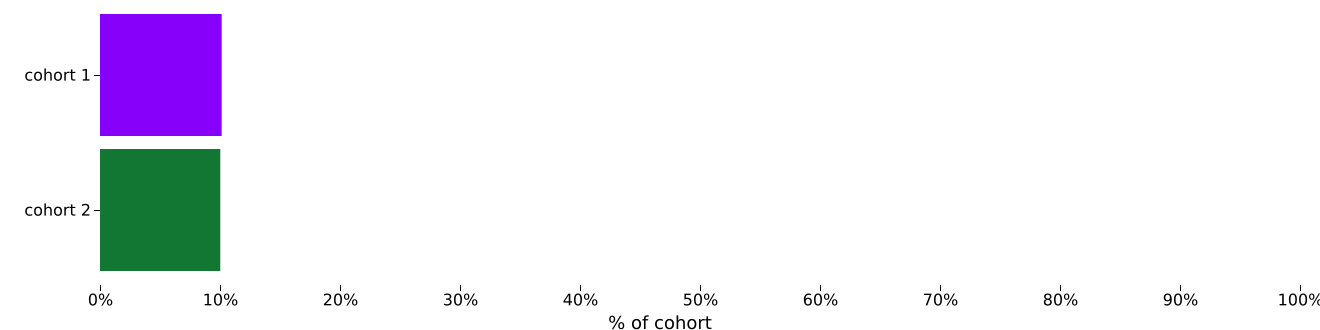 | | | | | | | |
|  | |  | | | 70,645 patients in Cohort 1 and 73,057 patients in Cohort 2 were excluded from results because they had the outcome prior to the time window. | | | | | | | |
|  | | Kaplan - Meier survival analysis excluding patients with outcome prior to the time window | | | | | | | | | | |
|  | | |  | Cohort | | | Patients in cohort | Patients with outcome | Median survival (days) | Survival probability at end of time window | | |
|  | | |  | 1 | | T2DM with ED | 484,863 | 49,149 | -- | 53.67% | | |
|  | | |  | 2 | | T2DM without ED | 482,451 | 48,350 | -- | 53.30% | | |
|  | | | | | | | | | | | | |
|  | | |  |  | | | χ^2^ | df | p |  |  |  |
|  | | |  | Log-Rank Test | | | 236.110 | 1 | 0.000 |  |  |  |
|  | | | | | | | | | | | | |
|  | | |  |  | | | Hazard Ratio | 95% CI | χ^2^ | df | p | |
|  | | |  | Hazard Ratio and Proportionality | | | 0.906 | (0.895, 0.918) | 96.648 | 1 | 0.000 | |
|  | | | | | | | | | | | | |
|  | |  | | | 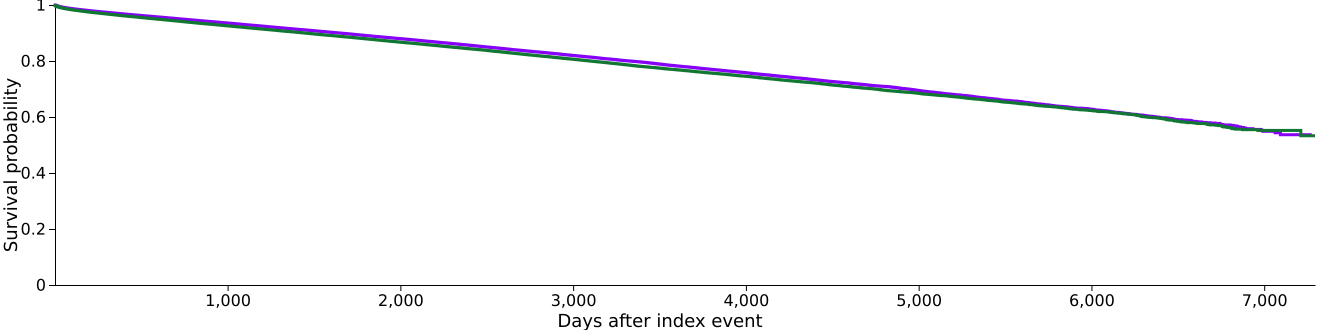 | | | | | | | |
|  | |  | | | 70,645 patients in Cohort 1 and 73,057 patients in Cohort 2 were excluded from results because they had the outcome prior to the time window. | | | | | | | |
|  | | Number of instances excluding patients with outcome prior to the time window | | | | | | | | | | |
|  | | |  | Cohort | | | Patients in cohort | Patients with outcome | Mean | Standard Deviation | Median | |
|  | | |  | 1 | | T2DM with ED | 484,863 | 49,149 | 9.446 | 17.905 | 3 | |
|  | | |  | 2 | | T2DM without ED | 482,451 | 48,350 | 8.422 | 15.300 | 3 | |
|  | | | | | | | | | | | | |
|  | | |  |  | | | t | df | p |  |  |  |
|  | | |  | Test Statistics | | | 9.601 | 97497 | 0.000 |  |  |  |
|  | | | | | | | | | | | | |
|  | |  | | | 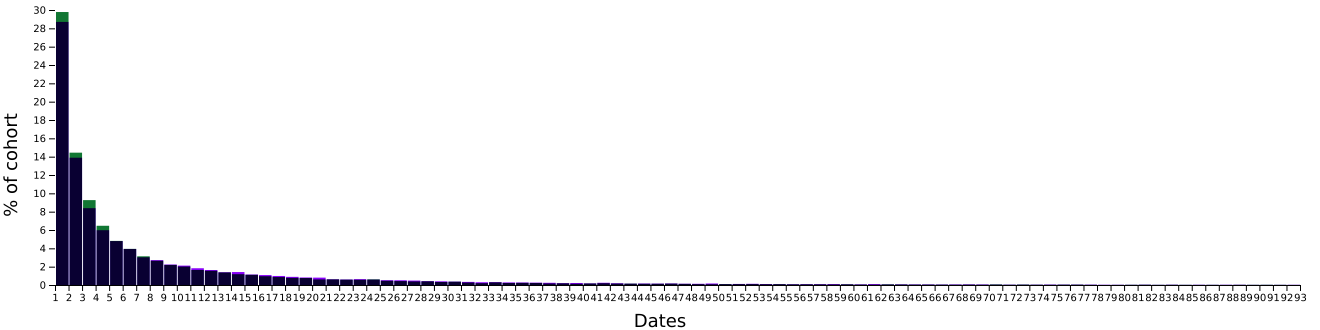 | | | | | | | |
|  | |  | | | 333 data points for Cohort 1 and 262 data points for Cohort 2 were omitted for display purposes. 70,645 patients in Cohort 1 and 73,057 patients in Cohort 2 were excluded from results because they had the outcome prior to the time window. | | | | | | | |
| 4 Peripheral artery disease | | | | | | | | | | | | |
|  | | Risk analysis excluding patients with outcome prior to the time window | | | | | | | | | | |
|  |  | | | Cohort | | | Patients in cohort | Patients with outcome | Risk | | | |
|  | | |  | 1 | | T2DM with ED | 497,508 | 43,283 | 0.087 | | | |
|  | | |  | 2 | | T2DM without ED | 507,938 | 38,386 | 0.076 | | | |
|  | | | | | | | | | | | | |
|  | | |  |  | | |  | 95% CI | z | p |  |  |
|  | | |  | Risk Difference | | | 0.011 | (0.010, 0.012) | 20.971 | 0.000 |  |  |
|  | | |  | Risk Ratio | | | 1.151 | (1.136, 1.166) | N/A | N/A |  |  |
|  | | |  | Odds Ratio | | | 1.166 | (1.149, 1.182) | N/A | N/A |  |  |
|  | | | | | | | | | | | | |
|  | |  | | | 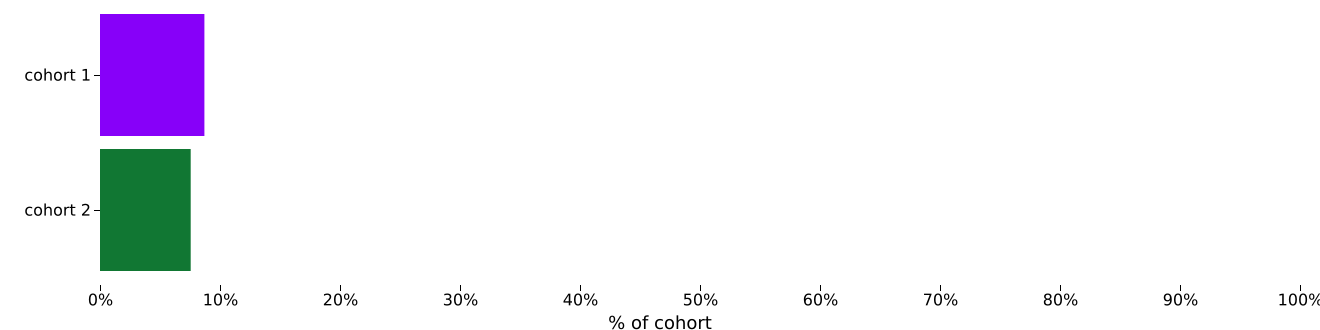 | | | | | | | |
|  | |  | | | 58,000 patients in Cohort 1 and 47,570 patients in Cohort 2 were excluded from results because they had the outcome prior to the time window. | | | | | | | |
|  | | Kaplan - Meier survival analysis excluding patients with outcome prior to the time window | | | | | | | | | | |
|  | | |  | Cohort | | | Patients in cohort | Patients with outcome | Median survival (days) | Survival probability at end of time window | | |
|  | | |  | 1 | | T2DM with ED | 497,508 | 43,283 | -- | 59.61% | | |
|  | | |  | 2 | | T2DM without ED | 507,938 | 38,386 | -- | 69.18% | | |
|  | | | | | | | | | | | | |
|  | | |  |  | | | χ^2^ | df | p |  |  |  |
|  | | |  | Log-Rank Test | | | 31.159 | 1 | 0.000 |  |  |  |
|  | | | | | | | | | | | | |
|  | | |  |  | | | Hazard Ratio | 95% CI | χ^2^ | df | p | |
|  | | |  | Hazard Ratio and Proportionality | | | 1.040 | (1.026, 1.054) | 38.841 | 1 | 0.000 | |
|  | | | | | | | | | | | | |
|  | |  | | | 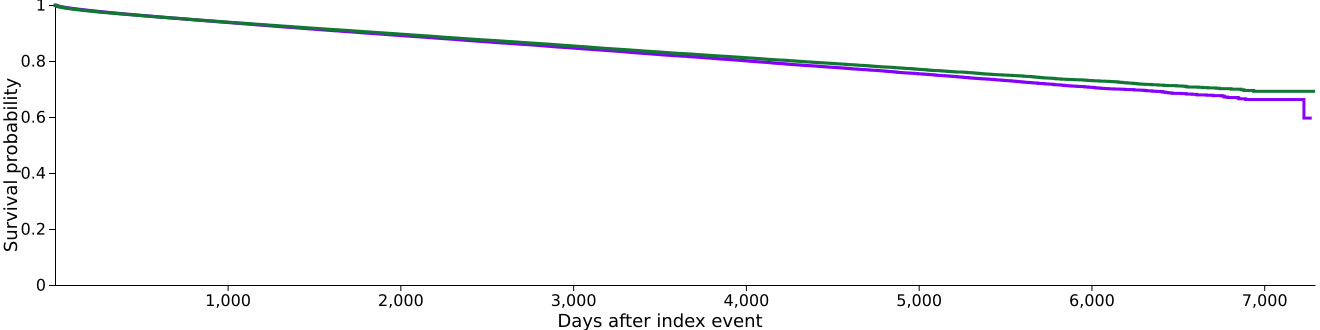 | | | | | | | |
|  | |  | | | 58,000 patients in Cohort 1 and 47,570 patients in Cohort 2 were excluded from results because they had the outcome prior to the time window. | | | | | | | |
|  | | Number of instances excluding patients with outcome prior to the time window | | | | | | | | | | |
|  | | |  | Cohort | | | Patients in cohort | Patients with outcome | Mean | Standard Deviation | Median | |
|  | | |  | 1 | | T2DM with ED | 497,508 | 43,283 | 4.687 | 12.226 | 2 | |
|  | | |  | 2 | | T2DM without ED | 507,938 | 38,386 | 4.707 | 12.321 | 2 | |
|  | | | | | | | | | | | | |
|  | | |  |  | | | t | df | p |  |  |  |
|  | | |  | Test Statistics | | | -0.234 | 81667 | 0.815 |  |  |  |
|  | | | | | | | | | | | | |
|  | |  | | | 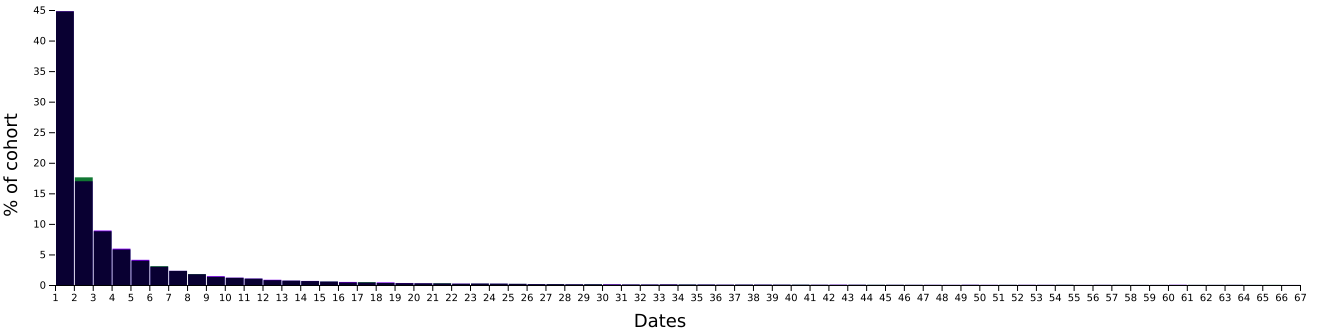 | | | | | | | |
|  | |  | | | 208 data points for Cohort 1 and 194 data points for Cohort 2 were omitted for display purposes. 58,000 patients in Cohort 1 and 47,570 patients in Cohort 2 were excluded from results because they had the outcome prior to the time window. | | | | | | | |
| 5 CVD | | | | | | | | | | | | |
|  | | Risk analysis excluding patients with outcome prior to the time window | | | | | | | | | | |
|  |  | | | Cohort | | | Patients in cohort | Patients with outcome | Risk | | | |
|  | | |  | 1 | | T2DM with ED | 321,207 | 82,829 | 0.258 | | | |
|  | | |  | 2 | | T2DM without ED | 311,504 | 73,660 | 0.236 | | | |
|  | | | | | | | | | | | | |
|  | | |  |  | | |  | 95% CI | z | p |  |  |
|  | | |  | Risk Difference | | | 0.021 | (0.019, 0.024) | 19.726 | 0.000 |  |  |
|  | | |  | Risk Ratio | | | 1.091 | (1.081, 1.100) | N/A | N/A |  |  |
|  | | |  | Odds Ratio | | | 1.122 | (1.109, 1.135) | N/A | N/A |  |  |
|  | | | | | | | | | | | | |
|  | |  | | | 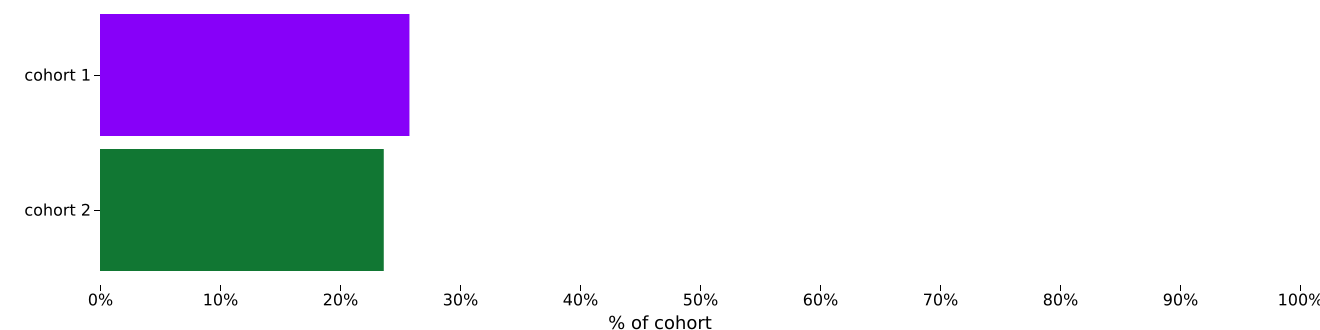 | | | | | | | |
|  | |  | | | 234,301 patients in Cohort 1 and 244,004 patients in Cohort 2 were excluded from results because they had the outcome prior to the time window. | | | | | | | |
|  | | Kaplan - Meier survival analysis excluding patients with outcome prior to the time window | | | | | | | | | | |
|  | | |  | Cohort | | | Patients in cohort | Patients with outcome | Median survival (days) | Survival probability at end of time window | | |
|  | | |  | 1 | | T2DM with ED | 321,207 | 82,829 | 3757 | 18.03% | | |
|  | | |  | 2 | | T2DM without ED | 311,504 | 73,660 | 3788 | 23.54% | | |
|  | | | | | | | | | | | | |
|  | | |  |  | | | χ^2^ | df | p |  |  |  |
|  | | |  | Log-Rank Test | | | 47.649 | 1 | 0.000 |  |  |  |
|  | | | | | | | | | | | | |
|  | | |  |  | | | Hazard Ratio | 95% CI | χ^2^ | df | p | |
|  | | |  | Hazard Ratio and Proportionality | | | 0.966 | (0.956, 0.975) | 160.987 | 1 | 0.000 | |
|  | | | | | | | | | | | | |
|  | |  | | | 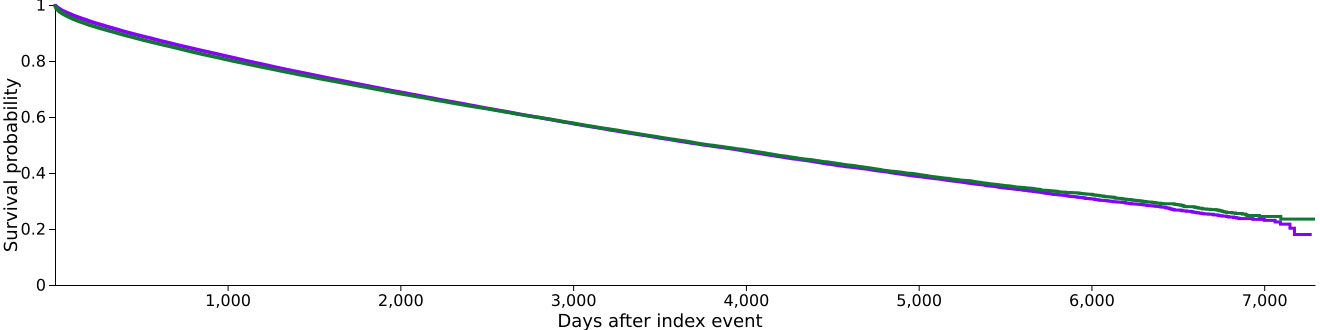 | | | | | | | |
|  | |  | | | 234,301 patients in Cohort 1 and 244,004 patients in Cohort 2 were excluded from results because they had the outcome prior to the time window. | | | | | | | |
|  | | Number of instances excluding patients with outcome prior to the time window | | | | | | | | | | |
|  | | |  | Cohort | | | Patients in cohort | Patients with outcome | Mean | Standard Deviation | Median | |
|  | | |  | 1 | | T2DM with ED | 321,207 | 82,829 | 9.571 | 18.322 | 3 | |
|  | | |  | 2 | | T2DM without ED | 311,504 | 73,660 | 8.616 | 17.401 | 3 | |
|  | | | | | | | | | | | | |
|  | | |  |  | | | t | df | p |  |  |  |
|  | | |  | Test Statistics | | | 10.536 | 156487 | 0.000 |  |  |  |
|  | | | | | | | | | | | | |
|  | |  | | | 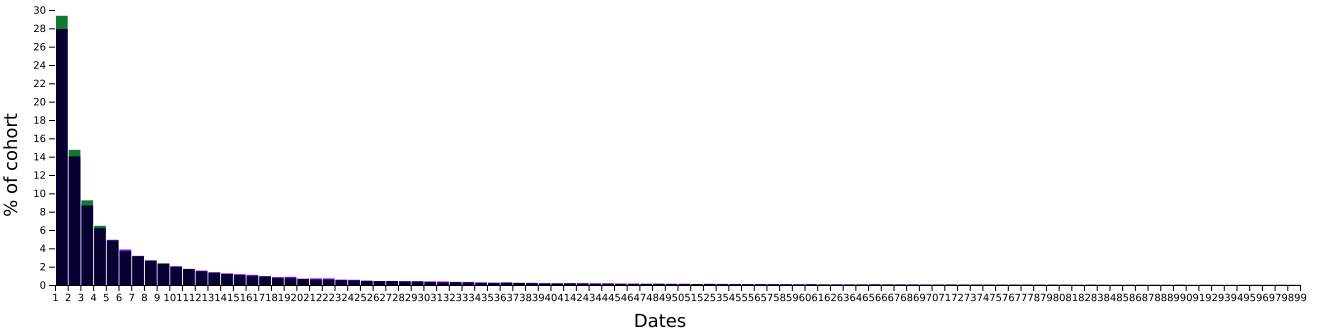 | | | | | | | |
|  | |  | | | 481 data points for Cohort 1 and 374 data points for Cohort 2 were omitted for display purposes. 234,301 patients in Cohort 1 and 244,004 patients in Cohort 2 were excluded from results because they had the outcome prior to the time window. | | | | | | | |

# Appendix A – Text Representation of the Cohorts Definition

This section lists all terms used in the definitions of the two cohorts.

### Query Criteria for Cohort 1 (query name: T2DM with ED)

Patients must have:
 all of the following:
 Age (Age) (at least 18 years (most recent occurrence)); and
 Male (UMLS:HL7V3.0:Gender:M); and
 Type 2 diabetes mellitus (UMLS:ICD10CM:E11) (between 20 and 80 years old at event); and
 any of the following:
 Male erectile dysfunction (UMLS:ICD10CM:N52); or
 Vasculogenic erectile dysfunction (UMLS:ICD10CM:N52.0); or
 Other male erectile dysfunction (UMLS:ICD10CM:N52.8); or
 Male erectile dysfunction, unspecified (UMLS:ICD10CM:N52.9); or
 Corporo-venous occlusive erectile dysfunction (UMLS:ICD10CM:N52.02); or
 Drugs used in erectile dysfunction (NLM:ATC:G04BE); or
 Vascular disorders of male genital organs (UMLS:ICD10CM:N50.1); or
 Erectile dysfunction due to diseases classified elsewhere (UMLS:ICD10CM:N52.1); or
 Erectile dysfunction due to arterial insufficiency (UMLS:ICD10CM:N52.01).

Patients cannot have:
 any of the following:
 Diabetes mellitus due to underlying condition (UMLS:ICD10CM:E08); or
 Drug or chemical induced diabetes mellitus (UMLS:ICD10CM:E09); or
 Type 1 diabetes mellitus (UMLS:ICD10CM:E10); or
 Other specified diabetes mellitus (UMLS:ICD10CM:E13).

### Query Criteria for Cohort 2 (query name: T2DM without ED)

Patients must have:
 all of the following:
 Age (Age) (at least 18 years (most recent occurrence)); and
 Male (UMLS:HL7V3.0:Gender:M); and
 Type 2 diabetes mellitus (UMLS:ICD10CM:E11) (between 20 and 80 years old at event).

Patients cannot have:
 any of the following:
 Diabetes mellitus due to underlying condition (UMLS:ICD10CM:E08); or
 Drug or chemical induced diabetes mellitus (UMLS:ICD10CM:E09); or
 Type 1 diabetes mellitus (UMLS:ICD10CM:E10); or
 Other specified diabetes mellitus (UMLS:ICD10CM:E13); or
 Male erectile dysfunction (UMLS:ICD10CM:N52); or
 Vasculogenic erectile dysfunction (UMLS:ICD10CM:N52.0); or
 Other male erectile dysfunction (UMLS:ICD10CM:N52.8); or
 Corporo-venous occlusive erectile dysfunction (UMLS:ICD10CM:N52.02); or
 Male erectile dysfunction, unspecified (UMLS:ICD10CM:N52.9); or
 Drugs used in erectile dysfunction (NLM:ATC:G04BE); or
 Erectile dysfunction due to arterial insufficiency (UMLS:ICD10CM:N52.01); or
 Vascular disorders of male genital organs (UMLS:ICD10CM:N50.1); or
 Erectile dysfunction due to diseases classified elsewhere (UMLS:ICD10CM:N52.1).

# Appendix B – Text Representation of the Analysis Setup

This section contains the Index Event definition for each cohort.

The index event for Cohort 1 (query name: T2DM with ED) is defined as the following:

Patients must have:
 all of the following:
 Type 2 diabetes mellitus (UMLS:ICD10CM:E11) (between 20 and 80 years old at event); and
 any of the following:
 Male erectile dysfunction (UMLS:ICD10CM:N52); or
 Vasculogenic erectile dysfunction (UMLS:ICD10CM:N52.0); or
 Other male erectile dysfunction (UMLS:ICD10CM:N52.8); or
 Male erectile dysfunction, unspecified (UMLS:ICD10CM:N52.9); or
 Corporo-venous occlusive erectile dysfunction (UMLS:ICD10CM:N52.02); or
 Drugs used in erectile dysfunction (NLM:ATC:G04BE); or
 Vascular disorders of male genital organs (UMLS:ICD10CM:N50.1); or
 Erectile dysfunction due to diseases classified elsewhere (UMLS:ICD10CM:N52.1); or
 Erectile dysfunction due to arterial insufficiency (UMLS:ICD10CM:N52.01).

The index event for Cohort 2 (query name: T2DM without ED) is defined as the following:

Patients must have:
 Type 2 diabetes mellitus (UMLS:ICD10CM:E11) (between 20 and 80 years old at event).

# Appendix C – Text Representation of the Outcomes Definition

This analysis includes the following outcomes:

Ischemic hearth disease
 Patients must have:
 Ischemic heart diseases (UMLS:ICD10CM:I20-I25).

Stroke
 Patients must have:
 Cerebrovascular diseases (UMLS:ICD10CM:I60-I69).

Heart failure
 Patients must have:
 Heart failure (UMLS:ICD10CM:I50).

Peripheral artery disease
 Patients must have:
 Diseases of veins, lymphatic vessels and lymph nodes, not elsewhere classified (UMLS:ICD10CM:I80-I89).

CVD
 Patients must have:
 any of the following:
 Ischemic heart diseases (UMLS:ICD10CM:I20-I25); or
 Cerebrovascular diseases (UMLS:ICD10CM:I60-I69); or
 Diseases of veins, lymphatic vessels and lymph nodes, not elsewhere classified (UMLS:ICD10CM:I80-I89); or
 Heart failure (UMLS:ICD10CM:I50).
