# Supplementary material for: Erectile dysfunction, type 2 diabetes, and cardiovascular disease: a narrative review and insights from a global real-world cohort analysis
Source: Front Clin Diabetes Healthc. 2026 Mar 6;7:1781581. doi: 10.3389/fcdhc.2026.1781581 (PMC13003461; doi:10.3389/fcdhc.2026.1781581)
Supplement: Supplementary file 2 [file DataSheet2.pdf]

Erectile dysfunction, type 2 diabetes, and cardiovascular disease: a narrative review and insights from a global real-world cohort analysis

Santiago Martínez Mores, Josep Franch, Didac Mauricio, Bogdan Vlacho

Title	Page
Supplementary figure 1. Shared pathophysiological mechanisms for erectile dysfunction, artery size hypothesis and cardiovascular disease	2
Supplementary Table S1. Summary table of the included articles	3
Appendix A: Analysis Report: Prevalence, incidence, risk factors and complications related to erectile dysfunction among people with type 2 diabetes mellitus Incidence and Prevalence	30
Appendix B: Propensity-score–matched comparative analysis for estimating the risk for different cardiovascular events among people with erectile dysfunction in the presence of type 2 diabetes and the absence of diabetes	34
Appendix C: Propensity-score–matched comparative analysis for estimating the risk for different cardiovascular events among people with type 2 diabetes regarding the presence or absence of erectile dysfunction	64

Supplementary figure 1. Shared pathophysiological mechanisms for erectile dysfunction, artery size hypothesis and cardiovascular disease

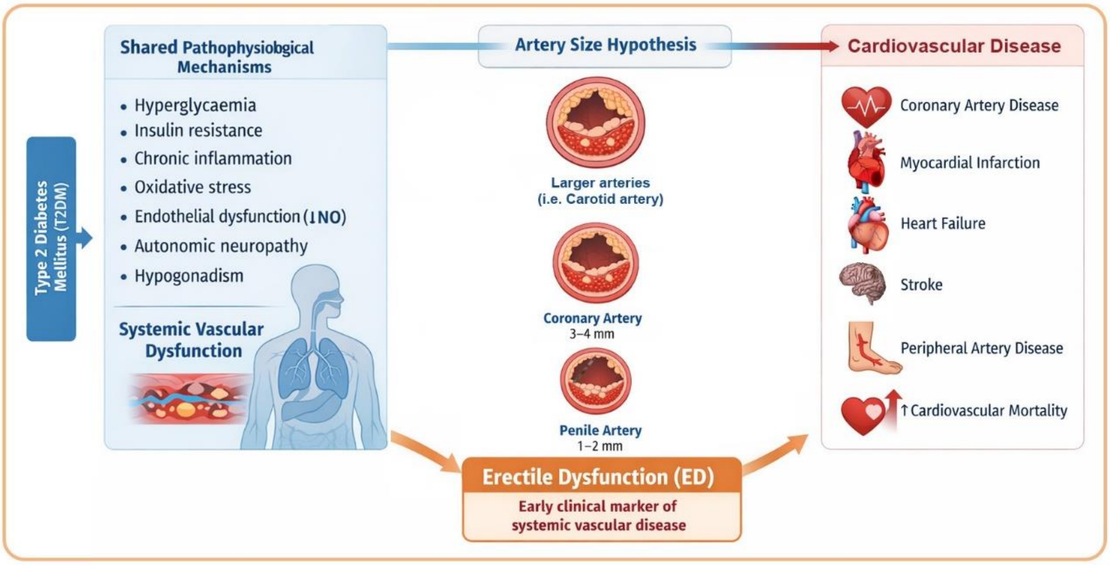

Artery size hypothesis. Penile arteries (1–2 mm) are two to four times smaller than coronary or carotid arteries (3–4 mm). For an equivalent atherosclerotic plaque burden, smaller vessels develop proportionally greater luminal obstruction, leading to earlier impairment of blood flow. Created with Adobe Illustrator by María Belén Martínez Mores and Santiago Martínez Mores (S.M.).

Supplementary Table S1. Summary table of the included articles

Number of the reference in the manuscript	Study identification	Study design	Participants	Interventions	Outcomes	Results	Quality/Risk of Bias
8	Jiaan, D B et al. (1995)	Experimental, cross sectional tissue study	Human penile tissue specimens from autopsy and living subjects (cadaveric and surgical samples)	Quantification of pentosidine (advanced glycation end product) in corpus cavernosum and tunica albuginea	AGE accumulation in penile tissue; mechanisms related to nitric oxide bioavailability	Pentosidine levels increased exponentially with age in penile tissues (≈4–6-fold from puberty to old age), indicating AGE-related collagen modification and impaired tissue turnover, providing a mechanistic link between aging, diabetes, and erectile dysfunction	Moderate
11	Seftel et al., 1997	Experimental, cross sectional human tissue study	Diabetic and nondiabetic men; human penile corpus cavernosum and tunica albuginea specimens	Quantification of AGEs (pentosidine, pyrraline) and assessment of NOS isoforms (iNOS, eNOS) in penile tissue; pharmacological iNOS inhibition	AGE accumulation, NOS expression, cavernosal smooth muscle relaxation	Diabetic penile tissue showed significantly higher AGE (pentosidine) levels localized to tunical and cavernosal collagen, with altered NOS expression (iNOS upregulation and reduced eNOS); selective iNOS inhibition significantly improved cavernosal relaxation, supporting an AGE-mediated mechanism of erectile dysfunction	Moderate
14	Hostnik et al., 2025	Narrative review	Men with diabetes mellitus and erectile dysfunction (literature-based)	Review of pathophysiological, genetic, and therapeutic studies	ED prevalence, mechanisms, genetic associations, and treatment response	ED prevalence was >3.5-fold higher in men with diabetes; diabetic ED was shown to be multifactorial, involving endothelial dysfunction, neuropathy, atherosclerosis, hormonal and structural changes; genetic polymorphisms were associated with ED risk and treatment response	Moderate

15	Feldman et al., 1994	Observational cohort study	Non-institutionalized men aged 40–70 years from the general population	Assessment of erectile function using a self-administered questionnaire; evaluation of medical, hormonal, and psychosocial factors	Prevalence of erectile dysfunction and associated medical and psychosocial correlates	Overall prevalence of erectile dysfunction was 52%, with complete ED increasing threefold from ages 40 to 70; ED was independently associated with diabetes, heart disease, hypertension, related medications, smoking, and depressive symptoms, supporting a strong link between ED and vascular risk factors	Low
16	Braun et al., 2000	Cross sectional survey	Representative sample of men aged 30–80 years from the Cologne urban district, Germany (n = 4,489 respondents)	Assessment of erectile function and sexual satisfaction using a validated self-administered questionnaire	Prevalence of erectile dysfunction and associated comorbidities	ED prevalence was 19.2%, with a marked age-related increase (2.3–53.4%); ED was frequently associated with hypertension, diabetes, pelvic surgery, and lower urinary tract symptoms, and was linked to significant dissatisfaction with sexual life	Moderate
17	Martin-Morales et al., 2001	Cross sectional survey	Non-institutionalized Spanish men aged 25–70 years (n = 2,476)	Assessment of erectile function using self-report and the International Index of Erectile Function; collection of medical, lifestyle, and medication data	Prevalence of erectile dysfunction and independent risk factors	ED prevalence ranged from 12.1% (self assessment) to 18.9% (IIEF); diabetes showed the strongest independent association with ED (age-adjusted OR= 4), followed by peripheral vascular disease (OR 2.63), hypertension (OR 1.58), hypercholesterolemia (OR 1.63), cardiac disease (OR 1.79), smoking (OR 2.5), and alcohol use (OR 1.53)	Moderate
19	Saigal et al., 2006	Cross sectional study (NHANES 2001–2002)	Non-institutionalized US men aged ≥20 years (n = 3,566)	Assessment of erectile dysfunction through standardized household interviews and medical history questionnaires	Prevalence of erectile dysfunction and independent risk factors	ED affected nearly 20% of adult men and 77.5% of those aged ≥75 years; diabetes was independently associated with ED (OR 2.69), obesity (OR 1.60), smoking (OR 1.74), and hypertension (OR 1.56); Hispanic ethnicity was associated with higher ED risk (OR 1.89)	Moderate

20	Corona et al., 2014	Multicenter prospective observational study: SUBITO-DE	Men with newly or recently diagnosed type 2 diabetes mellitus from 27 Italian diabetes centers (n = 499)	Clinical assessment of sexual function, hormonal profile, depressive symptoms, and cardiometabolic parameters	Prevalence and determinants of erectile dysfunction and other sexual dysfunctions at T2DM onset	ED was highly prevalent at T2DM diagnosis, with moderate-to-severe ED in 32% of patients; hypogonadism was present in 20%, and depressive symptoms and diabetes-related complications independently increased ED risk, highlighting early involvement of hormonal, psychological, and cardiovascular factors	Moderate
22	Guay, 2007	Narrative review	Men with erectile dysfunction and cardiovascular risk factors	Conceptual analysis of endothelial function linking erectile dysfunction and cardiovascular disease	Endothelial dysfunction as a shared mechanism between ED and CVD	The review proposed erectile dysfunction as a clinical manifestation of systemic endothelial dysfunction, highlighting that penile arteries, due to their smaller diameter, may become symptomatic earlier than coronary arteries, positioning ED as a potential early marker of cardiovascular disease and an opportunity for early cardiovascular risk assessment	Moderate
24	Liu et al., 2024	Mendelian randomisation study	Genome-wide association study (GWAS) data on inflammatory cytokines (n = 8,293) and erectile dysfunction cases and controls from FINNGEN R9 (2,205 ED cases; 164,104 controls)	Genetic instrumental variable analysis of 41 inflammatory cytokines using MR-Egger, IVW, and weighted median methods	Genetic causal associations between inflammatory cytokines and erectile dysfunction	Genetically predicted higher levels of IP-10 were associated with increased ED risk (OR 1.27, 95% CI 1.01-1.60), whereas higher IL-1RA levels were protective (OR 0.77, 95% CI 0.60-0.98); reverse MR analyses did not support causality from ED to cytokine levels	Moderate
25	Hegazy et al., 2020	Cross sectional clinical study	Controls (n=21), T2DM without CVD (n=30), and T2DM with macrovascular CVD (n=30)	Measurement of circulating soluble adhesion molecules (sVCAM-1, sICAM-1, sE-selectin) by ELISA	Endothelial activation markers and macrovascular complications in T2DM	Levels of sVCAM-1, sICAM-1, and sE-selectin were higher in T2DM patients than controls; sVCAM-1 was significantly higher in T2DM patients with CVD than those without CVD and showed positive correlations with other adhesion molecules, identifying sVCAM-1 as a marker of macrovascular disease in T2DM	Moderate

26	Isidori et al., 2014	Systematic review	Men with erectile dysfunction with or without hypogonadism (literature-based)	Systematic review of molecular studies, observational cohorts, and randomized controlled trials assessing testosterone and erectile function	Role of testosterone in erectile physiology and response to treatment	Testosterone was shown to modulate multiple components of erectile function, including endothelial cells, smooth muscle, neural pathways, and sexual desire; hypogonadism frequently coexisted with ED, and testosterone replacement therapy improved erectile function in appropriately selected hypogonadal men, particularly when used to restore physiological androgen levels rather than as routine combination therapy	Moderate
27	Dhindsa et al., 2004	Cross sectional clinical study	Men with type 2 diabetes mellitus (n = 103; mean age 54.7 years)	Measurement of total testosterone, free testosterone, SHBG, LH, FSH, and prolactin	Prevalence and type of hypogonadism in T2DM	Hypogonadism was present in 33% of men with T2DM and was predominantly hypogonadotropic, with significantly lower LH and FSH levels; free testosterone was inversely correlated with BMI, supporting a central, obesity-related mechanism	Moderate
28	Laaksonen et al., 2003	Cross sectional study	Non-diabetic middle-aged Finnish men from a community cohort (n = 1,896)	Measurement of total testosterone, calculated free testosterone, SHBG, and inflammatory markers	Association between sex hormones, metabolic syndrome, and inflammatory markers	Men with metabolic syndrome had significantly lower free testosterone (-11%) and SHBG (-18%); men in the lowest tertile of free testosterone had a higher likelihood of metabolic syndrome (OR 2.7, 95% CI 2.0-3.7; OR 1.7 after BMI adjustment), with inverse associations between sex hormones and insulin resistance, dyslipidemia, and inflammation	Moderate
29	Haffner et al., 1996	Prospective case control study	Initially nondiabetic men from the Multiple Risk Factor Intervention Trial (MRFIT) cohort (176 incident diabetes cases and matched controls)	Baseline measurement of total and free testosterone, SHBG, insulin, and glucose	Baseline measurement of total and free testosterone, SHBG, insulin, and glucose	Lower baseline levels of testosterone and SHBG were associated with a higher risk of developing diabetes over 5 years of follow up; these associations were attenuated after tight matching for BMI and fasting glucose, suggesting that low testosterone and SHBG are features of the prediabetic metabolic state rather than independent causal factors	Moderate

30	Saad, 2009	Narrative review	Men with type 2 diabetes mellitus, metabolic syndrome, or hypogonadism	Review of epidemiological and interventional studies evaluating testosterone and metabolic outcomes	Role of testosterone in glucose homeostasis, metabolic syndrome, and diabetes risk	Low testosterone and SHBG levels were consistently associated with insulin resistance, metabolic syndrome, and increased risk of type 2 diabetes; testosterone deficiency was proposed as a component of the metabolic syndrome, and testosterone replacement in hypogonadal men was associated with partial improvement of cardiometabolic risk factors, although long-term outcome data were limited	Moderate
31	Vikan et al., 2010	Prospective cohort study	Community dwelling men from the Tromsø Study (n = 1,454)	Baseline measurement of total testosterone, SHBG, and estradiol with longitudinal follow-up for incident diabetes	Incident type 2 diabetes mellitus	Higher total testosterone and SHBG levels were associated with a lower risk of incident diabetes (e.g., testosterone HR 0.71 per SD; SHBG HR 0.55 per SD), although these associations were attenuated after adjustment for waist circumference; higher estradiol levels independently predicted a higher risk of diabetes, even after accounting for obesity	Moderate
32	Simon et al., 1997	Case control study within a population cohort (Telecom Study)	Healthy adult men matched by age and ethnicity, stratified by total testosterone levels (n = 50)	Comparison of cardiometabolic risk factors according to plasma total testosterone and SHBG levels	Cardiovascular and metabolic risk factors associated with low testosterone	Men with low total testosterone had significantly higher BMI, waist/hip ratio, blood pressure, fasting and post load glucose, triglycerides, LDL cholesterol, and insulin levels, and lower HDL cholesterol and SHBG; associations with insulin and triglycerides persisted after adjustment for adiposity, linking low testosterone to insulin resistance and adverse cardiometabolic profile	Moderate
33	Andersson et al., 1994	Cross sectional clinical study	Men and women with non–insulin-dependent diabetes mellitus and non-diabetic controls	Measurement of total testosterone, free testosterone (calculated), SHBG, insulin, and anthropometric parameters	Associations between sex hormones, SHBG, insulin resistance, and NIDDM	Men with NIDDM had significantly lower total testosterone and SHBG levels than controls, with insulin concentrations inversely correlated with testosterone and SHBG; women with NIDDM showed lower SHBG and higher free testosterone, with insulin resistance closely associated with hyperandrogenicity and adiposity,	Moderate

						highlighting sex-specific hormonal patterns linked to insulin resistance	
34	Pitteloud et al., 2005	Mechanistic clinical study with metabolic clamp	Men with normal glucose tolerance, impaired glucose tolerance, and diabetes (n = 21; subset n = 18 for LH pulsatility)	Hyperinsulinemic euglycemic clamp, frequent LH sampling, GnRH stimulation, and hCG testing to assess pituitary and testicular function	Relationship between insulin resistance and hypothalamic pituitary gonadal axis function	Insulin sensitivity (M value) was positively associated with total testosterone levels (r = 0.46) and strongly correlated with testosterone response to hCG (r = 0.73), while LH secretion and pituitary responsiveness were preserved, indicating that insulin resistance is associated with reduced Leydig cell testosterone secretion rather than central hypogonadism	Moderate
35	Osuna et al., 2006	Cross sectional clinical study	Adult men stratified by BMI (normal weight, overweight, obese; n = 77)	Measurement of sex hormones (total and free testosterone, estradiol), SHBG, insulin, leptin, and insulin resistance (HOMA-IR)	Associations between obesity, insulin resistance, and sex hormone levels	Obese men had significantly lower total testosterone and SHBG and higher insulin levels than normal-weight and overweight men; total testosterone was inversely correlated with BMI, waist circumference, leptin, insulin, and HOMA-IR, supporting a close link between adiposity, insulin resistance, and androgen deficiency	Moderate
36	Rhoden et al., 2005	Cross sectional clinical study	Sexually active men with diabetes mellitus and erectile dysfunction (n = 115)	Assessment of erectile function using the IIEF questionnaire and measurement of fasting glucose and HbA1c levels	Association between glycaemic control and severity of erectile dysfunction	Higher HbA1c levels were associated with more severe ED: among men with HbA1c <8%, severe ED occurred in 32%, compared with 46% in those with HbA1c ≥8%; HbA1c ≥11% was associated with a significantly higher prevalence of severe ED, particularly in men with longer diabetes duration (>5 years), indicating a link between poor glycaemic control and ED severity	Moderate

37	Rhoden et al., 2005	Cross sectional study	Adult men with and without diabetes mellitus, age-matched (n = 746; 116 with DM)	Measurement of total and free testosterone, BMI and waist-to-hip ratio; stratified analysis by diabetes and adiposity	Association between diabetes, obesity, and subnormal testosterone levels	Subnormal free testosterone was present in 46% of diabetic men versus 24% of non-diabetics and was strongly associated with diabetes (OR 2.7; 95% CI 1.8 - 4.1), independent of BMI; in contrast, subnormal total testosterone was closely associated with obesity and central adiposity than with diabetes, highlighting distinct metabolic determinants of androgen deficiency	Moderate
38	Yagihashi et al., 2007	Narrative review	Patients with diabetes mellitus and impaired glucose tolerance (human pathological and clinical studies)	Histopathological evaluation of peripheral nerves and review of experimental and clinical data	Pathological features and pathogenetic mechanisms of diabetic neuropathy	Diabetic neuropathy is characterized by progressive, pan-modal nerve fiber loss with a distal predominance, accompanied by endoneurial microangiopathy that inversely correlates with nerve fiber density; small-caliber nerve fiber damage begins early, even at the stage of impaired glucose tolerance, and precedes overt nerve trunk involvement; multiple mechanisms, including glycation, polyol pathway activation, oxidative stress, and altered protein kinase C signaling, contribute to nerve injury	Low
39	Sima et al, 1999	Narrative review	Experimental animal models of type 1 and type 2 diabetes (mainly diabetic rodents)	Induction of diabetes and assessment of metabolic, vascular, and neural alterations	Pathogenetic mechanisms and structural functional features of diabetic neuropathy	Experimental studies demonstrate that diabetic neuropathy is a multifactorial disorder involving increased polyol pathway activity, non enzymatic glycation, oxidative stress, altered vasoactive signaling, and impaired neurotrophism; distinct functional and structural differences were identified between neuropathy in type 1 and type 2 diabetes, highlighting heterogeneity in underlying mechanisms	Low

40	Vernet et al., 1995	Experimental animal models study (rats)	Diabetic rat models of type 1 (BB/WORdp) and type 2 diabetes (BBZ/WORdp) and age matched non diabetic controls	Determinations of serum glucose, testosterone, and penile reflexes (cups and flips)	Penile nitric oxide synthase activity, erectile function, and hormonal status	Both type 1 and type 2 diabetic rats exhibited severe erectile dysfunction (>95% reduction in erectile reflexes), marked reductions in serum testosterone, and significant decreases in penile NOS activity (74% in type 1 and 55% in type 2 diabetes), with parallel reductions in nNOS protein, supporting a common NOS related mechanism of diabetic ED partly linked to androgen deficiency	Low
41	Fowler et al., 1988	Cross sectional clinical study	Men presenting with erectile dysfunction, including diabetic patients (n = 33)	Assessment of small-fiber neuropathy using thermal sensory threshold testing; electrophysiological testing of bulbocavernosus reflex	Presence of unmyelinated and small-fiber sensory neuropathy in diabetic erectile dysfunction	All men with clinically neuropathic erectile dysfunction showed abnormal thermal thresholds, indicating small-fiber neuropathy, whereas diabetics with non-neuropathic ED had normal results; standard electrophysiological testing failed to detect abnormalities in a substantial proportion of neuropathic cases, highlighting the sensitivity of small-fiber testing	Moderate
42	Hurt et al., 2002	Experimental study (animal models)	Mouse models, including eNOS knockout mice	Cavernous nerve stimulation, intracavernosal papaverine injection, pharmacological inhibition of PI3K/Akt pathway	Role of PI3K/Akt-mediated eNOS activation in penile erection	Cavernous nerve stimulation and papaverine rapidly increased Akt and eNOS phosphorylation in penile tissue; inhibition of PI3K/Akt signaling reduced eNOS activation and erectile responses, while eNOS-deficient mice showed markedly impaired erections, demonstrating that Akt-dependent eNOS activation is essential for sustained NO production and full erectile functio	Moderate
43	McCulloch et al., 1980	Cross sectional epidemiological study	Men with diabetes mellitus aged 20–59 years (n = 541)	Clinical survey assessing erectile function and diabetic complications	Prevalence of erectile dysfunction and associated clinical factors	Erectile dysfunction was present in 35% of diabetic men and was independently associated with age, diabetes treatment, retinopathy, symptomatic peripheral neuropathy, and autonomic neuropathy; the strongest associations were observed in patients with severe microangiopathy, particularly proliferative retinopathy and autonomic neuropathy, supporting a multifactorial etiology of diabetic ED	Moderate

44	Cho et al., 2006	Multicentre cross sectional study	Korean men with type 2 diabetes mellitus (n = 1312)	Assessment of erectile function using modified IIEF-5 and standardized face-to-face questionnaires	Prevalence and risk factors for erectile dysfunction	Overall prevalence of ED was 65.4%; age, longer diabetes duration, higher HbA1c, insulin use, neuropathy, and macrovascular complications were independently associated with ED, with the highest risk observed in men aged >60 years with diabetes duration >10 years.	Moderate
45	Zheng et al., 2006	Cross sectional clinical study	Cross-sectional clinical study	Face to face interviews, psychogenic assessment, penile hemodynamic testing, hormone measurements, and review of diabetic complications	Predictors of erectile dysfunction among diabetic men	Erectile dysfunction was diagnosed in 39.4% of participants and was independently associated with older age (OR 1.16), longer diabetes duration (OR 1.30), lower physical activity (OR 1.67), retinopathy (OR 1.15), neuropathy (OR 2.07), and depression (OR 1.46); men with ED exhibited more severe diabetic complications, supporting ED as a sentinel marker of underlying systemic disease	Moderate
46	Siu et al., 2001	Cross sectional clinical study	Chinese men with diabetes mellitus (n = 486)	Structured interviews assessing erectile dysfunction (NIH 1993 definition) and review of clinical records for diabetic complications	Prevalence, severity, and risk factors for erectile dysfunction	The prevalence of ED was 63.6%, increasing markedly with age and diabetes duration; ED prevalence rose from 56.0% in men with diabetes duration <5 years to 72.0% in those with >20 years, with severity also increasing over time; ED was independently associated with diabetes duration, retinopathy, albuminuria, sensory neuropathy, and higher educational level, while age was the main determinant of ED severity	Moderate
47	Klein et al., 1996	Population based cohort study (comparative study)	Men with long term insulin dependent diabetes mellitus (onset <30 years, duration ≥10 years; n = 365)	Self reported assessment of erectile dysfunction and evaluation of clinical and metabolic characteristics	Prevalence of erectile dysfunction and associated clinical factors	Erectile dysfunction was reported by 20% of participants and increased markedly with age and diabetes duration; ED was associated with severe retinopathy, peripheral neuropathy, cardiovascular disease, higher HbA1c, antihypertensive use, and higher BMI, indicating a strong link with cumulative microvascular and macrovascular damage	Moderate

48	Fedele et al., 1998	Multicentre cross-sectional study	Men with type 1 and type 2 diabetes mellitus aged 20 - 69 years, attending diabetes clinics in Italy (n = 9,868)	Structured interviews assessing erectile dysfunction and collection of clinical, metabolic, and lifestyle data	Prevalence of erectile dysfunction and associated risk factors	Erectile dysfunction was reported by 35.8% of diabetic men, with prevalence increasing markedly with age and diabetes duration; poorer glycaemic control, longer disease duration, microvascular complications (retinopathy, nephropathy, neuropathy), arterial disease, and smoking were independently associated with ED, while ED was less frequently reported in men with type 2 than type 1 diabetes after age adjustment	Moderate
49	Fukui et al., 2011	Cross-sectional clinical study	Men with type 2 diabetes mellitus (n = 197; vascular assessments in subsets of n = 125)	Assessment of erectile function using IIEF-5 and evaluation of albuminuria, pulse wave velocity, ankle-toe-brachial index, and diabetic complications	Association between erectile dysfunction, albuminuria, and subclinical atherosclerosis	Lower IIEF-5 scores were significantly associated with higher urinary albumin excretion, increased pulse wave velocity, and lower toe-brachial index; ED severity was greater in men with proliferative retinopathy, macroalbuminuria, neuropathy, or cardiovascular disease, supporting a close link between ED and both micro- and macrovascular disease	Moderate
50	Furukawa et al., 2017 (Dogo Study)	Multicentre cross-sectional study	Japanese men with type 2 diabetes mellitus aged 19- 65 years (n = 287)	Assessment of diabetic neuropathy using clinical criteria and erectile function using SHIM scores	Association between diabetic neuropathy and erectile dysfunction severity	Diabetic neuropathy was present in 47.0% of participants and severe ED in 39.0%; neuropathy was independently associated with severe ED, while no significant associations were observed with mild or moderate ED, nor with retinopathy or nephropathy, indicating a specific link between neuropathy and advanced erectile dysfunction	Moderate
51	Bandyk DF, 2018	Narrative review	Patients with diabetes mellitus, particularly those with diabetic foot complications	-	Pathophysiology of diabetic neuropathy, peripheral arterial disease, and critical limb ischemia	Diabetic neuropathy and peripheral arterial occlusive disease act synergistically, leading to micro- and macrovascular dysfunction, tissue ischemia, and impaired healing; highlights the systemic nature of diabetic neurovascular damage	Low-moderate

52	Coppola A et al., 2023	Retrospective cohort study	Men with type 2 diabetes and a recent, single diabetic foot ulcer. (n = 326)	Not applicable (observational study)	Wound healing, minor amputation, mortality, ulcer recurrence	ED prevalence was 56.1%. Wound healing was lower in men with ED vs no ED. Minor amputation and mortality were higher in the ED group. ED independently predicted mortality and ulcer recurrence, while absence of ED was associated with wound healing	Moderate
53	Henis O et al., 2011	Cross sectional study	Men with type 2 diabetes (n=102); mean age 64.0 ± 8.2 years; mean diabetes duration 14.5 ± 8.9 years	Not applicable (observational study)	Presence and severity of erectile dysfunction (SHIM score) according to diabetic retinopathy severity	ED prevalence was 73.5%. ED was significantly more frequent in men with severe NPDR or PDR compared with controls (87.5% vs 50.0%, p<0.0001). Mean SHIM score was lower in severe retinopathy. ED and SHIM score were independently associated with retinopathy severity after adjustment for age, diabetes duration, ischemic heart disease, cerebrovascular disease, hypertension, dyslipidemia, and smoking	Moderate
54	Chew SK et al., 2013	Cross sectional study (Hospital based)	Men with type 2 diabetes (n=324); mean age 65.2 ± 11.1 years	Comprehensive interview, a complete eye examination, fasting blood tests, and retinal and macula assessments using fundus images and optical coherence tomography	Presence of erectile dysfunction in relation to diabetic retinopathy (DR) and diabetic macular oedema (DME)	Compared with men without DR, those with any DR had a twofold higher odds of ED. Severe NPDR was associated with a fourfold increase in ED risk, and PDR with an approximately threefold increase. Diabetic macular oedema was not independently associated with ED, regardless of severity	Moderate
55	Katsimardou A et al., 2023	Cross sectional observational study	Patients with T2DM (n=80; 50 men, 30 women)	Comprehensive interviews with International Index of Erectile Function and the Female Sexual Function Index questionnaires for males and females, respectively, and patients were evaluated for DKD	Association between diabetic kidney disease (DKD), eGFR, albuminuria and sexual dysfunction (SD), including ED and FSD	Sexual dysfunction was present in 80% of participants; 45% had DKD. Reduced eGFR was significantly associated with SD, ED and FSD. In multivariable linear regression analyses, SD and ED were significant determinants of lower eGFR values. Albuminuria/proteinuria showed weaker and non-independent associations. In women, lower eGFR correlated with poorer desire, arousal, lubrication and total FSFI scores,	Moderate

						although these associations did not persist after multivariate adjustment	
56	Fu R et al., 2024	Crosssectional study (non interventional observational)	72 adult male patients with chronic kidney disease (CKD), China	Data collection included socio-demographic information, assessments via the 5-item version of the International Index of Erectile Function (IIEF-5), the Chinese version of the Premature Ejaculation Diagnostic Tool, the Patient Health Quentionnnaire-9 and the General Anxiety Disorder-7	Prevalence of erectile dysfunction (ED) and premature ejaculation (PE) and their association with kidney function and psychological factors	ED was present in 56.9% of CKD patients and PE in 29.2%. Sexual dysfunction was associated with reduced eGFR, higher albumin-to-creatinine ratio, psychological distress (depression and anxiety), and medication use. Findings support a multifactorial link between CKD severity and male sexual dysfunction	Moderate
57	Muniandy V et al., 2025	Cross sectional observational study	Men with type 2 diabetes mellitus and chronic kidney disease (n=280)	Questionnaires containing demographic and clinical information and the Malay Version of the Hospital Anxiety and Depression Scale (HADS). Sexual function was evaluated using the Malay Version of International Index of Erectile Function-5 (IIEF-5)	Prevalence of erectile dysfunction and associated clinical and metabolic determinants	ED prevalence was 95.0%. Independent factors associated with ED included metformin use, higher urea levels, higher HbA1c, while higher educational attainment was inversely associated with ED	Moderate
58	Dilixiati D et al., 2024	Systematic review and meta-analysis	58 studies; 66,925 men with diabetes mellitus (with and without erectile dysfunction)	Systematic review, encompassing studies published in the PubMed, Scopus and Embase databases up to August 24th, 2023	Identification of risk factors associated with erectile dysfunction in men with diabetes	ED risk was significantly associated with older age (OR 1.31), higher HbA1c (OR 1.44), longer diabetes duration (OR 1.39), diabetic neuropathy (OR 3.47), retinopathy (OR 3.01), diabetic foot (OR 3.96), cardiovascular disease (OR 1.92), hypertension (OR 1.74), microvascular disease (OR 2.14), vascular disease (OR 2.75), nephropathy (OR 2.67),	Low-moderate

						metabolic syndrome (OR 2.22), depression (OR 1.82), smoking (OR 1.32), and diuretic treatment (OR 2.42)	
59	Blair YA et al., 2024	Prospective cohort analysis within a randomized clinical trial (DPPOS)	568 men with prediabetes (PreD) or type 2 diabetes (T2D) from the Diabetes Prevention Program Outcomes Study	Prior randomization to intensive lifestyle intervention (ILS), metformin, or placebo (DPP); no ED-specific intervention	Prevalence of erectile dysfunction and identification of clinical, metabolic, and psychosocial predictors	ED prevalence was 38% overall, similar in PreD (41%) and T2D (37%). Age increased ED risk in all men. In PreD, ILS was associated with reduced odds of ED versus placebo (OR 0.35; 95% CI 0.13–0.94), whereas metformin showed no effect. In T2D, metabolic syndrome (OR 1.85; 95% CI 1.14–3.01) and depression (OR 2.05; 95% CI 1.10–3.79) were independently associated with ED	Low-moderate
60	Viigimaa M et al., 2020	Expert consensus, position paper	Men with arterial hypertension, with or without erectile dysfunction	Review of antihypertensive drug classes and their impact on erectile function; no experimental intervention	Association between arterial hypertension, erectile dysfunction, cardiovascular risk, and effects of antihypertensive treatment	ED frequently precedes overt coronary artery disease, supporting the artery size hypothesis. Hypertension and ED share common risk factors and mechanisms, including endothelial dysfunction and atherosclerosis. Treated hypertensive patients report ED more frequently than untreated ones. Antihypertensive drug classes show heterogeneous effects: diuretics and traditional beta-blockers are associated with worse erectile outcomes, whereas angiotensin receptor blockers and nebivolol show neutral or favorable profiles	Low
61	Vrentzos GE et al., 2007	Narrative review	Men with erectile dysfunction and cardiometabolic risk factors, including dyslipidemia	Review of lipid abnormalities, endothelial dysfunction, and pharmacological treatments (statins, PDE5 inhibitors)	Association between dyslipidemia and erectile dysfunction; endothelial and NO related mechanisms	Dyslipidemia is identified as an independent and contributory risk factor for ED. Elevated total and LDL cholesterol and reduced HDL cholesterol are associated with impaired erectile function. Oxidized LDL directly disrupts corpus cavernosum smooth muscle relaxation through endothelial dysfunction and reduced nitric oxide bioavailability. ED is	Moderate

						highlighted as an early marker of subclinical vascular disease. Statin therapy may improve erectile function and enhance responsiveness to PDE5 inhibitors by restoring endothelial function	
62	Bosevski M et al., 2019	Prospective cohort study	264 patients with type 2 diabetes and established coronary artery disease	Serial carotid ultrasound (CIMT, plaque detection, carotid stenosis) during follow-up	Cardiovascular events (new angina, myocardial infarction, stroke)	Progression of carotid artery disease was frequent (CIMT progression 86.8%, new plaques 41.8%). New carotid plaque formation was a strong independent predictor of total cardiovascular events (OR 13.5, 95% CI 1.68–108.4). CIMT progression showed modest association with new angina, while plaque progression carried greater prognostic value than CIMT alone	Moderate
63	Moon KH et al., 2019	Narrative review	Men with obesity and erectile dysfunction	Review of metabolic pathways (insulin resistance, leptin resistance, inflammation, oxidative stress) and combined therapeutic approaches (metformin, PDE5 inhibitors)	Association between obesity and erectile dysfunction; impact of metabolic modulation on erectile outcomes	Obesity and ED share a common pathophysiological background characterized by inflammation, oxidative stress, insulin and leptin resistance. Severity of ED correlates with obesity-related comorbidities. Metformin improves metabolic derangements, reduces oxidative stress and inflammation, and has been associated with improvements in erectile function in experimental and clinical studies. Combination therapy with metformin and PDE5 inhibitors improves erectile function in insulin resistant, sildenafil non responders	Moderate
64	Liu Y et al., 2023	Cross sectional observational study	878 men attending an andrology clinic in Central China	Erectile function was assessed by the International Index of Erectile Function (IIEF) scores. Questionnaires included questions about demographic characteristics (age, height, weight,	Presence and severity of erectile dysfunction assessed by IIEF	ED prevalence was 53.1%. BMI was higher in men with ED compared to non ED. Obesity was associated with increased ED risk. Obesity was strongly associated with moderate-severe ED, independent of demographic and lifestyle confounders	Moderate

				educational status), lifestyle habits (drinking, smoking, sleep time), and medical history.			
65	Wing RR et al., 2010	Randomized controlled tria	372 overweight/obese men with type 2 diabetes at baseline;	Intensive lifestyle intervention (ILI: weight loss + physical activity) vs diabetes support and education (DSE)	Change in erectile function assessed by IIEF-EF domain	ILI achieved greater weight loss (9.9% vs 0.6%) and fitness improvement (22.7% vs 4.6%). EF scores improved modestly in ILI compared with DSE (P = 0.04). EF category improved in 22% of ILI vs 23% of DSE, but worsening was significantly less frequent in ILI (8% vs 20%; P = 0.006), indicating preservation rather than recovery of erectile function	Low
66	Sattar N et al., 2023	Observational study	679,072 individuals with type 2 diabetes and 2,643,800 matched controls from the Swedish National Diabetes Register	Observational; longitudinal assessment of cardiometabolic risk factor control	Incident coronary artery disease, myocardial infarction, cerebrovascular disease, and heart failure	Marked decline in atherosclerotic events over time in T2DM, but heart failure incidence plateaued after 2013. Glycated hemoglobin and systolic blood pressure were most strongly associated with atherosclerotic events, while body mass index explained >30% of heart failure risk. Individuals with T2DM and all risk factors within target had no excess atherosclerotic risk compared with controls, except for heart failure (HR 1.50, 95% CI 1.35- 1.67)	Low
67	Mosenzon O et al., 2021	Cross sectional study	9,823 adults with type 2 diabetes from 13 countries across five continents	Observational; standardized assessment of cardiovascular disease prevalence and treatment patterns	Prevalence of established cardiovascular disease and atherosclerotic cardiovascular disease	Overall weighted prevalence of CVD was 34.8% (95% CI 32.7–36.8) and of atherosclerotic CVD 31.8% (95% CI 29.7–33.8). Median diabetes duration was 10.7 years and median HbA1c 7.3%. Use of glucose-lowering agents with proven cardiovascular benefit was low (21.9%) and similar in patients with and without established CVD	Low

68	Montorsi P et al., 2005	Narrative review	Men with erectile dysfunction and/or coronary artery disease, based on clinical and epidemiological evidence	Not aplicable	Conceptual framework linking erectile dysfunction and coronary artery disease	Proposes the “artery size hypothesis”, which states that atherosclerosis affects all vascular beds similarly, but symptoms appear earlier in smaller arteries. Because penile arteries are smaller than coronary arteries, erectile dysfunction often precedes clinical manifestations of coronary artery disease, whereas patients with established CAD frequently report ED	Low
69	Montorsi P et al., 2006	Observational, cross sectional study	285 men with angiographically proven coronary artery disease (acute coronary syndrome or chronic coronary syndrome) and 95 control subjects with normal coronary arteries	Diagnostic coronary angiography with quantitative assessment (vessel involvement and Gensini score), erectile function evaluation using the International Index of Erectile Function (IIEF-EFD), and laboratory assessment of cardiovascular and hormonal risk factors	Prevalence of erectile dysfunction according to coronary clinical presentation (ACS vs CCS) and extent of coronary artery disease (single- vs multi-vessel disease)	Erectile dysfunction prevalence increased with greater atherosclerotic burden and chronic coronary syndrome. ED prevalence was 22% in ACS with single-vessel disease, 55% in ACS with multi-vessel disease, and 65% in chronic coronary syndrome. In patients with chronic coronary syndrome and ED, erectile dysfunction preceded coronary artery disease in 93% of cases by a mean of 24 months. Multivessel disease and chronic coronary syndrome were independent predictors of ED	Moderate
70	Akili H et al., 2007	Cross sectional observational study	105 men with chest pain, positive exercise treadmill test, and no previous diagnosis of coronary artery disease	Exercise treadmill testing followed by diagnostic coronary angiography, and assessment of erectile function using the Sexual Health Inventory for Men (SHIM).	Significance of erectile dysfunction in males with a positive exercise treadmill test (ETT) to predict the severity of coronary artery disease (CAD)	The SHIM score is an independent parameter to define the presence of significant lesions in two or more coronary arteries (odds ratio, 0.84; 95% CI, 0.73-0.97; P = 0.019).	Moderate
71	Zhao B et al., 2019	Systematic review and metaanalysis	25 studies including 154,794 men from cohort and observational studies	Literature review and meta-analysis	Cardiovascular disease (CVD), coronary heart disease (CHD), stroke, all-cause mortality	Erectile dysfunction was associated with a significantly increased risk of CVD (RR 1.43), CHD (RR 1.59), stroke (RR 1.34), and all-cause mortality (RR 1.33), all p<0.001. Risk was higher in men ≥55 years, with shorter ED duration (<7 years), higher prevalence of diabetes (≥20%) and smoking (≥40%). Severe	Low

						ED predicted the highest risk of CVD and mortality	
72	Fang SC et al., 2015	Longitudinal population-based cohort study	965 men free of cardiovascular disease at baseline	ED was assessed with the five-item International Index of Erectile Function and classified as no ED/transient ED/persistent ED. CVD risk was assessed with 10-year Framingham CVD risk algorithm.	Framingham 10-year cardiovascular risk and change in Framingham risk over time	Transient and persistent erectile dysfunction were associated with higher Framingham cardiovascular risk and greater increase in risk over time, independent of baseline risk and other covariates. Persistent ED was associated with a higher Framingham risk particularly in men <50 years. Associations were stronger in younger men	Low
73	Gazzaruso C et al., 2011	Cross sectional study	293 men with newly diagnosed type 2 diabetes and no apparent vascular complications	Type 2 diabetes mellitus were evaluated to find patients with asymptomatic CAD and to assess the presence of diabetic complications with exercise stress testing. The presence and the degree of ED were assessed by the validated International Index Erectile Function-5 (IIEF-5) questionnaire	Presence of asymptomatic coronary artery disease (angiographically proven)	Erectile dysfunction was significantly more prevalent in men with asymptomatic CAD than in those without CAD (37.8% vs 15.1%). ED independently predicted silent CAD (OR 4.4; 95% CI 2.1–9.0). Adding ED to current screening risk factors increased sensitivity from 62% to 89%, improved negative predictive value from 82% to 94%, and reduced missed silent CAD cases from 37.8% to 10.8%, with minimal loss of specificity	Moderate
74	Turek SJ et al., 2013	Cross sectional analysis within a longitudinal cohort	301 men with ≥50 years of type 1 diabetes (Joslin Medalist Study	Self assessment of SD in males of the Medalist cohort by self-reported sexual problems with CVD. Sexual dysfunction is validated through the use of the abbreviated International Index of Erectile Dysfunction (IIEF).	Presence of cardiovascular disease	Sexual dysfunction was reported by 69.8% of participants. SD was independently associated with cardiovascular disease after adjustment for age, HbA1c, and BMI (OR 1.9; 95% CI 1.0–3.5). SD was associated with adverse cardiometabolic and inflammatory profiles (higher HbA1c, BMI, total cholesterol, IL-6; lower HDL). Retinal, neural, and renal microvascular complications were not associated with SD	Moderate

75	Nehra A et al., 2012	Expert consensus / clinical guideline	Men with erectile dysfunction with or without known cardiovascular disease	Cardiovascular risk stratification, exercise capacity assessment, and guideline-based management of ED	Identification of occult CVD; cardiovascular risk associated with sexual activity	The consensus highlights ED as an early clinical marker of cardiovascular disease, recommending systematic cardiovascular risk assessment in men with ED, including stress testing when indicated, to guide both sexual and cardiovascular management	Low
76	Hippisley-Cox J et al., 2017	Prospective open cohort study	Adults aged 25–84 years without baseline CVD from UK primary care (derivation cohort: 7.89 million; validation cohort: 2.67 million)	Development and validation of the QRISK3 cardiovascular risk prediction model, including erectile dysfunction diagnosis or treatment as a candidate risk factor in men	Incident cardiovascular disease (coronary heart disease and stroke)	Erectile dysfunction met inclusion criteria and was incorporated as an independent variable in the QRISK3 algorithm, which demonstrated good calibration and discrimination for 10-year CVD risk prediction	Low
77	Gazzaruso et al., 2008	Prospective cohort study	Men with type 2 diabetes and angiographically proven asymptomatic CAD (n = 291)	Erectile dysfunction assessed by IIEF-5; use of statins and PDE-5 inhibitors	Major adverse cardiovascular events (MACE) and all-cause mortality	ED prevalence was higher among patients with MACE (61.2% vs 36.4%; $p = 0.001$), and ED independently predicted MACE (HR 2.1; 95% CI 1.6–2.6; $p < 0.001$); statin use was associated with reduced MACE risk (HR 0.66; 95% CI 0.46–0.97), while PDE-5 inhibitor use showed a borderline protective association	Moderate
78	Batty et al., 2010	Prospective cohort study (post-hoc analysis of RCT cohort)	Men with type 2 diabetes aged 55–88 years from the ADVANCE trial (n = 6,304)	Erectile dysfunction assessed by self-report at baseline and follow-up	Incident cardiovascular disease (overall CVD, CHD, cerebrovascular events)	Baseline ED was independently associated with higher risk of all CVD events (HR 1.19; 95% CI 1.08–1.32), coronary heart disease (HR 1.35; 95% CI 1.16–1.56), and cerebrovascular disease (HR 1.36; 95% CI 1.11–1.67); persistent ED conferred the highest risk	Low

79	Grant et al., 2013	Narrative review	Men with erectile dysfunction	Clinical assessment of ED and associated cardiometabolic risk factors	Cardiovascular disease, diabetes mellitus, depression	ED is recognised as an early clinical marker of CVD and diabetes, sharing major risk factors with cardiometabolic disease and frequently under-recognised in routine clinical practice	Moderate
80	Zhang et al., 2025	Mendelian randomization study	Genetic datasets for erectile dysfunction and myocardial infarction derived from large genome-wide association data	Genetic instruments for ED and MI; multivariable and mediation Mendelian randomization analyses	Myocardial infarction	Genetically predicted ED was associated with an increased risk of MI, with consistent findings in validation datasets and no evidence of horizontal pleiotropy; smoking showed partial mediation	Low
81	Hermans et al., 2009	Crosssectional observational study	Men with type 2 diabetes mellitus attending outpatient clinics (n = 221)	Erectile dysfunction assessed using IIEF-5; comparison between ED(+) and ED(–) groups	Microangiopathy (retinopathy, neuropathy, albuminuria), metabolic syndrome, macrovascular disease, UKPDS CV risk	ED was associated with a higher prevalence of metabolic syndrome, central adiposity, and microangiopathy (retinopathy, neuropathy, albuminuria), as well as increased peripheral vascular and cerebrovascular events, despite similar glycaemic control and UKPDS 10-year CV risk	Moderate
82	Birgün et al., 2025	Narrative review	Patients with diabetes mellitus and sexual dysfunction and/or peripheral artery disease	Review of vascular and neural mechanisms linking sexual dysfunction and PAD in diabetes	Peripheral artery disease, sexual dysfunction, cardiovascular risk	Sexual dysfunction and peripheral artery disease share common vascular and neural mechanisms in diabetes, including endothelial dysfunction, atherosclerosis, and diabetic neuropathy, supporting sexual dysfunction as an early marker of systemic vascular impairment and increased cardiovascular risk	Moderate

83	Wang & Ni, 2024	Cross sectional study	Adult men from NHANES (n = 2,394), aged ≥40 years	Erectile dysfunction assessed by self-reported erection problems; PAD defined by ABI <0.9	Peripheral arterial disease	ED was independently associated with a higher prevalence of PAD after multivariable adjustment (OR 2.05; 95% CI 1.24–3.39), with consistent associations across multiple subgroups	Moderate
84	Polonsky et al., 2009	Prospective observational screening study	Men referred for stress testing without known PAD (n = 690)	ED assessed by IIEF; PAD screened using ankle–brachial index (ABI)	Peripheral arterial disease	ED was independently associated with PAD (OR 1.97; 95% CI 1.32–2.94), with a higher prevalence of PAD in men with ED compared with those without ED (32% vs 16%) and a stepwise increase in PAD prevalence with increasing ED severity	Moderate
85	Yuan et al., 2020	Cross sectional observational study	Chinese men with type 2 diabetes mellitus without clinical CVD (n =71)	Erectile function assessed by IIEF-5; carotid and lower limb Doppler ultrasound	Subclinical atherosclerosis (carotid IMT, peripheral plaques) and ED	ED prevalence was 71.2%; men with ED had higher carotid IMT and more frequent lower limb plaques; carotid IMT >0.75 mm and peripheral plaques remained independently associated with ED severity after adjustment	Moderate
86	Schwarz et al., 2006	Narrative review	Men with chronic heart failure	Clinical assessment of ED in the context of HF and standard HF therapies	Erectile dysfunction prevalence and contributing factors in HF	ED is highly prevalent in men with heart failure and reflects shared cardiometabolic risk factors as well as HF specific physiological and psychological mechanisms; standard HF therapies (e.g., beta blockers, thiazide diuretics) may further worsen sexual function	Moderate

87	Carella et al., 2023	Narrative review	Narrative review	Clinical assessment and management of ED in HF, including pharmacological and non-pharmacological strategies	Erectile dysfunction prevalence, cardiovascular events, mortality	ED is a frequent complication of heart failure and may act as a predictor of cardiovascular events and mortality; its pathophysiology is multifactorial, and individualized management may improve sexual function and cardiopulmonary parameters	Moderate
88	Ma et al.,2008	Prospective cohort study	Chinese men with T2DM and no clinically overt CVD at baseline (n = 2,306)	Presence of erectile dysfunction at baseline, defined by NIH criteria	Incident coronary heart disease (CHD) events	ED was present in 26.7% of participants and was associated with a higher incidence of CHD events (19.7 vs 9.5 per 1,000 person years); ED remained an independent predictor of CHD after multivariable adjustment (HR 1.58; 95% CI 1.08 - 2.30)	Low
89	Seidu et al., 2022	Systematic review and meta analysis	Adults with erectile dysfunction, with and without diabetes mellitus (18 studies)	Presence of ED; use of phosphodiesterase-5 inhibitors (PDE5i)	Cardiovascular disease, MACE, CHD, stroke, all cause mortality	ED was associated with increased risk of CVD and mortality in both the general population and people with diabetes (RRs 1.3 - 1.7); in diabetes, ED was associated with higher risks of composite CVD/MACE and CHD; limited RCT data showed no significant reduction in cardiovascular outcomes with PDE5i use	Low
90	Khatana et al., 2008	Prospective interventional study	Men with type 2 diabetes mellitus (veterans; n = 41)	Short term behavioral and pharmacologic cardiovascular risk reduction program	Change in erectile function (IIEF-5 score)	Improvements in blood pressure and glycaemic control were significantly associated with improvements in erectile function; changes in systolic and diastolic blood pressure and maintenance of HbA1c <7% correlated with IIEF-5 improvement	Moderate

91	Fu et al., 2024	Narrative review	Individuals with type 2 diabetes and non diabetic populations with cardiovascular and renal disease	Use of SGLT2 inhibitors, GLP-1 receptor agonists, and DPP-4 inhibitors	Cardiovascular and renal outcomes; cardiorenal protection mechanisms	Emerging antidiabetic drugs (SGLT2i, GLP-1RAs, DPP-4i) demonstrate consistent cardiorenal protective effects through glucose dependent and independent mechanisms, improving cardiovascular and renal outcomes beyond glycaemic control	Moderate
92	Salvatore et al., 2022	Narrative review	Individuals with type 2 diabetes and patients with cardiovascular and renal disease	Use of SGLT2 inhibitors	Cardiovascular and renal outcomes; mechanisms of cardiorenal protection	SGLT2 inhibitors exert cardiorenal protective effects beyond glycaemic control through haemodynamic changes (diuresis, natriuresis), metabolic shifts in myocardial substrate use, improved vascular function, and antifibrotic, anti inflammatory, and antioxidative mechanisms	Moderate
93	Yang et al., 2025	Systematic review and meta analysis	Adults with diabetes mellitus and erectile dysfunction (3 clinical studies included)	Use of antidiabetic drugs (GLP-1RAs, metformin, thiazolidinediones, SGLT2i, insulin, sulfonylureas)	Change in erectile function	GLP-1 receptor agonists showed greater improvement in erectile function compared with metformin (Z = 2.41; *P* = 0.02), with a more pronounced effect in overweight or obese patients; other antidiabetic drug classes showed potential benefits, but evidence was limited and heterogeneous	Moderate
94	Endocrine Practice, 2024 (TriNetX cohort)	Retrospective cohort study (real-world data)	Male patients with type 2 diabetes mellitus (SGLT2i users n=322,000; non users n=1,280,000)	Use of SGLT2 inhibitors vs non-use (propensity score matched)	Incident erectile dysfunction; use of phosphodiesterase-5 inhibitors	SGLT2 inhibitor use was associated with a higher incidence of ED diagnoses (RR 1.51; *P* < 0.001) and greater use of PDE5 inhibitors (RR 1.57; *P* < 0.001) compared with non users	Moderate

95	Kounatidis et al., 2025	Narrative review	Narrative review	Use of glucagon-like peptide-1 receptor agonists (GLP-1 RAs)	Erectile function; mechanistic links with microvascular disease and metabolic control	GLP-1 RAs may improve erectile function through weight loss, improved glycaemic control, endothelial function, and androgen profile; however, available evidence is heterogeneous and preliminary signals of a possible association with ED have also been reported, precluding definitive conclusions	Moderate
96	Bajaj et al., 202	Double-blind, placebo-controlled randomized trial	Men with type 2 diabetes mellitus participating in REWIND (n = 3,725)	Dulaglutide vs placebo	Incident moderate or severe erectile dysfunction; change in erectile function score (IIEF)	Dulaglutide was associated with a lower incidence of moderate or severe ED compared with placebo (21.3 vs 22.0 per 100 person years; HR 0.92, 95% CI 0.85 - 0.99) and a smaller decline in erectile function scores over time	Low
97	Giagulli VA et al., 2015	Retrospective observational study	Obese men with type 2 diabetes, overt hypogonadism, and erectile dysfunction (n = 43)	Addition of liraglutide to lifestyle intervention, metformin, and testosterone replacement therapy	Change in erectile function (IIEF score); metabolic and hormonal parameters	In men with inadequate metabolic control on testosterone plus metformin, the addition of liraglutide was associated with improved glycaemic control, weight loss, increased testosterone and SHBG levels, and a significant further improvement in erectile function scores; no further ED improvement was observed in patients who did not receive liraglutide	Moderate
98	An et al., 2024	Mendelian randomization study	Genetic datasets representing individuals with type 2 diabetes, obesity, hypertension, and cardiovascular disease	Genetically proxied exposure to GLP-1 receptor agonists	Erectile dysfunction	Genetically proxied GLP-1RA exposure was associated with a lower risk of ED (OR 0.49; 95% CI 0.43- 0.57); mediation analyses suggested that a small proportion of this effect was mediated through reductions in T2D, obesity, hypertension, and CVD	Moderate

99	Pourabhari Langroudi et al., 2025	Cross sectional study	Male patients reported in FAERS receiving GLP-1 receptor agonists	Exposure to GLP-1 receptor agonists (tirzepatide, semaglutide, dulaglutide, exenatide, lixisenatide, liraglutide)	Reports of male sexual dysfunction (erectile dysfunction, orgasmic dysfunction, reduced libido)	Among 182 reported cases, disproportionality analyses showed statistically significant signals but low effect sizes (ROR =0.41), indicating a weak association between GLP-1RA use and male sexual dysfunction; overall clinical risk appeared low	High
100	UKPDS Group, 1998	Randomized controlled trial	Overweight patients with newly diagnosed type 2 diabetes mellitus (n = 753 in metformin RCT)	Intensive glucose control with metformin vs conventional therapy (diet) or other glucose-lowering agents	Diabetes related clinical endpoints, cardiovascular outcomes, all-cause mortality	Intensive treatment with metformin significantly reduced diabetes related endpoints (32%), diabetes related mortality (42%), and all cause mortality (36%) compared with conventional therapy, and showed superior outcomes compared with sulphonylureas or insulin	Low
101	Shah et al., 2023	Narrative review	Patients with type 2 diabetes mellitus and chronic kidney disease, with or without cardiovascular disease	Use of finerenone	Cardiovascular and renal outcomes	Evidence from large clinical trials (FIDELIO-DKD, FIGARO-DKD) shows that finerenone reduces cardiovascular events and slows kidney disease progression in patients with T2DM and CKD, supporting a role in cardiorenal protection beyond glycaemic control	Moderate
102	Doumas & Douma, 2006	Narrative review	Men with hypertension, with or without erectile dysfunction	Antihypertensive drug classes (beta-blockers, diuretics, ACE inhibitors, calcium channel blockers, ARBs); use of PDE5 inhibitors	Erectile function; treatment adherence	Older generation antihypertensive drugs (beta blockers, diuretics, centrally acting agents) were associated with worsening erectile function, whereas newer agents (ACE inhibitors, calcium channel blockers) appeared neutral and angiotensin receptor blockers showed potential beneficial effects; PDE5 inhibitors were considered safe and effective in hypertensive patients	Moderate

103	Dash et al., 2024	Systematic review	Hypertensive men with erectile dysfunction (12 studies; n = 11,672)	Angiotensin receptor blockers compared with other antihypertensive drug classes	Erectile function, sexual activity, treatment satisfaction	Across included studies, ARBs were associated with significant improvements in erectile function, frequency of sexual activity, and overall sexual satisfaction compared with older-generation antihypertensive agents, while effectively reducing blood pressure	Moderate
104	Ismail et al., 2019	Meta analysis of randomized controlled trials	Hypertensive men, with or without diabetes (4 RCTs; n = 2,809)	Angiotensin receptor blockers (valsartan, losartan, telmisartan) vs conventional therapy or placebo	Erectile function and sexual activity	ARBs were associated with improved sexual activity, particularly with valsartan (MD 0.71; 95% CI 0.66 - 0.76); however, improvements in erectile function were not consistently observed across ARBs, with substantial heterogeneity for erectile function outcomes	Moderate
105	Vlachopoulos et al., 2003	Randomized, placebo controlled, double-blind crossover study	Men with coronary artery disease, including hypertensive patients (n = 24)	Sildenafil 50 mg vs placebo	Arterial stiffness and wave reflection (pulse wave velocity, augmentation index)	Sildenafil significantly reduced aortic stiffness (PWV -0.65 m/s) and wave reflection (augmentation index -4.47%), indicating improved central arterial function beyond its effects on erectile performance	Moderate
106	Santi et al., 2015	Systematic review and meta-analysis of RCTs	Men with type 2 diabetes mellitus (6 RCTs; n = 476)	Chronic phosphodiesterase-5 inhibitor therapy (sildenafil) vs placebo	Endothelial function markers (flow-mediated dilation, inflammatory biomarkers)	Endothelial function markers (flow-mediated dilation, inflammatory biomarkers)	Moderate

107	Sanchez et al., 2017	Narrative review	Men with erectile dysfunction, metabolic syndrome, and cardiovascular risk factors	Clinical evaluation and risk stratification in men with ED	Cardiovascular disease risk; metabolic syndrome	ED shares common risk factors with metabolic syndrome and cardiovascular disease and is considered an independent predictor of future cardiovascular events; clinical encounters for ED represent an opportunity for cardiovascular risk assessment and stratification	Moderate
108	Roy et al., 2003	Narrative review	Patients with erectile dysfunction and cardiovascular disease; experimental models of cardiac disease	Phosphodiesterase 5 inhibitors	Cardiovascular outcomes; cardiac structure and function; safety	Preclinical studies suggest potential cardioprotective effects of PDE5 inhibitors through improved endothelial function and modulation of ischemia–reperfusion injury and cardiac remodeling; however, clinical trials in heart failure and coronary artery disease have shown mixed or neutral efficacy despite a favorable safety profile	Moderate
109	Kloner et al., 2023	Retrospective observational study	Men with erectile dysfunction and cardiovascular risk factors, without prior MACE (n =72,498)	Exposure to phosphodiesterase-5 inhibitors vs non exposure	Major adverse cardiovascular events (MACE) and all cause mortality	PDE-5i exposure was associated with a lower risk of MACE (HR 0.87; 95% CI 0.79 - 0.95) and overall mortality (HR 0.75; 95% CI 0.65 - 0.87); risk reduction showed a dose response relationship and was also observed in men with type 2 diabetes	Moderate
110	Soulaidopoulos et al., 2024	Systematic review and meta-analysis	Men with erectile dysfunction, with or without coronary artery disease (16 cohort studies; n=1,257,759)	Use of phosphodiesterase-5 inhibitors vs placebo, other ED treatments, or no treatment	Major adverse cardiovascular events (MACE) and all cause mortality	PDE5i use was associated with a significant reduction in MACE (RR 0.78; 95% CI 0.69-0.89) and all cause mortality (RR 0.70; 95% CI 0.56-0.87) over long term follow-up	Moderate

111	Koka et al., 2012	Experimental preclinical study	Diabetic db/db mouse model of type 2 diabetes	Chronic treatment with long acting PDE5i (tadalafil)	Cardiac proteomic profile; myocardial structure and redox signaling	Chronic tadalafil treatment reversed diabetes-associated proteomic alterations related to cytoskeletal organization, contractile dysfunction, and oxidative stress, improving myocardial structure and redox balance in diabetic hearts	Moderate
112	Koka et al., 2014	Experimental preclinical study	Diabetic db/db mouse model of type 2 diabetes; wild-type controls	Chronic treatment with long acting PDE-5 inhibitor (tadalafil) for 8 weeks	Mitochondrial function, NO/SIRT1/PGC-1α signaling, oxidative stress, and left ventricular function	Chronic tadalafil treatment activated NO-SIRT1-PGC-1α signaling, improved mitochondrial oxidative phosphorylation, reduced mitochondrial ROS generation, and attenuated left ventricular dysfunction in diabetic hearts	Moderate

Appendix A: Analysis Report: Prevalence, incidence, risk factors and complications related to erectile dysfunction among people with type 2 diabetes mellitus Incidence and Prevalence

TriNetX is the global federated health research network providing access to electronic medical records (diagnoses, procedures, medications, laboratory values, genomic information) across large healthcare organizations (HCOs). This report was run on the set of HCOs grouped into a network called Global Collaborative Network. This network included 165 HCO(s). This report describes an Incidence and Prevalence Analysis, named WITH T2DM, generated by the TriNetX platform on Dec 9, 2025, 12:38:46 UTC. This analysis describes events of interests for the cohort T2DM 09.12 with 4,321,390 patients.

This analysis was run by Bogdan Vlacho (bvlacho@idiapjgol.info) and downloaded by Bogdan Vlacho (bvlacho@idiapjgol.info).

Analysis Inputs for definition of cohort with T2DM

Methods

The analysis process includes two main steps: 1) Defining the cohort through query criteria; 2) Setting up and running the analysis.

Cohorts definition

This section lists all terms used in the definitions of the cohort.

Query Criteria for the Cohort (query name: T2DM 09.12)

This query was run on the network Global Collaborative Network with 165 HCO(s) queried and 165 HCO(s) responded. A total of 161 provider(s) responded with patients. The final cohort included 4,321,390 patients who matched the query criteria listed in the table below.

Ungrouped terms				
must have		demographics	Age	Age (at least 18 years (most recent occurrence))
	and	demographics	UMLS:HL7V3.0:Gender:M	Male
	and	diagnosis	UMLS:ICD10CM:E11	Type 2 diabetes mellitus (between 20 and 80 years old at event)
cannot have		diagnosis	UMLS:ICD10CM:E08	Diabetes mellitus due to underlying condition
	or	diagnosis	UMLS:ICD10CM:E09	Drug or chemical induced diabetes mellitus
	or	diagnosis	UMLS:ICD10CM:E10	Type 1 diabetes mellitus
	or	diagnosis	UMLS:ICD10CM:E13	Other specified diabetes mellitus

Lookback Period

A lookback period determines how far back in the database the analysis will look when determining whether cases are incident, prevalent, or neither. Patients who had an instance of the event of interest during the lookback period will be considered a prevalent case if they have an instance of the event of interest in the time window. The following is the setting used for this analysis' lookback period:

Look back from any time to one day before the start of each time window.

Strata Inputs

Below are the selected strata inputs used to stratify the results of the analysis:

Age: Selected

Sex: Not Selected

Race: Selected

Ethnicity: Selected

1 Analysis Inputs for cohort without diabetes

Methods

The analysis process includes two main steps: 1) Defining the cohort through query criteria; 2) Setting up and running the analysis.

Cohorts definition

This section lists all terms used in the definitions of the cohort.

1.1.1 Query Criteria for the Cohort (query name: without diabetes)

This query was run on the network Global Collaborative Network with 168 HCO(s) queried and 168 HCO(s) responded. A total of 168 provider(s) responded with patients. The final cohort included 67,108,795 patients who matched the query criteria listed in the table below. For the text representation of the query criteria please see Appendix A.

Ungrouped terms				
must have		demographics	Age	Age (at least 20 years (most recent occurrence))
	and	demographics	UMLS:HL7V3.0:Gender:M	Male
cannot have		diagnosis	UMLS:ICD10CM:E08	Diabetes mellitus due to underlying condition
	or	diagnosis	UMLS:ICD10CM:E09	Drug or chemical induced diabetes mellitus
	or	diagnosis	UMLS:ICD10CM:E10	Type 1 diabetes mellitus
	or	diagnosis	UMLS:ICD10CM:E13	Other specified diabetes mellitus

or	diagnosis	UMLS:ICD10CM:E11	Type 2 diabetes mellitus (between 20 and 80 years old at event)

1.2 Time Window Inputs

Time Window Start	Time Window End	Label
2024-12-09	2025-12-09	

1.3 Lookback Period

A lookback period determines how far back in the database the analysis will look when determining whether cases are incident, prevalent, or neither. Patients who had an instance of the event of interest during the lookback period will be considered a prevalent case if they have an instance of the event of interest in the time window. The following is the setting used for this analysis' lookback period:

Look back from 360 days to one day before the start of each time window.

1.4 Strata Inputs

Below are the selected strata inputs used to stratify the results of the analysis:

Age: Selected

Sex: Not Selected

Race: Selected

Ethnicity: Selected

Event of Interest Definitions

Table below outlines definitions for each event of interest. For the definitions of the events of interest consisting of more than one term, at least one term must match. Please see Appendix B for the text representation of these definitions.

Erectile disfunction		
**Event of interest definition**		
Diagnosis	UMLS:ICD10CM:N52	Male erectile dysfunction
Diagnosis	UMLS:ICD10CM:N52.0	Vasculogenic erectile dysfunction
Diagnosis	UMLS:ICD10CM:N52.9	Male erectile dysfunction, unspecified
Diagnosis	UMLS:ICD10CM:N52.8	Other male erectile dysfunction
Diagnosis	UMLS:ICD10CM:N52.02	Corporo-venous occlusive erectile dysfunction
Diagnosis	UMLS:ICD10CM:N52.1	Erectile dysfunction due to diseases classified elsewhere
Diagnosis	UMLS:ICD10CM:N52.01	Erectile dysfunction due to arterial insufficiency
Diagnosis	UMLS:ICD10CM:I77.1	Stricture of artery
Diagnosis	UMLS:ICD10CM:E11.618	Type 2 diabetes mellitus with other diabetic arthropathy
Diagnosis	UMLS:ICD10CM:E11.69	Type 2 diabetes mellitus with other specified complication
Diagnosis	UMLS:ICD10CM:E11.40	Type 2 diabetes mellitus with diabetic neuropathy, unspecified
Diagnosis	UMLS:ICD10CM:N52.03	Combined arterial insufficiency and corporo-venous occlusive erectile dysfunction
Medication	NLM:ATC:G04BE	Drugs used in erectile dysfunction

Appendix B: Propensity-score–matched comparative analysis for estimating the risk for different cardiovascular events among people with erectile dysfunction in the presence of type 2 diabetes and the absence of diabetes

2 Methods

The analysis process includes two main steps: 1) Defining the cohorts through query criteria; 2) Setting up and running the analysis. Setting up the analysis requires definitions for the index event, outcomes criteria, and the time frame. Compare outcomes supports four analyses: Measures of Association, Survival, Number of Instances and Lab result distribution. These analyses have additional options that are listed in the Outcomes Definitions and Analyses Specifications section below. Furthermore, characteristics of the cohorts that are balanced using propensity score matching are also included in the Propensity Score Matching section.

2.1 Cohorts definition

This section lists all terms used in the definitions of the two cohorts.

2.1.1

2.1.2 Query Criteria for Cohort 1 (query name: ED with T2DM)

This query was run on the network Global Collaborative Network with 161 HCO(s) queried and 161 HCO(s) responded. A total of 83 provider(s) responded with patients. The final cohort included 312,644 patients who matched the query criteria listed in the table below. For the text representation of the query criteria please see Appendix A.

Ungrouped terms				
must have		demographics	Age	Age (between 20 and 80 years (most recent occurrence))
	and	demographics	UMLS:HL7V3.0:Gender: M	Male
	and any of	diagnosis	UMLS:ICD10CM:N52	Male erectile dysfunction
		diagnosis	UMLS:ICD10CM:N52.0	Vasculogenic erectile dysfunction
	and any of	diagnosis	UMLS:ICD10CM:N52.9	Male erectile dysfunction, unspecified
		diagnosis	UMLS:ICD10CM:N52.8	Other male erectile dysfunction
		diagnosis	UMLS:ICD10CM:N52.02	Corporo-venous occlusive erectile dysfunction

		medication	NLM:ATC:G04BE	Drugs used in erectile dysfunction
		diagnosis	UMLS:ICD10CM:N52.1	Erectile dysfunction due to diseases classified elsewhere
		diagnosis	UMLS:ICD10CM:N52.01	Erectile dysfunction due to arterial insufficiency
		diagnosis	UMLS:ICD10CM:N52.03	Combined arterial insufficiency and corporo-venous occlusive erectile dysfunction
	and	diagnosis	UMLS:ICD10CM:E11	Type 2 diabetes mellitus
cannot have		diagnosis	UMLS:ICD10CM:E08	Diabetes mellitus due to underlying condition
	or	diagnosis	UMLS:ICD10CM:E09	Drug or chemical induced diabetes mellitus
	or	diagnosis	UMLS:ICD10CM:E10	Type 1 diabetes mellitus
	or	diagnosis	UMLS:ICD10CM:E13	Other specified diabetes mellitus

2.1.3 Query Criteria for Cohort 2 (query name: ED without T2DM)

This query was run on the network Global Collaborative Network with 161 HCO(s) queried and 161 HCO(s) responded. A total of 150 provider(s) responded with patients. The final cohort included 1,459,702 patients who matched the query criteria listed in the table below.

Ungrouped terms				
must have		demographics	Age	Age (between 20 and 80 years (most recent occurrence))
	and	demographics	UMLS:HL7V3.0:Gender:M	Male
	and any of	diagnosis	UMLS:ICD10CM:N52	Male erectile dysfunction
		diagnosis	UMLS:ICD10CM:N52.0	Vasculogenic erectile dysfunction
		diagnosis	UMLS:ICD10CM:N52.9	Male erectile dysfunction, unspecified
		diagnosis	UMLS:ICD10CM:N52.8	Other male erectile dysfunction

		diagnosis	UMLS:ICD10CM:N52.02	Corporo-venous occlusive erectile dysfunction
		medication	NLM:ATC:G04BE	Drugs used in erectile dysfunction
		diagnosis	UMLS:ICD10CM:N52.1	Erectile dysfunction due to diseases classified elsewhere
		diagnosis	UMLS:ICD10CM:N52.01	Erectile dysfunction due to arterial insufficiency
		diagnosis	UMLS:ICD10CM:N52.03	Combined arterial insufficiency and corporo-venous occlusive erectile dysfunction
cannot have		diagnosis	UMLS:ICD10CM:E08-E13	Diabetes mellitus

2.2 Analysis Setup

This section contains the Index Event and Time Window definitions and a list of selected outcomes and the analyses.

2.2.1 Index Event & Time Window Definitions

The index event defines the point in time when each patient in the cohort enters the analysis. To define an index event for the cohort, one or more criteria for the cohort must be selected. The index date for each patient within a cohort is the day on which the patient first met the selected criteria for the cohort (listed in the table below).

As the index event defines the earliest time point after which outcomes are analyzed, the time window defines the duration during which outcomes are analyzed. The time window can start on the same day as the index event or at any specified time interval after the index event. The time window can end any time after the start date. Outcomes are defined as diagnoses, medications, procedures, or laboratory values that happened in the time window starting after the first occurrence of the index event.

2.2.2 Time Window Used in this Analysis

This analysis included outcomes that occurred in the time window that started 1 day after the first occurrence of the index event. Since no end date was specified all outcomes after the first occurrence of the index event were included.

The index event only includes events that occurred up to 20 years ago. Patients whose index event occurred 20 years or more ago are excluded. In this analysis, 0 patients in Cohort 1 and 0 patients in Cohort 2 were excluded because they met the index event more than 20 years ago.

2.2.3 Index Events Used in this Analysis

Index events for the Compare Outcomes analysis were derived from the cohort definitions. Index events were defined separately for each cohort and were based on the criteria used in the original cohort definition. Please see Appendix B for the text representation of the index event definition.

The index event for Cohort 1 (query name: ED with T2DM) was defined as the following:

Ungrouped terms				
must have	any of	diagnosis	UMLS:ICD10CM:N52	Male erectile dysfunction
		diagnosis	UMLS:ICD10CM:N52.0	Vasculogenic erectile dysfunction
	and any of	diagnosis	UMLS:ICD10CM:N52.9	Male erectile dysfunction, unspecified
		diagnosis	UMLS:ICD10CM:N52.8	Other male erectile dysfunction
		diagnosis	UMLS:ICD10CM:N52.02	Corporo-venous occlusive erectile dysfunction
		medication	NLM:ATC:G04BE	Drugs used in erectile dysfunction
		diagnosis	UMLS:ICD10CM:N52.1	Erectile dysfunction due to diseases classified elsewhere
		diagnosis	UMLS:ICD10CM:N52.01	Erectile dysfunction due to arterial insufficiency
		diagnosis	UMLS:ICD10CM:N52.03	Combined arterial insufficiency and corporo-venous occlusive erectile dysfunction
	and	diagnosis	UMLS:ICD10CM:E11	Type 2 diabetes mellitus

The index event for Cohort 2 (query name: ED without T2DM) was defined as the following:

Ungrouped terms				
must have	any of	diagnosis	UMLS:ICD10CM:N52	Male erectile dysfunction
		diagnosis	UMLS:ICD10CM:N52.0	Vasculogenic erectile dysfunction

diagnosis	UMLS:ICD10CM:N52.9	Male erectile dysfunction, unspecified
diagnosis	UMLS:ICD10CM:N52.8	Other male erectile dysfunction
diagnosis	UMLS:ICD10CM:N52.02	Corporo-venous occlusive erectile dysfunction
medication	NLM:ATC:G04BE	Drugs used in erectile dysfunction
diagnosis	UMLS:ICD10CM:N52.1	Erectile dysfunction due to diseases classified elsewhere
diagnosis	UMLS:ICD10CM:N52.01	Erectile dysfunction due to arterial insufficiency
diagnosis	UMLS:ICD10CM:N52.03	Combined arterial insufficiency and corporo-venous occlusive erectile dysfunction

2.2.4 Analyses Specifications

The Compare Outcomes Analytic supports four types of analyses: Measure of Association, Survival, Number of Instances, and Lab result distribution. The first three analyses support the "exclude patients with outcomes prior to the window" setting. This option can exclude patients from the analysis if they are not at risk for an outcome (e.g., if the outcome is a chronic disease). When "exclude patients with the outcome prior to the time window" is not checked, all patients in the cohort are included in the analysis, regardless of whether they had the outcome prior to the time window. When "exclude patients with the outcome prior to the time window" is checked, patients are excluded from the analysis if their record includes the outcome prior to the beginning of the time window. This selection will exclude all patients with the outcome prior to the index event. If the start of the time window for the analysis falls some days after the index event, patients will also be excluded if they have the outcome between the index event and the start of the time window.

2.2.5 Measure of Association Analysis

The Measure of Association Analysis calculates and compares the fraction of patients with the selected outcome. The output summary includes: Patients in each Cohort (count of patients meeting query criteria); Patients with Outcome in each Cohort (of the patients in the cohort, count of patients that had the outcome in the time window); and Risk (the fraction of patients in the cohort that have the outcome in the time window, i.e. Patients with Outcome / Patients in Cohort). In addition, Risk Difference (the difference in the risks in Cohort 1 and Cohort 2), Risk Ratio (the ratio of the risks in Cohort 1 and Cohort 2), and Odds Ratio (the ratio of the odds in Cohort 1 and Cohort 2). The bar chart shows the risk of the outcome for the both cohorts.

2.2.6 Survival Analysis

The Kaplan-Meier Analysis estimates probability of the outcome at a respective time interval (daily time interval is used in this analysis). In order to account for the patients who exited the cohort during the analysis period, and therefore should not be included in the analysis, censoring is applied. In this analysis, patients are removed from the analysis (censored) after the last fact in their record.

The output summary includes: Patients in each Cohort (count of patients meeting query criteria); Patients with Outcome (of the patients in the cohort, count of patients that had the outcome in the time window); Median Survival (the number of days when the survival drops below 50%; the "-" indicates that survival does not drop below 50% during the time window); and Survival Probability at End of Time Window (the % survival at the end of the time window). In addition, Log-Rank test, Hazard Ratio and test for Proportionality.

2.2.7 Number of Instances Analysis

The Number of Instances Analysis calculates how many times the outcome occurred in the time window. This analysis includes two additional settings: include patients with zero instances; the definition of an instance.

Selecting to exclude patients with zero instances will remove these patients from the calculations for mean number of instances, standard deviation, or median. The histogram showing the distribution of patients by number of instances will not contain a bar for zero. Alternatively, by selecting to include patients with zero instances, the mean, standard deviation, and median for number of instances will reflect these patients. The histogram will contain a bar for zero patients.

The definition of an instance affects how counts are analyzed. By selecting Date, each calendar date on which any of the terms selected in the outcome are recorded will represent one instance. For example, if the outcome is "Med A or Med B," and a patient has "Med A" on January 3, then both medications on January 4, then "Med B" on January 6, then that patient is considered to have three instances– January 3, January 4, and January 6. Note that if an outcome occurs across several dates (e.g. Visit: inpatient encounter), then only the start date is tracked for the purpose of counting instances. A patient who begins at stay on January 1, ends that stay on January 3, begins another stay on January 10, and ends that stay on January 15, is considered to have two instances of the outcome.

Selecting Visit as an Instance will count any visit that includes the outcome as one instance, regardless of how many times it occurred. For instance, consider a patient administered an analgesic on each of the three days that make up an inpatient stay following some index event. If analgesic is an outcome, these three administrations will represent only one instance, because all three are associated with the same visit.

The output summary includes: Patients in Cohort (count of patients meeting query criteria); Patients with Outcome (of the patients in the cohort, count of patients that had the outcome in the time window); Mean (mean of the counts); Standard Deviation (standard deviation of the counts); Median (median of the counts); and Median (1+

instances) when patients with zero instances included in the analysis. In addition, T-Test statistics testing for the difference between the cohorts is included.

2.2.8 Laboratory Results Analysis

Lab Results can be included in the analysis only for the outcomes that are labs. Only the most recent lab values in the time window are included. For the lab results that are numeric, the outcome summary includes: Patients in Cohort (count of patients meeting query criteria); Patients with Outcome (of the patients in the cohort, count of patients that had the outcome in the time window); Mean (mean of the counts); and Standard Deviation (the standard deviation for lab values across patients in the cohort). In addition, T-Test statistics testing for the difference between the cohorts is included.

For the non-numeric lab results, three values are reported: counts of Negative; Positives; and Unknowns.

The counts are represented in the bar chart as percentages of the total counts.

2.2.9 Outcome Definitions

Table below outlines the definitions for each outcome and the analysis specifications. For outcome definitions consisting of more than one term, at least one term must match. Please see Appendix C for the text representation of the outcome definitions.

**Ischemic hearth disease**		
Outcome definition		
Diagnosis	UMLS:ICD10CM:I20-I25	Ischemic heart diseases
Settings for the performed analyses		
Kaplan - Meier survival analysis		excluding patients with outcome prior to the time window
Risk analysis		excluding patients with outcome prior to the time window
Number of instances analysis		excluding patients with outcome prior to the time window<br>excluding patients with zero outcomes<br>counts are grouped by date
**Stroke**		
Outcome definition		
Diagnosis	UMLS:ICD10CM:I60-I69	Cerebrovascular diseases
Settings for the performed analyses		

Risk analysis		excluding patients with outcome prior to the time window
Kaplan - Meier survival analysis		excluding patients with outcome prior to the time window
Number of instances analysis		excluding patients with outcome prior to the time window<br>excluding patients with zero outcomes<br>counts are grouped by date
**Heart failure**		
Outcome definition		
Diagnosis	UMLS:ICD10CM:I50	Heart failure
Settings for the performed analyses		
Risk analysis		excluding patients with outcome prior to the time window
Kaplan - Meier survival analysis		excluding patients with outcome prior to the time window
Number of instances analysis		excluding patients with outcome prior to the time window<br>excluding patients with zero outcomes<br>counts are grouped by date
**Peripheral artery disease**		
Outcome definition		
Diagnosis	UMLS:ICD10CM:I80-I89	Diseases of veins, lymphatic vessels and lymph nodes, not elsewhere classified
Settings for the performed analyses		
Risk analysis		excluding patients with outcome prior to the time window
Kaplan - Meier survival analysis		excluding patients with outcome prior to the time window
Number of instances analysis		excluding patients with outcome prior to the time window<br>excluding patients with zero outcomes<br>counts are grouped by date
**CVD**		
Outcome definition		
Diagnosis	UMLS:ICD10CM:I20-I25	Ischemic heart diseases
Diagnosis	UMLS:ICD10CM:I60-I69	Cerebrovascular diseases

Diagnosis	UMLS:ICD10CM:I80-I89	Diseases of veins, lymphatic vessels and lymph nodes, not elsewhere classified
Diagnosis	UMLS:ICD10CM:I50	Heart failure
Settings for the performed analyses		
Risk analysis		excluding patients with outcome prior to the time window
Kaplan - Meier survival analysis		excluding patients with outcome prior to the time window
Number of instances analysis		excluding patients with outcome prior to the time window<br>excluding patients with zero outcomes<br>counts are grouped by date

2.3 Propensity Score Matching

Propensity score matching was performed on 18 characteristic(s). In the Demographics category patients were matched on Age at Index characteristic(s). In the Diagnosis category patients were matched on Hypertensive diseases, Heart failure, Ischemic heart diseases, Cerebrovascular diseases, Diseases of arteries, arterioles and capillaries, Disorders of lipoprotein metabolism and other lipidemias, Chronic kidney disease (CKD), Nicotine dependence, unspecified, uncomplicated characteristic(s). In the Laboratory category patients were matched on Creatinine [Mass/volume] in Serum, Plasma or Blood, Cholesterol [Mass/volume] in Serum or Plasma, Cholesterol in LDL [Mass/volume] in Serum or Plasma, Cholesterol in HDL [Mass/volume] in Serum or Plasma, Triglyceride [Mass/volume] in Serum, Plasma or Blood, Hemoglobin A1c/Hemoglobin.total in Blood, BMI, Blood Pressure, Systolic, Blood Pressure, Diastolic characteristic(s). Characteristics of the cohorts before and after matching are summarized in the table below.

Cohort 1 and cohort 2 patient count before and after propensity score matching		
Cohort	Patient count before matching	Patient count after matching
1 - ED with T2DM	331,649	312,545
2 - ED without T2DM	1,579,633	312,545

Propensity score density function - Before and after matching (cohort 1 - purple, cohort 2 - green)

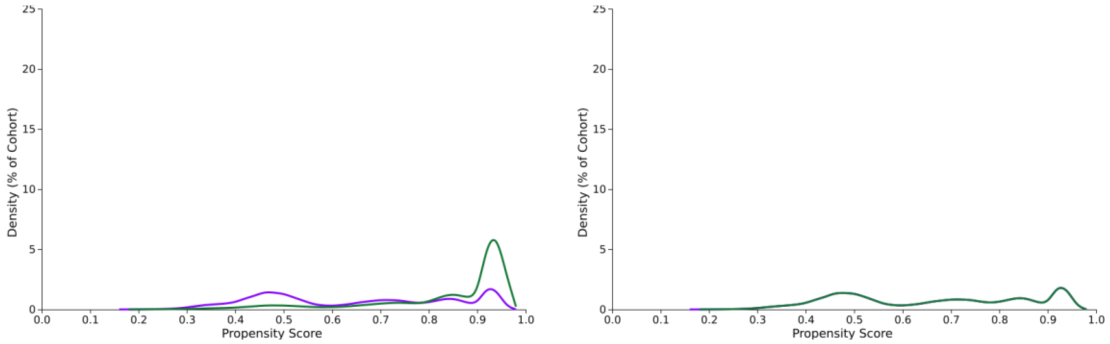

Cohort 1 (N = 331,649) and cohort 2 (N = 1,579,633) characteristics before propensity score matching							
**Demographics**							
Cohort			Mean ± SD	Patients	% of Cohort	P-Value	Std diff.
1<br>2	AI	Age at Index	59.2 +/- 9.7<br>54.8 +/- 12.6	330,120<br>1,561,521	100%<br>100%	<0.001	0.395
**Diagnosis**							
Cohort			Mean ± SD	Patients	% of Cohort	P-Value	Std diff.
1<br>2	I10-I1A	Hypertensive diseases		224,758<br>447,998	68.1%<br>28.7%	<0.001	0.858
1<br>2	I50	Heart failure		27,479<br>41,632	8.3%<br>2.7%	<0.001	0.250
1<br>2	I20-I25	Ischemic heart diseases		71,403<br>149,539	21.6%<br>9.6%	<0.001	0.337
1<br>2	I60-I69	Cerebrovascular diseases		26,554<br>53,714	8.0%<br>3.4%	<0.001	0.199
1<br>2	I70-I79	Diseases of arteries, arterioles and capillaries		41,037<br>86,108	12.4%<br>5.5%	<0.001	0.244
1<br>2	E78	Disorders of lipoprotein metabolism and other lipidemias		211,948<br>429,807	64.2%<br>27.5%	<0.001	0.792
1<br>2	N18	Chronic kidney disease (CKD)		36,688<br>48,816	11.1%<br>3.1%	<0.001	0.314
1<br>2	F17.200	Nicotine dependence, unspecified, uncomplicated		41,768<br>103,886	12.7%<br>6.7%	<0.001	0.204

Procedure							
Cohort			Mean ± SD	Patients	% of Cohort	P-Value	Std diff.
1<br>2	1018513	Smoking and tobacco use cessation counseling visit		5,069<br>9,755	1.5%<br>0.6%	<0.001	0.088

Laboratory							
Cohort			Mean ± SD	Patients	% of Cohort	P-Value	Std diff.
1<br>2	9024	Creatinine [Mass/volume] in Serum, Plasma or Blood	1.3 +/- 4.7<br>1.2 +/- 4.3	256,699<br>822,643	77.8%<br>52.7%	<0.001	0.011
1<br>2		0 - 0 mg/dL		256,712<br>822,650	77.8%<br>52.7%	<0.001	0.546
1<br>2	9000	Cholesterol [Mass/volume] in Serum or Plasma	167.1 +/- 46.4<br>180.8 +/- 42.7	213,096<br>577,231	64.6%<br>37.0%	<0.001	0.307
1<br>2		0 - 0 mg/dL		213,101<br>577,234	64.6%<br>37.0%	<0.001	0.574
1<br>2	9002	Cholesterol in LDL [Mass/volume] in Serum or Plasma	93.0 +/- 37.1<br>107.5 +/- 35.9	211,929<br>575,517	64.2%<br>36.9%	<0.001	0.396
1<br>2		0 - 0 mg/dL		211,944<br>575,529	64.2%<br>36.9%	<0.001	0.569
1<br>2	9001	Cholesterol in HDL [Mass/volume] in Serum or Plasma	40.2 +/- 14.8<br>46.6 +/- 16.5	214,268<br>579,912	64.9%<br>37.1%	<0.001	0.411
1<br>2		0 - 0 mg/dL		214,270<br>579,920	64.9%<br>37.1%	<0.001	0.578
1<br>2	9004	Triglyceride [Mass/volume] in Serum, Plasma or Blood	173.6 +/- 165.6<br>133.5 +/- 97.2	212,708<br>576,165	64.4%<br>36.9%	<0.001	0.296
1<br>2		0 - 0 mg/dL		212,708<br>576,166	64.4%<br>36.9%	<0.001	0.573
1<br>2	9037	Hemoglobin A1c/Hemoglobin.total in Blood	7.1 +/- 1.8<br>5.6 +/- 0.8	216,100<br>335,298	65.5%<br>21.5%	<0.001	1.128

Cohort			Mean ± SD	Patients	% of Cohort	P-Value	Std diff.
1<br>2		0 - 0 %		216,104<br>335,323	65.5%<br>21.5%	<0.001	0.990
1<br>2	9083	BMI	32.8 +/- 6.6<br>29.3 +/- 5.6	216,226<br>737,920	65.5%<br>47.3%	<0.001	0.575
1<br>2		0 - 0 kg/m2		216,260<br>738,063	65.5%<br>47.3%	<0.001	0.374
1<br>2	9085	Blood Pressure, Systolic	132.4 +/- 18.1<br>129.2 +/- 17.2	243,416<br>855,820	73.7%<br>54.8%	<0.001	0.184
1<br>2		0 - 0 mm[Hg]		243,418<br>855,834	73.7%<br>54.8%	<0.001	0.403
1<br>2	9086	Blood Pressure, Diastolic	78.6 +/- 11.3<br>78.4 +/- 11.2	243,525<br>856,159	73.8%<br>54.8%	<0.001	0.021
1<br>2		0 - 0 mm[Hg]		243,528<br>856,187	73.8%<br>54.8%	<0.001	0.403
1<br>2	88031-0	Smokeless tobacco status		0<br>0	0%<br>0%	--	--
1<br>2		0 - 0 units		0<br>0	0%<br>0%	--	--

Cohort 1 (N = 312,545) and cohort 2 (N = 312,545) characteristics after propensity score matching

Demographics

Cohort			Mean ± SD	Patients	% of Cohort	P-Value	Std diff.
1<br>2	AI	Age at Index	58.9 +/- 9.7<br>59.2 +/- 9.9	312,545<br>312,545	100%<br>100%	<0.001	0.027

Diagnosis

Cohort			Mean ± SD	Patients	% of Cohort	P-Value	Std diff.
1<br>2	I10-I1A	Hypertensive diseases		207,192<br>206,609	66.3%<br>66.1%	0.119	0.004
1<br>2	I50	Heart failure		23,721<br>23,052	7.6%<br>7.4%	0.001	0.008

Cohort	Code	Description	Mean ± SD	Patients	% of Cohort	P-Value	Std diff.
1<br>2	I20-I25	Ischemic heart diseases		66,114<br>69,036	21.2%<br>22.1%	<0.001	0.023
1<br>2	I60-I69	Cerebrovascular diseases		24,629<br>25,656	7.9%<br>8.2%	<0.001	0.012
1<br>2	I70-I79	Diseases of arteries, arterioles and capillaries		37,444<br>37,429	12.0%<br>12.0%	0.953	<0.001
1<br>2	E78	Disorders of lipoprotein metabolism and other lipidemias		194,620<br>191,900	62.3%<br>61.4%	<0.001	0.018
1<br>2	N18	Chronic kidney disease (CKD)		30,036<br>27,526	9.6%<br>8.8%	<0.001	0.028
1<br>2	F17.200	Nicotine dependence, unspecified, uncomplicated		37,949<br>36,684	12.1%<br>11.7%	<0.001	0.012

Procedure

Cohort			Mean ± SD	Patients	% of Cohort	P-Value	Std diff.
1<br>2	1018513	Smoking and tobacco use cessation counseling visit		4,574<br>4,409	1.5%<br>1.4%	0.080	0.004

Laboratory

Cohort			Mean ± SD	Patients	% of Cohort	P-Value	Std diff.
1<br>2	9024	Creatinine [Mass/volume] in Serum, Plasma or Blood	1.3 +/- 4.8<br>1.3 +/- 4.6	240,001<br>240,227	76.8%<br>76.9%	0.072	0.005
1<br>2		0 - 0 mg/dL		240,014<br>240,228	76.8%<br>76.9%	0.521	0.002
1<br>2	9000	Cholesterol [Mass/volume] in Serum or Plasma	167.6 +/- 46.5<br>176.7 +/- 43.3	198,490<br>193,984	63.5%<br>62.1%	<0.001	0.203
1<br>2		0 - 0 mg/dL		198,495<br>193,984	63.5%<br>62.1%	<0.001	0.030
1<br>2	9002	Cholesterol in LDL [Mass/volume] in Serum or Plasma	93.5 +/- 37.2<br>103.1 +/- 36.8	197,592<br>193,371	63.2%<br>61.9%	<0.001	0.260
1<br>2		0 - 0 mg/dL		197,607<br>193,374	63.2%<br>61.9%	<0.001	0.028

1<br>2	9001	Cholesterol in HDL [Mass/volume] in Serum or Plasma	40.2 +/- 14.7<br>45.5 +/- 16.7	199,619<br>195,335	63.9%<br>62.5%	<0.001	0.338
1<br>2		0 - 0 mg/dL		199,621<br>195,337	63.9%<br>62.5%	<0.001	0.028
1<br>2	9004	Triglyceride [Mass/volume] in Serum, Plasma or Blood	174.0 +/- 166.0<br>138.6 +/- 101.5	198,156<br>193,882	63.4%<br>62.0%	<0.001	0.257
1<br>2		0 - 0 mg/dL		198,156<br>193,882	63.4%<br>62.0%	<0.001	0.028
1<br>2	9037	Hemoglobin A1c/Hemoglobin.total in Blood	7.2 +/- 1.8<br>5.6 +/- 0.6	198,526<br>199,242	63.5%<br>63.7%	<0.001	1.187
1<br>2		0 - 0 %		198,529<br>199,251	63.5%<br>63.8%	0.058	0.005
1<br>2	9083	BMI	32.8 +/- 6.6<br>30.0 +/- 5.8	201,990<br>202,197	64.6%<br>64.7%	<0.001	0.458
1<br>2		0 - 0 kg/m2		202,023<br>202,218	64.6%<br>64.7%	0.606	0.001
1<br>2	9085	Blood Pressure, Systolic	132.5 +/- 18.0<br>130.7 +/- 17.8	227,817<br>227,535	72.9%<br>72.8%	<0.001	0.100
1<br>2		0 - 0 mm[Hg]		227,819<br>227,537	72.9%<br>72.8%	0.423	0.002
1<br>2	9086	Blood Pressure, Diastolic	78.7 +/- 11.2<br>78.6 +/- 11.4	227,926<br>227,588	72.9%<br>72.8%	<0.001	0.013
1<br>2		0 - 0 mm[Hg]		227,929<br>227,594	72.9%<br>72.8%	0.341	0.002
1<br>2	88031-0	Smokeless tobacco status		0<br>0	0%<br>0%	--	--
1<br>2		0 - 0 units		0<br>0	0%<br>0%	--	--

3 Results

Results are summarized in the tables below. Outcomes analysis was performed on the cohorts after propensity score matching.

Follow-up Time (Before Matching)

Cohort	Mean Follow-up (Days)	Standard Deviation	Median Follow-up (Days)	Interquartile Range
ED with T2DM	1527.913	1318.283	1177	1788
ED without T2DM	1586.411	1463.342	1180	2052

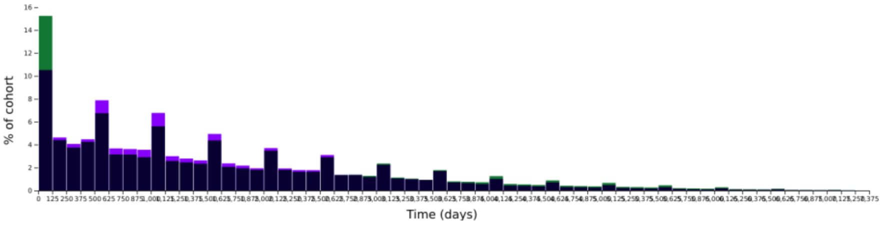

1,529 patients in Cohort 1 and 18,112 patients in Cohort 2 were excluded because they met the index event more than 20 years ago.

Follow-up Time (After Matching)

Cohort	Mean Follow-up (Days)	Standard Deviation	Median Follow-up (Days)	Interquartile Range
ED with T2DM	1541.981	1327.086	1190	1808
ED without T2DM	1415.098	1287.523	1058	1715

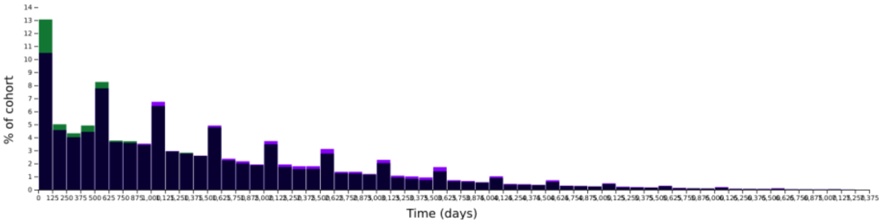

1 Ischemic hearth disease

Risk analysis excluding patients with outcome prior to the time window

Cohort		Patients in cohort	Patients with outcome	Risk
1	ED with T2DM	238,185	37,506	0.157
2	ED without T2DM	241,115	27,784	0.115

		95% CI	z	p
Risk Difference	0.042	(0.040, 0.044)	42.620	0
Risk Ratio	1.367	(1.347, 1.386)	N/A	N/A
Odds Ratio	1.435	(1.411, 1.459)	N/A	N/A

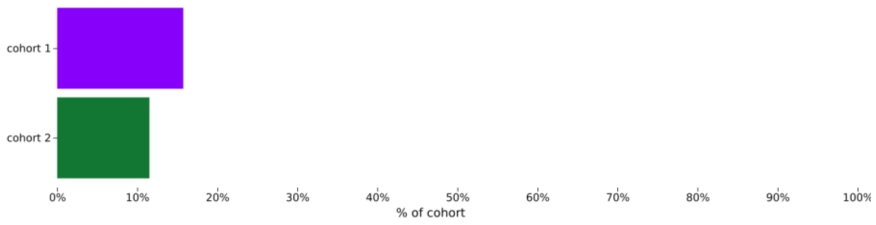

74,360 patients in Cohort 1 and 71,430 patients in Cohort 2 were excluded from results because they had the outcome prior to the time window.

Kaplan - Meier survival analysis excluding patients with outcome prior to the time window

Cohort		Patients in cohort	Patients with outcome	Median survival (days)	Survival probability at end of time window
1	ED with T2DM	238,185	37,506	5911	40.38%
2	ED without T2DM	241,115	27,784	6842	47.74%

	χ^2	df	p
Log-Rank Test	1057.189	1	0.000

	Hazard Ratio	95% CI	χ^2	df	p
Hazard Ratio and Proportionality	1.293	(1.273, 1.313)	0.753	1	0.385

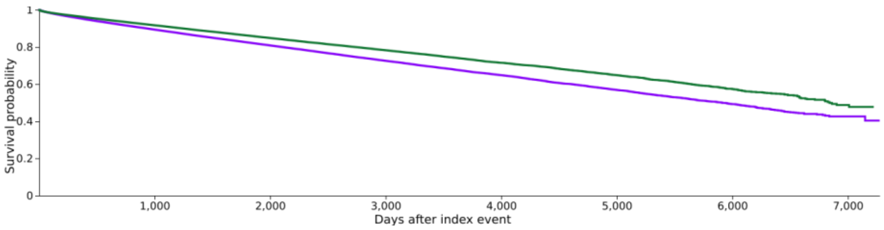

74,360 patients in Cohort 1 and 71,430 patients in Cohort 2 were excluded from results because they had the outcome prior to the time window.

Number of instances excluding patients with outcome prior to the time window

Cohort		Patients in cohort	Patients with outcome	Mean	Standard Deviation	Median
1	ED with T2DM	238,185	37,506	7.896	14.527	3
2	ED without T2DM	241,115	27,784	6.128	11.018	2

	t	df	p
Test Statistics	16.984	65288	0.000

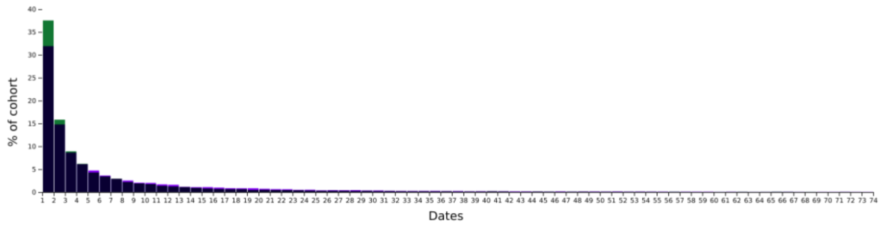

239 data points for Cohort 1 and 103 data points for Cohort 2 were omitted for display purposes.
74,360 patients in Cohort 1 and 71,430 patients in Cohort 2 were excluded from results because they had the outcome prior to the time window.

2 Stroke

Risk analysis excluding patients with outcome prior to the time window

Cohort		Patients in cohort	Patients with outcome	Risk
1	ED with T2DM	285,311	22,450	0.079
2	ED without T2DM	285,837	15,895	0.056

		95% CI	z	p
Risk Difference	0.023	(0.022, 0.024)	34.845	0.000
Risk Ratio	1.415	(1.387, 1.443)	N/A	N/A
Odds Ratio	1.450	(1.420, 1.481)	N/A	N/A

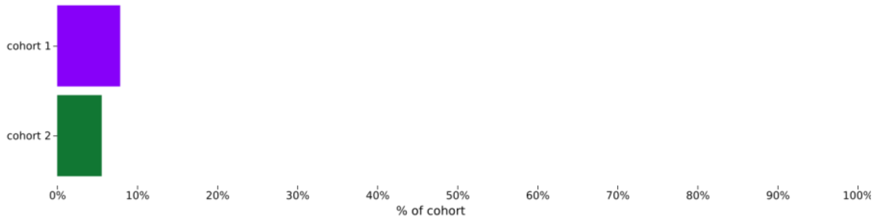

27,234 patients in Cohort 1 and 26,708 patients in Cohort 2 were excluded from results because they had the outcome prior to the time window.

Kaplan - Meier survival analysis excluding patients with outcome prior to the time window

Cohort		Patients in cohort	Patients with outcome	Median survival (days)	Survival probability at end of time window
1	ED with T2DM	285,311	22,450	--	60.82%
2	ED without T2DM	285,837	15,895	--	66.70%

	χ^2	df	p
Log-Rank Test	699.087	1	0.000

	Hazard Ratio	95% CI	χ^2	df	p
Hazard Ratio and Proportionality	1.314	(1.288, 1.341)	10.877	1	0.001

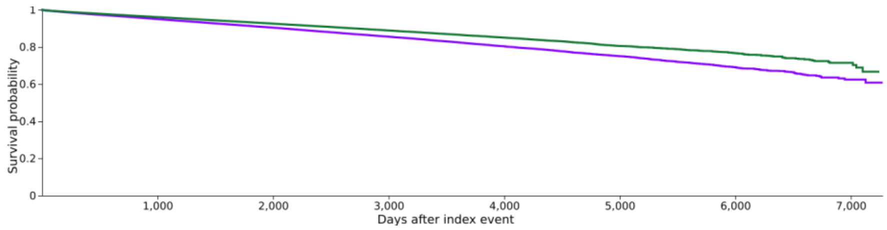

27,234 patients in Cohort 1 and 26,708 patients in Cohort 2 were excluded from results because they had the outcome prior to the time window.

Number of instances excluding patients with outcome prior to the time window

Cohort		Patients in cohort	Patients with outcome	Mean	Standard Deviation	Median
1	ED with T2DM	285,311	22,450	5.667	11.526	2
2	ED without T2DM	285,837	15,895	4.878	10.219	2

	t	df	p
Test Statistics	6.921	38343	0.000

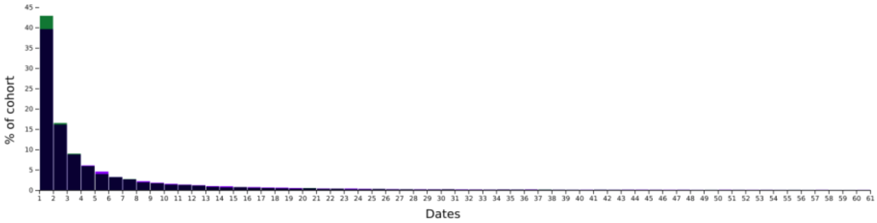

154 data points for Cohort 1 and 64 data points for Cohort 2 were omitted for display purposes.
27,234 patients in Cohort 1 and 26,708 patients in Cohort 2 were excluded from results because they had the outcome prior to the time window.

3 Heart failure

Risk analysis excluding patients with outcome prior to the time window

Cohort		Patients in cohort	Patients with outcome	Risk
1	ED with T2DM	285,408	23,842	0.084
2	ED without T2DM	287,971	14,069	0.049

		95% CI	z	p
Risk Difference	0.035	(0.033, 0.036)	52.841	0
Risk Ratio	1.710	(1.676, 1.745)	N/A	N/A
Odds Ratio	1.775	(1.737, 1.813)	N/A	N/A

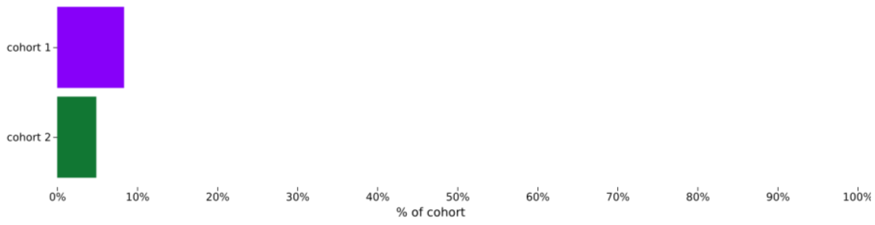

27,137 patients in Cohort 1 and 24,574 patients in Cohort 2 were excluded from results because they had the outcome prior to the time window.

Kaplan - Meier survival analysis excluding patients with outcome prior to the time window

Cohort	Patients in cohort	Patients with outcome	Median survival (days)	Survival probability at end of time window
1 ED with T2DM	285,408	23,842	--	58.30%
2 ED without T2DM	287,971	14,069	--	70.75%

	χ^2	df	p
Log-Rank Test	1985.220	1	0

	Hazard Ratio	95% CI	χ^2	df	p
Hazard Ratio and Proportionality	1.599	(1.566, 1.633)	42.686	1	0.000

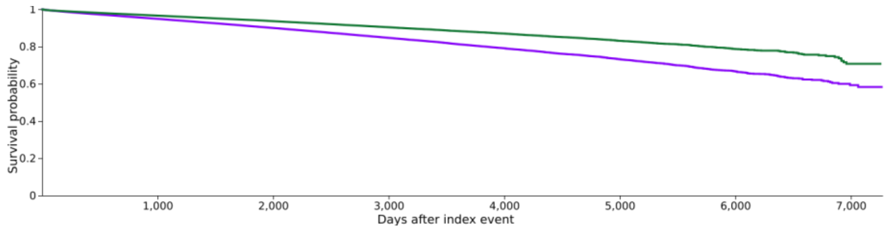

27,137 patients in Cohort 1 and 24,574 patients in Cohort 2 were excluded from results because they had the outcome prior to the time window.

Number of instances excluding patients with outcome prior to the time window

Cohort		Patients in cohort	Patients with outcome	Mean	Standard Deviation	Median
1	ED with T2DM	285,408	23,842	9.756	19.006	3
2	ED without T2DM	287,971	14,069	7.040	12.338	3

	t	df	p
Test Statistics	15.168	37909	0.000

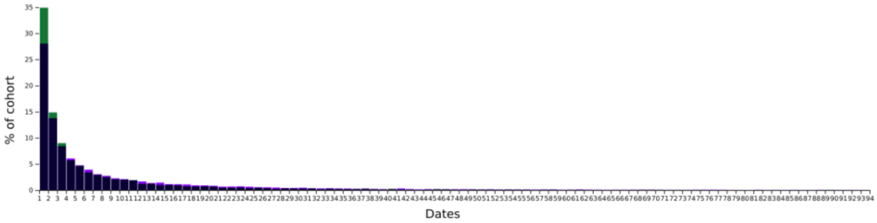

167 data points for Cohort 1 and 45 data points for Cohort 2 were omitted for display purposes.
27,137 patients in Cohort 1 and 24,574 patients in Cohort 2 were excluded from results because they had the outcome prior to the time window.

4 Peripheral artery disease

Risk analysis excluding patients with outcome prior to the time window

Cohort		Patients in cohort	Patients with outcome	Risk
1	ED with T2DM	280,898	23,016	0.082
2	ED without T2DM	286,968	17,473	0.061

	95% CI	z	p

Risk Difference	0.021	(0.020, 0.022)	30.819	0.000
Risk Ratio	1.346	(1.320, 1.371)	N/A	N/A
Odds Ratio	1.377	(1.349, 1.405)	N/A	N/A

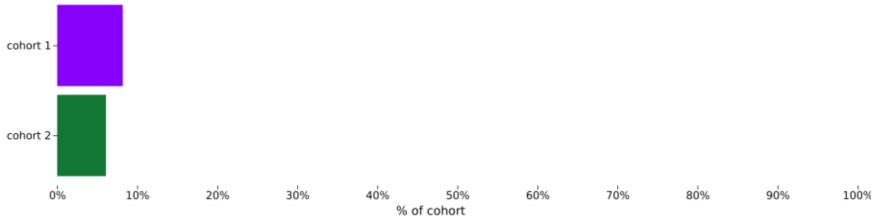

31,647 patients in Cohort 1 and 25,577 patients in Cohort 2 were excluded from results because they had the outcome prior to the time window.

Kaplan - Meier survival analysis excluding patients with outcome prior to the time window

Cohort		Patients in cohort	Patients with outcome	Median survival (days)	Survival probability at end of time window
1	ED with T2DM	280,898	23,016	--	67.00%
2	ED without T2DM	286,968	17,473	--	71.75%

	χ^2	df	p
Log-Rank Test	491.828	1	0.000

	Hazard Ratio	95% CI	χ^2	df	p
Hazard Ratio and Proportionality	1.249	(1.224, 1.274)	15.526	1	0.000

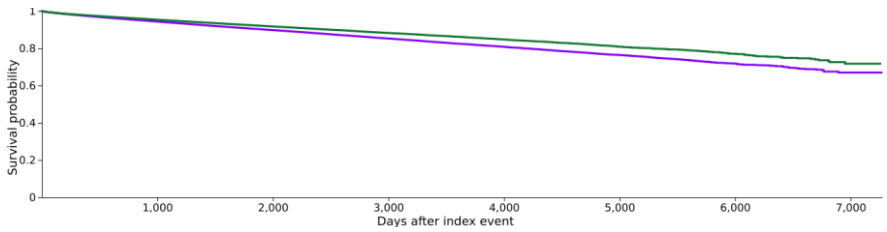

31,647 patients in Cohort 1 and 25,577 patients in Cohort 2 were excluded from results because they had the outcome prior to the time window.

Number of instances excluding patients with outcome prior to the time window

Cohort		Patients in cohort	Patients with outcome	Mean	Standard Deviation	Median
1	ED with T2DM	280,898	23,016	4.701	12.596	2
2	ED without T2DM	286,968	17,473	4.219	9.795	2

	t	df	p
Test Statistics	4.189	40487	0.000

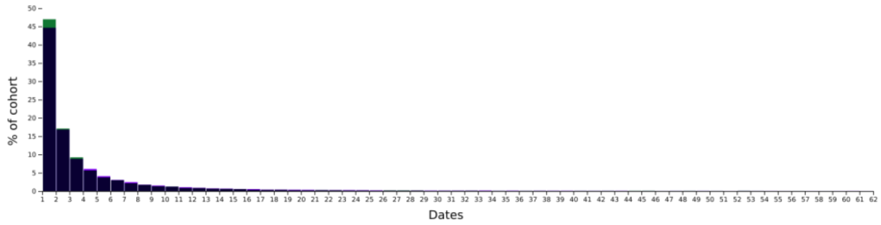

121 data points for Cohort 1 and 82 data points for Cohort 2 were omitted for display purposes.
31,647 patients in Cohort 1 and 25,577 patients in Cohort 2 were excluded from results because they had the outcome prior to the time window.

5 CVD

Risk analysis excluding patients with outcome prior to the time window

Cohort	Patients in cohort	Patients with outcome	Risk

1	ED with T2DM	201,440	48,769	0.242
2	ED without T2DM	207,930	38,055	0.183

		95% CI	z	p
Risk Difference	0.059	(0.057, 0.062)	46.232	0
Risk Ratio	1.323	(1.307, 1.339)	N/A	N/A
Odds Ratio	1.426	(1.405, 1.448)	N/A	N/A

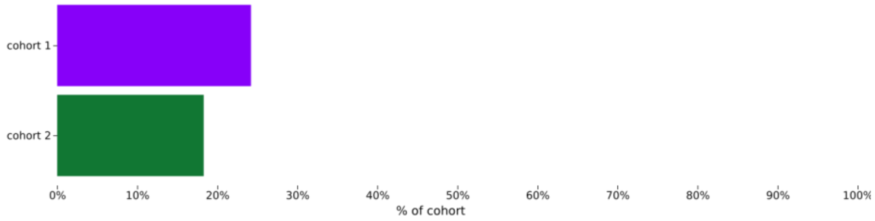

111,105 patients in Cohort 1 and 104,615 patients in Cohort 2 were excluded from results because they had the outcome prior to the time window.

Kaplan - Meier survival analysis excluding patients with outcome prior to the time window

Cohort		Patients in cohort	Patients with outcome	Median survival (days)	Survival probability at end of time window
1	ED with T2DM	201,440	48,769	3977	22.05%
2	ED without T2DM	207,930	38,055	4917	30.80%

	χ^2	df	p
Log-Rank Test	1166.514	1	0.000

	Hazard Ratio	95% CI	χ^2	df	p
Hazard Ratio and Proportionality	1.262	(1.246, 1.280)	4.541	1	0.033

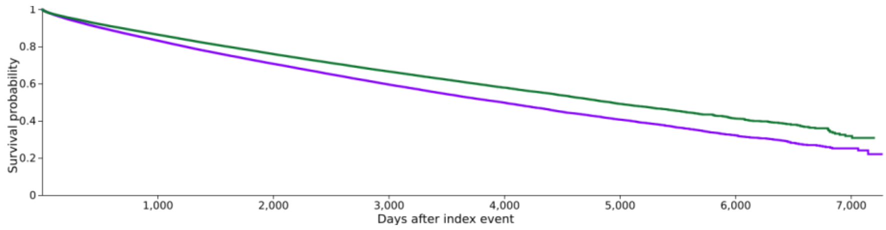

111,105 patients in Cohort 1 and 104,615 patients in Cohort 2 were excluded from results because they had the outcome prior to the time window.

Number of instances excluding patients with outcome prior to the time window

Cohort		Patients in cohort	Patients with outcome	Mean	Standard Deviation	Median
1	ED with T2DM	201,440	48,769	9.428	18.244	3
2	ED without T2DM	207,930	38,055	6.949	12.934	3

	t	df	p
Test Statistics	22.469	86822	0.000

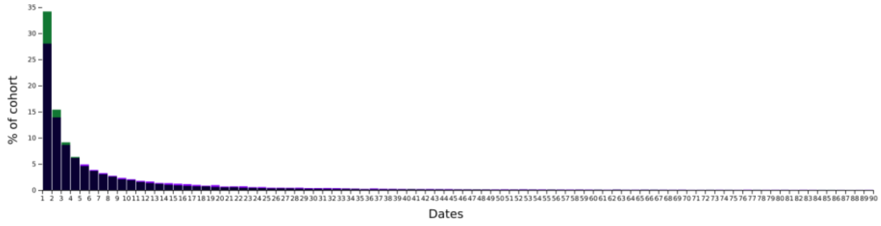

343 data points for Cohort 1 and 133 data points for Cohort 2 were omitted for display purposes.
111,105 patients in Cohort 1 and 104,615 patients in Cohort 2 were excluded from results because they had the outcome prior to the time window.

4 Appendix A – Text Representation of the Cohorts Definition

This section lists all terms used in the definitions of the two cohorts.

4.1.1 Query Criteria for Cohort 1 (query name: ED with T2DM)

Patients must have:
  all of the following:
    Age (Age) (between 20 and 80 years (most recent occurrence)); and
    Male (UMLS:HL7V3.0:Gender:M); and
    any of the following:
      Male erectile dysfunction (UMLS:ICD10CM:N52); or
      Vasculogenic erectile dysfunction (UMLS:ICD10CM:N52.0); and
    any of the following:
      Male erectile dysfunction, unspecified (UMLS:ICD10CM:N52.9); or
      Other male erectile dysfunction (UMLS:ICD10CM:N52.8); or
      Corporo-venous occlusive erectile dysfunction (UMLS:ICD10CM:N52.02); or
      Drugs used in erectile dysfunction (NLM:ATC:G04BE); or
      Erectile dysfunction due to diseases classified elsewhere (UMLS:ICD10CM:N52.1); or
      Erectile dysfunction due to arterial insufficiency (UMLS:ICD10CM:N52.01); or
      Combined arterial insufficiency and corporo-venous occlusive erectile dysfunction (UMLS:ICD10CM:N52.03); and
    Type 2 diabetes mellitus (UMLS:ICD10CM:E11).

Patients cannot have:
  any of the following:
    Diabetes mellitus due to underlying condition (UMLS:ICD10CM:E08); or
    Drug or chemical induced diabetes mellitus (UMLS:ICD10CM:E09); or
    Type 1 diabetes mellitus (UMLS:ICD10CM:E10); or
    Other specified diabetes mellitus (UMLS:ICD10CM:E13).

4.1.2 Query Criteria for Cohort 2 (query name: ED without T2DM)

Patients must have:
  all of the following:
    Age (Age) (between 20 and 80 years (most recent occurrence)); and
    Male (UMLS:HL7V3.0:Gender:M); and
    any of the following:
      Male erectile dysfunction (UMLS:ICD10CM:N52); or
      Vasculogenic erectile dysfunction (UMLS:ICD10CM:N52.0); or
      Male erectile dysfunction, unspecified (UMLS:ICD10CM:N52.9); or
      Other male erectile dysfunction (UMLS:ICD10CM:N52.8); or
      Corporo-venous occlusive erectile dysfunction (UMLS:ICD10CM:N52.02); or
      Drugs used in erectile dysfunction (NLM:ATC:G04BE); or
      Erectile dysfunction due to diseases classified elsewhere

(UMLS:ICD10CM:N52.1); or
Erectile dysfunction due to arterial insufficiency (UMLS:ICD10CM:N52.01); or
Combined arterial insufficiency and corporo-venous occlusive erectile dysfunction (UMLS:ICD10CM:N52.03).

Patients cannot have:
Diabetes mellitus (UMLS:ICD10CM:E08-E13).

5 Appendix B – Text Representation of the Analysis Setup

This section contains the Index Event definition for each cohort.

The index event for Cohort 1 (query name: ED with T2DM) is defined as the following:

Patients must have:
all of the following:
any of the following:
Male erectile dysfunction (UMLS:ICD10CM:N52); or
Vasculogenic erectile dysfunction (UMLS:ICD10CM:N52.0); and
any of the following:
Male erectile dysfunction, unspecified (UMLS:ICD10CM:N52.9); or
Other male erectile dysfunction (UMLS:ICD10CM:N52.8); or
Corporo-venous occlusive erectile dysfunction (UMLS:ICD10CM:N52.02); or
Drugs used in erectile dysfunction (NLM:ATC:G04BE); or
Erectile dysfunction due to diseases classified elsewhere (UMLS:ICD10CM:N52.1); or
Erectile dysfunction due to arterial insufficiency (UMLS:ICD10CM:N52.01); or
Combined arterial insufficiency and corporo-venous occlusive erectile dysfunction (UMLS:ICD10CM:N52.03); and
Type 2 diabetes mellitus (UMLS:ICD10CM:E11).

The index event for Cohort 2 (query name: ED without T2DM) is defined as the following:

Patients must have:
any of the following:
Male erectile dysfunction (UMLS:ICD10CM:N52); or
Vasculogenic erectile dysfunction (UMLS:ICD10CM:N52.0); or
Male erectile dysfunction, unspecified (UMLS:ICD10CM:N52.9); or
Other male erectile dysfunction (UMLS:ICD10CM:N52.8); or
Corporo-venous occlusive erectile dysfunction (UMLS:ICD10CM:N52.02); or
Drugs used in erectile dysfunction (NLM:ATC:G04BE); or
Erectile dysfunction due to diseases classified elsewhere (UMLS:ICD10CM:N52.1);

or
Erectile dysfunction due to arterial insufficiency (UMLS:ICD10CM:N52.01); or
Combined arterial insufficiency and corporo-venous occlusive erectile dysfunction (UMLS:ICD10CM:N52.03).

5.1.1

6 Appendix C – Text Representation of the Outcomes Definition

This analysis includes the following outcomes:

Ischemic hearth disease
Patients must have:
Ischemic heart diseases (UMLS:ICD10CM:I20-I25).

Stroke
Patients must have:
Cerebrovascular diseases (UMLS:ICD10CM:I60-I69).

Heart failure
Patients must have:
Heart failure (UMLS:ICD10CM:I50).

Peripheral artery disease
Patients must have:
Diseases of veins, lymphatic vessels and lymph nodes, not elsewhere classified (UMLS:ICD10CM:I80-I89).

CVD
Patients must have:
any of the following:
Ischemic heart diseases (UMLS:ICD10CM:I20-I25); or
Cerebrovascular diseases (UMLS:ICD10CM:I60-I69); or
Diseases of veins, lymphatic vessels and lymph nodes, not elsewhere classified (UMLS:ICD10CM:I80-I89); or
Heart failure (UMLS:ICD10CM:I50).

Appendix C: Propensity-score–matched comparative analysis for estimating the risk for different cardiovascular events among people with type 2 diabetes regarding the presence or absence of erectile dysfunction

7 Methods

The analysis process includes two main steps: 1) Defining the cohorts through query criteria; 2) Setting up and running the analysis. Setting up the analysis requires definitions for the index event, outcomes criteria, and the time frame. Compare outcomes supports four analyses: Measures of Association, Survival, Number of Instances and Lab result distribution. These analyses have additional options that are listed in the Outcomes Definitions and Analyses Specifications section below. Furthermore, characteristics of the cohorts that are balanced using propensity score matching are also included in the Propensity Score Matching section.

7.1 Cohorts definition

This section lists all terms used in the definitions of the two cohorts.

7.1.1

7.1.2 Query Criteria for Cohort 1 (query name: T2DM with ED)

This query was run on the network Global Collaborative Network with 166 HCO(s) queried and 166 HCO(s) responded. A total of 147 provider(s) responded with patients. The final cohort included 568,893 patients who matched the query criteria listed in the table below. For the text representation of the query criteria please see Appendix A.

Ungrouped terms				
must have		demographics	Age	Age (at least 18 years (most recent occurrence))
	and	demographics	UMLS:HL7V3.0:Gender:M	Male
	and	diagnosis	UMLS:ICD10CM:E11	Type 2 diabetes mellitus (between 20 and 80 years old at event)
	and any of	diagnosis	UMLS:ICD10CM:N52	Male erectile dysfunction
		diagnosis	UMLS:ICD10CM:N52.0	Vasculogenic erectile dysfunction
		diagnosis	UMLS:ICD10CM:N52.8	Other male erectile dysfunction

		diagnosis	UMLS:ICD10CM:N52.9	Male erectile dysfunction, unspecified
		diagnosis	UMLS:ICD10CM:N52.02	Corporo-venous occlusive erectile dysfunction
		medication	NLM:ATC:G04BE	Drugs used in erectile dysfunction
		diagnosis	UMLS:ICD10CM:N50.1	Vascular disorders of male genital organs
		diagnosis	UMLS:ICD10CM:N52.1	Erectile dysfunction due to diseases classified elsewhere
		diagnosis	UMLS:ICD10CM:N52.01	Erectile dysfunction due to arterial insufficiency
cannot have		diagnosis	UMLS:ICD10CM:E08	Diabetes mellitus due to underlying condition
	or	diagnosis	UMLS:ICD10CM:E09	Drug or chemical induced diabetes mellitus
	or	diagnosis	UMLS:ICD10CM:E10	Type 1 diabetes mellitus
	or	diagnosis	UMLS:ICD10CM:E13	Other specified diabetes mellitus

7.1.3

7.1.4 Query Criteria for Cohort 2 (query name: T2DM without ED)

This query was run on the network Global Collaborative Network with 161 HCO(s) queried and 161 HCO(s) responded. A total of 157 provider(s) responded with patients. The final cohort included 3,428,405 patients who matched the query criteria listed in the table below.

Ungrouped terms				
must have		demographics	Age	Age (at least 18 years (most recent occurrence))
	and	demographics	UMLS:HL7V3.0:Gender: M	Male
	and	diagnosis	UMLS:ICD10CM:E11	Type 2 diabetes mellitus (between 20 and 80 years old at event)
cannot have		diagnosis	UMLS:ICD10CM:E08	Diabetes mellitus due to underlying condition

or	diagnosis	UMLS:ICD10CM:E09	Drug or chemical induced diabetes mellitus
or	diagnosis	UMLS:ICD10CM:E10	Type 1 diabetes mellitus
or	diagnosis	UMLS:ICD10CM:E13	Other specified diabetes mellitus
or	diagnosis	UMLS:ICD10CM:N52	Male erectile dysfunction
or	diagnosis	UMLS:ICD10CM:N52.0	Vasculogenic erectile dysfunction
or	diagnosis	UMLS:ICD10CM:N52.8	Other male erectile dysfunction
or	diagnosis	UMLS:ICD10CM:N52.02	Corporo-venous occlusive erectile dysfunction
or	diagnosis	UMLS:ICD10CM:N52.9	Male erectile dysfunction, unspecified
or	medication	NLM:ATC:G04BE	Drugs used in erectile dysfunction
or	diagnosis	UMLS:ICD10CM:N52.01	Erectile dysfunction due to arterial insufficiency
or	diagnosis	UMLS:ICD10CM:N50.1	Vascular disorders of male genital organs
or	diagnosis	UMLS:ICD10CM:N52.1	Erectile dysfunction due to diseases classified elsewhere

7.2 Analysis Setup

This section contains the Index Event and Time Window definitions and a list of selected outcomes and the analyses.

7.2.1 Index Event & Time Window Definitions

The index event defines the point in time when each patient in the cohort enters the analysis. To define an index event for the cohort, one or more criteria for the cohort must be selected. The index date for each patient within a cohort is the day on which the patient first met the selected criteria for the cohort (listed in the table below).

As the index event defines the earliest time point after which outcomes are analyzed, the time window defines the duration during which outcomes are analyzed. The time window can start on the same day as the index event or at any specified time interval after the index event. The time window can end any time after the start date. Outcomes

are defined as diagnoses, medications, procedures, or laboratory values that happened in the time window starting after the first occurrence of the index event.

7.2.2 Time Window Used in this Analysis

This analysis included outcomes that occurred in the time window that started 1 day after the first occurrence of the index event. Since no end date was specified all outcomes after the first occurrence of the index event were included.

The index event only includes events that occurred up to 20 years ago. Patients whose index event occurred 20 years or more ago are excluded. In this analysis, 0 patients in Cohort 1 and 0 patients in Cohort 2 were excluded because they met the index event more than 20 years ago.

7.2.3 Index Events Used in this Analysis

Index events for the Compare Outcomes analysis were derived from the cohort definitions. Index events were defined separately for each cohort and were based on the criteria used in the original cohort definition. Please see Appendix B for the text representation of the index event definition.

The index event for Cohort 1 (query name: T2DM with ED) was defined as the following:

Ungrouped terms				
must have		diagnosis	UMLS:ICD10CM:E11	Type 2 diabetes mellitus (between 20 and 80 years old at event)
	and any of	diagnosis	UMLS:ICD10CM:N52	Male erectile dysfunction
		diagnosis	UMLS:ICD10CM:N52.0	Vasculogenic erectile dysfunction
		diagnosis	UMLS:ICD10CM:N52.8	Other male erectile dysfunction
		diagnosis	UMLS:ICD10CM:N52.9	Male erectile dysfunction, unspecified
		diagnosis	UMLS:ICD10CM:N52.02	Corporo-venous occlusive erectile dysfunction
		medication	NLM:ATC:G04BE	Drugs used in erectile dysfunction

	diagnosis	UMLS:ICD10CM:N50.1	Vascular disorders of male genital organs
	diagnosis	UMLS:ICD10CM:N52.1	Erectile dysfunction due to diseases classified elsewhere
	diagnosis	UMLS:ICD10CM:N52.01	Erectile dysfunction due to arterial insufficiency

The index event for Cohort 2 (query name: T2DM without ED) was defined as the following:

Ungrouped terms			
must have	diagnosis	UMLS:ICD10CM:E11	Type 2 diabetes mellitus (between 20 and 80 years old at event)

7.2.4 Analyses Specifications

The Compare Outcomes Analytic supports four types of analyses: Measure of Association, Survival, Number of Instances, and Lab result distribution. The first three analyses support the “exclude patients with outcomes prior to the window” setting. This option can exclude patients from the analysis if they are not at risk for an outcome (e.g., if the outcome is a chronic disease). When "exclude patients with the outcome prior to the time window" is not checked, all patients in the cohort are included in the analysis, regardless of whether they had the outcome prior to the time window. When "exclude patients with the outcome prior to the time window" is checked, patients are excluded from the analysis if their record includes the outcome prior to the beginning of the time window. This selection will exclude all patients with the outcome prior to the index event. If the start of the time window for the analysis falls some days after the index event, patients will also be excluded if they have the outcome between the index event and the start of the time window.

7.2.5 Measure of Association Analysis

The Measure of Association Analysis calculates and compares the fraction of patients with the selected outcome. The output summary includes: Patients in each Cohort (count of patients meeting query criteria); Patients with Outcome in each Cohort (of the patients in the cohort, count of patients that had the outcome in the time window); and Risk (the fraction of patients in the cohort that have the outcome in the time window, i.e. Patients with Outcome / Patients in Cohort). In addition, Risk Difference (the difference in the risks in Cohort 1 and Cohort 2), Risk Ratio (the ratio of the risks in

Cohort 1 and Cohort 2), and Odds Ratio (the ratio of the odds in Cohort 1 and Cohort 2). The bar chart shows the risk of the outcome for the both cohorts.

7.2.6 Survival Analysis

The Kaplan-Meier Analysis estimates probability of the outcome at a respective time interval (daily time interval is used in this analysis). In order to account for the patients who exited the cohort during the analysis period, and therefore should not be included in the analysis, censoring is applied. In this analysis, patients are removed from the analysis (censored) after the last fact in their record.

The output summary includes: Patients in each Cohort (count of patients meeting query criteria); Patients with Outcome (of the patients in the cohort, count of patients that had the outcome in the time window); Median Survival (the number of days when the survival drops below 50%; the "-" indicates that survival does not drop below 50% during the time window); and Survival Probability at End of Time Window (the % survival at the end of the time window). In addition, Log-Rank test, Hazard Ratio and test for Proportionality.

7.2.7 Number of Instances Analysis

The Number of Instances Analysis calculates how many times the outcome occurred in the time window. This analysis includes two additional settings: include patients with zero instances; the definition of an instance.

Selecting to exclude patients with zero instances will remove these patients from the calculations for mean number of instances, standard deviation, or median. The histogram showing the distribution of patients by number of instances will not contain a bar for zero. Alternatively, by selecting to include patients with zero instances, the mean, standard deviation, and median for number of instances will reflect these patients. The histogram will contain a bar for zero patients.

The definition of an instance affects how counts are analyzed. By selecting Date, each calendar date on which any of the terms selected in the outcome are recorded will represent one instance. For example, if the outcome is "Med A or Med B," and a patient has "Med A" on January 3, then both medications on January 4, then "Med B" on January 6, then that patient is considered to have three instances– January 3, January 4, and January 6. Note that if an outcome occurs across several dates (e.g. Visit: inpatient encounter), then only the start date is tracked for the purpose of counting instances. A patient who begins at stay on January 1, ends that stay on January 3, begins another stay on January 10, and ends that stay on January 15, is considered to have two instances of the outcome.

Selecting Visit as an Instance will count any visit that includes the outcome as one instance, regardless of how many times it occurred. For instance, consider a patient administered an analgesic on each of the three days that make up an inpatient stay following some index event. If analgesic is an outcome, these three administrations will represent only one instance, because all three are associated with the same visit.

The output summary includes: Patients in Cohort (count of patients meeting query criteria); Patients with Outcome (of the patients in the cohort, count of patients that had the outcome in the time window); Mean (mean of the counts); Standard Deviation (standard deviation of the counts); Median (median of the counts); and Median (1+ instances) when patients with zero instances included in the analysis. In addition, T-Test statistics testing for the difference between the cohorts is included.

7.2.8 Laboratory Results Analysis

Lab Results can be included in the analysis only for the outcomes that are labs. Only the most recent lab values in the time window are included. For the lab results that are numeric, the outcome summary includes: Patients in Cohort (count of patients meeting query criteria); Patients with Outcome (of the patients in the cohort, count of patients that had the outcome in the time window); Mean (mean of the counts); and Standard Deviation (the standard deviation for lab values across patients in the cohort). In addition, T-Test statistics testing for the difference between the cohorts is included.

For the non-numeric lab results, three values are reported: counts of Negative; Positives; and Unknowns.

The counts are represented in the bar chart as percentages of the total counts.

7.2.9 Outcome Definitions

Table below outlines the definitions for each outcome and the analysis specifications. For outcome definitions consisting of more than one term, at least one term must match. Please see Appendix C for the text representation of the outcome definitions.

Ischemic hearth disease		
Outcome definition		
Diagnosis	UMLS:ICD10CM:I20-I25	Ischemic heart diseases

Settings for the performed analyses		
Kaplan - Meier survival analysis		excluding patients with outcome prior to the time window
Risk analysis		excluding patients with outcome prior to the time window
Number of instances analysis		excluding patients with outcome prior to the time window<br>excluding patients with zero outcomes<br>counts are grouped by date
**Stroke**		
Outcome definition		
Diagnosis	UMLS:ICD10CM:I60-I69	Cerebrovascular diseases
Settings for the performed analyses		
Risk analysis		excluding patients with outcome prior to the time window
Kaplan - Meier survival analysis		excluding patients with outcome prior to the time window
Number of instances analysis		excluding patients with outcome prior to the time window<br>excluding patients with zero outcomes<br>counts are grouped by date
**Heart failure**		
Outcome definition		
Diagnosis	UMLS:ICD10CM:I50	Heart failure
Settings for the performed analyses		
Risk analysis		excluding patients with outcome prior to the time window
Kaplan - Meier survival analysis		excluding patients with outcome prior to the time window
Number of instances analysis		excluding patients with outcome prior to the time window<br>excluding patients with zero outcomes<br>counts are grouped by date
**Peripheral artery disease**		
Outcome definition		
Diagnosis	UMLS:ICD10CM:I80-I89	Diseases of veins, lymphatic vessels and lymph nodes, not elsewhere classified

Settings for the performed analyses		
Risk analysis		excluding patients with outcome prior to the time window
Kaplan - Meier survival analysis		excluding patients with outcome prior to the time window
Number of instances analysis		excluding patients with outcome prior to the time window<br>excluding patients with zero outcomes<br>counts are grouped by date
CVD		
Outcome definition		
Diagnosis	UMLS:ICD10CM:I20-I25	Ischemic heart diseases
Diagnosis	UMLS:ICD10CM:I60-I69	Cerebrovascular diseases
Diagnosis	UMLS:ICD10CM:I80-I89	Diseases of veins, lymphatic vessels and lymph nodes, not elsewhere classified
Diagnosis	UMLS:ICD10CM:I50	Heart failure
Settings for the performed analyses		
Risk analysis		excluding patients with outcome prior to the time window
Kaplan - Meier survival analysis		excluding patients with outcome prior to the time window
Number of instances analysis		excluding patients with outcome prior to the time window<br>excluding patients with zero outcomes<br>counts are grouped by date

7.3

7.4 Propensity Score Matching

Propensity score matching was performed on 4 characteristic(s). In the Demographics category patients were matched on Age at Index characteristic(s). In the Diagnosis category patients were matched on Hypertensive diseases, Disorders of lipoprotein metabolism and other lipidemias, Nicotine dependence, unspecified, uncomplicated characteristic(s). Characteristics of the cohorts before and after matching are summarized in the table below.

Cohort 1 and cohort 2 patient count before and after propensity score matching		
Cohort	Patient count before matching	Patient count after matching
1 - T2DM with ED	559,583	555,508
2 - T2DM without ED	3,412,080	555,508

Propensity score density function - Before and after matching (cohort 1 - purple, cohort 2 - green)

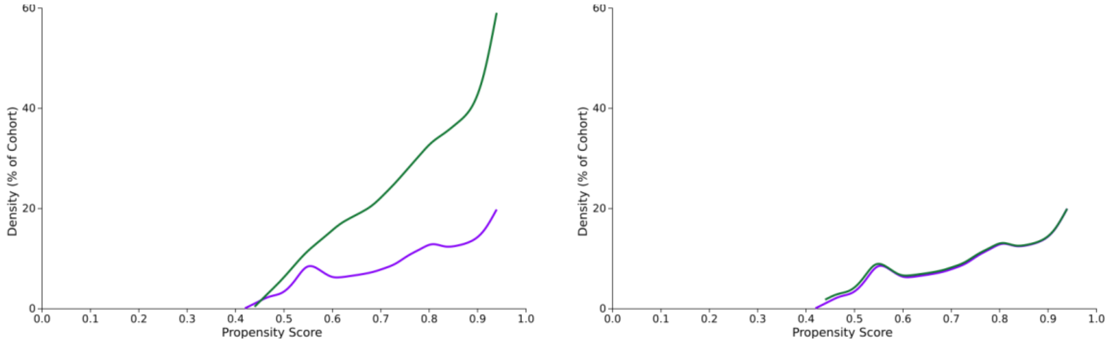

Cohort 1 (N = 559,583) and cohort 2 (N = 3,412,080) characteristics before propensity score matching

Demographics

Cohort			Mean ± SD	Patients	% of Cohort	P-Value	Std diff.
1<br>2	AI	Age at Index	61.1 +/- 10.4<br>59.0 +/- 12.7	555,508<br>3,340,610	100%<br>100%	<0.001	0.183

Diagnosis

Cohort			Mean ± SD	Patients	% of Cohort	P-Value	Std diff.
1<br>2	I10-I1A	Hypertensive diseases		367,715<br>652,169	66.2%<br>19.5%	<0.001	1.069
1<br>2	I50	Heart failure		61,364<br>110,545	11.0%<br>3.3%	<0.001	0.303
1<br>2	I20-I25	Ischemic heart diseases		151,294<br>279,758	27.2%<br>8.4%	<0.001	0.509
1<br>2	I60-I69	Cerebrovascular diseases		55,890<br>118,511	10.1%<br>3.5%	<0.001	0.261

Cohort	Code	Description	Mean ± SD	Patients	% of Cohort	P-Value	Std diff.
1<br>2	I70-I79	Diseases of arteries, arterioles and capillaries		79,060<br>128,701	14.2%<br>3.9%	<0.001	0.368
1<br>2	E78	Disorders of lipoprotein metabolism and other lipidemias		336,630<br>475,858	60.6%<br>14.2%	<0.001	1.091
1<br>2	N18	Chronic kidney disease (CKD)		73,962<br>119,487	13.3%<br>3.6%	<0.001	0.356
1<br>2	F17.200	Nicotine dependence, unspecified, uncomplicated		60,804<br>118,741	10.9%<br>3.6%	<0.001	0.288
Procedure							
Cohort			Mean ± SD	Patients	% of Cohort	P-Value	Std diff.
1<br>2	1018513	Smoking and tobacco use cessation counseling visit		7,166<br>10,311	1.3%<br>0.3%	<0.001	0.110
Laboratory							
Cohort			Mean ± SD	Patients	% of Cohort	P-Value	Std diff.
1<br>2	9024	Creatinine [Mass/volume] in Serum, Plasma or Blood	1.4 +/- 4.2<br>1.4 +/- 5.4	419,804<br>1,081,815	75.6%<br>32.4%	<0.001	0.012
1<br>2		0 - 0 mg/dL		419,820<br>1,081,912	75.6%<br>32.4%	<0.001	0.961
1<br>2	9000	Cholesterol [Mass/volume] in Serum or Plasma	164.4 +/- 46.7<br>172.2 +/- 49.5	326,523<br>600,666	58.8%<br>18.0%	<0.001	0.162
1<br>2		0 - 0 mg/dL		326,529<br>600,686	58.8%<br>18.0%	<0.001	0.924
1<br>2	9002	Cholesterol in LDL [Mass/volume] in Serum or Plasma	91.3 +/- 37.2<br>98.0 +/- 38.4	324,562<br>588,710	58.4%<br>17.6%	<0.001	0.178
1<br>2		0 - 0 mg/dL		324,584<br>588,767	58.4%<br>17.6%	<0.001	0.926
1<br>2	9001	Cholesterol in HDL [Mass/volume] in Serum or Plasma	40.3 +/- 14.6<br>40.2 +/- 14.2	328,183<br>594,834	59.1%<br>17.8%	0.006	0.006

1<br>2		0 - 0 mg/dL		328,186<br>594,864	59.1%<br>17.8%	<0.001	0.937
1<br>2	9004	Triglyceride [Mass/volume] in Serum, Plasma or Blood	170.1 +/- 163.6<br>181.7 +/- 199.6	326,299<br>595,689	58.7%<br>17.8%	<0.001	0.064
1<br>2		0 - 0 mg/dL		326,299<br>595,690	58.7%<br>17.8%	<0.001	0.928
1<br>2	9037	Hemoglobin A1c/Hemoglobin.total in Blood	7.1 +/- 1.8<br>7.1 +/- 1.8	340,933<br>624,831	61.4%<br>18.7%	<0.001	0.010
1<br>2		0 - 0 %		340,938<br>624,873	61.4%<br>18.7%	<0.001	0.967
1<br>2	9083	BMI	32.2 +/- 6.6<br>32.5 +/- 7.5	350,386<br>945,040	63.1%<br>28.3%	<0.001	0.044
1<br>2		0 - 0 kg/m2		350,452<br>945,777	63.1%<br>28.3%	<0.001	0.745
1<br>2	9085	Blood Pressure, Systolic	131.9 +/- 18.7<br>133.5 +/- 20.0	398,775<br>1,085,031	71.8%<br>32.5%	<0.001	0.083
1<br>2		0 - 0 mm[Hg]		398,777<br>1,085,078	71.8%<br>32.5%	<0.001	0.856
1<br>2	9086	Blood Pressure, Diastolic	77.2 +/- 11.7<br>78.3 +/- 12.7	398,991<br>1,085,248	71.8%<br>32.5%	<0.001	0.093
1<br>2		0 - 0 mm[Hg]		399,008<br>1,085,340	71.8%<br>32.5%	<0.001	0.857
1<br>2	88031-0	Smokeless tobacco status		0<br>0	0%<br>0%	--	--
1<br>2		0 - 0 units		0<br>0	0%<br>0%	--	--

Cohort 1 (N = 555,508) and cohort 2 (N = 555,508) characteristics after propensity score matching

Demographics

Cohort			Mean ± SD	Patients	% of Cohort	P-Value	Std diff.
1<br>2	AI	Age at Index	61.1 +/- 10.4<br>61.0 +/- 10.4	555,508<br>555,508	100%<br>100%	<0.001	0.015

Diagnosis							
Cohort			Mean ± SD	Patients	% of Cohort	P-Value	Std diff.
1<br>2	I10-I1A	Hypertensive diseases		367,715<br>367,437	66.2%<br>66.1%	0.577	0.001
1<br>2	I50	Heart failure		61,364<br>52,575	11.0%<br>9.5%	<0.001	0.052
1<br>2	I20-I25	Ischemic heart diseases		151,294<br>136,268	27.2%<br>24.5%	<0.001	0.062
1<br>2	I60-I69	Cerebrovascular diseases		55,890<br>50,330	10.1%<br>9.1%	<0.001	0.034
1<br>2	I70-I79	Diseases of arteries, arterioles and capillaries		79,060<br>61,901	14.2%<br>11.1%	<0.001	0.093
1<br>2	E78	Disorders of lipoprotein metabolism and other lipidemias		336,630<br>336,908	60.6%<br>60.6%	0.589	0.001
1<br>2	N18	Chronic kidney disease (CKD)		73,962<br>50,858	13.3%<br>9.2%	<0.001	0.132
1<br>2	F17.200	Nicotine dependence, unspecified, uncomplicated		60,804<br>62,530	10.9%<br>11.3%	<0.001	0.010

Procedure							
Cohort			Mean ± SD	Patients	% of Cohort	P-Value	Std diff.
1<br>2	1018513	Smoking and tobacco use cessation counseling visit		7,166<br>6,242	1.3%<br>1.1%	<0.001	0.015

Laboratory							
Cohort			Mean ± SD	Patients	% of Cohort	P-Value	Std diff.
1<br>2	9024	Creatinine [Mass/volume] in Serum, Plasma or Blood	1.4 +/- 4.2<br>1.3 +/- 4.4	419,804<br>335,761	75.6%<br>60.4%	0.006	0.006
1<br>2		0 - 0 mg/dL		419,820<br>335,771	75.6%<br>60.4%	<0.001	0.329

1<br>2	9000	Cholesterol [Mass/volume] in Serum or Plasma	164.4 +/- 46.7<br>170.6 +/- 48.1	326,523<br>240,489	58.8%<br>43.3%	<0.001	0.131
1<br>2		0 - 0 mg/dL		326,529<br>240,492	58.8%<br>43.3%	<0.001	0.314
1<br>2	9002	Cholesterol in LDL [Mass/volume] in Serum or Plasma	91.3 +/- 37.2<br>96.4 +/- 38.3	324,562<br>237,943	58.4%<br>42.8%	<0.001	0.136
1<br>2		0 - 0 mg/dL		324,584<br>237,955	58.4%<br>42.8%	<0.001	0.316
1<br>2	9001	Cholesterol in HDL [Mass/volume] in Serum or Plasma	40.3 +/- 14.6<br>40.3 +/- 14.3	328,183<br>239,520	59.1%<br>43.1%	0.472	0.002
1<br>2		0 - 0 mg/dL		328,186<br>239,525	59.1%<br>43.1%	<0.001	0.323
1<br>2	9004	Triglyceride [Mass/volume] in Serum, Plasma or Blood	170.1 +/- 163.6<br>178.3 +/- 171.5	326,299<br>238,295	58.7%<br>42.9%	<0.001	0.049
1<br>2		0 - 0 mg/dL		326,299<br>238,295	58.7%<br>42.9%	<0.001	0.321
1<br>2	9037	Hemoglobin A1c/Hemoglobin.total in Blood	7.1 +/- 1.8<br>6.8 +/- 1.5	340,933<br>223,255	61.4%<br>40.2%	<0.001	0.203
1<br>2		0 - 0 %		340,938<br>223,261	61.4%<br>40.2%	<0.001	0.434
1<br>2	9083	BMI	32.2 +/- 6.6<br>32.6 +/- 7.3	350,386<br>278,869	63.1%<br>50.2%	<0.001	0.064
1<br>2		0 - 0 kg/m2		350,452<br>279,006	63.1%<br>50.2%	<0.001	0.262
1<br>2	9085	Blood Pressure, Systolic	131.9 +/- 18.7<br>133.3 +/- 19.7	398,775<br>318,288	71.8%<br>57.3%	<0.001	0.070
1<br>2		0 - 0 mm[Hg]		398,777<br>318,294	71.8%<br>57.3%	<0.001	0.306
1<br>2	9086	Blood Pressure, Diastolic	77.2 +/- 11.7<br>78.3 +/- 12.4	398,991<br>318,379	71.8%<br>57.3%	<0.001	0.094

1<br>2		0 - 0 mm[Hg]	399,008<br>318,395	71.8%<br>57.3%	<0.001	0.307
1<br>2	88031-0	Smokeless tobacco status	0<br>0	0%<br>0%	--	--
1<br>2		0 - 0 units	0<br>0	0%<br>0%	--	--

8 Results

Results are summarized in the tables below. Outcomes analysis was performed on the cohorts after propensity score matching.

Follow-up Time (Before Matching)				
Cohort	Mean Follow-up (Days)	Standard Deviation	Median Follow-up (Days)	Interquartile Range
T2DM with ED	1528.561	1355.267	1160	1850
T2DM without ED	1233.571	1361.267	768	1858

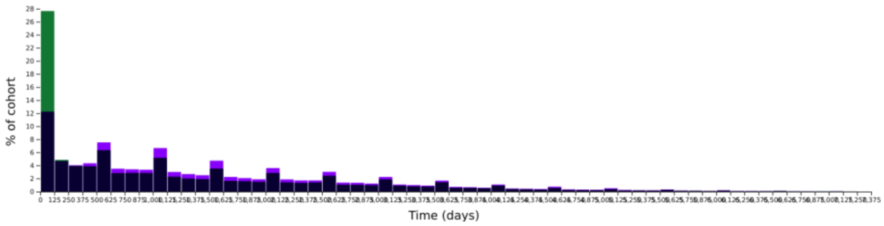

4,075 patients in Cohort 1 and 71,470 patients in Cohort 2 were excluded because they met the index event more than 20 years ago.

Follow-up Time (After Matching)				
Cohort	Mean Follow-up (Days)	Standard Deviation	Median Follow-up (Days)	Interquartile Range

T2DM with ED	1528.561	1355.267	1160	1850
T2DM without ED	1368.141	1350.528	960	1859

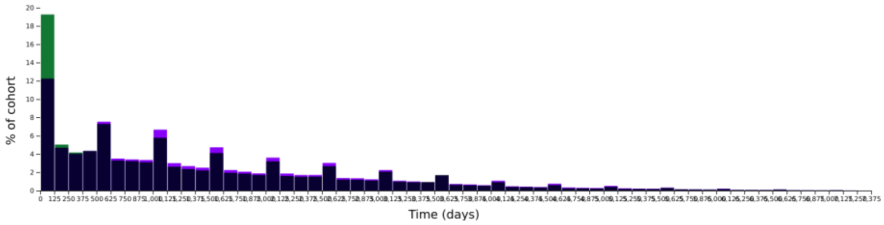

1 Ischemic hearth disease

Risk analysis excluding patients with outcome prior to the time window

Cohort		Patients in cohort	Patients with outcome	Risk
1	T2DM with ED	385,971	65,253	0.169
2	T2DM without ED	382,050	59,504	0.156

		95% CI	z	p
Risk Difference	0.013	(0.012, 0.015)	15.815	0.000
Risk Ratio	1.085	(1.074, 1.097)	N/A	N/A
Odds Ratio	1.103	(1.090, 1.116)	N/A	N/A

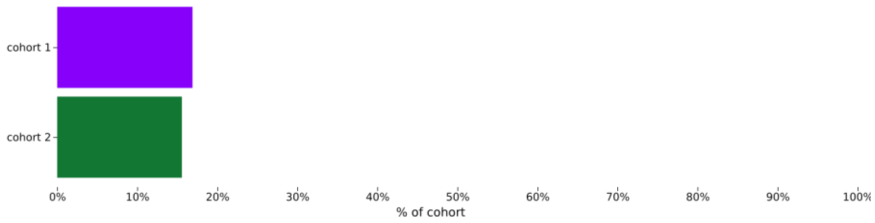

169,537 patients in Cohort 1 and 173,458 patients in Cohort 2 were excluded from results because they had the outcome prior to the time window.

Kaplan - Meier survival analysis excluding patients with outcome prior to the time window

Cohort		Patients in cohort	Patients with outcome	Median survival (days)	Survival probability at end of time window
1	T2DM with ED	385,971	65,253	5733	39.00%
2	T2DM without ED	382,050	59,504	5845	40.86%

	χ^2	df	p
Log-Rank Test	71.241	1	0.000

	Hazard Ratio	95% CI	χ^2	df	p
Hazard Ratio and Proportionality	0.953	(0.943, 0.964)	160.843	1	0.000

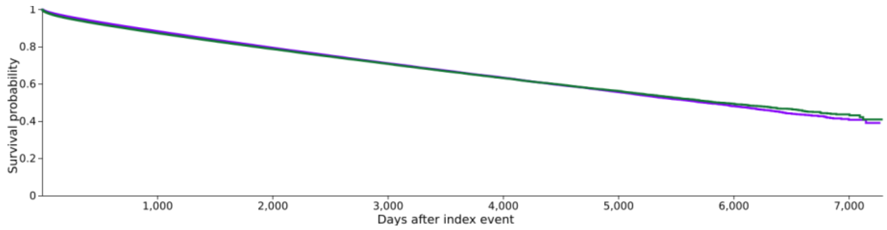

169,537 patients in Cohort 1 and 173,458 patients in Cohort 2 were excluded from results because they had the outcome prior to the time window.

Number of instances excluding patients with outcome prior to the time window

Cohort		Patients in cohort	Patients with outcome	Mean	Standard Deviation	Median
1	T2DM with ED	385,971	65,253	7.911	14.450	3
2	T2DM without ED	382,050	59,504	6.920	13.320	2

	t	df	p
Test Statistics	12.562	124755	0.000

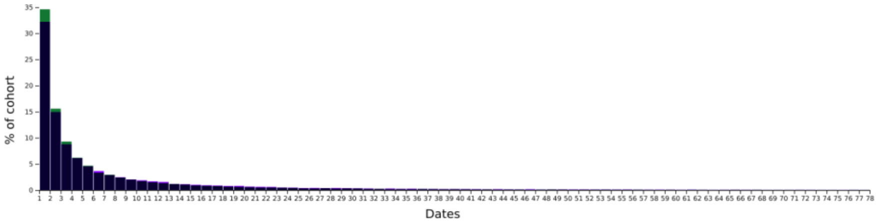

382 data points for Cohort 1 and 286 data points for Cohort 2 were omitted for display purposes.
169,537 patients in Cohort 1 and 173,458 patients in Cohort 2 were excluded from results because they had the outcome prior to the time window.

2 Stroke

Risk analysis excluding patients with outcome prior to the time window

Cohort		Patients in cohort	Patients with outcome	Risk
1	T2DM with ED	493,588	44,683	0.091
2	T2DM without ED	490,722	41,161	0.084

	95% CI	z	p

Risk Difference	0.007	(0.006, 0.008)	11.689	0.000
Risk Ratio	1.079	(1.066, 1.093)	N/A	N/A
Odds Ratio	1.087	(1.072, 1.102)	N/A	N/A

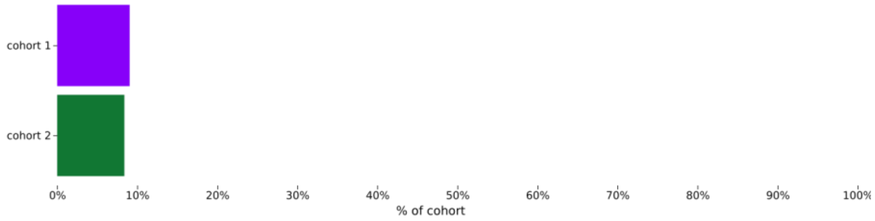

61,920 patients in Cohort 1 and 64,786 patients in Cohort 2 were excluded from results because they had the outcome prior to the time window.

Kaplan - Meier survival analysis excluding patients with outcome prior to the time window

Cohort		Patients in cohort	Patients with outcome	Median survival (days)	Survival probability at end of time window
1	T2DM with ED	493,588	44,683	--	55.25%
2	T2DM without ED	490,722	41,161	--	58.31%

	χ^2	df	p
Log-Rank Test	23.270	1	0.000

	Hazard Ratio	95% CI	χ^2	df	p
Hazard Ratio and Proportionality	0.968	(0.955, 0.981)	116.012	1	0.000

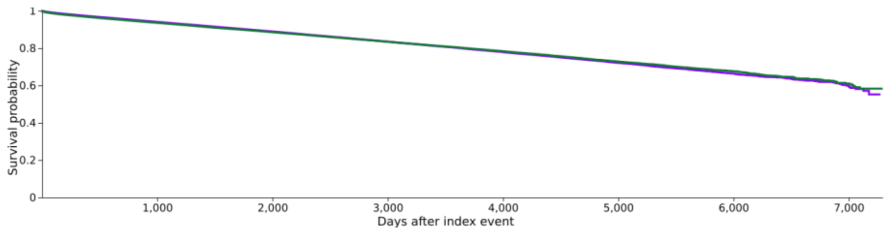

61,920 patients in Cohort 1 and 64,786 patients in Cohort 2 were excluded from results because they had the outcome prior to the time window.

Number of instances excluding patients with outcome prior to the time window

Cohort		Patients in cohort	Patients with outcome	Mean	Standard Deviation	Median
1	T2DM with ED	493,588	44,683	5.543	11.258	2
2	T2DM without ED	490,722	41,161	5.220	11.969	2

	t	df	p
Test Statistics	4.079	85842	0.000

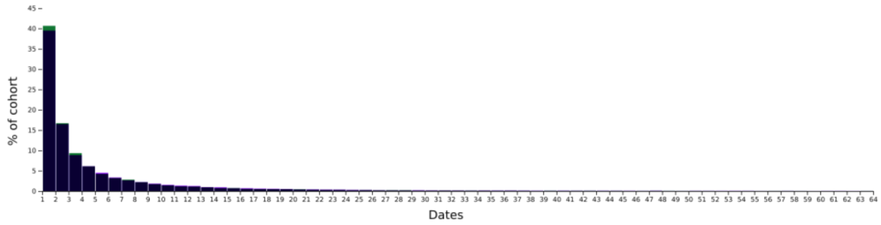

253 data points for Cohort 1 and 214 data points for Cohort 2 were omitted for display purposes.
61,920 patients in Cohort 1 and 64,786 patients in Cohort 2 were excluded from results because they had the outcome prior to the time window.

3 Heart failure

Risk analysis excluding patients with outcome prior to the time window

Cohort	Patients in cohort	Patients with outcome	Risk

1	T2DM with ED	484,863	49,149	0.101
2	T2DM without ED	482,451	48,350	0.100

		95% CI	z	p
Risk Difference	0.001	(-0.000, 0.002)	1.877	0.060
Risk Ratio	1.011	(0.999, 1.024)	N/A	N/A
Odds Ratio	1.013	(0.999, 1.026)	N/A	N/A

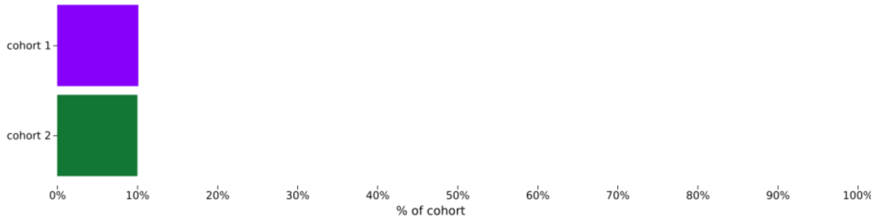

70,645 patients in Cohort 1 and 73,057 patients in Cohort 2 were excluded from results because they had the outcome prior to the time window.

Kaplan - Meier survival analysis excluding patients with outcome prior to the time window

Cohort		Patients in cohort	Patients with outcome	Median survival (days)	Survival probability at end of time window
1	T2DM with ED	484,863	49,149	--	53.67%
2	T2DM without ED	482,451	48,350	--	53.30%

	χ^2	df	p
Log-Rank Test	236.110	1	0.000

	Hazard Ratio	95% CI	χ^2	df	p
Hazard Ratio and Proportionality	0.906	(0.895, 0.918)	96.648	1	0.000

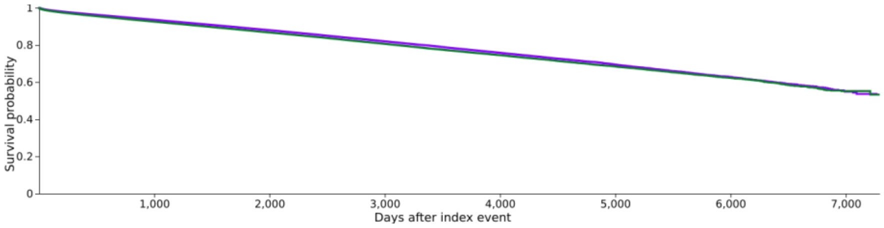

70,645 patients in Cohort 1 and 73,057 patients in Cohort 2 were excluded from results because they had the outcome prior to the time window.

Number of instances excluding patients with outcome prior to the time window

Cohort		Patients in cohort	Patients with outcome	Mean	Standard Deviation	Median
1	T2DM with ED	484,863	49,149	9.446	17.905	3
2	T2DM without ED	482,451	48,350	8.422	15.300	3

	t	df	p
Test Statistics	9.601	97497	0.000

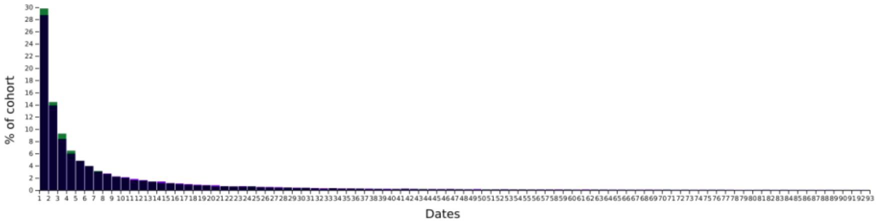

333 data points for Cohort 1 and 262 data points for Cohort 2 were omitted for display purposes.
70,645 patients in Cohort 1 and 73,057 patients in Cohort 2 were excluded from results because they had the outcome prior to the time window.

4 Peripheral artery disease

Risk analysis excluding patients with outcome prior to the time window

Cohort		Patients in cohort	Patients with outcome	Risk
1	T2DM with ED	497,508	43,283	0.087
2	T2DM without ED	507,938	38,386	0.076

		95% CI	z	p
Risk Difference	0.011	(0.010, 0.012)	20.971	0.000
Risk Ratio	1.151	(1.136, 1.166)	N/A	N/A
Odds Ratio	1.166	(1.149, 1.182)	N/A	N/A

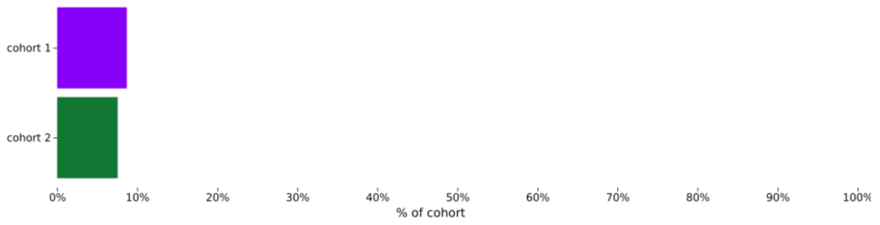

58,000 patients in Cohort 1 and 47,570 patients in Cohort 2 were excluded from results because they had the outcome prior to the time window.

Kaplan - Meier survival analysis excluding patients with outcome prior to the time window

Cohort		Patients in cohort	Patients with outcome	Median survival (days)	Survival probability at end of time window
1	T2DM with ED	497,508	43,283	--	59.61%
2	T2DM without ED	507,938	38,386	--	69.18%

	χ^2	df	p
Log-Rank Test	31.159	1	0.000

	Hazard Ratio	95% CI	χ^2	df	p
Hazard Ratio and Proportionality	1.040	(1.026, 1.054)	38.841	1	0.000

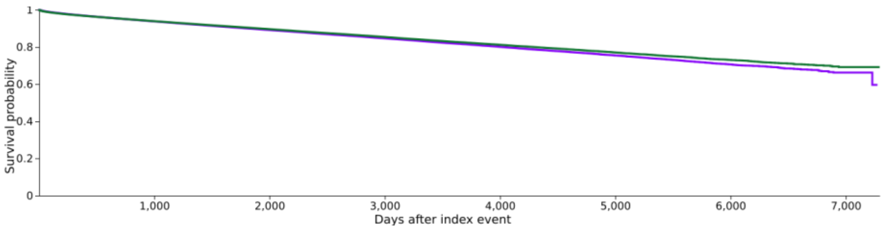

58,000 patients in Cohort 1 and 47,570 patients in Cohort 2 were excluded from results because they had the outcome prior to the time window.

Number of instances excluding patients with outcome prior to the time window

Cohort		Patients in cohort	Patients with outcome	Mean	Standard Deviation	Median
1	T2DM with ED	497,508	43,283	4.687	12.226	2
2	T2DM without ED	507,938	38,386	4.707	12.321	2

	t	df	p
Test Statistics	-0.234	81667	0.815

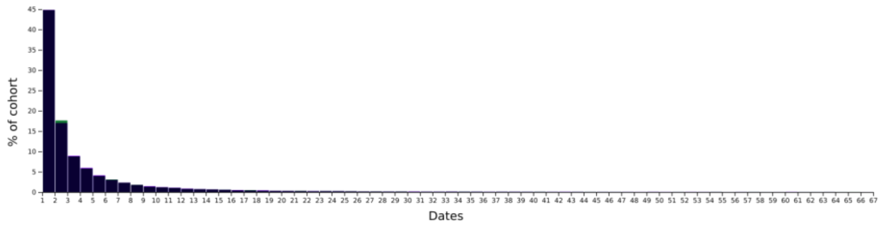

208 data points for Cohort 1 and 194 data points for Cohort 2 were omitted for display purposes.
58,000 patients in Cohort 1 and 47,570 patients in Cohort 2 were excluded from results because they had the outcome prior to the time window.

5 CVD

Risk analysis excluding patients with outcome prior to the time window

Cohort		Patients in cohort	Patients with outcome	Risk
1	T2DM with ED	321,207	82,829	0.258
2	T2DM without ED	311,504	73,660	0.236

		95% CI	z	p
Risk Difference	0.021	(0.019, 0.024)	19.726	0.000
Risk Ratio	1.091	(1.081, 1.100)	N/A	N/A
Odds Ratio	1.122	(1.109, 1.135)	N/A	N/A

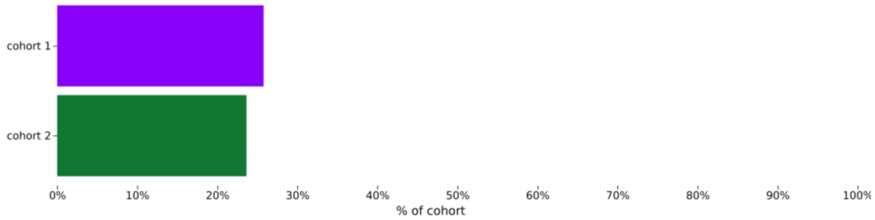

234,301 patients in Cohort 1 and 244,004 patients in Cohort 2 were excluded from results because they had the outcome prior to the time window.

Kaplan - Meier survival analysis excluding patients with outcome prior to the time window

Cohort		Patients in cohort	Patients with outcome	Median survival (days)	Survival probability at end of time window
1	T2DM with ED	321,207	82,829	3757	18.03%
2	T2DM without ED	311,504	73,660	3788	23.54%

	χ^2	df	p
Log-Rank Test	47.649	1	0.000

	Hazard Ratio	95% CI	χ^2	df	p
Hazard Ratio and Proportionality	0.966	(0.956, 0.975)	160.987	1	0.000

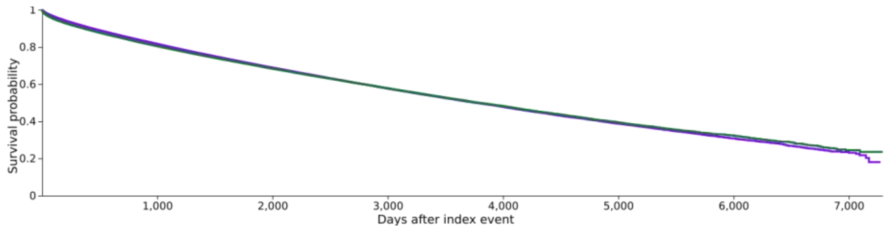

234,301 patients in Cohort 1 and 244,004 patients in Cohort 2 were excluded from results because they had the outcome prior to the time window.

Number of instances excluding patients with outcome prior to the time window

Cohort		Patients in cohort	Patients with outcome	Mean	Standard Deviation	Median
1	T2DM with ED	321,207	82,829	9.571	18.322	3
2	T2DM without ED	311,504	73,660	8.616	17.401	3

	t	df	p
Test Statistics	10.536	156487	0.000

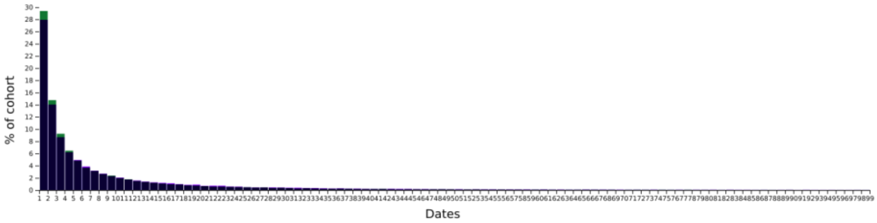

481 data points for Cohort 1 and 374 data points for Cohort 2 were omitted for display purposes.
234,301 patients in Cohort 1 and 244,004 patients in Cohort 2 were excluded from results because they had the outcome prior to the time window.

9 Appendix A – Text Representation of the Cohorts Definition

This section lists all terms used in the definitions of the two cohorts.

9.1.1 Query Criteria for Cohort 1 (query name: T2DM with ED)

Patients must have:
- all of the following:
  - Age (Age) (at least 18 years (most recent occurrence)); and
  - Male (UMLS:HL7V3.0:Gender:M); and
  - Type 2 diabetes mellitus (UMLS:ICD10CM:E11) (between 20 and 80 years old at event); and
  - any of the following:
    - Male erectile dysfunction (UMLS:ICD10CM:N52); or
    - Vasculogenic erectile dysfunction (UMLS:ICD10CM:N52.0); or
    - Other male erectile dysfunction (UMLS:ICD10CM:N52.8); or
    - Male erectile dysfunction, unspecified (UMLS:ICD10CM:N52.9); or
    - Corporo-venous occlusive erectile dysfunction (UMLS:ICD10CM:N52.02); or
    - Drugs used in erectile dysfunction (NLM:ATC:G04BE); or
    - Vascular disorders of male genital organs (UMLS:ICD10CM:N50.1); or
    - Erectile dysfunction due to diseases classified elsewhere (UMLS:ICD10CM:N52.1); or
    - Erectile dysfunction due to arterial insufficiency (UMLS:ICD10CM:N52.01).

Patients cannot have:
- any of the following:
  - Diabetes mellitus due to underlying condition (UMLS:ICD10CM:E08); or
  - Drug or chemical induced diabetes mellitus (UMLS:ICD10CM:E09); or
  - Type 1 diabetes mellitus (UMLS:ICD10CM:E10); or
  - Other specified diabetes mellitus (UMLS:ICD10CM:E13).

9.1.2 Query Criteria for Cohort 2 (query name: T2DM without ED)

Patients must have:
- all of the following:
  - Age (Age) (at least 18 years (most recent occurrence)); and
  - Male (UMLS:HL7V3.0:Gender:M); and
  - Type 2 diabetes mellitus (UMLS:ICD10CM:E11) (between 20 and 80 years old at event).

Patients cannot have:
- any of the following:
  - Diabetes mellitus due to underlying condition (UMLS:ICD10CM:E08); or
  - Drug or chemical induced diabetes mellitus (UMLS:ICD10CM:E09); or
  - Type 1 diabetes mellitus (UMLS:ICD10CM:E10); or
  - Other specified diabetes mellitus (UMLS:ICD10CM:E13); or

Male erectile dysfunction (UMLS:ICD10CM:N52); or
Vasculogenic erectile dysfunction (UMLS:ICD10CM:N52.0); or
Other male erectile dysfunction (UMLS:ICD10CM:N52.8); or
Corporo-venous occlusive erectile dysfunction (UMLS:ICD10CM:N52.02); or
Male erectile dysfunction, unspecified (UMLS:ICD10CM:N52.9); or
Drugs used in erectile dysfunction (NLM:ATC:G04BE); or
Erectile dysfunction due to arterial insufficiency (UMLS:ICD10CM:N52.01); or
Vascular disorders of male genital organs (UMLS:ICD10CM:N50.1); or
Erectile dysfunction due to diseases classified elsewhere (UMLS:ICD10CM:N52.1).

10 Appendix B – Text Representation of the Analysis Setup

This section contains the Index Event definition for each cohort.

The index event for Cohort 1 (query name: T2DM with ED) is defined as the following:

Patients must have:
all of the following:
Type 2 diabetes mellitus (UMLS:ICD10CM:E11) (between 20 and 80 years old at event); and
any of the following:
Male erectile dysfunction (UMLS:ICD10CM:N52); or
Vasculogenic erectile dysfunction (UMLS:ICD10CM:N52.0); or
Other male erectile dysfunction (UMLS:ICD10CM:N52.8); or
Male erectile dysfunction, unspecified (UMLS:ICD10CM:N52.9); or
Corporo-venous occlusive erectile dysfunction (UMLS:ICD10CM:N52.02); or
Drugs used in erectile dysfunction (NLM:ATC:G04BE); or
Vascular disorders of male genital organs (UMLS:ICD10CM:N50.1); or
Erectile dysfunction due to diseases classified elsewhere (UMLS:ICD10CM:N52.1); or
Erectile dysfunction due to arterial insufficiency (UMLS:ICD10CM:N52.01).

The index event for Cohort 2 (query name: T2DM without ED) is defined as the following:

Patients must have:
Type 2 diabetes mellitus (UMLS:ICD10CM:E11) (between 20 and 80 years old at event).

10.1.1

11 Appendix C – Text Representation of the Outcomes Definition

This analysis includes the following outcomes:

Ischemic hearth disease
Patients must have:
Ischemic heart diseases (UMLS:ICD10CM:I20-I25).

Stroke
Patients must have:
Cerebrovascular diseases (UMLS:ICD10CM:I60-I69).

Heart failure
Patients must have:
Heart failure (UMLS:ICD10CM:I50).

Peripheral artery disease
Patients must have:
Diseases of veins, lymphatic vessels and lymph nodes, not elsewhere classified (UMLS:ICD10CM:I80-I89).

CVD
Patients must have:
any of the following:
Ischemic heart diseases (UMLS:ICD10CM:I20-I25); or
Cerebrovascular diseases (UMLS:ICD10CM:I60-I69); or
Diseases of veins, lymphatic vessels and lymph nodes, not elsewhere classified (UMLS:ICD10CM:I80-I89); or
Heart failure (UMLS:ICD10CM:I50).
